# Design, Synthesis, Characterization, and Cytotoxicity of New Pyrazolylmethylene-2-thioxoimidazolidin-4-one Derivatives towards Androgen-Sensitive LNCaP Prostate Cancer Cells

**DOI:** 10.3390/biom14070811

**Published:** 2024-07-08

**Authors:** Mohamed A. El-Atawy, Rashad Kebeish, Awatif Rashed Z. Almotairy, Alaa Z. Omar

**Affiliations:** 1 Chemistry Department, Faculty of Science, Taibah University, Yanbu 46423, Saudi Arabia; amotairy@taibahu.edu.sa; 2 Chemistry Department, Faculty of Science, Alexandria University, P.O. 426 Ibrahemia, Alexandria 21321, Egypt; alaazaki@alexu.edu.eg; 3 Department of Biology, Faculty of Science in Yanbu, Taibah University, Yanbu 46423, Saudi Arabia; 4 Botany and Microbiology Department, Faculty of Science, Zagazig University, Zagazig 44519, Egypt

**Keywords:** prostate cancer, PC3, LNCaP, pyrazole, imidazole, androgen receptors blockers

## Abstract

A new class of pyrazolylmethylene-2-thioxoimidazolidin-4-one derivatives **3a–p** were rationally designed and synthesized with the aim of exploring their potential as treatments for prostate cancer. The synthesized compounds **3a–p** were biologically analyzed for their anticancer effects against AR^+^LNCaP, AR^-^PC-3, and Wi38 cell lines. The observed IC_50_ values against AR^+^LNCaP ranged between 10.27 ± 0.14 and 109.72 ± 2.06 µM after 24 h of incubation. Compounds **3i–k**, **3m**, and **3o–p** recorded IC_50_ values of 05.22 ± 0.12 to 11.75 ± 0.07 µM after 48 h incubation in the presence of 1 nM DHT, with higher selectivity towards AR^+^LNCaP. Moreover, compounds **3i** and **3k** significantly induced Caspase 3 accumulation, reduced DNA content at the various stages of the cell cycle, and ultimately caused AR^+^LNCaP cell growth arrest, as confirmed by cell apoptosis assays. These findings suggest that these analogues of androgen receptor blockers have promising potential for further investigation as effective treatments for prostate cancer.

## 1. Introduction

Global prostate cancer is growing. It is the second most common malignancy and fifth leading cause of mortality in men [1]. Most prostate cancer metastases occur in lymph nodes and bones [2]. The treatment of prostate cancer now encompasses a variety of methods [3]. Hormone therapy is a common treatment that shrinks tumors or slows their growth. It works by reducing testosterone, the “fuel” for prostate cancer cells. Hormone therapy has two main approaches. The first approach, known as androgen suppression therapy [4], lowers testosterone production either through surgery (testicle removal) [5] or medications (LHRH agonists/antagonists). While surgery is permanent, medications can cause temporary side effects like testosterone flares [6].

LHRH agonists block body signals to generate testosterone in the testicles. Thus, LHRH agonists reduce testosterone. Among these drugs, Leuprolide (Lupron, Eligard) [7], Goserelin (Zoladex) [8], Triptorelin (Trelstar) [9], and Histrelin (Vantas) [10] are employed. LHRH antagonists such as Degarelix (Firmagon) [11] work in the same way as LHRH agonists; however, they decrease testosterone levels more rapidly without flare effects.

**Figure 1:**
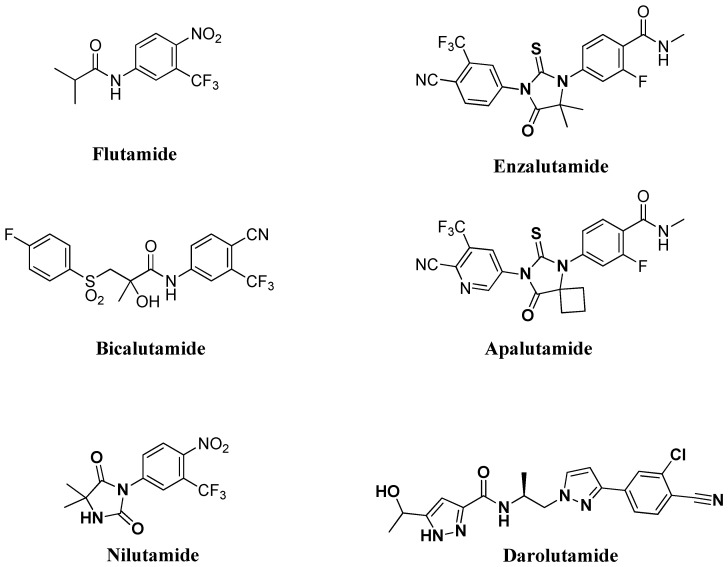
Some examples of androgen receptor blockers.

The second approach is androgen receptor blockers (Figure 1) that rely on blocking the actions of androgens via binding to “androgen receptors”. These drugs include Flutamide (Eulexin), Bicalutamide (Casodex), and Nilutamide (Nilandron). Moreover, recent androgen receptor blockers also exist, including Enzalutamide (Xtandi), apalutamide (Erleada), and darolutamide (Nubeqa). These drugs may cause nervous system side effects such as dizziness and, rarely, seizures. Combination therapy, which is called combined androgen blockade (CAB), using both approaches can further suppress testosterone.

Imidazolidine-2,4-dione derivatives are a class of compounds that have been shown as potential anticancer agents in various studies [12,13,14,15,16]. They can interfere with cell cycle progression of cancer cells, leading to their growth arrest at various stages, particularly at G1 and S phases [17]. Thus, they can disrupt DNA repair mechanisms, leading to great DNA damage, and ultimately induce cell death [18]. Moreover, such derivatives have also been shown to inhibit angiogenesis [19], thereby reducing the blood supply to tumors, inhibiting their growth, and preventing metastasis. They can additionally induce apoptosis in cancer cells by activating certain cellular pathways involved in this process [20].

**Figure 2:**
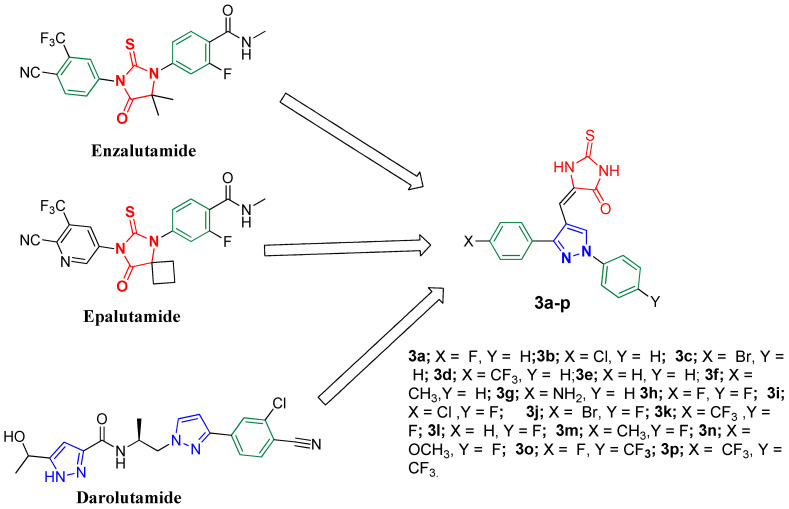
Design of the target hybrid compounds **3a–p.**

In line with our previous work to look for effective analogs of androgen receptor blockers [21], the current study aims to develop hybrid pharmacophores based on a combination of different pharmacophoric moieties, namely, 2-thioxoimidazolidin-4-one and pyrazole scaffolds that exist in FDA-approved prostate cancer drugs as enzalutamide and darolutamide; respectively (Figure 2). Moreover, the pyrazole pharmacophore of the designed target molecules is attached to two aromatic rings that possess a variety of substituents. These substituents include electron-donating groups such as Me, OMe, and NH_2_, as well as electron-attracting groups such as CF_3_, F, Cl, and Br, thus modulating both electronic and steric properties of the target compounds.

The synthesized compounds were characterized with different spectroscopic techniques such as IR, ^1^H-NMR, ^13^C-NMR, and elemental analysis. Moreover, these compounds were biologically tested for their cytotoxic effects against androgen-sensitive (AR^+^LNCaP) and androgen-insensitive (AR^-^PC-3) human prostate cancer cell lines, in addition to normal human lung fibroblast (Wi38) as control. Caspase 3 accumulation, cell apoptosis, and DNA contents measurements at variable stages of the cell cycle were also performed to ensure the effectiveness of the designed compounds as potential anti-prostate-cancer drugs.

## 2. Materials and Methods

### 2.1. Materials

All the chemicals used were of analytical grade. Acetic acid, acetophenones, phenylhydrazines, 2-thioxoimidazolidin-4-one, ethanol, and DMF were purchased from Sigma (St. Louis, MO, USA) and Sigma-Aldrich (Germany; Darmstadt), and were used as received. The Invitrogen Elisa kit for detection of active human caspase-3 (Invitrogen, Camarillo, CA, USA) was used to evaluate Caspase 3 in tumor cells. The Ab139418–Propidium Iodide Flow Cytometry Kit for Cell Cycle Analysis (ab139418, Abcam, Cambridge, UK) was employed to measure DNA contents of AR^+^LNCaP cells stimulated with 1 nM DHT and treated with test compounds **3i** and **3k**. The BioVision Annexin V-FITC apoptosis detection kit (BioVision, Cambridge, UK) was applied to perform cell apoptosis assay.

### 2.2. Measurements

Melting points were determined on a Koffler block and are uncorrected. A Perkin Elmer-USA Spectrometer was used for recording FT-IR spectra using KBr discs at room temperature within the wave number range of 4000 to 400 cm^−1^. ^1^H-NMR was recorded on a JEOL JNM ECA 500 MHz instrument (JEOL Ltd., Tokyo, Japan) using in DMSO-d^6^ as a solvent at 25 °C. Chemical shifts (δ) are expressed in part per million (ppm).

### 2.3. Synthesis of the Compounds under Investigation

#### 2.3.1. General Method for Synthesis of 1,3-Diaryl-1H-pyrazole-4-carbaldehyde **2a–p**

Anhydrous dimethyl formamide (2.58 g, 2.73 mL, 0.03 mol) was added to a flame-dried flask under nitrogen then cooled to 0 °C. Phosphorus oxychloride (5.40 g, 3.28 mL, 0.03 mol) was added and the mixture allowed to stir for 15 min at 0 °C. A solution of acetophenone phenylhydrazone derivatives **1a–p** (0.01 mol) in anhydrous DMF (3 mL) was added dropwise to the reaction mixture and heated at 75 °C for 5 h. The reaction mixture was cooled and a solution of 10% sodium carbonate (2 mL) was added. The obtained precipitate was filtered, washed with water (15 mL), dried, and crystallized from ethanol. The supporting information are shown in Supplementary Materials.

3-(4-Fluorophenyl)-1-phenyl-1*H*-pyrazole-4-carbaldehyde **2a** [22]

Colorless crystals, 2.34 g (88%) yield; m.p. 164–166 °C. IR (KBr): ῡ 3068 (S_P_^2^ =C-H), 2837–2780 (-CO-H), 1672 (C=O) and 1598 (C=N) cm^−1^. ^1^H-NMR (400 MHz, DMSO): δ 9.98 (s, 1H, CHO), 9.37 (s, 1H, Pyrazol-H), 8.08–7.91 (m, 4H, Ar-H), 7.59 (t, *J* = 7.9 Hz, 2H, Ar-H), 7.45 (t, *J* = 7.4 Hz, 1H, Ar-H) and 7.36 (t, *J* = 8.9 Hz, 2H, Ar-H) ppm. ^13^C-NMR (101 MHz, DMSO): δ 184.99 (HCO), 163.17 (d, ^1^*J*c_,F_ = 246.5 Hz, C), 151.88 (C), 139.07 (C), 136.04 (CH), 131.34 (d, ^3^*J*c_,F_ = 8.5 Hz, CH), 130.21 (CH), 128.24 (CH), 122.53 (C), 119.70 (CH), 116.05 (CH) and 115.94 (d, ^2^*J*c_,F_ = 21.7 Hz, CH) ppm.

3-(4-Chlorophenyl)-1-phenyl-1*H*-pyrazole-4-carbaldehyde **2b** [23]

Colorless crystals, 2.45 g (87%) yield; m.p. 111–113 °C. IR (KBr): ῡ 3063 (S_P_^2^ = C-H), 2830–2778 (-CO-H), 1670 (C=O) and 1596 (C=N) cm^−1^. ^1^H-NMR (400 MHz, DMSO): δ 9.98 (s, 1H, CHO), 9.37 (s, 1H, Pyrazol-H), 7.99 (m, 5H, Ar-H), 7.57 (m, 4H, Ar-H) and 7.44 (m, *J* = 5.7 Hz, 1H, Ar-H) ppm. ^13^C-NMR (101 MHz, DMSO): δ 184.91 (HCO), 151.54 (C), 138.96 (C), 136.26 (CH), 134.44 (C), 130.81 (CH), 130.60 (C), 130.18 (CH), 129.01 (CH), 128.26 (CH), 122.64 (C) and 119.69 (CH) ppm.

3-(4-Bromophenyl)-1-phenyl-1*H*-pyrazole-4-carbaldehyde **2c** [24]

Colorless crystals, 2.91 g (89%) yield; m.p. 139–140 °C. IR (KBr): ῡ 3121 (S_P_^2^ =C-H), 2827–2771 (-CO-H), 1674 (C=O) and 1595 (C=N) cm^−1^. ^1^H-NMR (400 MHz, DMSO): δ 10.00 (s, 1H, CHO), 9.39 (s, 1H, Pyrazol-H), 8.00 (d, *J* = 7.9 Hz, 2H, Ar-H), 7.95 (d, *J* = 8.4 Hz, 2H, Ar-H), 7.73 (d, *J* = 8.4 Hz, 2H, Ar-H), 7.60 (t, *J* = 7.8 Hz, 2H, Ar-H) and 7.46 (t, *J* = 7.3 Hz, 1H, Ar-H) ppm. ^13^C-NMR (101 MHz, DMSO) δ 184.98 (HC=O), 151.57 (C), 138.99 (C), 136.24 (CH), 132.00 (CH), 131.11 (CH), 130.97 (C), 130.24 (CH), 128.32 (CH), 123.18 (C), 122.65 (C) and 119.75 (CH) ppm.

1-Phenyl-3-(4-(trifluoromethyl)phenyl)-1*H*-pyrazole-4-carbaldehyde **2d** [25]

Colorless crystals, 2.93 g (93%) yield; m.p. 144–145 °C. IR (KBr): ῡ 3070 (S_P_^2^ = C-H), 2840–2781 (-CO-H), 1678 (C=O) and 1597 (C=N) cm^−1^. ^1^H-NMR (400 MHz, DMSO): δ 10.02 (s, 1H, CHO), 9.43 (s, 1H, Pyrazol-H), 8.20 (d, *J* = 8.0 Hz, 2H, Ar-H), 8.02 (d, *J* = 7.9 Hz, 2H, Ar-H), 7.89 (d, *J* = 8.1 Hz, 2H, Ar-H), 7.61 (t, *J* = 7.8 Hz, 2H, Ar-H) and 7.47 (t, *J* = 7.3 Hz, 1H, Ar-H) ppm. ^13^C-NMR (101 MHz, DMSO): δ 184.96 (HC=O), 151.21 (C), 138.95 (C), 136.49 (CH), 135.76 (C), 130.25 (CH), 129.84 (CH), 129.78(q, ^2^*J*c_,F_ = 32.0 Hz, C), 128.43 (CH), 127.37 (q, ^1^*J*c_,F_ = 273.3 Hz, CF_3_), 125.87 (q, ^3^*J*c_,F_ = 3.6 Hz, CH), 120.61 (C) and 119.81 (CH) ppm.

1,3-Diphenyl-1H-pyrazole-4-carbaldehyde **2e** [22]

Colorless crystals, 2.28 g (92%) yield; m.p. 138–140 °C. IR (KBr): ῡ 3061 (S_P_^2^ = C-H), 2865–2837 (-CO-H), 1673 (C=O) and 1597 (C=N) cm^−1^. ^1^H-NMR (400 MHz, DMSO): δ 10.00 (s, 1H, CHO), 9.34 (s, 1H, Pyrazol-H), 8.01 (d, *J* = 7.7 Hz, 2H, Ar-H), 7.94 (d, *J* = 6.4 Hz, 2H, Ar-H), 7.58 (t, *J* = 7.6 Hz, 2H, Ar-H), 7.52 (d, *J* = 7.0 Hz, 3H, Ar-H) and 7.44 (t, *J* = 7.1 Hz, 1H, Ar-H) ppm. ^13^C-NMR (101 MHz, DMSO): δ 185.08 (HC=O), 153.15 (C), 139.09 (C), 131.75 (CH), 130.18 (C), 129.65 (CH), 129.17 (CH), 129.01 (CH), 128.20 (CH), 122.65 (C) and 119.74 (CH) ppm.

1-Phenyl-3-(*p*-tolyl)-1*H*-pyrazole-4-carbaldehyde **2f** [25]

Colorless crystals, 2.38 g (91%) yield; m.p. 120–122 °C. IR (KBr): ῡ 3032 (S_P_^2^ =C-H), 2921 (S_P_^3^ = C-H), 2835–2779 (-CO-H), 1671 (C=O) and 1598 (C=N) cm^−1^. ^1^H-NMR (400 MHz, DMSO): δ 9.99 (s, 1H, CHO), 9.32 (s, 1H, Pyrazol-H), 8.01 (d, *J* = 8.0 Hz, 2H, Ar-H), 7.84 (d, *J* = 7.8 Hz, 2H, Ar-H), 7.58 (t, *J* = 7.7 Hz, 2H, Ar-H), 7.44 (t, *J* = 7.3 Hz, 1H, Ar-H), 7.33 (d, *J* = 7.8 Hz, 2H, Ar-H) and 2.39 (s, 3H, CH_3_) ppm. ^13^C- NMR (101 MHz, DMSO): δ 185.14 (HCO), 153.21 (C), 139.23 (C), 139.09 (C), 135.21 (CH), 130.19 (CH), 129.61 (CH), 129.06 (CH), 128.90 (C), 128.16 (CH), 122.57 (C), 119.70 (CH) and 21.39 (CH_3_) ppm.

3-(4-Aminophenyl)-1-phenyl-1*H*-pyrazole-4-carbaldehyde **2g** [26]

Colorless crystals, 1.97 g (75%) yield; m.p. 105–107 °C. IR (KBr): ῡ 3343 (NH_2_), 3058 (S_P_^2^ = C-H), 2815–2767 (-CO-H), 1685 (C=O) and 1597 (C=N) cm^−1^. ^1^H-NMR (400 MHz, DMSO): δ 9.97 (s, 1H, CHO), 9.91 (s, 2H, NH_2_), 9.29 (s, 1H, Pyrazol-H), 7.99 (d, *J* = 7.8 Hz, 2H, Ar-H), 7.80 (d, *J* = 8.6 Hz, 2H, Ar-H), 7.58 (t, *J* = 7.9 Hz, 2H, Ar-H), 7.43 (t, *J* = 7.4 Hz, 1H, Ar-H) and 6.92 (d, *J* = 8.6 Hz, 2H, Ar-H) ppm. ^13^C-NMR (101 MHz, DMSO): δ 185.19 (HC=O), 158.98 (C), 153.39 (C), 139.12 (C), 135.14 (CH), 130.57 (CH), 130.17 (CH), 128.03 (CH), 122.47 (C), 122.33 (C), 119.61 (CH) and 115.83 (CH) ppm.

1,3-Bis(4-fluorophenyl)-1*H*-pyrazole-4-carbaldehyde **2h** [27]

Colorless crystals, 2.32 g (82%) yield; m.p. 145–147 °C. IR (KBr): ῡ 3125 (S_P_^2^ =C-H), 2871–2839 (-CO-H), 1675 (C=O) and 1601 (C=N) cm^−1^. ^1^H-NMR (400 MHz, DMSO): δ 9.98 (s, 1H, CHO), 9.34 (s, 1H, Pyrazol-H), 8.03 (td, *J* = 8.6, 5.3 Hz, 4H, Ar-H) and 7.40 (dt, *J* = 33.6, 8.8 Hz, 4H, Ar-H) ppm. ^13^C-NMR (101 MHz, DMSO): δ 184.99 (HC=O), 163.62 (d, ^1^*J*c_,F_ = 159.3 Hz, CF), 161.18 (d, ^1^*J*c_,F_ = 157.7 Hz, CF), 151.97 (C), 136.11 (CH), 135.61 (C), 131.36 (d, ^3^*J*c_,F_ = 8.4 Hz, CH^/^), 128.17 (C), 122.54 (C), 121.97 (d, ^3^*J*c_,F_ = 8.7 Hz, CH), 117.00 (d, ^2^*J*c_,F_ = 23.2 Hz, CH), 115.96 (d, ^2^*J*c_,F_ = 21.5 Hz, CH) ppm.

3-(4-Chlorophenyl)-1-(4-fluorophenyl)-1*H*-pyrazole-4-carbaldehyde **2i** [28]

Colorless crystals, 1.92 g (64%) yield; m.p. 122–124 °C. IR (KBr): ῡ 3028 (S_P_^2^ = C-H), 2865–2832 (-CO-H), 1674 (C=O) and 1571 (C=N) cm^−1^. ^1^H-NMR (400 MHz, DMSO): δ 9.98 (s, 1H, CHO), 9.35 (s, 1H, Pyrazol-H), 8.04 (dd, *J* = 9.1, 4.7 Hz, 2H, Ph-H_2_ and H_6_), 8.00 (d, *J* = 8.5 Hz, 2H, Ph-H_2_^/^ and H_6_^/^), 7.59 (d, *J* = 8.5 Hz, 2H, Ph-H_3_^/^ and H_5_^/^) and 7.44 (t, *J* = 8.8 Hz, 2H, Ph-H_3_ and H_5_) ppm. ^13^C-NMR (101 MHz, DMSO): δ 184.96 (HC=O), 161.64 (d, ^1^*J*c_,F_ = 244.9 Hz, CF), 151.67 (C), 136.30 (CH), 135.56 (C), 134.48 (C), 130.85 (CH), 130.52 (C), 129.15 (CH), 122.66 (C), 122.00 (d, ^3^*J*c_,F_ = 8.7 Hz, Ph-C_2_) and 117.01 (d, ^2^*J*c_,F_ = 23.3 Hz, Ph-C_3_) ppm.

3-(4-Bromophenyl)-1-(4-fluorophenyl)-1*H*-pyrazole-4-carbaldehyde **2j**

Colorless crystals, 3.00 g (87%) yield; m.p. 168–170 °C. IR (KBr): ῡ 3024 (S_P_^2^ = C-H), 2863–2829 (-CO-H), 1674 (C=O) and 1567 (C=N) cm^−1^. ^1^H-NMR (400 MHz, DMSO): δ 9.98 (s, 1H, CHO), 9.35 (s, 1H, Pyrazol-H), 8.04 (dd, *J* = 8.8, 4.6 Hz, 2H, Ph-H_2_ and H_6_), 7.93 (d, *J* = 8.4 Hz, 2H, Ph-H_2_^/^ and H_6_^/^), 7.73 (d, *J* = 8.3 Hz, 2H, Ph-H_3_^/^ and H_5_^/^) and 7.45 (t, *J* = 8.7 Hz, 2H, Ph-H_3_ and H_5_) ppm. ^13^C-NMR (101 MHz, DMSO): δ 184.97 (HC=O), 161.65 (d, ^1^*J*c_,F_ = 245.0 Hz, CF), 151.75 (C), 136.30 (CH), 135.58 (C), 132.01 (CH), 131.11 (CH), 130.88 (C), 123.21 (C), 122.65 (C), 122.01 (d, ^3^*J*c_,F_ = 8.7 Hz, CH) and 117.02 (d, ^2^*J*c_,F_ = 23.2 Hz, CH) ppm. C_16_H_10_BrFN_2_O requires C, 55.68; H, 2.92; N, 8.12%. Found: C, 55.61; H, 3.02; N, 8.19.

1-(4-Fluorophenyl)-3-(4-(trifluoromethyl)phenyl)-1H-pyrazole-4-carbaldehyde **2k**

Colorless crystals, 3.03 g (91%) yield; m.p. 153–155 °C. IR (KBr): ῡ 3032 (S_P_^2^ = C-H), 2871–2842 (-CO-H), 1674 (C=O) and 1618 (C=N) cm^−1^. ^1^H-NMR (400 MHz, DMSO): δ 10.00 (s, 1H, CHO), 9.37 (s, 1H, Pyrazol-H), 8.17 (d, *J* = 8.0 Hz, 2H, Ph-H_2_^/^ and H_6_^/^), 8.03 (dd, *J* = 8.5, 4.5 Hz, 2H, Ph-H_2_ and H_6_), 7.86 (d, *J* = 8.0 Hz, 2H, Ph-H_3_^/^ and H_5_^/^) and 7.43 (t, *J* = 8.6 Hz, 2H, Ph-H_3_ and H_5_) ppm. ^13^C-NMR (101 MHz, DMSO): δ 184.88 (HC=O), 161.70 (d, ^1^*J*c_,F_ = 245.1 Hz, CF), 151.24 (C), 136.51 (CH), 135.65 (C), 129.8 (q, ^2^*J*c_,F_ = 32.3 Hz, C), 129.81 (CH), 125.84 (q, ^3^*J*c_,F_ = 4.0 Hz, CH), 124.63 (q, ^1^*J*c_,F_ = 273.2 Hz, CF_3_), 122.88 (C), 122.02 (d, ^3^*J*c_,F_ = 8.7 Hz, CH) and 117.00 (d, ^2^*J*c_,F_ = 23.3 Hz, CH) ppm. C_17_H_10_F_4_N_2_O requires C, 61.08; H, 3.02; N, 8.38%. Found: C, 61.15; H, 3.08; N, 8.29.

1-(4-Fluorophenyl)-3-phenyl-1H-pyrazole-4-carbaldehyde **2l** [29]

Colorless crystals, 2.15 g (81%) yield; m.p. 171–173 °C. IR (KBr): ῡ 3062 (S_P_^2^ = C-H), 2870–2838 (-CO-H), 1673 (C=O) and 1583 (C=N) cm^−1^. ^1^H-NMR (400 MHz, DMSO): δ 9.99 (s, 1H, CHO), 9.32 (s, 1H, Pyrazol-H), 8.05 (dd, *J* = 9.0, 4.7 Hz, 2H, Ph-H_2_ and H_6_), 7.93 (d, *J* = 7.2 Hz, 2H, Ph-H_2_^/^ and H_6_^/^), 7.59–7.47 (m, 3H, Ph-H_3_^/^, H_4_^/^ and H_5_^/^) and 7.44 (t, *J* = 8.8 Hz, 2H, Ph-H_3_ and H_5_) ppm. ^13^C-NMR (101 MHz, DMSO): δ 185.12 (HC=O), 161.59 (d, ^1^*J*c_,F_ = 244.7 Hz, CF), 153.22 (C), 135.63 (C), 135.42 (CH), 131.64 (C), 129.69 (CH), 129.17 (CH), 129.04 (CH), 122.63 (C), 121.95 (d, ^3^*J*c_,F_ = 8.7 Hz, CH) and 116.98 (d, ^2^*J*c_,F_ = 23.3 Hz, CH) ppm.

1-(4-Fluorophenyl)-3-(*p*-tolyl)-1*H*-pyrazole-4-carbaldehyde **2m** [30]

Colorless crystals, 2.46 g (88%) yield; m.p. 143–144 °C. IR (KBr): ῡ 3031 (S_P_^2^ = C-H), 2922 (S_P_^3^ -C-H), 2869–2838 (-CO-H), 1674 (C=O) and 1573 (C=N) cm^−1^. ^1^H-NMR (400 MHz, DMSO): δ 9.98 (s, 1H, CHO), 9.28 (s, 1H, Pyrazol-H), 8.03 (s, 2H, Ph-H_2_ and H_6_), 7.82 (d, *J* = 7.2 Hz, 2H, Ph-H_2_^/^ and H_6_^/^), 7.43 (t, *J* = 7.9 Hz, 2H, Ph-H_3_ and H_5_), 7.32 (d, *J* = 7.0 Hz, 2H, Ph-H_3_^/^ and H_5_^/^) and 2.38 (s, 3H, CH_3_) ppm. ^13^C-NMR (101 MHz, DMSO): δ 185.10 (HC=O), 161.55 (d, ^1^*J*c_,F_ = 244.8 Hz, CF), 153.27 (C), 139.24 (C), 135.64 (C), 135.24 (CH), 129.61 (CH), 129.05 (CH), 128.81 (C), 122.57 (C), 121.89 (d, ^3^*J*c_,F_ = 8.7 Hz, CH), 116.93 (d, ^2^*J*c_,F_ = 23.1 Hz, CH) and 21.37 (CH_3_) ppm.

1-(4-Fluorophenyl)-3-(4-methoxyphenyl)-1H-pyrazole-4-carbaldehyde **2n** [31]

Colorless crystals, 2.81 g (95%) yield; m.p. 144–146 °C. IR (KBr): ῡ 3035 (S_P_^2^ = C-H), 2998 (S_P_^3^ -C-H), 2868–2838 (-CO-H), 1674 (C=O) and 1610 (C=N) cm^−1^. ^1^H-NMR (400 MHz, DMSO): δ 9.97 (s, 1H, CHO), 9.29 (s, 1H, Pyrazol-H), 8.04 (dd, *J* = 7.6, 6.0 Hz, 2H, Ph-H_2_ and H_6_), 7.91 (d, *J* = 8.6 Hz, 2H, Ph-H_2_^/^ and H_6_^/^), 7.43 (t, *J* = 8.7 Hz, 2H, Ph-H_3_ and H_5_), 7.08 (d, *J* = 8.7 Hz, 2H, Ph-H_3_^/^ and H_5_^/^) and 3.84 (s, 3H, CH_3_) ppm. ^13^C-NMR (101 MHz, DMSO): δ 185.08 (HC=O), 161.65 (d, ^1^*J*c_,F_ = 239.6 Hz, CF), 160.56 (C), 152.97 (C), 135.69 (C), 135.59 (CH), 130.53 (CH), 124.01 (C), 122.44 (C), 121.86 (d, ^3^*J*c_,F_ = 8.6 Hz, CH), 116.96 (d, ^2^*J*c_,F_ = 23.1 Hz, CH), 114.45 (CH) and 55.72 (CH_3_) ppm.

3-(4-Fluorophenyl)-1-(4-(trifluoromethyl)phenyl)-1H-pyrazole-4-carbaldehyde **2o**

Colorless crystals, 3.03 g (91%) yield; m.p. 154–156 °C. IR (KBr): ῡ 3040 (S_P_^2^ = C-H), 2870–2845 (-CO-H), 1680 (C=O) and 1613 (C=N) cm^−1^. ^1^H-NMR (400 MHz, DMSO): δ 9.97 (s, 1H, CHO), 9.49 (s, 1H, Pyrazol-H) and 8.50–7.19 (m, 8H, Ar-H) ppm. ^13^C-NMR (101 MHz, DMSO): δ 185.02 (HC=O), 163.28 (d, ^1^*J*c_,F_ = 246.6 Hz, CF), 152.39 (C), 141.78 (C), 136.71 (CH), 131.41 (d, ^3^*J*c_,F_ = 8.3 Hz, CH), 127.84 (q, ^2^*J*c_,F_ = 31.3 Hz, C), 127.48 (q, ^3^*J*c_,F_ = 2.0 Hz, CH), (CH), 124.40 (q, ^1^*J*c_,F_ = 276.4 Hz, CF_3_), 120.05 (CH) and 115.99 (d, ^2^*J*c_,F_ = 21.6 Hz, CH) ppm. C_17_H_10_F_4_N_2_O requires C, 61.08; H, 3.02; N, 8.38%. Found: C, 61.01; H, 2.95; N, 8.44.

1,3-Bis(4-(trifluoromethyl)phenyl)-1*H*-pyrazole-4-carbaldehyde **2p** [32]

Colorless crystals, 3.00 g (90%) yield; m.p. 117–119 °C. IR (KBr): ῡ 3130 (S_P_^2^ = C-H), 2869–2843 (-CO-H), 1677 (C=O) and 1618 (C=N) cm^−1^. ^1^H-NMR (400 MHz, DMSO): δ 10.01 (s, 1H, CHO), 9.54 (s, 1H, Pyrazol-H), 8.22 (d, *J* = 8.3 Hz, 2H, Ar-H), 8.18 (d, *J* = 7.9 Hz, 2H, Ar-H), 7.95 (d, *J* = 8.4 Hz, 2H, Ar-H) and 7.86 (d, *J* = 8.0 Hz, 2H, Ar-H) ppm. ^13^C-NMR (101 MHz, DMSO): δ 184.91 (HC=O), 151.68 (C), 141.66 (C), 137.10 (CH), 135.40 (C), 129.95(q, ^2^*J*c_,F_ = 32.2 Hz, C), 129.84 (CH), 128.32 (q, ^2^*J*c_,F_ = 32.3 Hz, C), 124.60 (q, ^1^*J*c_,F_ = 273.3 Hz, CF_3_), 124.38 (q, ^1^*J*c_,F_ = 273.0 Hz, CF_3_), 123.33 (C), 127.47 (q, ^3^*J*c_,F_ = 3.7 Hz, CH), 125.84 (q, ^3^*J*c_,F_ = 3.7 Hz, CH), and 120.10 (CH) ppm.

#### 2.3.2. General Method for Synthesis of 5-((1,3-Diaryl-1H-pyrazol-4-yl)methylene)-2-Thioxoimidazolidin-4-one **3a–p**

A mixture of pyrazole carboxaldehydes **2a–p** (0.01 mol), 2-thiohydantoin (0.011 mol) and sodium acetate (0.03 mol) in acetic acid (4 mL) was refluxed for 24 h. After cooling, the reaction mixture was poured into ice water and the precipitate formed was filtered, washed with water, dried, and crystalized from ethanol. The supporting information are shown in Supplementary Materials.

5-((3-(4-Fluorophenyl)-1-phenyl-1*H*-pyrazol-4-yl)methylene)-2-thioxoimidazolidin-4-one **3a**

Yellow crystals, 2.73 g (79%) yield; m.p. 186–188 °C. IR (KBr): ῡ 3441 (N-H), 3399 (NH), 3067 (S_P_^2^ = C-H), 1726 (C=O), 1650 (C=S) and 1599 (C=N) cm^−1^. ^1^H-NMR (400 MHz, DMSO): δ 12.17 (s, 2H, 2NH), 9.32 (s, 1H, Pyrazol-H), 7.97 (d, *J* = 7.9 Hz, 2H, Ph-H_2_^/^ and H_6_^/^), 7.70 (dd, *J* = 8.3, 5.6 Hz, 2H, Ph-H_2_ and H_6_), 7.59 (t, *J* = 7.8 Hz, 2H, Ph-H_3_ and H_5_), 7.41 (t, *J* = 8.6 Hz, 3H, Ph-H_3_, H_4_ and H_5_) and 6.32 (s, 1H, CH=C) ppm. ^13^C-NMR (101 MHz, DMSO): δ 178.69 (C=S), 165.88 (C=O), 162.93 (d, ^1^*J*c_,F_ = 246.2 Hz, CF), 152.69 (C), 139.41 (C), 131.17 (d, ^2^*J*c_,F_ = 8.3 Hz, CH^/^), 130.10 (CH), 129.27 (CH), 128.54 (C), 127.60 (CH), 127.32 (C), 119.17 (CH), 116.39 (d, ^2^*J*c_,F_ = 21.7 Hz, CH^/^), 114.04 (C) and 101.89 (CH) ppm. C_19_H_13_FN_4_OS requires C, 62.63; H, 3.60; N, 15.38%. Found: C, 62.71; H, 3.54; N, 15.42.

5-((3-(4-Chlorophenyl)-1-phenyl-1*H*-pyrazol-4-yl)methylene)-2-thioxoimidazolidin-4-one **3b**

Yellow crystals, 2.35 g (62%) yield; m.p. 183–184 °C. IR (KBr): ῡ 3462 (N-H), 3401 (NH), 3058 (S_P_^2^ = C-H), 1723 (C=O), 1645 (C=S) and 1598 (C=N) cm^−1^. ^1^H-NMR (400 MHz, DMSO): δ 12.43 (s, 1H (66%), NH), 12.33 (s, 1H (33%), NH), 12.08 (s, 1H (66%), NH), 11.92 (s, 1H (33%), NH), 9.47 (s, 1H (33%), Pyrazol-H), 9.33 (s, 1H (66%), Pyrazol-H), 7.98 (d, *J* = 7.7 Hz, 1H, Ar-H), 7.86 (d, *J* = 7.7 Hz, 1H, Ar-H), 7.69 (dd, *J* = 8.7, 2.7 Hz, 2H, Ar-H), 7.66–7.61 (m, 2H, Ar-H), 7.59 (d, *J* = 8.3 Hz, 2H, Ar-H), 7.43 (t, *J* = 7.9 Hz, 1H, Ar-H), 6.55 (s, 1H (33%), CH=C) and 6.33 (s, 1H (66%), CH=C) ppm. ^13^C-NMR (101 MHz, DMSO): δ 178.69 (C=S), 175.27 (C=S), 165.82 (C=O), 164.32 (C=O), 153.09 (C), 152.37 (C), 139.36 (C), 134.13 (C), 131.03 (CH), 130.97 (C), 130.94 (C), 130.86 (CH), 130.72 (CH), 130.34 (CH), 130.24 (CH), 130.14 (CH), 129.50 (CH), 129.37 (CH), 129.07 (CH), 127.70 (CH), 127.36 (C), 119.44 (CH), 119.23 (CH), 114.54 (C), 114.08 (C), 107.67 (CH) and 101.76 (CH) ppm. C_19_H_13_ClN_4_OS requires C, 59.92; H, 3.44; N, 14.71%. Found: C, 60.01; H, 3.39; N, 14.76.

5-((3-(4-Bromophenyl)-1-phenyl-1*H*-pyrazol-4-yl)methylene)-2-thioxoimidazolidin-4-one **3c**

Yellow crystals, 3.44 g (81%) yield; m.p. 193–195 °C. IR (KBr): ῡ 3462 (N-H), 3404 (NH), 3071 (S_P_^2^ = C-H), 1723 (C=O), 1644 (C=S) and 1596 (C=N) cm^−1^. ^1^H-NMR (400 MHz, DMSO): δ 12.29 (s, 1H, NH), 12.03 (s, 1H, NH), 9.32 (s, 1H, Pyrazol-H), 7.97 (d, *J* = 7.9 Hz, 2H, Ar-H), 7.77 (d, *J* = 7.8 Hz, 2H, Ar-H), 7.60 (m, 4H, Ar-H), 7.42 (t, *J* = 7.2 Hz, 1H, Ar-H) and 6.33 (s, 1H, CH=C) ppm. ^13^C-NMR (101 MHz, DMSO): δ 178.71 (C=S), 165.82 (C=O), 152.35 (C), 139.38 (C), 132.41 (CH), 131.31 (C), 130.97 (CH), 130.13 (CH), 129.49 (CH), 127.69 (CH), 127.41 (C), 122.81 (C), 119.23 (CH), 114.06 (C) and 101.72 (CH) ppm. C_19_H_13_BrN_4_OS requires C, 53.66; H, 3.08; N, 13.17%. Found: C, 53.57; H, 3.19; N, 13.26.

5-((1-Phenyl-3-(4-(trifluoromethyl)phenyl)-1*H*-pyrazol-4-yl)methylene)-2-thioxoimidazolidin-4-one **3d**

Yellow crystals, 3.10 g (75%) yield; m.p. 183–185 °C. IR (KBr): ῡ 3422 (N-H), 3183 (S_P_^2^ = C-H), 1727 (C=O), 1651 (C=S) and 1543 (C=N) cm^−1^. ^1^H-NMR (400 MHz, DMSO): δ 12.23 (s, 2H, NH), 9.34 (s, 1H, Pyrazol-H), 7.98 (d, *J* = 8.0 Hz, 2H, Ar-H), 7.91 (dd, *J* = 17.0, 8.2 Hz, 4H, Ar-H), 7.60 (t, *J* = 7.8 Hz, 2H, Ar-H), 7.43 (t, *J* = 7.3 Hz, 1H, Ar-H) and 6.34 (s, 1H, CH=C) ppm. ^13^C-NMR (101 MHz, DMSO): δ 178.85 (C=S), 165.87 (C=O), 151.92 (C), 139.34 (C), 136.18 (C), 130.34 (C), 130.13 (CH), 130.01 (CH), 129.66 (CH) 129.47(q, ^2^*J*c_,F_ = 32.3 Hz, C), 127.79 (CH), 127.75 (C), 126.32 (q, ^3^*J*c_,F_ = 3.7 Hz, CH), 124.66 (q, ^1^*J*c_,F_ = 273.2 Hz, CF_3_), 119.29 (CH), 114.34 (C) and 101.35 (CH) ppm. C_20_H_13_F_3_N_4_OS requires C, 57.97; H, 3.16; N, 13.52%. Found: C, 58.05; H, 3.24; N, 13.43.

5-((1,3-Diphenyl-1H-pyrazol-4-yl)methylene)-2-thioxoimidazolidin-4-one **3e**

Yellow crystals, 2.66 g (77%) yield; m.p. 168–179 °C. IR (KBr): ῡ 3454 (N-H), 3058 (S_P_^2^ = C-H), 1722 (C=O), 1642 (C=S) and 1597 (C=N) cm^−1^. ^1^H-NMR (400 MHz, DMSO): δ 12.40 (s, 1H, NH), 12.05 (s, 1H, NH), 9.32 (s, 1H, Pyrazol-H), 7.97 (d, *J* = 7.8 Hz, 2H, Ar-H), 7.64 (d, *J* = 6.9 Hz, 2H, Ar-H), 7.61–7.46 (m, 5H, Ar-H), 7.40 (t, *J* = 7.4 Hz, 1H, Ar-H) and 6.37 (s, 1H, CH=N) ppm. ^13^C-NMR (101 MHz, DMSO): δ 178.59 (C=S), 165.85 (C=O), 153.67 (C), 139.42 (C), 131.94 (C), 130.05 (CH), 129.35 (CH), 129.26 (CH), 129.16 (CH), 129.06 (CH), 127.51 (CH), 127.03, 119.11 (CH), 114.03 (C) and 102.01 (CH) ppm. C_19_H_14_N_4_OS requires C, 65.88; H, 4.07; N, 16.17%. Found: C, 65.94; H, 3.92; N, 16.25.

5-((1-Phenyl-3-(*p*-tolyl)-1*H*-pyrazol-4-yl)methylene)-2-thioxoimidazolidin-4-one **3f**

Yellow crystals, 2.62 g (73%) yield; m.p. 189–191 °C. IR (KBr): ῡ 3454 (N-H), 3049 (S_P_^2^ =C-H), 2918 (S_P_^3^ C-H), 1724 (C=O), 1643 (C=S) and 1596 (C=N) cm^−1^. ^1^H-NMR (400 MHz, DMSO): δ 12.40 (s, 1H, NH), 12.05 (s, 1H, NH), 9.33 (s, 1H, Pyrazol-H), 7.97 (d, *J* = 7.8 Hz, 2H, Ar-H), 7.89–7.80 (m, 1H, Ar-H), 7.60 (t, *J* = 7.8 Hz, 2H, Ar-H), 7.54 (d, *J* = 8.0 Hz, 2H, Ar-H), 7.38 (d, *J* = 8.0 Hz, 2H, Ar-H), 6.36 (s, 1H, CH=C) and 2.40 (s, 3H, CH_3_) ppm. ^13^C-NMR (101 MHz, DMSO): δ 178.56 (C=S), 165.86 (C=O), 153.79 (C), 139.46 (C), 138.85 (C), 130.11 (CH), 129.96 (CH), 129.62 (CH), 129.17 (C), 128.95 (CH), 127.53 (CH), 126.95 (C), 119.69 (CH), 119.13 (CH), 113.99 (C) and 21.37 (CH_3_) ppm. C_20_H_16_N_4_OS requires C, 66.65; H, 4.47; N, 15.54%. Found: C, 66.69; H, 4.53; N, 15.64.

5-((3-(4-Aminophenyl)-1-phenyl-1*H*-pyrazol-4-yl)methylene)-2-thioxoimidazolidin-4-one **3g**

Yellow crystals, 2.34 g (65%) yield; m.p. 195–197 °C. IR (KBr): ῡ 3309 (N-H), 3129 (NH_2_), 3069 (S_P_^2^ = C-H), 1715 (C=O), 1660 (C=S) and 1593 (C=N) cm^−1^. ^1^H-NMR (400 MHz, DMSO): δ 12.35 (s, 1H, NH), 11.97 (s, 1H, NH), 9.83 (s, 1H, NH_2_), 9.24 (s, 1H, Pyrazol-H), 7.92 (d, *J* = 8.1 Hz, 2H, Ar-H), 7.53 (t, *J* = 7.8 Hz, 2H, Ar-H), 7.46 (d, *J* = 8.4 Hz, 2H, Ar-H), 7.34 (t, *J* = 7.4 Hz, 1H, Ar-H), 6.95 (d, *J* = 8.5 Hz, 2H, Ar-H) and 6.36 (s, 1H, CH=C) ppm. ^13^C-NMR (101 MHz, DMSO): δ 178.40 (C=S), 165.85 (C=O), 158.56 (C), 153.97 (C), 139.47 (C), 130.41 (CH), 129.91 (CH), 128.83 (CH), 127.20 (CH), 126.65 (C), 122.78 (C), 118.92 (CH), 116.13 (CH), 113.76 (C) and 102.79 (CH) ppm. C_19_H_15_N_5_OS requires C, 63.14; H, 4.18; N, 19.38%. Found: C, 63.05; H, 4.29; N, 19.43.

5-((1,3-Bis(4-fluorophenyl)-1H-pyrazol-4-yl)methylene)-2-thioxoimidazolidin-4-one **3h**

Yellow crystals, 3.24 g (82%) yield; m.p. 178–179 °C. IR (KBr): ῡ 3420 (N-H), 3066 (S_P_^2^ = C-H), 1724 (C=O), 1649 (C=S) and 1545 (C=N) cm^−1^. ^1^H-NMR (400 MHz, DMSO): δ 12.41 (s, 1H, NH), 12.03 (s, 1H, NH), 9.29 (s, 1H, Pyrazol-H), 8.00 (dd, *J* = 9.1, 4.7 Hz, 2H, Ar-H), 7.71 (dd, *J* = 8.6, 5.5 Hz, 2H, Ar-H), 7.47 (t, *J* = 8.8 Hz, 2H, Ar-H), 7.42 (t, *J* = 8.9 Hz, 2H, Ar-H) and 6.32 (s, 1H, CH=C) ppm. ^13^C-NMR (101 MHz, DMSO): δ 178.67 (C=S), 165.81 (C=O), 163.33 (d, ^1^*J*c_,F_ = 171.5 Hz, CF), 160.89 (d, ^1^*J*c_,F_ = 169.1 Hz, CF), 152.70 (C), 136.02 (C), 131.16 (d, ^3^*J*c_,F_ = 8.5 Hz, CH^/^), 129.52 (CH), 128.49 (C), 127.33 (C), 121.29 (d, ^3^*J*c_,F_ = 8.6 Hz, CH), 116.95 (d, ^2^*J*c_,F_ = 23.2 Hz, CH^/^), 116.43 (d, ^2^*J*c_,F_ = 21.8 Hz, CH), 114.04 (C) and 101.85 (CH) ppm. C_19_H_12_F_2_N_4_OS requires C, 59.68; H, 3.16; N, 14.65%. Found: C, 59.80; H, 3.22; N, 14.55. 

5-((3-(4-Chlorophenyl)-1-(4-fluorophenyl)-1H-pyrazol-4-yl)methylene)-2-thioxoimidazolidin-4-one **3i**

Yellow crystals, 2.42 g (61%) yield; m.p. 168–170 °C. IR (KBr): ῡ 3421 (N-H), 3140 (S_P_^2^ = C-H), 1731 (C=O), 1651 (C=S) and 1614 (C=N) cm^−1^. ^1^H-NMR (400 MHz, DMSO): δ 9.42 (s, 1H (50%), Pyrazol-H), 9.26 (s, 1H(50%), Pyrazol-H), 7.99 (dd, *J* = 7.5, 4.8 Hz, 1H, Ar-H), 7.89 (dd, *J* = 7.4, 4.8 Hz, 1H, Ar-H), 7.73–7.65 (m, 2H, Ar-H), 7.65–7.60 (m, 2H, Ar-H), 7.44 (dt, *J* = 13.7, 8.2 Hz, 2H, Ar-H), 6.53 (s, 1H (50%), CH=C) and 6.29 (s, 1H (50%), CH=C) ppm. ^13^C-NMR (101 MHz, DMSO): δ 178.79 (C=S), 175.33 (C=S), 165.93 (C=S), 164.32 (C=S), 161.28 (d, ^1^*J*c_,F_ = 244.0 Hz, CF), 153.04 (C), 152.30 (C), 135.97 (C), 134.16 (C), 131.00 (CH), 130.88 (C), 130.66 (CH), 129.62 (CH), 129.48 (CH), 129.36 (CH), 129.12 (C), 127.68 (C), 121.63 (d, ^3^*J*c_,F_ = 8.6 Hz, CH), 121.30 (d, ^3^*J*c_,F_ = 8.5 Hz, CH), 117.13 (d, ^2^*J*c_,F_ = 16.0 Hz, CH), 116.89 (d, ^2^*J*c_,F_ = 16.1 Hz, CH), 114.55 (C), 114.16 (C), 107.56 (CH) and 101.56 (CH) ppm. C_19_H_12_ClFN_4_OS requires C, 57.22; H, 3.03; N, 14.05%. Found: C, 57.11; H, 3.12; N, 14.13.

5-((3-(4-Bromophenyl)-1-(4-fluorophenyl)-1H-pyrazol-4-yl)methylene)-2-thioxoimidazolidin-4-one **3j**

Yellow crystals, 3.76 g (85%) yield; m.p. 199–201 °C. IR (KBr): ῡ 3441 (N-H), 3146 (S_P_^2^ = C-H), 1726 (C=O), 1648 (C=S) and 1553 (C=N) cm^−1^. ^1^H-NMR (400 MHz, DMSO): δ 12.42 (s, 1H, NH), 12.03 (s, 1H, NH), 9.28 (s, 1H, Pyrazol-H), 8.00 (dd, *J* = 8.9, 4.7 Hz, 2H, Ph-H_2_ and H_6_), 7.78 (d, *J* = 8.3 Hz, 2H, Ph-H_2_^/^ and H_6_^/^), 7.62 (d, *J* = 8.4 Hz, 3H, Ph-H_3_^/^ and H_5_^/^), 7.47 (t, *J* = 8.7 Hz, 2H, Ph-H_3_ and H_5_) and 6.32 (s, 1H, CH=C) ppm. ^13^C-NMR (101 MHz, DMSO): δ 178.71 (C=S), 165.79 (C=O), 161.30 (d, ^1^*J*c_,F_ = 244.2 Hz, CF), 135.98 (C), 132.42 (C), 131.23 (C), 130.94 (CH), 129.69 (CH), 127.49 (C), 122.84 (C), 121.34 (d, ^3^*J*c_,F_ = 8.6 Hz, CH), 116.95 (d, ^2^*J*c_,F_ = 23.2 Hz, CH), 114.07 (C) and 101.65 (CH) ppm. C_19_H_12_BrFN_4_OS requires C, 51.48; H, 2.73; N, 12.64%. Found: C, 51.55; H, 2.72; N, 12.81.

5-((1-(4-Fluorophenyl)-3-(4-(trifluoromethyl)phenyl)-1*H*-pyrazol-4-yl)methylene)-2-thioxoimidazolidin-4-one **3k**

Yellow crystals, 3.36 g (78%) yield; m.p. 210–212 °C. IR (KBr): ῡ 3441 (N-H), 3146 (S_P_^2^ = C-H), 1726 (C=O), 1648 (C=S) and 1553 (C=N) cm^−1^. ^1^H-NMR (400 MHz, DMSO): δ 12.40 (s, 1H, NH), 12.09 (s, 1H, NH), 9.28 (s, 1H, Pyrazol-H), 8.00 (dd, *J* = 9.0, 4.7 Hz, 2H, Ph-H_2_ and H_6_), 7.91 (dd, *J* = 18.6, 8.3 Hz, 4H, Ar-H), 7.47 (t, *J* = 8.8 Hz, 2H, Ph-H_3_ and H_5_) and 6.34 (s, 1H, CH=C) ppm. ^13^C-NMR (101 MHz, DMSO): δ 178.81 (C=S), 165.78 (C=O), 161.37 (d, ^1^*J*c_,F_ = 244.3 Hz, CF), 151.90 (C), 136.09 (C), 135.93 (C), 129.84 (CH), 129.64 (CH), 129.49 (q, ^2^*J*c_,F_ = 32.2 Hz, C), 127.72 (C), 126.33 (q, ^3^*J*c_,F_ = 4.0 Hz, CH), 127.35 (q, ^1^*J*c_,F_ = 272.7 Hz, CF_3_), 121.40 (d, ^3^*J*c_,F_ = 8.6 Hz, CH), 116.96 (d, ^2^*J*c_,F_ = 23.2 Hz, CH), 114.33 (C) and 101.32 (CH) ppm. C_20_H_12_F_4_N_4_OS requires C, 55.55; H, 2.80; N, 12.96%. Found: C, 55,43; H, 2.89; N, 12.88.

5-((1-(4-Fluorophenyl)-3-phenyl-1*H*-pyrazol-4-yl)methylene)-2-thioxoimidazolidin-4-one **3l**

Yellow crystals, 2.47 g (68%) yield; m.p. 189–191 °C. IR (KBr): ῡ 3419 (N-H), 3213 (N-H), 3069 (S_P_^2^ = C-H), 1724 (C=O) and 1645 (C=S) cm^−1^. ^1^H-NMR (400 MHz, DMSO): δ 11.81 (s, 2H, 2NH), 9.28 (s, 1H, Pyrazol-H), 7.99 (dd, *J* = 8.6, 4.6 Hz, 2H, Ph-H_2_ and H_6_), 7.65 (d, *J* = 7.2 Hz, 2H, Ar-H), 7.57 (t, *J* = 7.2 Hz, 2H, Ar-H), 7.54–7.49 (m, 1H, Ar-H), 7.45 (t, *J* = 8.6 Hz, 2H, Ph-H_3_ and H_5_) and 6.32 (s, 1H, CH=C) ppm. ^13^C-NMR (101 MHz, DMSO): δ 178.85 (C=S), 166.16 (C=O), 161.22 (d, ^1^*J*c_,F_ = 244.1 Hz, CF), 153.63 (C), 136.07 (C), 132.03 (C), 129.38 (CH), 129.33 (CH), 129.30 (CH), 129.02 (CH), 127.74 (C), 121.22 (d, ^3^*J*c_,F_ = 8.5 Hz, CH), 116.91 (d, ^2^*J*c_,F_ = 23.1 Hz, CH), 114.22 (C) and 101.85 (CH) ppm. C_19_H_13_FN_4_OS requires C, 62.63; H, 3.60; N, 15.38%. Found: C, 62.71; H, 3.72; N, 15.32.

5-((1-(4-Fluorophenyl)-3-(*p*-tolyl)-1*H*-pyrazol-4-yl)methylene)-2-thioxoimidazolidin-4-one **3m**

Yellow crystals, 2.68 g (71%) yield; m.p. 167–169 °C. IR (KBr): ῡ 3435 (N-H), 3068 (S_P_^2^ = C-H), 2920 (S_P_^3^ C-H), 1723 (C=O), 1644 (C=S) and 1539 (C=N) cm^−1^. ^1^H-NMR (400 MHz, DMSO): δ 12.40 (s, 1H, NH), 11.99 (s, 1H, NH), 9.26 (s, 1H, Pyrazol-H), 7.98 (dd, *J* = 9.1, 4.7 Hz, 2H, Ph-H_2_ and H_6_), 7.53 (d, *J* = 8.0 Hz, 2H, Ph-H_3_^/^ and H_5_^/^), 7.45 (t, *J* = 8.8 Hz, 2H, Ph-H_3_ and H_5_), 7.37 (d, *J* = 7.9 Hz, 2H, Ph-H_2_^/^ and H_6_^/^), 6.35 (s, 1H, CH=C), 2.40 (s, 3H, CH_3_). ^13^C-NMR (101 MHz, DMSO): δ 178.57 (C=S), 165.82 (C=O), 161.19 (d, ^1^*J*c_,F_ = 244.0 Hz, CF), 153.74 (C), 138.85 (C), 136.05 (C), 129.94 (CH), 129.32 (CH), 129.11 (C), 128.91 (CH), 127.03 (C), 121.18 (d, ^3^*J*c_,F_ = 8.5 Hz, CH), 116.88 (d, ^2^*J*c_,F_ = 23.1 Hz, CH), 114.00 (C), 102.27 (CH) and 21.44 (CH_3_) ppm. C_20_H_15_FN_4_OS requires C, 63.48; H, 4.00; N, 14.81%. Found: C, 63.56; H, 3.92; N, 14.86.

5-((1-(4-Fluorophenyl)-3-(4-methoxyphenyl)-1*H*-pyrazol-4-yl)methylene)-2-thioxoimidazolidin-4-one **3n**

Yellow crystals, 3.03 g (77%) yield; m.p. 179–181 °C. IR (KBr): ῡ 3445 (N-H), 3146 (S_P_^2^ = C-H), 2963 (S_P_^3^ C-H), 1719 (C=O), 1641 (C=S) and 1581 (C=N) cm^−1^. ^1^H-NMR (400 MHz, DMSO): δ 12.37 (s, 1H, NH), 12.01 (s, 1H, NH), 9.25 (s, 1H, Pyrazol-H), 7.97 (dd, *J* = 9.0, 4.7 Hz, 2H, Ph-H_2_ and H_6_), 7.57 (d, *J* = 8.6 Hz, 2H, Ph-H_3_^/^ and H_5_^/^), 7.44 (t, *J* = 8.7 Hz, 2H, Ph-H_3_ and H_5_), 7.12 (d, *J* = 8.7 Hz, 2H, Ph-H_2_^/^ and H_6_^/^), 6.35 (s, 1H, CH=C) and 3.84 (s, 3H, OCH_3_) ppm. ^13^C-NMR (101 MHz, DMSO): δ 178.52 (C=S), 165.82 (C=O), 161.15 (d, ^1^*J*c_,F_ = 243.8 Hz, CF), 160.24 (C), 153.58 (C), 136.08 (C), 130.33 (CH), 126.96 (C), 124.27 (C), 121.12 (d, ^1^*J*c_,F_ = 8.5 Hz, CH), 116.87 (d, ^2^*J*c_,F_ = 23.1 Hz, CH), 114.84 (CH), 113.88 (C), 102.41 (CH) and 55.74 (OCH_3_) ppm. C_20_H_15_FN_4_O_2_S requires C, 60.90; H, 3.83; N, 14.20%. Found: C, 60.82; H, 3.92; N, 14.38.

5-((3-(4-Fluorophenyl)-1-(4-(trifluoromethyl)phenyl)-1*H*-pyrazol-4-yl)methylene)-2-thioxoimidazolidin-4-one **3o**

Yellow crystals, 3.62 g (84%) yield; m.p. 205–207 °C. IR (KBr): ῡ 3416 (N-H), 3072 (S_P_^2^ = C-H), 1721 (C=O), 1644 (C=S) and 1621 (C=N) cm^−1^. ^1^H-NMR (400 MHz, DMSO): δ 12.11 (s, 2H, 2NH), 9.33 (s, 1H, Pyrazol-H), 7.78 (m, 8H, Ar-H) and 6.27 (s, 1H, CH=C) ppm. ^13^C-NMR (101 MHz, DMSO): δ 178.86 (C=S), 165.72 (C=O), 163.03 (d, ^1^*J*c_,F_ = 246.6 Hz, CF), 153.20 (C), 142.08 (C), 131.10 (d, ^3^*J*c_,F_ = 8.5 Hz, CH), 129.59 (CH), 128.05 (q, ^2^*J*c_,F_ = 34.3 Hz, C), 127.34 (q, ^3^*J*c_,F_ = 3.6 Hz, CH), 124.52 (q, ^1^*J*c_,F_ = 273.0 Hz, CF_3_), 119.29 (CH), 116.35 (d, ^3^*J*c_,F_ = 21.7 Hz, CH), 114.72 (C), and 101.20 (CH) ppm. C_20_H_12_F_4_N_4_OS requires C, 55.55; H, 2.80; N, 12.96%. Found: C, 55.48; H, 2.92; N, 13.03.

5-((1,3-Bis(4-(trifluoromethyl)phenyl)-1*H*-pyrazol-4-yl)methylene)-2-thioxoimidazolidin-4-one **3p**

Yellow crystals, 3.85 g (80%) yield; m.p. 223–225 °C. IR (KBr): ῡ 3446 (N-H), 3081 (S_P_^2^ = C-H), 1731 (C=O), 1661 (C=S) and 1617 (C=N) cm^−1^. ^1^H-NMR (400 MHz, DMSO): δ 11.46 (s, 2H, 2NH), 9.32 (s, 1H, Pyrazol-H), 8.12 (d, *J* = 8.1 Hz, 2H, Ar-H), 7.92 (d, *J* = 8.3 Hz, 2H, Ar-H), 7.86 (dd, *J* = 15.0, 8.0 Hz, 4H, Ar-H) and 6.26 (s, 1H, CH=C) ppm. ^13^C-NMR (101 MHz, DMSO): δ 179.03 (C=S), 165.84 (C=O), 152.38 (C), 141.93 (C), 135.75 (C), 129.56 (CH), 128.22 (C), 129.61(q, ^2^*J*c_,F_ = 32.0 Hz, C), 127.60 (q, ^2^*J*c_,F_ = 32.3 Hz, C), 127.34 (q, ^3^*J*c_,F_ = 3.6 Hz, CH), 126.22 (q, ^3^*J*c_,F_ = 3.5 Hz, CH), 124.57 (q, ^1^*J*c_,F_ = 273.2 Hz, CF_3_), 124.47 (q, ^1^*J*c_,F_ = 273.7 Hz, CF_3_), 119.32 (CH), 115.00 (C) and 100.54 (CH) ppm. C_21_H_12_F_6_N_4_OS requires C, 52.29; H, 2.51; N, 11.61%. Found: C, 52.37; H, 2.44; N, 11.70.

### 2.4. Biological Assays

#### 2.4.1. In Vitro Cytotoxicity Assay (CPE Assay)

The antitumor properties of pyrazolylmethylene-2-thioxoimidazolidin-4-one derivatives (**3a–p**) against androgen-sensitive (AR^+^LNCaP; ATCC CRL-1740, Accession: CVCL_0395) and androgen-insensitive (AR^-^PC-3; ATCC CRL-1435, Accession: CVCL_D5ZS) human prostate cancer cell lines in addition to normal human lung fibroblast cell line (Wi38, Accession: CVCL_2759) were evaluated by MTT assay. AR^+^LNCaP, AR^-^PC-3, and Wi38 cell lines were purchased frozen in liquid nitrogen from Egyptian Company for Production of Vaccines, Sera and Drugs, Cairo, Egypt (VACSERA). Tumor cell lines were stored as monolayer cultures in DMEM supplemented with 10% FBS and 1% penicillin–streptomycin. MTT assay was carried out as described previously in the literature [33,34]. In brief, AR^+^LNCaP, AR^-^PC-3, and Wi38 cells were cultured into 96-well plates containing 100 µL of culture medium per each well. Cells were incubated with dilution series compounds (**3a–p**) to give a final concentration of 10, 25, 50, 75, 100, 200, 300, 500, 750, and 1000 ug/mL. A control containing only culture medium was also applied. The assay was performed in triplicate. The cells were incubated overnight to grow at 37 °C in 5% CO_2_. After removing the growth medium, 100 µL of MTT (0.4 mg/mL of PBS) were added. After 24 h of incubation, the supernatant was removed and 100 µL of DMSO (Sigma Aldrich, Germany) were then added to each sample. Absorbance at 550 nm was measured. The formula (Reading of extract/Reading of negative control) × 100 was applied to calculate the viability percentage. The IC_50_ value of test compounds (**3a–p**) was also determined using CPE measurements of normal Wi38 cells (normal human lung fibroblast cells). The MTT assay was repeated with the test compounds (**3i**, **3k**, **3o**, **3j**, **3p**, and **3m**) using the three cell types which were stimulated by 1 nM DHT after 48 h of incubation [35]. IC_50_ values were estimated using Graph Pad Prism computer software (International Scientific Community, San Diego, CA, USA) from linear regression analysis of the concentration–response curves plotted for each tested compound [36].

#### 2.4.2. Detection of Caspase-3 in AR^+^LNCaP

Detection of Caspase-3 in AR^+^LNCaP cells stimulated with 1 nM DHT and treated with IC_50_ concentrations of test compounds **3i–k**, **3m** and **3o–p** was determined as described previously [37]. Untreated AR^+^LNCaP and Enzalutamide-treated AR^+^LNCaP were used as negative and positive controls, respectively. AR^+^LNCaP cells were cultured in 96-well plates at a cell density of 1 × 10^4^ cells/well. Cells were then lysed with 50 µL lysis buffer/well supplemented with the Invitrogen Elisa kit for detection of active human caspase-3 (Invitrogen, Camarillo, CA, USA). Plates were frozen at −80 °C for 30 min and then thawed. The lysate was mixed with 2 Z-DEVD-R110 substrate and incubated at room temperature for 30 min. A fluorescence microplate reader (Tecan, Mainz-Kastel, Germany), was used to measure fluorescence via excitation at 496 nm and emission detection at 520 nm. Values were normalized with total protein content. Active caspase 3 accumulation was determined based on standard dilution series of Human Caspase-3 samples provided with the kit.

#### 2.4.3. Evaluation of DNA Content in AR^+^LNCaP Cells

The amount of DNA contents of AR^+^LNCaP cells stimulated with 1 nM DHT and treated with representative test compounds **3i** and **3k** at the different stages of the cell cycle were quantitatively evaluated as described previously [38]. Untreated AR^+^LNCaP and Enzalutamide-treated AR^+^LNCaP were used as negative and positive controls, respectively. The Ab139418–Propidium Iodide Flow Cytometry Kit for Cell Cycle Analysis (ab139418, Abcam, Cambridge, UK) was employed for this assay following the procedures provided by the supplier.

#### 2.4.4. Cell Apoptosis

Cell apoptosis assay was performed to ensure the efficacy of compounds **3i** and **3k** against the progression of AR^+^LNCaP tumor cell growth in the presence of 1 nM DHT. Untreated AR^+^LNCaP and Enzalutamide-treated AR^+^LNCaP were used in this assay as negative and positive controls, respectively. AR^+^LNCaP cells were precultured in 25 cm^2^ flasks. Briefly, 1 nM DHT was then added to the growing cells. IC_50_ concentrations of **3i** and **3k** in DMEM-media and RPMI-1640 (Sigma-Aldrich, Germany) were applied and incubated for 24 h. AR^+^LNCaP cells were harvested and fixed with 70% (*v*/*v*) ethanol in FBS (Sigma-Aldrich, Germany), then maintained at 4 °C overnight. The cells were resuspended in FBS containing 0.1% (*v*/*v*) Triton X-100, 40 µg/mL penicillin, and 0.1 mg/mL RNase then incubated in a dark chamber at 37 °C for 30 min. A flow-cytometer (Becton Dickinson, San Jose, CA, USA) supplemented with an argon ion laser at a wavelength of 488 nm was used to analyze the AR^+^LNCaP cell apoptotic stages. Based on the protocol described by Ozgur et al. (2003) [39], analysis of the data was implemented using Multicycle Software (Phoenix Flow Systems, San Diego, CA, USA; http://www.phoenixflow.com/index.html). 

### 2.5. Statistical Analysis

Data are presented as mean ± standard error of the mean. Significance was determined according to Student‘s *t*-test using Excel software (Microsoft Corporation, Redmond, DC, USA; https://www.microsoft.com/en-us/microsoft-365/excel) and one-way ANOVA test. Two-sided tests were performed for homoscedastic matrices.

## 3. Results and Discussion

### 3.1. Chemistry

**Scheme 1:**
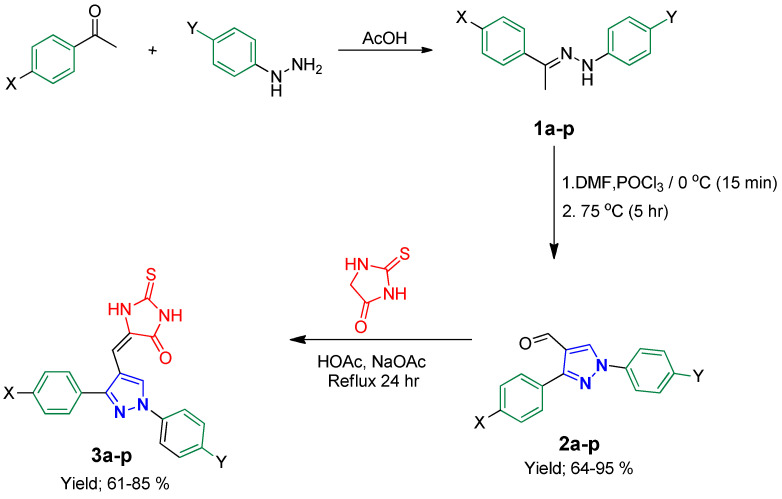
Synthesis of derivatives of pyrazolylmethylene-2-thioxoimidazolidin-4-one **3a–p.**

Derivatives of pyrazolylmethylene-2-thioxoimidazolidin-4-one **3a–p** were synthesized, in moderate to good yields, using Knoevenagel condensation reaction between aldehydic group of pyrazole carboxaldehydes **2a–p** and C-5 of 2-thioxoimidazolidin-4-one in acetic acid and in presence of sodium acetate. The starting pyrazole carboxaldehydes **2a–p** were initially prepared by Vilsmeier–Haack formylation of their corresponding acetophenone phenylhydrazone derivatives, as illustrated in Scheme 1.


**Entry**

**Substrates**

**(2a–p)**

**Products**

**(3a–p)**

**m.p. ^a^**

**(°C)**

**Yield ^b^**

**%**

**1**

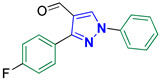
 **2a**
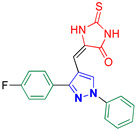
 **3a**186–188 79
**2**

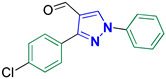
 **2b**
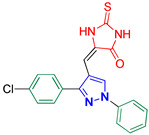
 **3b**183–18462
**3**

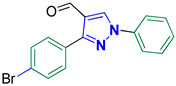
 **2c**
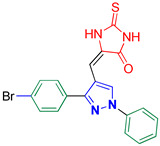
 **3c**193–19581
**4**

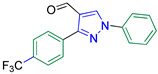
 **2d**
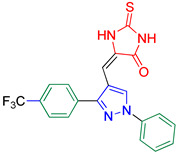
 **3d**183–18575
**5**

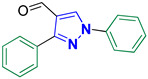
 **2e**
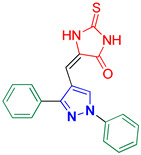
 **3e**168–17977
**6**

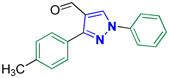
 **2f**
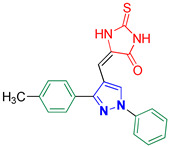
 **3f**189–19173
**7**

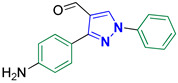
 **2g**
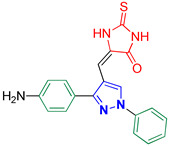
 **3g**195–19765
**8**

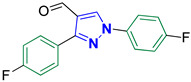
 **2h**
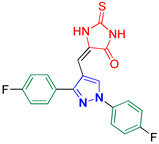
 **3h**178–17982
**9**

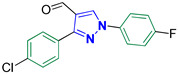
 **2i**
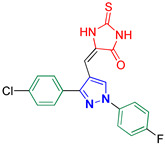
 **3i**168–17061
**10**

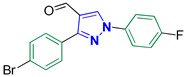
 **2j**
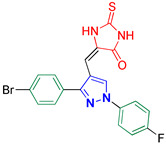
 **3j**199–20185
**11**

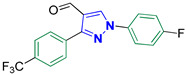
 **2k**
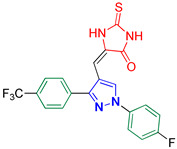
 **3k**210–21278
**12**

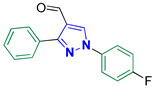
 **2l**
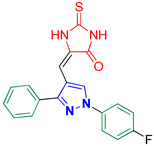
 **3l**189–19168
**13**

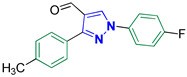
 **2m**
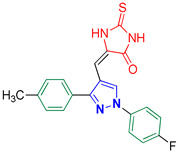
 **3m**167–16971
**14**

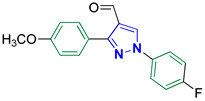
 **2n**
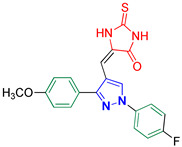
 **3n**179–18177
**15**

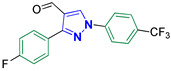
 **2o**
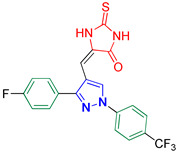
 **3o**205–20784
**16**

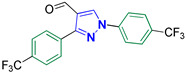
 **2p**
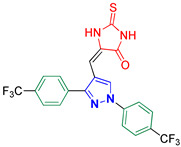
 **3p**223–22580^a^ Melting point of products **3a–p**; ^b^ isolated yield of products **3a–p**.

The molecular structure of the Knoevenagel adducts **3a–p** were characterized by IR, ^1^H-NMR, ^13^C-NMR, and elemental analysis. Infrared spectra of adducts **3a–p** revealed an absorption peak for N-H stretching between 3300 and 3465 cm^−1^. Moreover, adducts **3a–p** showed two sharp absorption peaks in the regions 1640–1660 cm^−1^ and 1720–1730 cm^−1^ that were ascribed to the stretching of the thiocarbonyl group and lactam carbonyl group, respectively. The ^1^H-NMR spectra of adducts **3a–p** showed a singlet signal near ≈6.30 ppm that is attributed to the proton of the methine bridge. This proves the success of the kneovengal condensation process. Moreover, the olefinic linker group has (*E)* configuration [40]. Only two compounds, **3b** and **3i**, were prepared as *E* and *Z* isomers. The proton of the methine bridge of the *Z* isomer was relatively more deshielded (δ ≈ 6.55 ppm) due to the magnetic anisotropy effect of the nearby lactam carbonyl group [40]. Pyrazolyl proton revealed a highly deshielded singlet at ≈9.30 ppm, which validates the accomplishment of the Vilsmeier–Haack formylation of acetophenone phenylhydrazone derivatives. Exchangeable protons of imidazolidine NH groups displayed downfield broad signals at ≈12.40 and 12.00 ppm that were identified based on their D_2_O exchange. Furthermore, protons of the benzene rings exhibited downfield signals at the region ≈7.10 to 8.10 ppm. Consequently, as a prototype, the protons of benzene rings of **3j** exhibited a doublet of doublet, a pair of doublet, and pseudo triplet (pst) with integral of two protons per each signal. The two doublets with total integral of four protons are attributed to the protons of the aryl bromide ring. The doublet of doublet peak is ascribed to the two protons in *meta* position with respect to the fluorine atom in the aryl fluoride ring. This splitting arose due to both homocoupling with the vicinal proton (^3^*J*_H,H_ = 8.9 Hz) and long-range heterocoupling with the fluorine atom (^4^*J*_H,F_ = 4.7 Hz). Moreover, the pseudo triplet (pst) peak originated from the two ortho protons with respect to the fluorine atom. One possible explanation for the pst signal being detected rather than a doublet of doublet is that three-bond coupling constants for both heterocoupling and homocoupling are almost the same (^3^*J*_H,F_ ≈ ^3^*J*_H,H_ ≈ 8.7 Hz).

**Figure 3:**
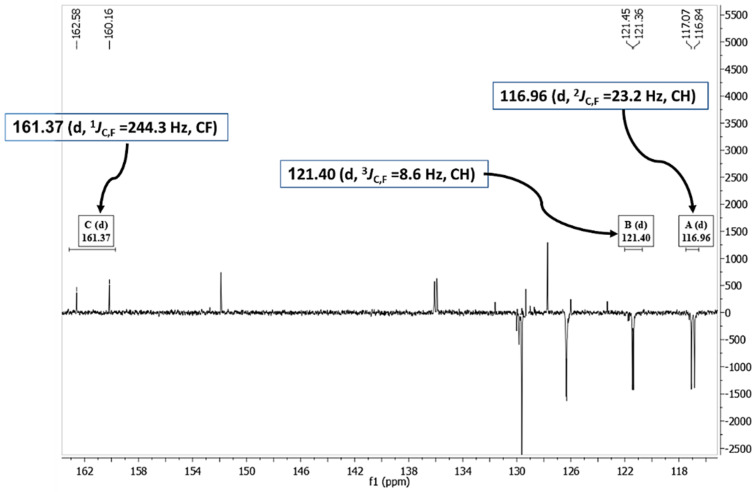

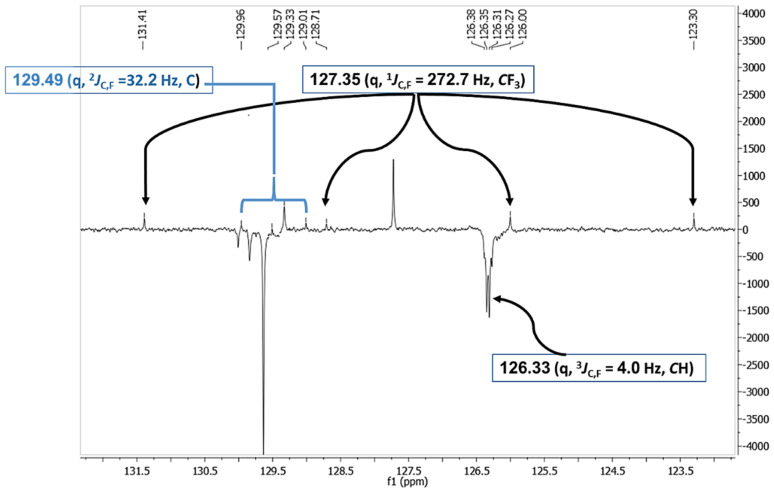
Carbon–fluorine splitting in ^13^C-NMR (APT) spectrum of compound **3k**.

The ^13^C-NMR spectra of the adducts **3a–p** displayed downfield signals at ≈δ 178.69 and 165.88 that correspond to the carbons of thiocarbonyl and carbonyl groups, respectively. Carbon of the methine bridge revealed a signal at ≈101.65 ppm. Furthermore, compounds **3f**, **3m**, and **3n** exhibited signals at 21.37, 21.44, and 55.74 ppm, respectively, that match the aliphatic methyl carbons of these compounds. The carbons of the aromatic rings exhibited downfield signals in the region ≈107–160 ppm. Compounds **3a**, **3d**, and **3h–p** containing either fluoro or trifluoromethyl groups showed splitting in their ^13^C-NMR spectra due to carbon–fluorine heterocoupling [41,42]. As a prototype, the ^13^C-NMR spectrum of **3k** (Figure 3) displayed three doublets at ≈161.37, 121.40, and 116.96 ppm, with heterocoupling constants *J*_C,F_ equal to 244.3, 8.6, and 23.2 Hz, respectively. These splittings were attributed to the heterocoupling that occurred in the aryl fluoride ring. The magnitude of these coupling constants relies on the bond distance between carbon and fluorine atoms, i.e., the quaternary aromatic carbon bearing the fluorine atom showed the highest coupling magnitude while the lowest coupling magnitude is ascribed to the carbon atom in *meta* position relative to the fluorine atom. Moreover, three quartets were also observed at 129.49 ppm (^2^*J*_C,F_ = 32.2 Hz), 127.35 ppm (^1^*J*_C,F_ = 272.7 Hz), and 126.33 ppm (^3^*J*_C,F_ = 4.0 Hz) due to carbon–fluorine coupling of the benzotriflouride moiety. 

### 3.2. Biological Assays

#### 3.2.1. In Vitro CYTOTOXICITY ASSAY

In vitro cytotoxicity assay was performed to elucidate the antitumor properties of the synthesized derivatives of pyrazolylmethylene-2-thioxoimidazolidin-4-one. Primarily, all the 16 newly chemically synthesized compounds were tested in addition to doxorubicin and enzalutamide, as reference drugs, at varying concentrations against the androgen-sensitive (AR^+^LNCaP) and androgen-insensitive (AR^-^PC-3) prostate cancer cells as well as the noncancerous human lung fibroblast cell line (Wi38) human lung cell line. The cytotoxicity assay was performed using MTT assay as described in the Materials and Methods Section 2.4.1. The test compounds were dissolved in DMSO as stock solutions (<0.5% (*v*/*v*)) and stored in the dark at −20 °C. The human prostate cancer cells and normal human lung fibroblast cells were subjected to variable doses of the compounds for 24 h. The IC_50_ value, which is defined as the concentration of test compound resulting in 50% inhibition of cell growth, was calculated by log-linear interpolation of the observed data points from each applied concentration of the test compounds. Variable inhibitory effects were observed for all the tested 16 compounds (**3a–p**) as shown in Table 1. However, results indicated potent cytotoxicity for samples **3i–k**, **3m**, and **3o–p** against the tested tumor cell line with superior selectivity towards androgen-sensitive prostate cancer AR^+^LNCaP cells (Table 1). The recorded IC_50_ values for compound **3i–k**, **3m**, and **3o–p** against AR^+^LNCaP are 12.07 ± 0.09 µM, 13.89 ± 0.14 µM, 10.27 ± 0.14 µM, 12.97 ± 0.06 µM, 12.66 ± 0.11 µM, and 12.11 ± 0.08 µM with selectivity index of 2.5, 2.2, 3.2, 2.4, 2.0, and 2.1 respectively.

The observed IC_50_ values for these compounds are comparable to the results recorded for the commercial drugs doxorubicin, and enzalutamide. The other test samples **3a–g, 3n**, and **3l** showed low to moderate activity or minor selectivity against AR^+^LNCaP. 

Based on the measured IC_50_ values and selectivity index of the newly synthesized derivatives (**3a–p**) against AR^+^ LNCap cells (Table 1), the synthesized target compounds analogues **3i–k**, **3m**, and **3o–p** were selected and further re-evaluated for their in vitro cytotoxicity against human prostate cancer cell lines (AR^+^LNCaP and AR^-^PC-3) and noncancerous cells (Wi38). The assay was performed in the presence of dihydrotestosterone (1 nM DHT) for 48 h of incubation as previously described [43,44]. DHT is a potent inducer of androgens that enhances prostate cancer disease progression [45]. The Enzalutamide reference drug, a representative compound of the second-generation AR antagonists [46,47], was used in this assay as a growth inhibitor of AR^+^LNCaP androgen-sensitive prostate cancer cells. Results of the second cytotoxicity assay using the selected compounds **3i–k**, **3m**, and **3o–p**, with the best performance against AR^+^LNCaP tumor cells, are shown in Table 2. IC_50_ value recorded for the reference drug enzalutamide is 06.52 ± 0.16 µM for AR^+^LNCaP and 13.58 ± 0.27 µM for AR^-^PC-3 with a selectivity index of 2.1 towards AR^+^LNCaP. All the test target compounds **3i–k**, **3m**, and **3o–p** show comparable IC_50_ values against AR^+^LNCaP.

The results indicate that compounds **3i–k** and **3p** showed cytotoxic potential with IC_50_ values of 5.22 to 7.72 μM against AR^+^LNCaP, 13.87 to 15.52 μM against AR^-^PC-3, and 19.81 to 21.93 μM against Wi38 cell lines. The IC_50_ values indicated that compounds **3i–k** and **3p** were more selective towards the AR^+^LNCaP cell line with selectivity index of 2.6, 2.7, 1.8, and 2.7, respectively. Compounds **3k** and **3p** showed the most potent inhibition of AR^+^LNCaP growth, with 1.2- and 1.1-fold, compared to the clinically used antiandrogens enzalutamide reference drug. The safety index (SI) is a measure of the drug candidate’s safety towards normal cells. SI can be calculated as the ratio between the tested compound IC_50_ on normal cells to IC_50_ on cancer cells. Accordingly, compound **3k** showed the highest safety index (3.8) followed by compounds **3i** (3.6) and **3p** (3.4), as shown in Table 2. Therefore, it can be concluded that compound **3k** showed the highest anticancer activity compared to all the test target compounds and it is especially safe and more selective against AR^+^LNCaP under the assay conditions.

#### 3.2.2. Evaluation of Active Caspase 3 Content from Lysate of AR^+^LNCaP Cells Treated with the Synthesized Compounds **3i–k**, **3m**, and **3o–p**

**Figure 4:**
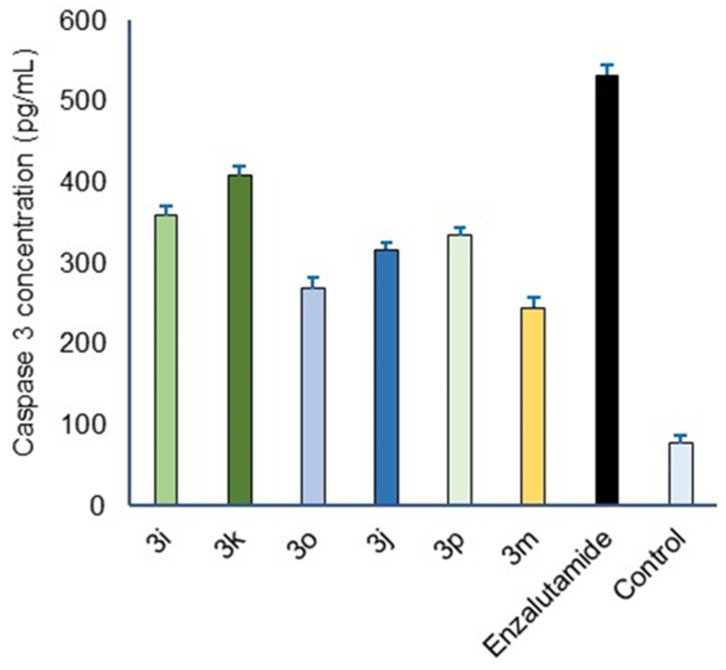
Effect of compounds **3i–k**, **3m**, and **3o–p** and reference drug Enzalutamide treatment on the accumulation of Caspase 3 in AR^+^LNCaP cells compared to control (untreated AR+LNCaP) measured by Invitrogen Elisa kit for detection of active human caspase 3. Data are presented as mean ± SE of at least three independent measurments. Statistical significance was determined compared to untreteated AR+LNCaP cells using one-way ANOVA test.

The most powerful approach to stop the growth of tumors is to induce apoptosis in cancer cells by therapeutic drugs. There are two main apoptotic pathways: the extrinsic pathway, which ultimately activates Caspase-3 and Caspase-8, and the intrinsic pathway, in which the endogenous signal activates Caspase-3 and Caspase-9 [48]. The majority of apoptotic signaling pathways activate caspase-3 [49]. Therefore, the main executioner protease in a cell that initiates the apoptotic process is caspase 3. Consequently, detection of active caspase 3 in cells and tissues is an important tool to confirm the initiation of cell apoptosis induced by a wide variety of apoptotic signals. For the study of cellular processes and various illnesses with various etiologies, sensitive and repeatable detection of activated caspase 3 is crucial [50,51,52]. Accordingly, active Caspase 3 accumulation was measured from lysates of AR^+^LNCaP cells treated with the IC_50_ concentrations of the synthesized compounds **3i–k**, **3m**, and **3o–p** and reference drug Enzalutamide in addition to untreated cell controls using Invitrogen Elisa kit for detection of active human caspase 3. The results of this assay are shown in Figure 4.

The reference drug Enzalutamide records about 6.6-fold of active Caspase-3 compared to untreated cell control. The active caspase 3 contents measured from AR^+^LNCaP cells lysates treated with the synthesized compounds **3i–k**, **3m**, and **3o–p** showed significant 4.7-fold, 4.2-fold, 5.4-fold, 3.2-fold, 3.5-fold, and 4.4-fold changes, respectively, compared to untreated cells (*** *p* < 0.001.). Compared to the reference drug Enzalutamide, induction of active caspase 3 in AR^+^LNCaP cells with the synthesized compounds **3i–k**, **3m**, and **3o–p** is 71.4%, 63.0%, 81.2%, 48.6%, 53.4%, and 66.6%, respectively, as a percentage of the reference drug. The observed results in this assay are correlated with the results observed with the cytotoxicity assays (Table 1 and Table 2). It can be therefore concluded that all the synthesized compounds are able to induce the accumulation of the apoptotic process initiator caspase 3 in treated AR^+^LNCaP cells, consequently causing cell death. 

#### 3.2.3. DNA Evaluation at Different Stages of Cell Cycle in AR^+^LNCaP Treated with Compounds **3i** and **3k** in Presence of DHT

**Figure 5:**
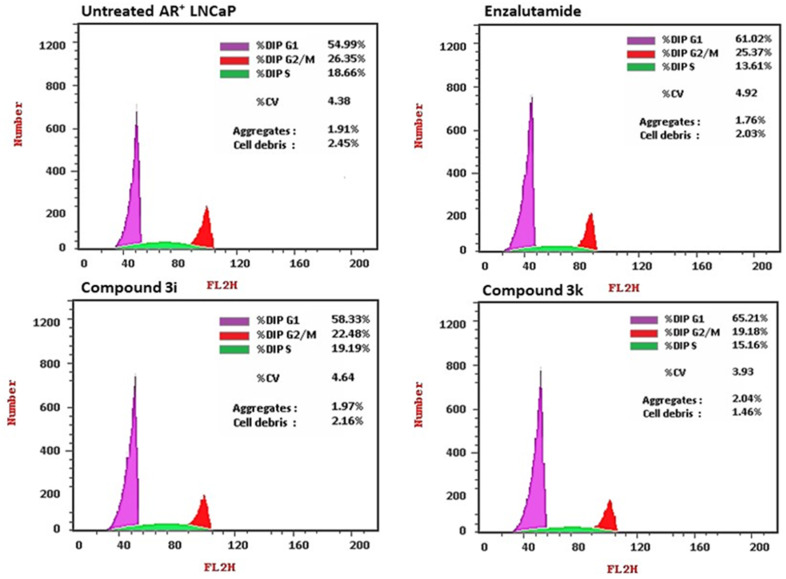
Evaluation of DNA contents of AR^+^LNCaP cells at the different stages of the cell cycle after treatment with the test compounds **3i** and **3k**.

The synthesized compounds **3i** and **3k**, that induced higher production of Caspase 3 in AR^+^LNCaP cells (Figure 4), were further analyzed. Both compounds were evaluated for their effect on the different stages of AR^+^LNCaP prostate cancer cells apoptosis in a direct comparison with untreated AR^+^LNCaP cells and Enzalutamide reference drug. The IC_50_ concentrations of the test compounds were applied in the presence of 1 nM DHT. Firstly, the ab139418-Propidium Iodide Flow Cytometry Kit for Cell Cycle Analysis (Bio vision, Boston, MA, USA) was used to quantify DNA contents at each cell cycle stage as an indicator of the cell cycle growth arrest [38]. The result of this assay is shown in Table 3 and Figure 5. 

The reference drug Enzalutamide causes AR^+^LNCaP growth arrest at G_1_ phase as DNA contents at G_0_-G_1_ records 61.02%. Similarly, the test compound **3k** initiates cell growth arrest at G_1_ phase with DNA content of 65.21% at G_0_- G_1_ phase. However, the test compound **3i** causes cell growth arrest at G_1_/S with DNA content of 58.33% at G_0_-G_1_ phase and 19.19% at S phase. Therefore, it can be concluded that both representative test compounds **3i** and **3k** can initiate AR^+^LNCaP cell growth arrest and stop the progression of tumor cell growth at the early stages of the cell cycle.

#### 3.2.4. AR^+^LNCaP Cell Apoptosis Analysis after Treatment with Compounds **3i** and **3k** in Presence of DHT

**Figure 6:**
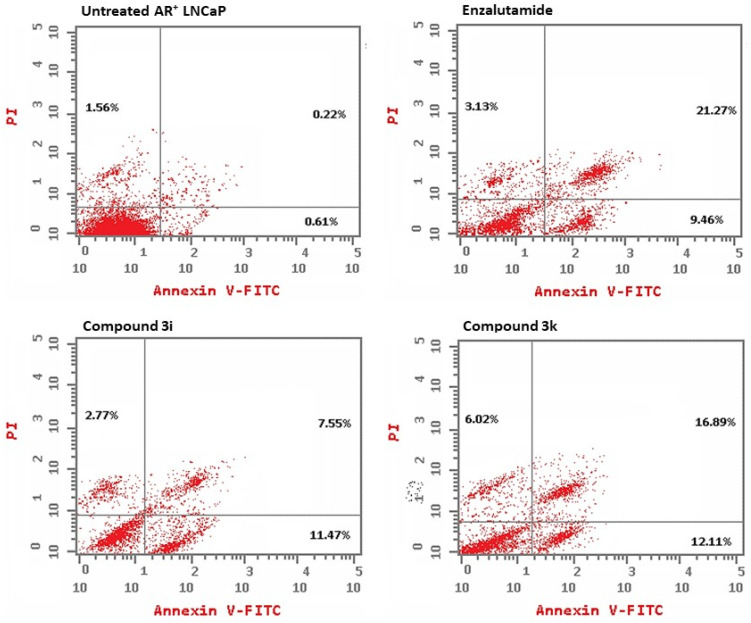
Effect of the test compounds **3i** and **3k** on AR^+^LNCaP cell apoptosis.

The active compounds **3i** and **3k** were further analyzed to evaluate their antitumor properties against the growth of the AR^+^LNCaP prostate cancer cell line. The BioVision Annexin V-FITC apoptosis detection kit (BioVision, Cambridge, UK) was used to evaluate the AR^+^LNCaP cell apoptosis after applying the IC_50_ concentrations of the test compounds in the presence of 1 nM DHT. Untreated AR^+^LNCaP cells and Enzalutamide reference drug were also used as negative and positive controls in this assay, respectively. Results of the AR^+^LNCaP cell apoptosis assay are shown in Table 4 and Figure 6.

The application of IC_50_ concentrations of Enzalutamide reference drug, compound **3i**, and compound **3k** displays variable effects on the growth of AR^+^LNCaP cells under the assay conditions (Table 4). By comparing the results observed for the test compounds **3i** and **3k** with the results observed for the reference drug Enzalutamide, it is obvious that the effect of compound **3k** on AR^+^LNCaP cell apoptosis is greater than the Enzalutamide reference drug. Compound **3k** records a 1.9-fold increase in the percentage of necrotic cells and a 1.3-fold increase in the percentage of early apoptotic cells compared to Enzalutamide. Although compound **3i** caused significant induction of AR^+^LNCaP cell apoptosis (11.47% early apoptosis, 7.55% late apoptosis, and 2.77% necrosis), the effect of compound **3i** on AR^+^LNCaP cell apoptosis was lower when compared to both Enzalutamide and compound **3k**. The observed results suggest potential proapoptotic effects of compounds **3i** and **3k**, with a superior effect of compound **3k**. 

Cell apoptosis is widely considered to be one of the major parameters that incur growth loss to cancer cells. Therefore, the synthesized compounds in the current study can induce cytotoxicity and cell apoptosis of AR^+^LNCaP prostate cancer cells. They could be therefore effectively used as promising safe cure materials for the treatment of prostate cancer diseases. 

### 3.3. Structure Activity Relationship (SAR)

The preliminary results of the biological assays showed that the compounds under investigation, **3a–p**, were potentially effective cytotoxic against PC-3 and LNCaP cells. Their efficiency, selectivity, and safety index were significantly affected by benzene ring substituents. Obviously, compound **3k**, which contains a fluoro substituent on the benzene ring (derived primary from phenyl hydrazine) and trifluoromethyl substituent on the other benzene ring (derived primary from acetophenone), enabled the scaffold to exhibit optimum anticancer profile regarding potency, as detected from IC_50_ values of 5.22 ± 0.12, selectivity towards the androgen sensitive LNCaP cell line (AR^-^/AR^+^ = 2.7), and safety index (Wi38/LNCaP = 3.8). However, its positional isomer **3o** showed a ≈2.25-fold lower potency (IC_50 =_11.75 ± 0.07) and selectivity to LNCaP (AR^-^/AR^+^ = 1.4). This indicates that both type of substituents and their positions are vital for effective binding to the biological target. Changing the trifluoromethyl group of compound **3k** by other substituents such as F, Cl, Br, H, CH_3_, and OCH_3_ led to a variable reduction in the cytotoxic activities, safety, and selectivity towards androgen-sensitive cancer cells (Table 1 and Scheme 1). However, compared to **3k**, the chloro substituent in compound **3i** exhibited reduced but comparable results regarding cytotoxicity (IC_50 =_ 06.08 ± 0.04), selectivity (AR^-^/AR^+^ = 2.6), and safety index (Wi38/LNCaP = 3.6). Replacement of the fluoro substituent of **3k** with a trifluoromethyl group in compound **3p** resulted in similar selectivity towards LNCaP and a slight decrease in the observed potency (IC_50 =_ 05.91 ± 0.13) and safety index (Wi38/LNCaP =3.4).

## 4. Conclusions

The current study reveals the synthesis of new pyrazolylmethylene-2-thioxoimidazolidin-4-one derivatives as anti-prostate-cancer agents via targeting the androgen receptor. The design was based on a combination of two pharmacophoric moieties, namely, 2-thioxoimidazolidin-4-one and pyrazole scaffolds that exist in FDA-approved prostate cancer drugs enzalutamide and darolutamide. The biological analyses and antiproliferative activity of the synthesized compounds (**3a–p**) as potential candidates for prostate cancer therapy (Figure 2) showed excellent anticancer properties against the tested AR^+^LNCaP and AR^-^PC-3 prostate cancer cell lines. Of these, compounds **3i–k**, **3m**, and **3o–p** exhibited the significant in vitro cytotoxicity and they were especially selective against the AR^+^LNCaP cell line (IC_50_ values range: 05.22 ± 0.12 to 11.75 ± 0.07 µM). These compounds have been shown to cause enhanced accumulation of the apoptotic pathway executioner, Caspase 3 in AR^+^LNCaP cells, with superior action of compounds **3i** and **3k**. DNA content analysis of AR^+^LNCaP cells treated with compounds **3i** and **3k** in the presence of 1 nM DHT provided evidence for their ability to reduce DNA contents at the various stages of the cell cycle (Table 4). Therefore, compounds **3i** and **3k** can trigger the apoptotic pathway, consequently causing AR^+^LNCaP cell growth arrest. The overall results observed in the current study support the efficacy of the synthesized analogs of androgen receptor blockers as potential anticancer drugs in the treatment of prostate cancer tumors. However, more investigations have to be performed to elucidate their exact mode of action and their safety aspects when applied to prostate cancer patients.

## Figures and Tables

**Table 1:** In vitro cytotoxicity assay of the test compounds **3a–p** (24 h incubation).

Compound	IC_50_ (µM)			Selective Index	
	AR^+^Lncap	AR^-^PC-3	Wi38	Wi38/Lncap	Wi38/PC-3
**3a**	26.57 ± 0.04	16.76 ± 0.34	63.27 ± 0.18	2.4	3.8
**3b**	20.79 ± 0.06	21.92 ± 0.12	24.26 ± 0.06	1.0	0.9
**3c**	109.72 ± 2.06	20.87 ± 1.12	87.88 ± 2.96	0.8	4.2
**3d**	23.53 ± 0.74	177.25 ± 0.61	48.19 ± 0.21	2.0	0.3
**3e**	33.90 ± 0.12	20.06 ± 0.21	63.24 ± 0.26	1.9	3.2
**3f**	54.19 ± 0.11	20.19 ± 0.04	56.56 ± 0.14	1.0	2.8
**3g**	47.31 ± 0.18	26.79 ± 0.39	56.62 ± 0.16	1.2	2.1
**3h**	19.48 ± 0.05	18.80 ± 0.14	20.96 ± 0.13	1.0	1.0
**3i**	12.07 ± 0.09	19.51 ± 0.21	29.93 ± 0.12	2.5	1.5
**3j**	13.89 ± 0.14	24.44 ± 0.12	31.18 ± 0.16	2.2	1.5
**3k**	10.27 ± 0.14	22.47 ± 0.29	32.81 ± 0.08	3.2	1.5
**3l**	20.80 ± 0.04	15.30 ± 0.04	26.81 ± 0.06	1.3	1.8
**3m**	12.97 ± 0.06	22.48 ± 0.46	30.54 ± 0.08	2.4	1.3
**3n**	15.02 ± 0.15	23.25 ± 0.08	26.93 ± 0.09	1.8	1.2
**3o**	12.66 ± 0.11	21.08 ± 0.10	25.08 ± 0.08	2.0	1.2
**3p**	12.11 ± 0.08	23.20 ± 0.12	25.35 ± 0.12	2.1	1.0
Doxorubicin	11.39 ± 0.16	10.02 ± 0.18	15.14 ± 0.12	1.3	1.5
Enzalutamide	7.32 2 ± 0.05	15.291± 0.19	27.52 ± 0.36	3.7	1.8

IC_50_ values measured after 24 h of incubation after applying the target compounds. Values are represented as means ± SE from at least three independent measurements. AR^+^LNCaP and AR^-^PC-3: androgen-sensitive and androgen-insensitive human prostate cancer cell lines; Wi38: normal human lung fibroblast cell line.

**Table 2:** In vitro cytotoxicity of compounds **3i–k**, **3m**, and **3o–p** (48 h incubation).

Compound	IC_50_ (µM)			Selectivity	Safety Index
	AR^+^LNCaP	AR^-^PC-3	Wi38	AR^-^/AR^+^	Wi38/LNCaP
**3i**	06.08 ± 0.04	15.52 ± 0.12	21.93 ± 0.14	2.6	3.6
**3j**	07.72 ± 0.15	14.22 ± 0.15	21.08 ± 0.06	1.8	2.7
**3k**	05.22 ± 0.12	13.87 ± 0.18	19.81 ± 0.07	2.7	3.8
**3m**	10.46 ± 0.11	13.11 ± 0.14	23.33 ± 0.19	1.3	2.2
**3o**	11.75 ± 0.07	16.42 ± 0.17	22.35 ± 0.13	1.4	1.9
**3p**	05.91 ± 0.13	15.16 ± 0.25	20.18 ± 0.14	2.7	3.4
Enzalutamide	06.52 ± 0.16	13.58 ± 0.27	21.53 ± 0.32	2.1	3.3

IC_50_ values for the test compounds **3i–k**, **3m**, and **3o–p** in the presence of 1 nM DHT measured after 48 h of incubation. IC_50_ values are taken as means ± SE from at least three independent measurements. AR^+^LNCaP and AR^-^PC-3: androgen-sensitive and androgen-insensitive human prostate cancer cell lines; Wi38: normal human lung fibroblast cell line.

**Table 3:** Quantitative evaluation of DNA contents of AR^+^LNCaP cells at the different stages of the cell cycle after treatment with the test compounds **3i** and **3k**.

Sample	DNA Content (%)			Cell Growth Arrest Stage
	G_0_–G_1_	S	G_2_/M	
Untreated AR^+^LNCaP	54.99	18.66	26.35	nd
**3i**	58.33	19.19	22.48	G1/S
**3k**	65.21	15.61	19.18	G1
Enzalutamide	61.02	13.61	25.37	G1

G_0_, G_1_, S, G_2_, and M: the different stages of the cell cycle; nd: not detected.

**Table 4:** Effect of compounds **3i** and **3k** on different stages of AR^+^LNCaP cell apoptosis in the presence of DHT.

Compound	Total Apoptosis (%)	Early Apoptosis (%)	Late Apoptosis (%)	Necrotic Cells (%)
**Untreated control**	2.39	0.61	0.22	1.56
**3i**	21.79	11.47	7.55	2.77
**3k**	35.02	12.11	16.89	6.02
**Enzalutamide**	33.86	9.46	21.27	3.13

## Data Availability

The datasets used and analyzed during the current study are available from the corresponding author upon reasonable request.

## Supplementary Materials

The following supporting information can be downloaded at: https://www.mdpi.com/article/10.3390/biom14070811/s1. File S1: ^1^H and ^13^C-NMR spectra for compounds **3a–p**.

## References

[1] Ettridge K., Bowden J., Chambers S., Smith D., Murphy M., Evans S., Roder D., Miller C. (2018). “Prostate cancer is far more hidden…”: Perceptions of stigma, social isolation and help-seeking among men with prostate cancer. Eur. J. Cancer Care.

[2] Msaouel P., Pissimissis N., Halapas A., Koutsilieris M. (2008). Mechanisms of bone metastasis in prostate cancer: Clinical implications. Best Pract. Res. Clin. Endocrinol. Metab..

[3] Litwin M.S., Tan H.-J. (2017). The diagnosis and treatment of prostate cancer: A review. JAMA.

[4] Abrahamsson P.-A. (2010). Potential benefits of intermittent androgen suppression therapy in the treatment of prostate cancer: A systematic review of the literature. Eur. Urol..

[5] Kunath F., Grobe H.R., Ruecker G., Motschall E., Antes G., Dahm P., Wullich B., Meerpohl J.J. (2014). Non-steroidal antiandrogen monotherapy compared with luteinising hormone–releasing hormone agonists or surgical castration monotherapy for advanced prostate cancer. Cochrane Database Syst. Rev..

[6] Thompson I.M. (2001). Flare associated with LHRH-agonist therapy. Rev. Urol..

[7] Hoda M.R., Kramer M.W., Merseburger A.S., Cronauer M.V. (2017). Androgen deprivation therapy with Leuprolide acetate for treatment of advanced prostate cancer. Expert Opin. Pharmacother..

[8] Fontana D., Mari M., Martinelli A., Boccafoschi C., Magno C., Turriziani M., Maymone S., Cunico S.C., Zanollo A., Montagna G. (2003). 3-month formulation of goserelin acetate (‘Zoladex’10.8-mg depot) in advanced prostate cancer: Results from an Italian, open, multicenter trial. Urol. Int..

[9] Millar J.L. (2000). Triptorelin Approved for Prostate Cancer Treatment.

[10] Djavan B., Schlegel P., Salomon G., Eckersberger E., Sadri H., Graefen M. (2010). Analysis of testosterone suppression in men receiving histrelin, a novel GnRH agonist for the treatment of prostate cancer. Can. J. Urol..

[11] Carter N.J., Keam S.J. (2014). Degarelix: A review of its use in patients with prostate cancer. Drugs.

[12] Cherneva E., Atanasova M., Buyukliev R., Tomovic K., Smelcerovic Z., Bakalova A., Smelcerovic A. (2020). 3′-Methyl-4-thio-1H-tetrahydropyranspiro-5′-hydantoin platinum complex as a novel potent anticancer agent and xanthine oxidase inhibitor. Arch. Pharm..

[13] Zhang M., Liang Y.-R., Li H., Liu M.-M., Wang Y. (2017). Design, synthesis, and biological evaluation of hydantoin bridged analogues of combretastatin A-4 as potential anticancer agents. Bioorganic Med. Chem..

[14] Yao C.-H., Hsieh T.-C., Song J.-S., Lee J.-C. (2020). Design, synthesis and anticancer evaluation of β-carboline-1-one hydantoins. Future Med. Chem..

[15] Ganatra S., Patle M., Bhagat G. (2011). Studies of Quantitative Structure-Activity Relationship (QSAR) of Hydantoin Based Active Anti-Cancer Drugs. Asian J. Res. Chem..

[16] Azizmohammadi M., Khoobi M., Ramazani A., Emami S., Zarrin A., Firuzi O., Miri R., Shafiee A. (2013). 2H-chromene derivatives bearing thiazolidine-2, 4-dione, rhodanine or hydantoin moieties as potential anticancer agents. Eur. J. Med. Chem..

[17] Zuliani V., Carmi C., Rivara M., Fantini M., Lodola A., Vacondio F., Bordi F., Plazzi P.V., Cavazzoni A., Galetti M. (2009). 5-Benzylidene-hydantoins: Synthesis and antiproliferative activity on A549 lung cancer cell line. Eur. J. Med. Chem..

[18] Cao S., Rogers J., Yeo J., Anderson-Steele B., Ashby J., David S.S. (2020). 2′-Fluorinated Hydantoins as Chemical Biology Tools for Base Excision Repair Glycosylases. ACS Chem. Biol..

[19] Kumar C.A., Swamy S.N., Sugahara K., Rangappa K.S. (2009). Anti-tumor and anti-angiogenic activity of novel hydantoin derivatives: Inhibition of VEGF secretion in liver metastatic osteosarcoma cells. Bioorganic Med. Chem..

[20] Spengler G., Handzlik J., Ocsovszki I., Viveiros M., Kieć-Kononowicz K., Molnar J., Amaral L. (2011). Modulation of multidrug efflux pump activity by new hydantoin derivatives on colon adenocarcinoma cells without inducing apoptosis. Anticancer. Res..

[21] El-Atawy M.A., Alshaye N.A., Elrubi N., Hamed E.A., Omar A.Z. (2022). Pyrimidines-Based Heterocyclic Compounds: Synthesis, Cytoxicity Evaluation and Molecular Docking. Molecules.

[22] Taher A.T., Mostafa Sarg M.T., El-Sayed Ali N.R., Hilmy Elnagdi N. (2019). Design, synthesis, modeling studies and biological screening of novel pyrazole derivatives as potential analgesic and anti-inflammatory agents. Bioorganic Chem..

[23] Abdelwahed R.E., Radhi A.H., Awad H.M., El Gokha A.A., Goda A.E.S., El Sayed I.E.-T. (2021). Synthesis and Anti-Proliferative Activity of New α-Amino Phosphonate Derivatives Bearing Heterocyclic Moiety. Pharm. Chem. J..

[24] Harikrishna N., Isloor A.M., Ananda K., Obaid A., Fun H.-K. (2015). 1,3,4-Trisubstituted pyrazole bearing a 4-(chromen-2-one) thiazole: Synthesis, characterization and its biological studies. RSC Adv..

[25] Wang Y.-T., Shi T.-Q., Fu J., Zhu H.-L. (2019). Discovery of novel bacterial FabH inhibitors (Pyrazol-Benzimidazole amide derivatives): Design, synthesis, bioassay, molecular docking and crystal structure determination. Eur. J. Med. Chem..

[26] Shrivastava N., Khan S.A., Alam M.M., Akhtar M., Srivastava A., Husain A. (2023). Anticancer heterocyclic hybrids: Design, synthesis, molecular docking and evaluation of new thiazolidinone-pyrazoles. Z. Für Naturforschung B.

[27] Nandurkar Y., Bhoye M.R., Maliwal D., Pissurlenkar R.R.S., Chavan A., Katade S., Mhaske P.C. (2023). Synthesis, biological screening and in silico studies of new N-phenyl-4-(1,3-diaryl-1H-pyrazol-4-yl)thiazol-2-amine derivatives as potential antifungal and antitubercular agents. Eur. J. Med. Chem..

[28] Khonde L.P., Müller R., Boyle G.A., Reddy V., Nchinda A.T., Eyermann C.J., Fienberg S., Singh V., Myrick A., Abay E. (2021). 1,3-Diarylpyrazolyl-acylsulfonamides as Potent Anti-tuberculosis Agents Targeting Cell Wall Biosynthesis in Mycobacterium tuberculosis. J. Med. Chem..

[29] Lv X.-H., Xiao J.-J., Ren Z.-L., Chu M.-J., Wang P., Meng X.-F., Li D.-D., Cao H.-Q. (2015). Design, synthesis and insecticidal activities of N-(4-cyano-1-phenyl-1H-pyrazol-5-yl)-1,3-diphenyl-1H-pyrazole-4-carboxamide derivatives. RSC Adv..

[30] Yu D.D., Lin W., Forman B.M., Chen T. (2014). Identification of trisubstituted-pyrazol carboxamide analogs as novel and potent antagonists of farnesoid X receptor. Bioorganic Med. Chem..

[31] Alshabani L.A., Kumar A., Willcocks S.J., Srithiran G., Bhakta S., Estrada D.F., Simons C. (2022). Synthesis, biological evaluation and computational studies of pyrazole derivatives as Mycobacterium tuberculosis CYP121A1 inhibitors. RSC Med. Chem..

[32] Hwang J.Y., Kim H.-Y., Park D.-S., Choi J., Baek S.M., Kim K., Kim S., Seong S., Choi I., Lee H.-g. (2013). Identification of a series of 1,3,4-trisubstituted pyrazoles as novel hepatitis C virus entry inhibitors. Bioorganic Med. Chem. Lett..

[33] Mosmann T. (1983). Rapid colorimetric assay for cellular growth and survival: Application to proliferation and cytotoxicity assays. J. Immunol. Methods.

[34] Van de Loosdrecht A., Beelen R., Ossenkoppele g., Broekhoven M., Langenhuijsen M. (1994). A tetrazolium-based colorimetric MTT assay to quantitate human monocyte mediated cytotoxicity against leukemic cells from cell lines and patients with acute myeloid leukemia. J. Immunol. Methods.

[35] Elancheran R., Saravanan K., Divakar S., Kumari S., Maruthanila V.L., Kabilan S., Ramanathan M., Devi R., Kotoky J. (2017). Design, synthesis and biological evaluation of novel 1, 3-thiazolidine-2, 4-diones as anti-prostate cancer agents. Anti-Cancer Agents Med. Chem. (Former. Curr. Med. Chem.-Anti-Cancer Agents).

[36] Lee S.-T., Wong P.-F., Cheah S.-C., Mustafa M.R. (2011). Alpha-tomatine induces apoptosis and inhibits nuclear factor-kappa B activation on human prostatic adenocarcinoma PC-3 cells. PLoS ONE.

[37] Kumi-Diaka J., Sanderson N.A., Hall A. (2000). The mediating role of caspase-3 protease in the intracellular mechanism of genistein-induced apoptosis in human prostatic carcinoma cell lines, DU145 and LNCaP. Biol. Cell.

[38] El-Sheikh S.M., Khairy M.H., Osama E., Metwally M.M., Galal A.A. (2021). Nanotechnology improves the therapeutic efficacy of gemcitabine against a human hepatocellular carcinoma cell line and minimizes its in vivo side effects. Naunyn-Schmiedeberg’s Arch. Pharmacol..

[39] Ozgur O., Karti S., Sonmez M., Yilmaz M., Karti D., Ozdemir F., Ovali E. (2003). Effects of interferon-alfa-2a on human hepatoma HepG2 cells. Exp. Oncol..

[40] Khanfar M.A., El Sayed K.A. (2010). Phenylmethylene hydantoins as prostate cancer invasion and migration inhibitors. CoMFA approach and QSAR analysis. Eur. J. Med. Chem..

[41] El-Atawy M.A., Alsubaie M.S., Alazmi M.L., Hamed E.A., Hanna D.H., Ahmed H.A., Omar A.Z. (2023). Synthesis, characterization, and anticancer activity of new N, N′-Diarylthiourea derivative against breast cancer cells. Molecules.

[42] El-Atawy M.A., Omar A.Z., Alazmi M.L., Alsubaie M.S., Hamed E.A., Ahmed H.A. (2023). Synthesis and characterization of new imine liquid crystals based on terminal perfluoroalkyl group. Heliyon.

[43] Anand S.A.A., Loganathan C., Thomas N.S., Saravanan K., Alphonsa A.T., Kabilan S. (2015). Synthesis, structure prediction, pharmacokinetic properties, molecular docking and antitumor activities of some novel thiazinone derivatives. New J. Chem..

[44] Elancheran R., Saravanan K., Choudhury B., Divakar S., Kabilan S., Ramanathan M., Das B., Devi R., Kotoky J. (2016). Design and development of oxobenzimidazoles as novel androgen receptor antagonists. Med. Chem. Res..

[45] Mabjeesh N.J., Willard M.T., Frederickson C.E., Zhong H., Simons J.W. (2003). Androgens stimulate hypoxia-inducible factor 1 activation via autocrine loop of tyrosine kinase receptor/phosphatidylinositol 3′-kinase/protein kinase B in prostate cancer cells. Clin. Cancer Res..

[46] Beer T., Armstrong A., Rathkopf D. (2014). September is Prostate Cancer Awareness Month!. N. Engl. J. Med..

[47] Luan H., Xu P., Meng Y., Li Z., Bian J. (2020). A critical update on the strategies towards modulators targeting androgen receptors. Bioorganic Med. Chem..

[48] Chen L., Zeng Y., Zhou S.-F. (2018). Role of apoptosis in cancer resistance to chemotherapy. Current Understanding of Apoptosis-Programmed Cell Death.

[49] Han J.-H., Tweedell R.E., Kanneganti T.-D. (2023). Evaluation of caspase activation to assess innate immune cell death. JoVE (J. Vis. Exp.).

[50] Kumar S. (1997). The apoptotic cysteine protease CPP32. Int. J. Biochem. Cell Biol..

[51] Kaufmann S.H., Earnshaw W.C. (2000). Induction of apoptosis by cancer chemotherapy. Exp. Cell Res..

[52] Johnson V., Ko S., Holmstrom T., Eriksson J., Chow S. (2000). Effector caspases are dispensable for the early nuclear morphological changes during chemical-induced apoptosis. J. Cell Sci..

