# Supplementary material for: Design, Synthesis, Characterization, and Cytotoxicity of New Pyrazolylmethylene-2-thioxoimidazolidin-4-one Derivatives towards Androgen-Sensitive LNCaP Prostate Cancer Cells
Source: Biomolecules. 2024 Jul 8;14(7):811. doi: 10.3390/biom14070811 (PMC11274643; doi:10.3390/biom14070811)
Supplement: Supplementary file 1 [file biomolecules-14-00811-s001.zip › biomolecules-3076579-supplementary.pdf]

Design, synthesis, characterization, and cytotoxicity of new pyrazolylmethylene-2-thioxoimidazolidin-4-one derivatives towards androgen sensitive LNCaP prostate cancer cells

Mohamed A. El-Atawy [1,2,*], Rashad Kebeish [3,4,*], Awatif Rashed Z. Almotairy [1] and Alaa Z. Omar [2]

[1] Chemistry Department, Faculty of Science, Taibah University, Yanbu 46423, Saudi Arabia;
[2] Chemistry Department, Faculty of Science, Alexandria University, P.O. 426 Ibrahemia, Alexandria 21321, Egypt
[3] Department of Biology, Faculty of Science in Yanbu, Taibah University, Yanbu 46423, Saudi Arabia
[4] Botany and Microbiology Department, Faculty of Science, Zagazig University, Zagazig 44519, Egypt
* Correspondence: maatawy@taibahu.edu.sa (M.A.E.-A.); rkebeish@taibahu.edu.sa (R.K.)

[*] Correspondence Mohamed A. El-Atawy (maatawy@taibahu.edu.sa), Rashad Kebeish (rke-beish@taibahu.edu.sa)

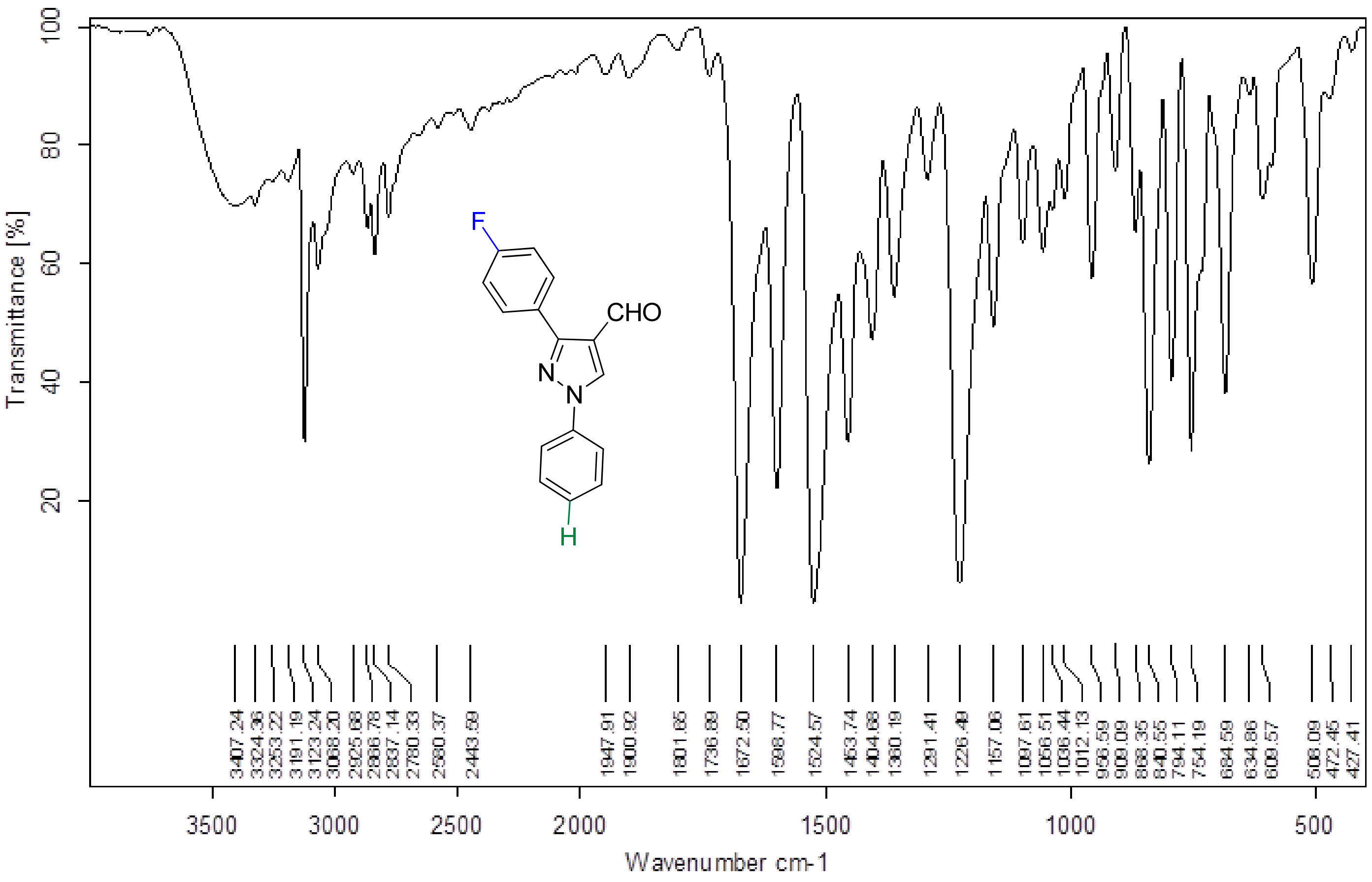

IR (KBr) spectrum of 3-(4-fluorophenyl)-1-phenyl-1*H*-pyrazole-4-carbaldehyde **2a**

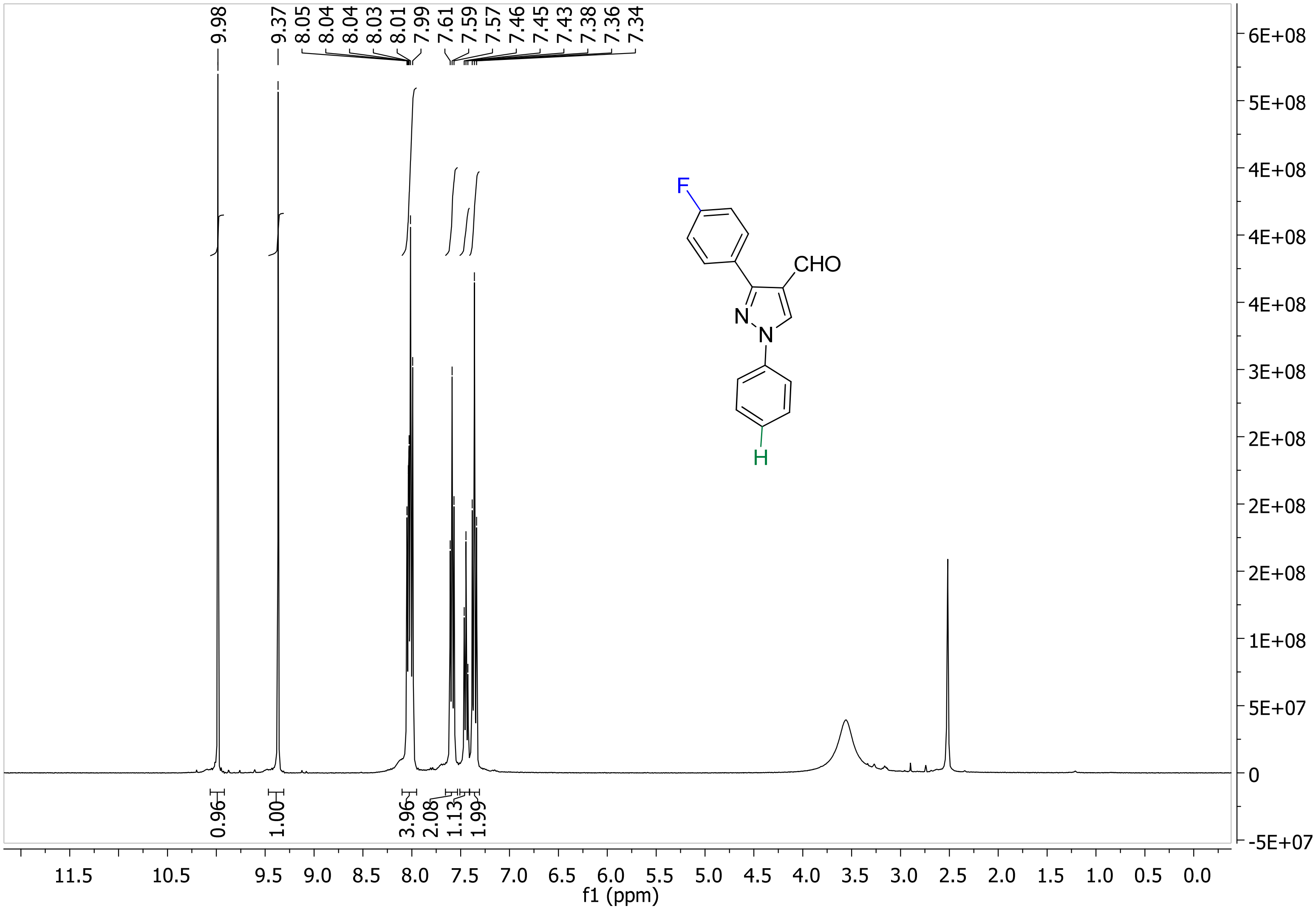

^{1}H NMR (DMSO-*d6*, 400 MHz) spectrum of 3-(4-fluorophenyl)-1-phenyl-1*H*-pyrazole-4-carbaldehyde **2a**

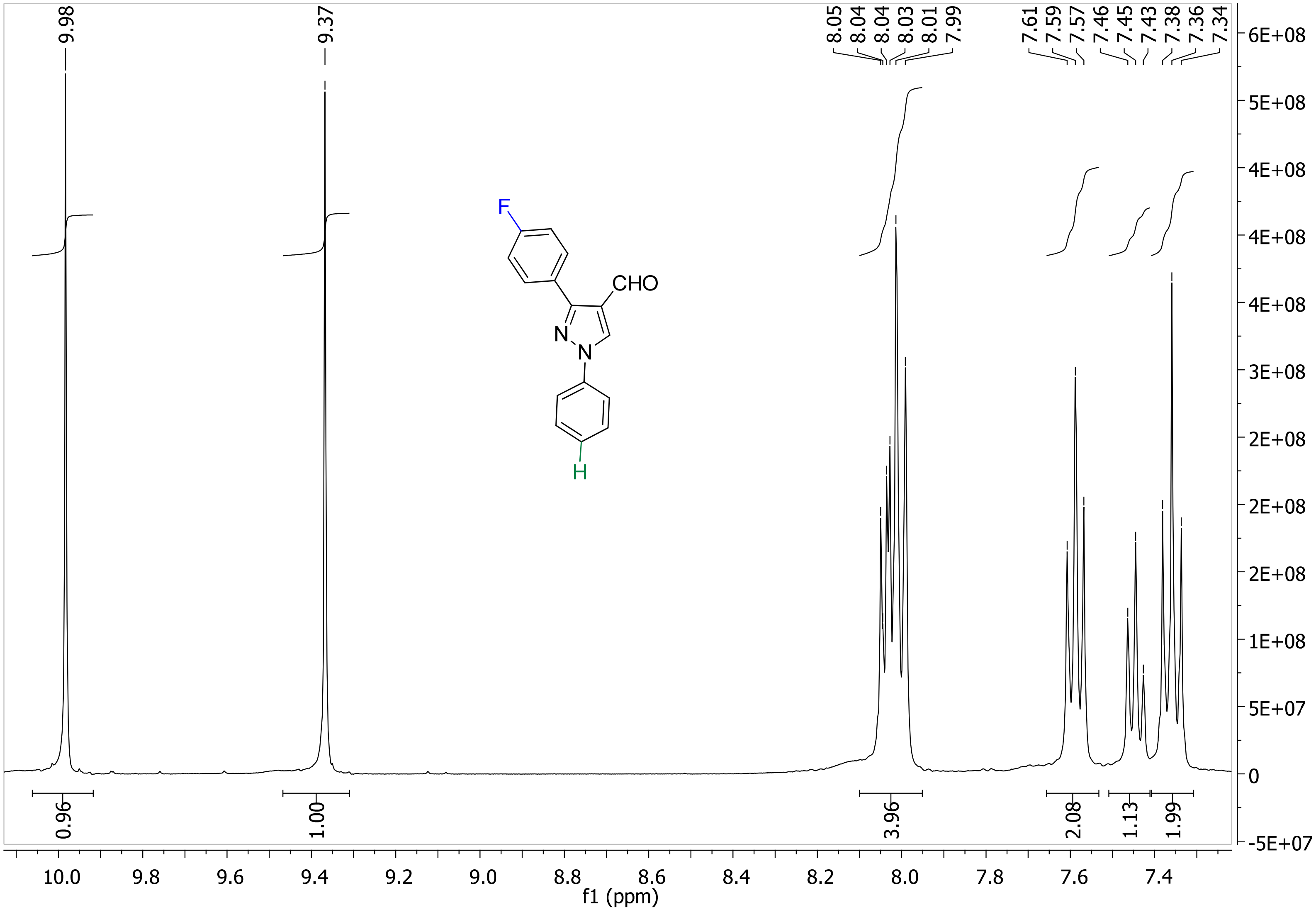

^{1}H NMR (DMSO-*d6*, 400 MHz) spectrum of 3-(4-fluorophenyl)-1-phenyl-1*H*-pyrazole-4-carbaldehyde **2a**

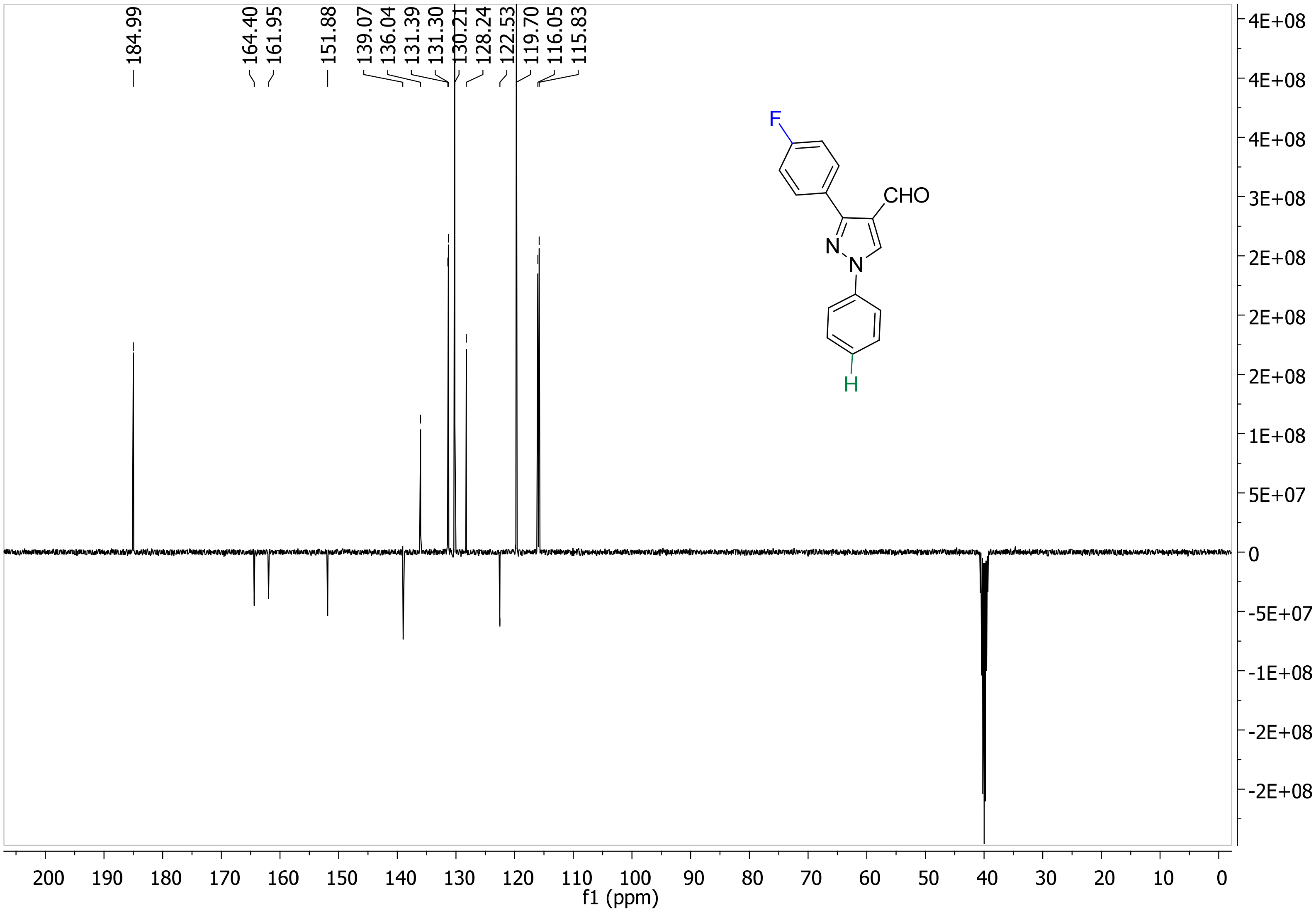

^{13}C APT NMR (DMSO-*d6*, 101 MHz) spectrum of 3-(4-fluorophenyl)-1-phenyl-1*H*-pyrazole-4-carbaldehyde **2a**

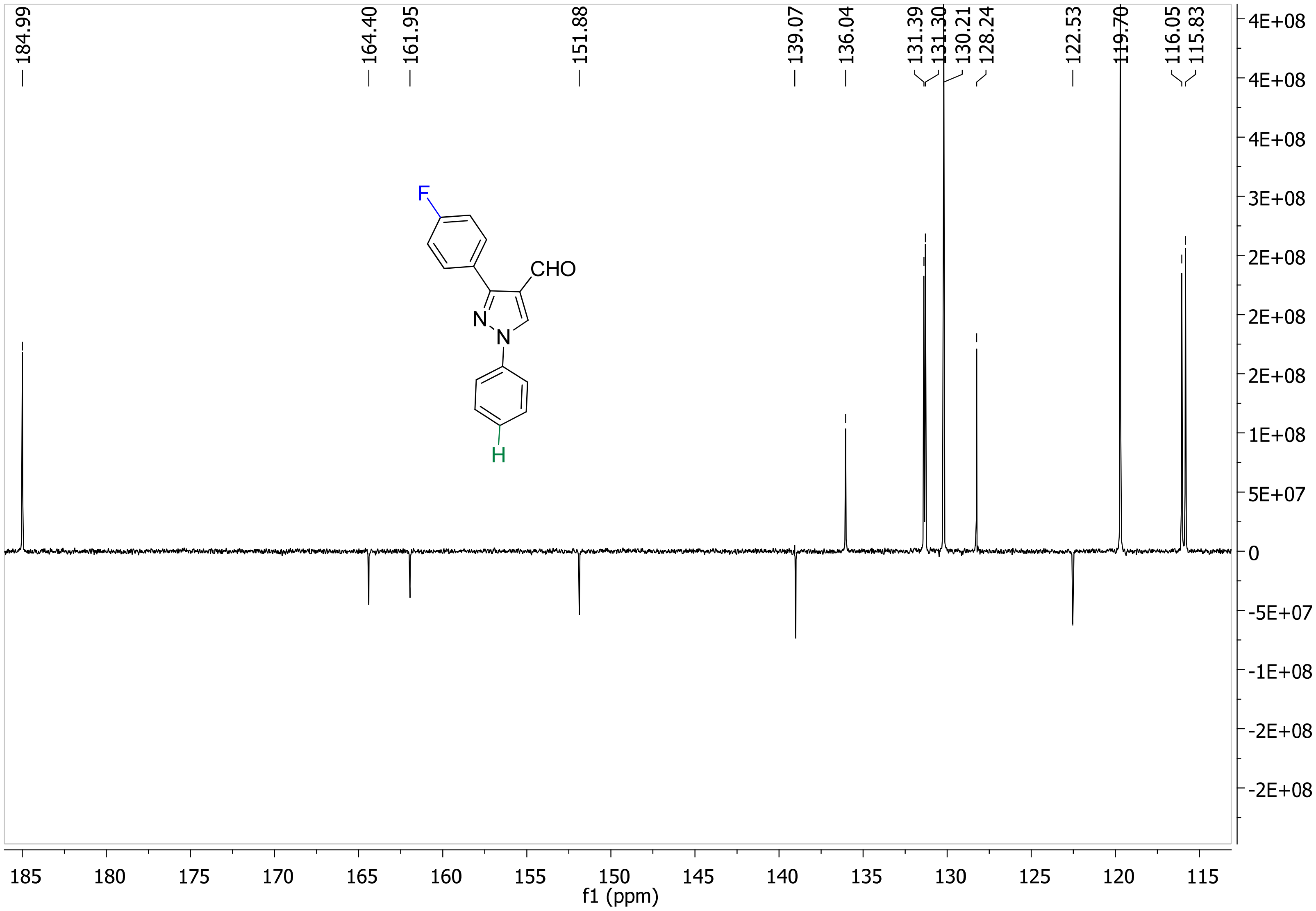

^{13}C APT NMR (DMSO-*d6*, 101 MHz) spectrum of 3-(4-fluorophenyl)-1-phenyl-1*H*-pyrazole-4-carbaldehyde **2a**

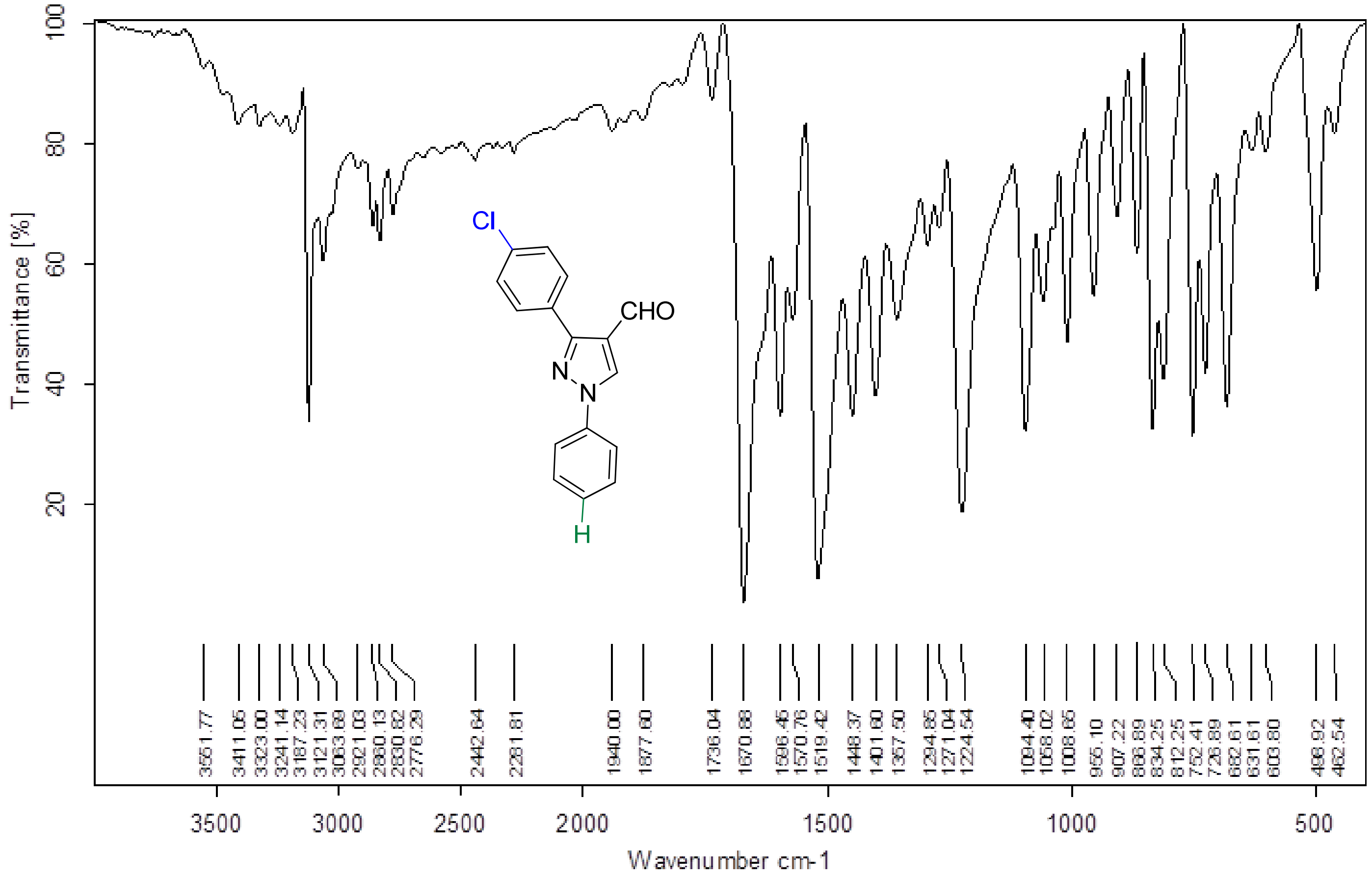

IR (KBr) spectrum of 3-(4-chlorophenyl)-1-phenyl-1*H*-pyrazole-4-carbaldehyde **2b**

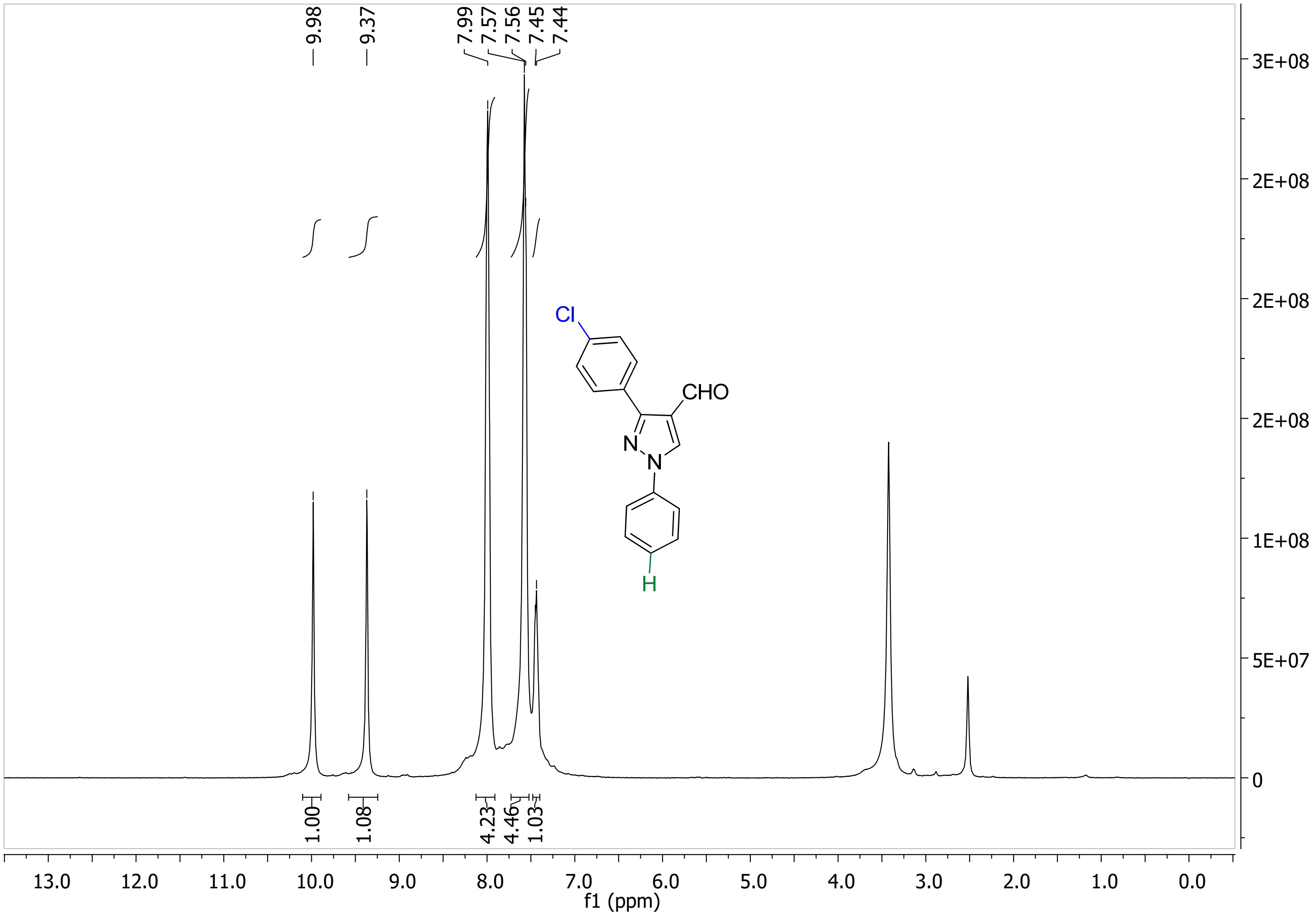

^{1}H NMR (DMSO-*d6*, 101 MHz) spectrum of 3-(4-chlorophenyl)-1-phenyl-1*H*-pyrazole-4-carbaldehyde **2b**

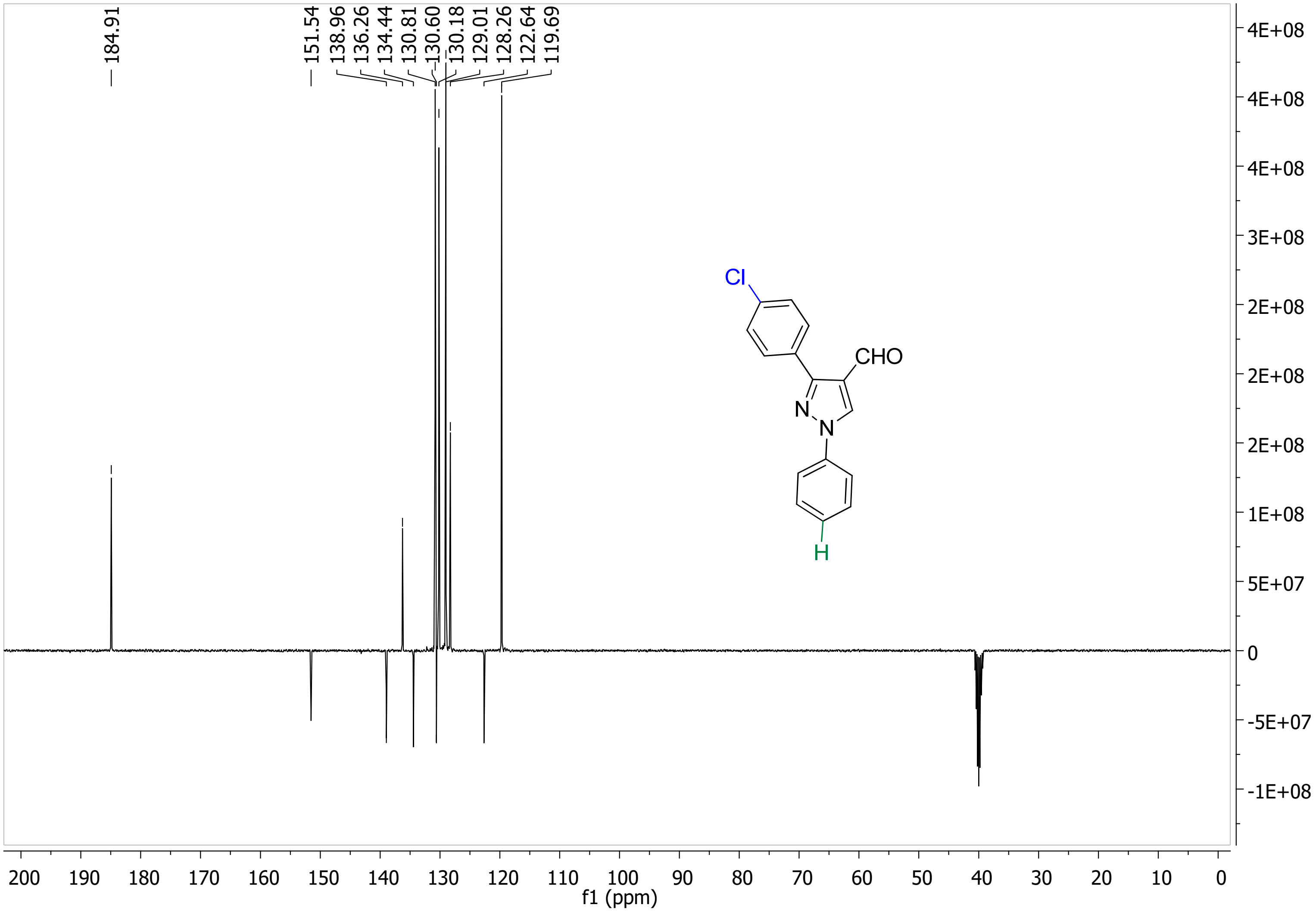

^{13}C APT NMR (DMSO-*d6*, 101 MHz) spectrum of 3-(4-chlorophenyl)-1-phenyl-1*H*-pyrazole-4-carbaldehyde **2b**

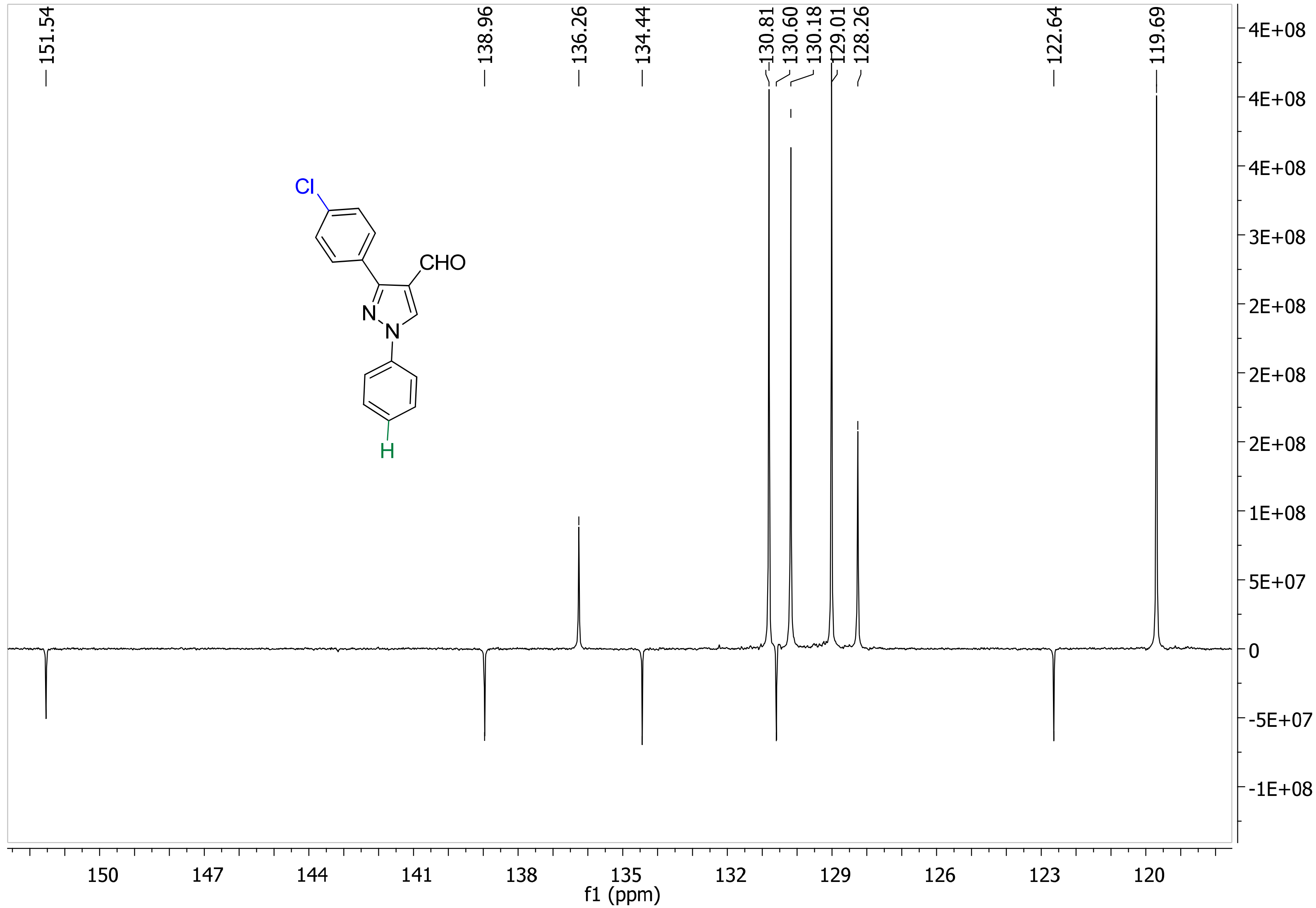

^{13}C APT NMR (DMSO-*d6*, 101 MHz) spectrum of 3-(4-chlorophenyl)-1-phenyl-1*H*-pyrazole-4-carbaldehyde **2b**

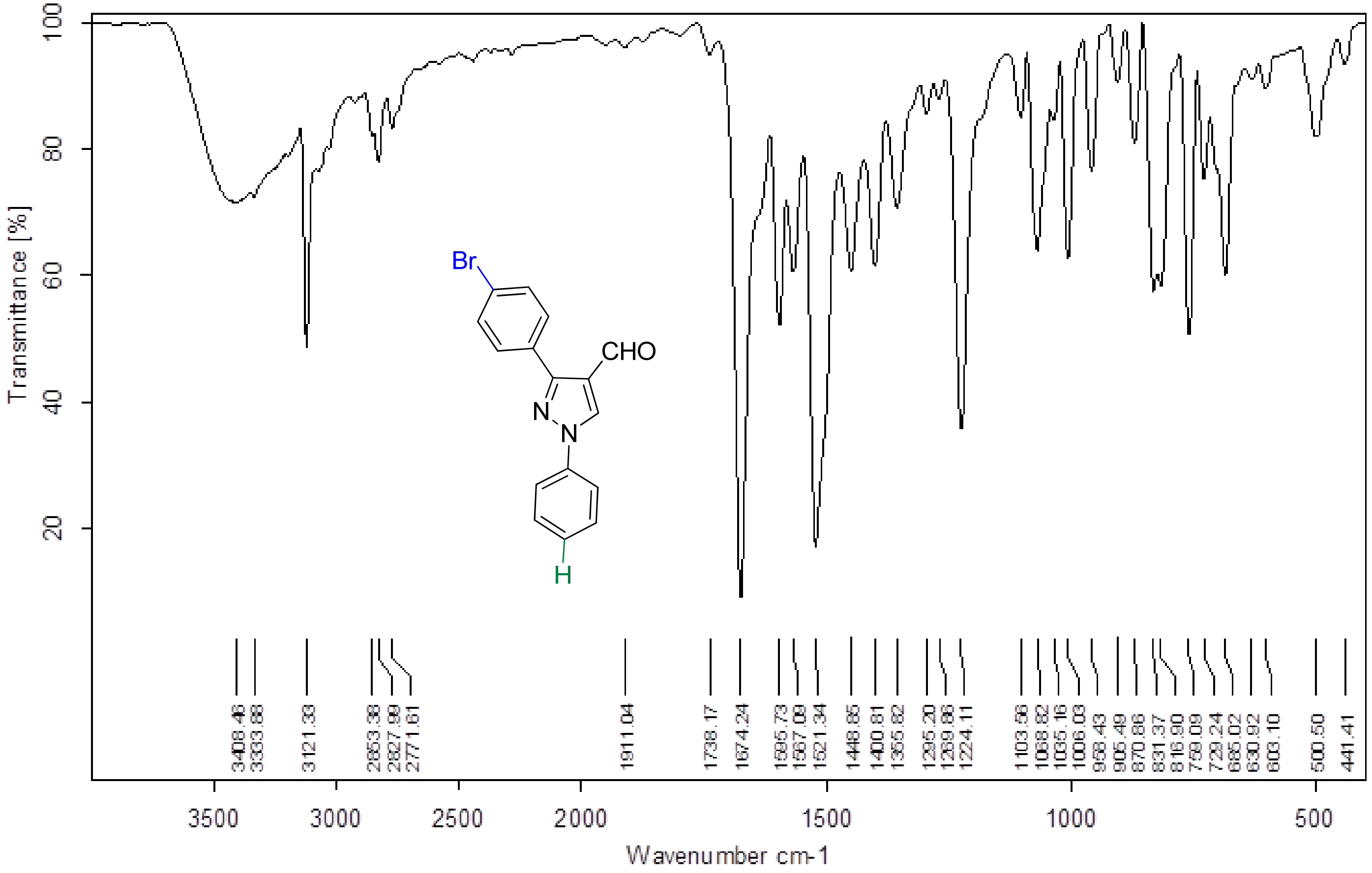

IR (KBr) spectrum of 3-(4-bromophenyl)-1-phenyl-1*H*-pyrazole-4-carbaldehyde **2c**

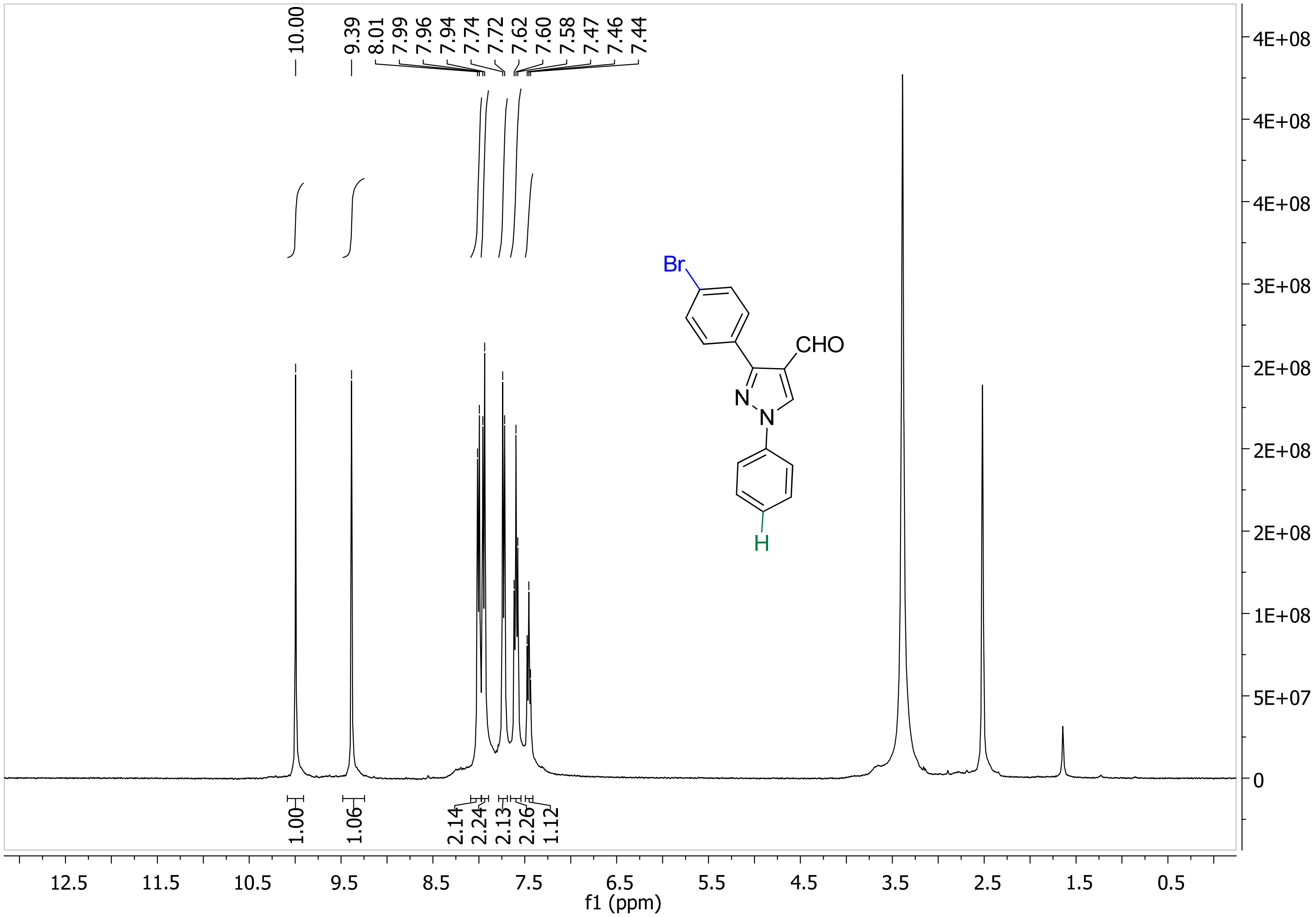

^{1}H NMR (DMSO-*d6*, 400 MHz) spectrum of 3-(4-bromophenyl)-1-phenyl-1*H*-pyrazole-4-carbaldehyde **2c**

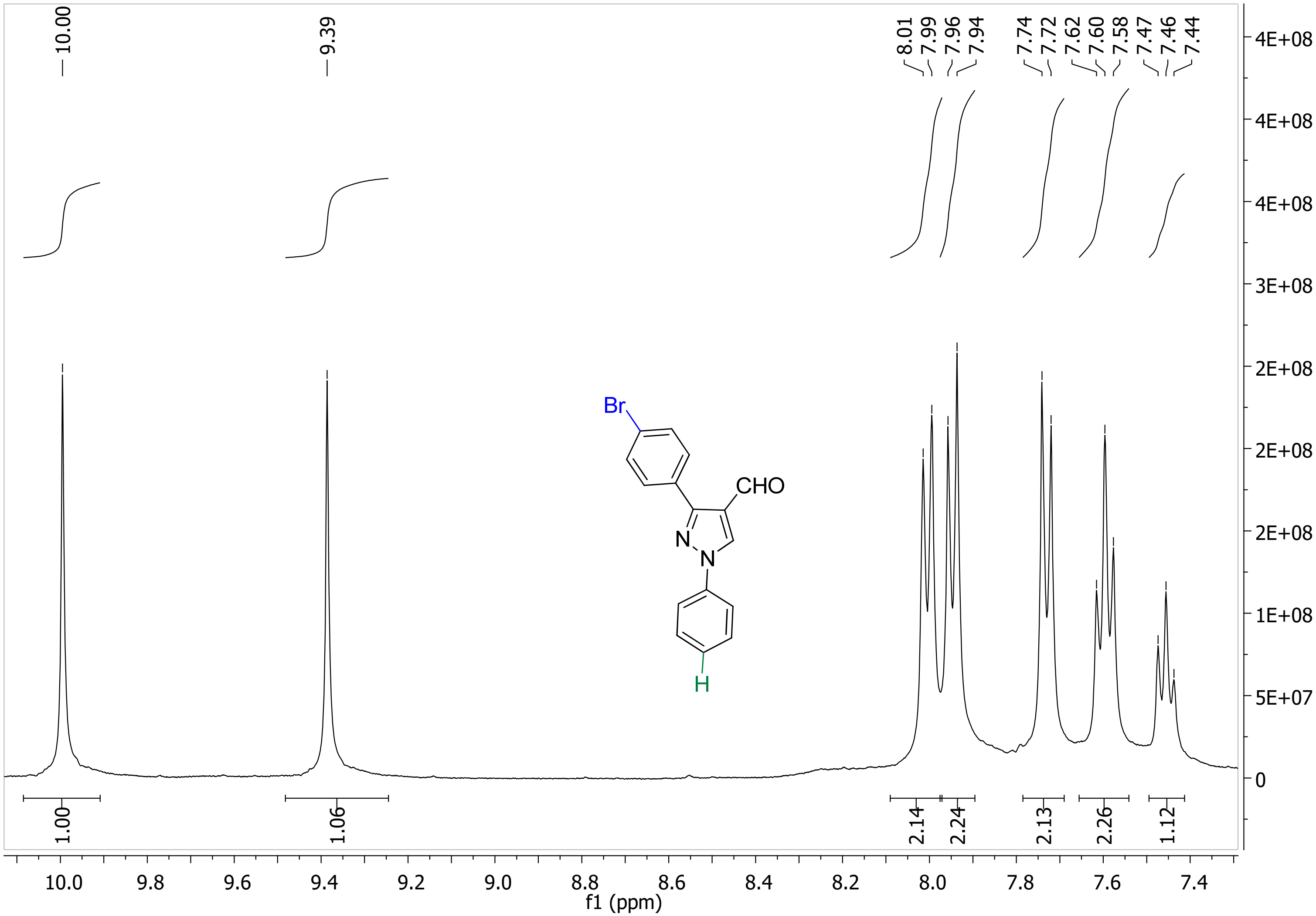

^{1}H NMR (DMSO-*d6*, 400 MHz) spectrum of 3-(4-bromophenyl)-1-phenyl-1*H*-pyrazole-4-carbaldehyde **2c**

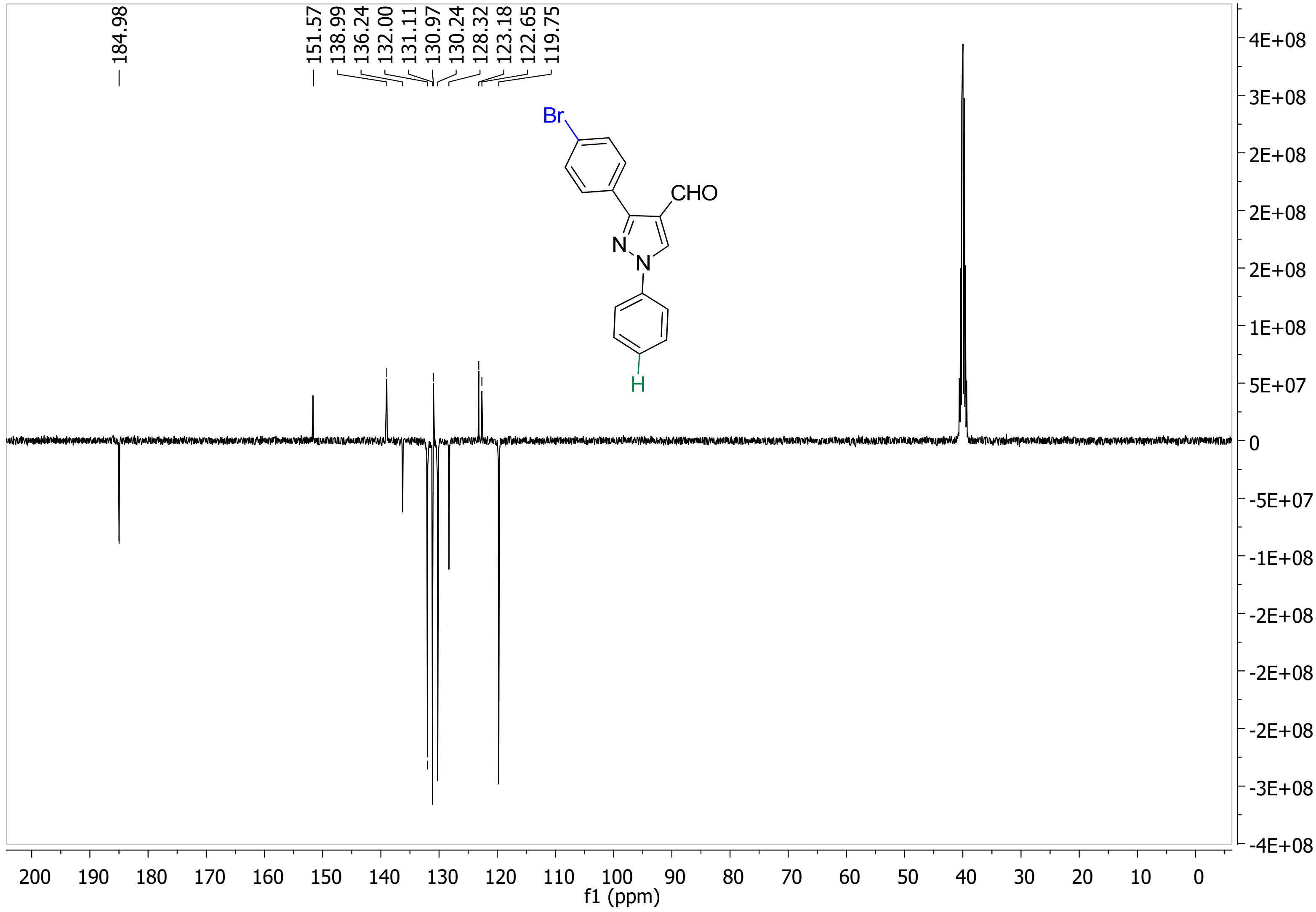

^{13}C APT NMR (DMSO-*d6*, 101 MHz) spectrum of 3-(4-bromophenyl)-1-phenyl-1*H*-pyrazole-4-carbaldehyde **2c**

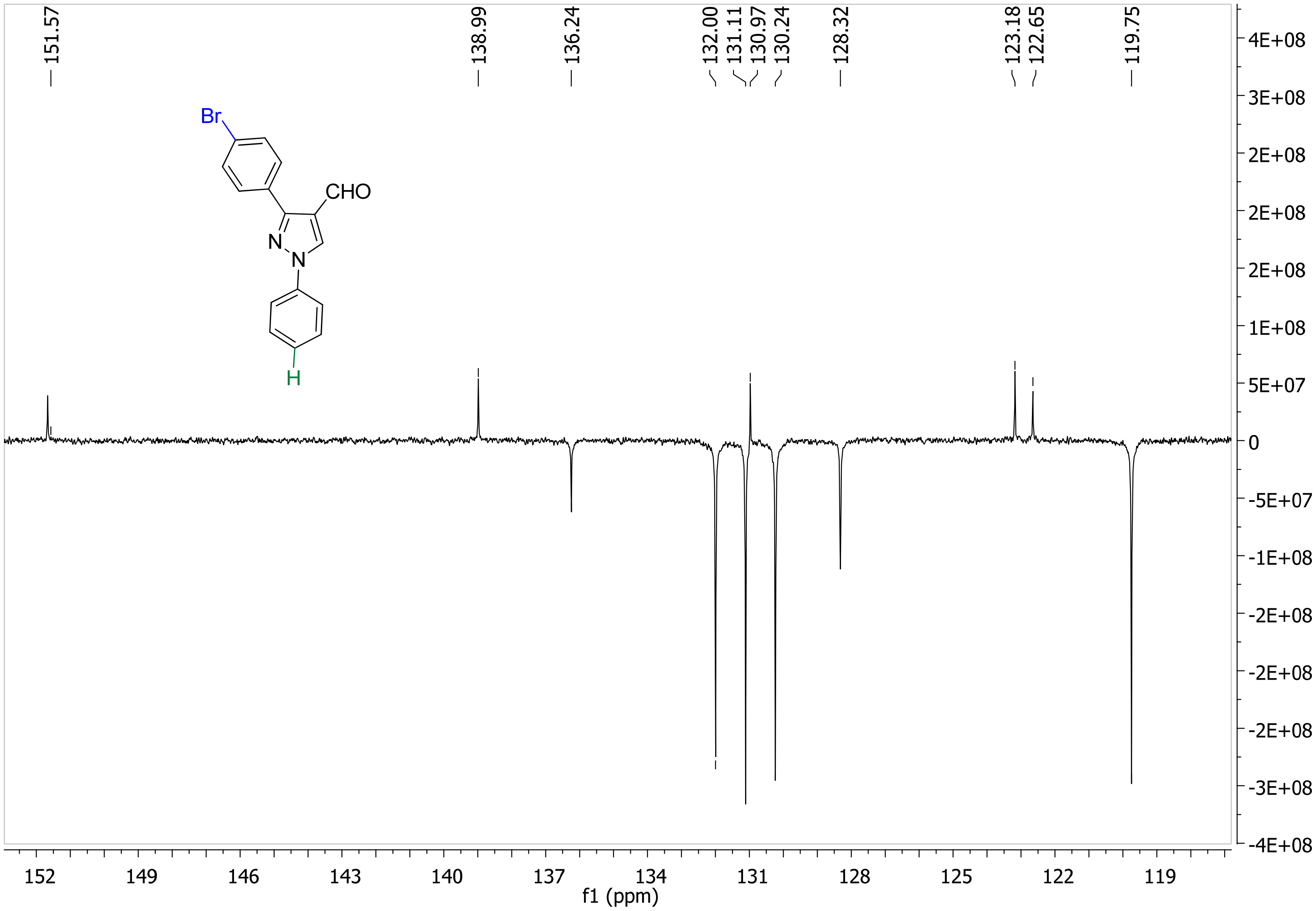

^{13}C APT NMR (DMSO-*d6*, 101 MHz) spectrum of 3-(4-bromophenyl)-1-phenyl-1*H*-pyrazole-4-carbaldehyde **2c**

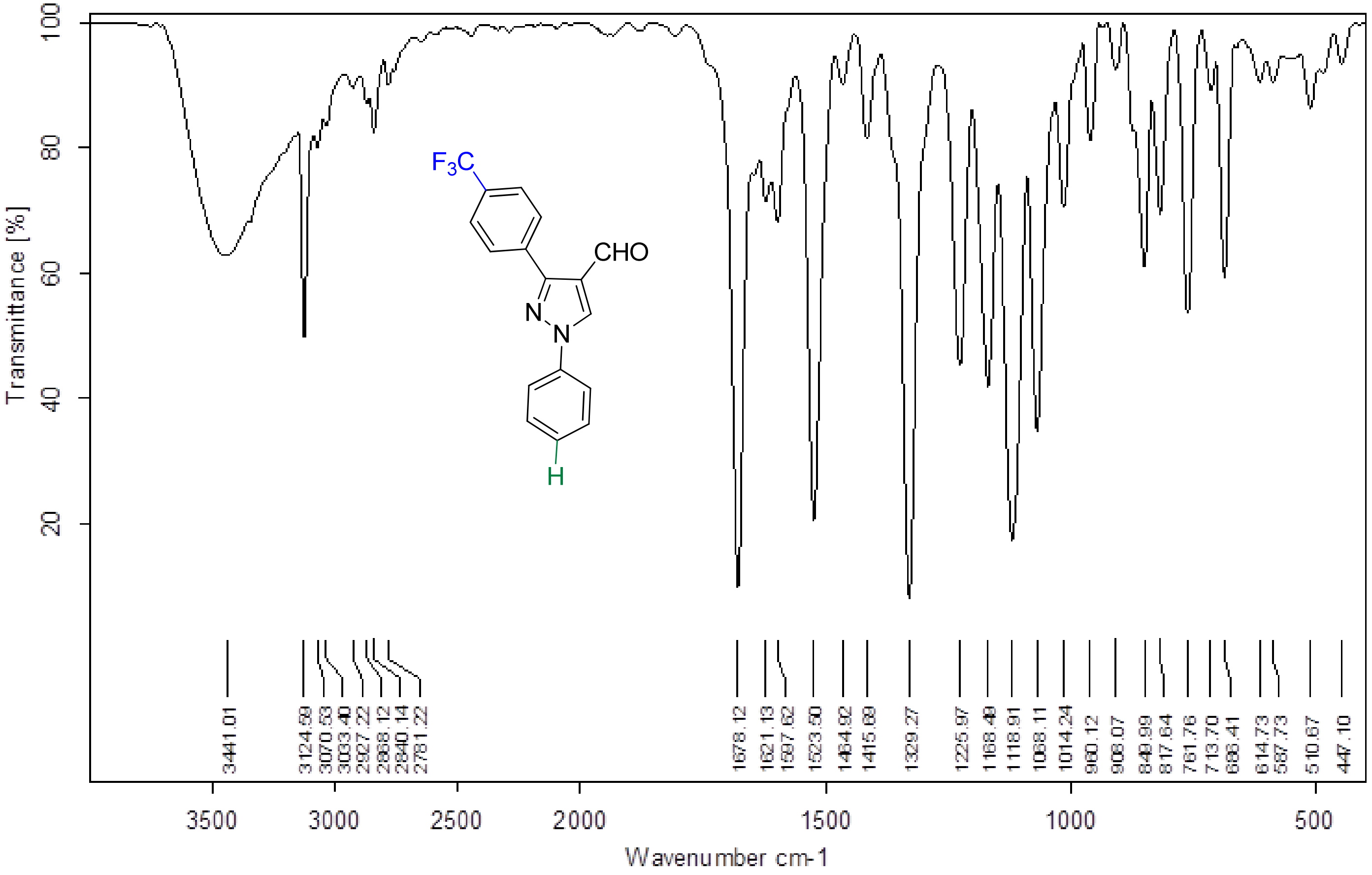

IR (KBr) spectrum of 1-phenyl-3-(4-(trifluoromethyl)phenyl)-1*H*-pyrazole-4-carbaldehyde **2d**

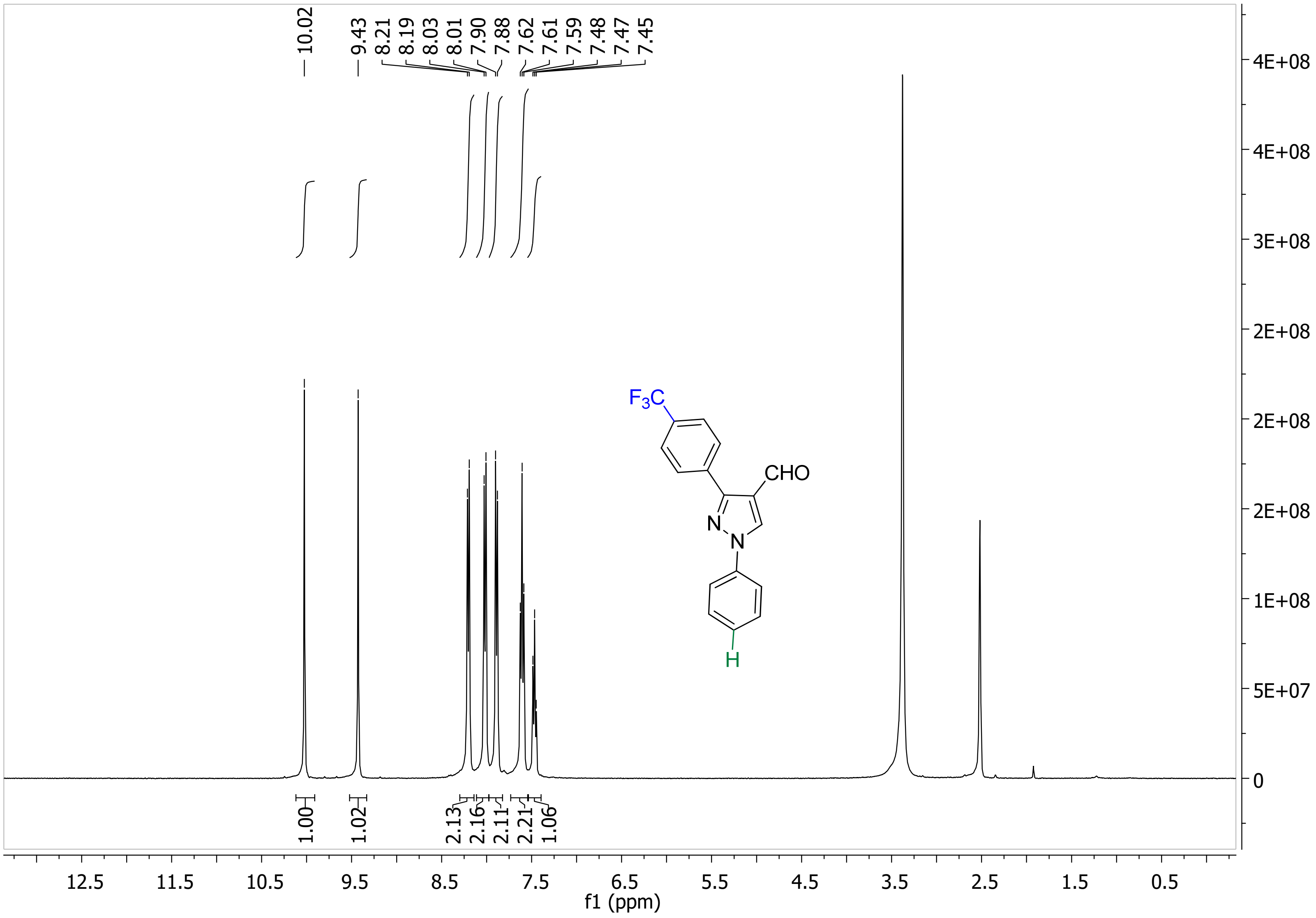

^{1}H NMR (DMSO-*d6*, 400 MHz) spectrum of 1-phenyl-3-(4-(trifluoromethyl)phenyl)-1*H*-pyrazole-4-carbaldehyde **2d**

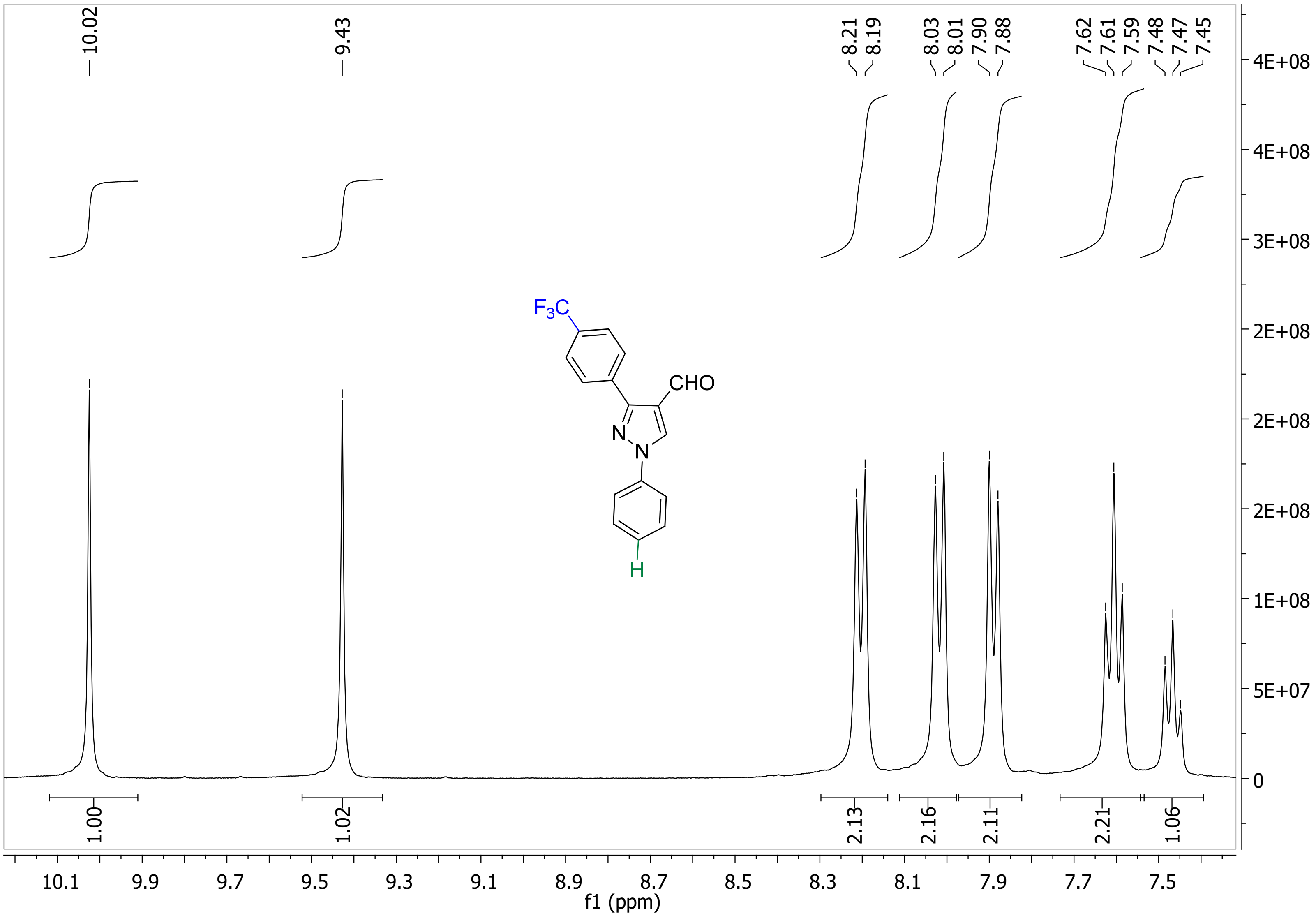

^{1}H NMR (DMSO-*d6*, 400 MHz) spectrum of 1-phenyl-3-(4-(trifluoromethyl)phenyl)-1*H*-pyrazole-4-carbaldehyde **2d**

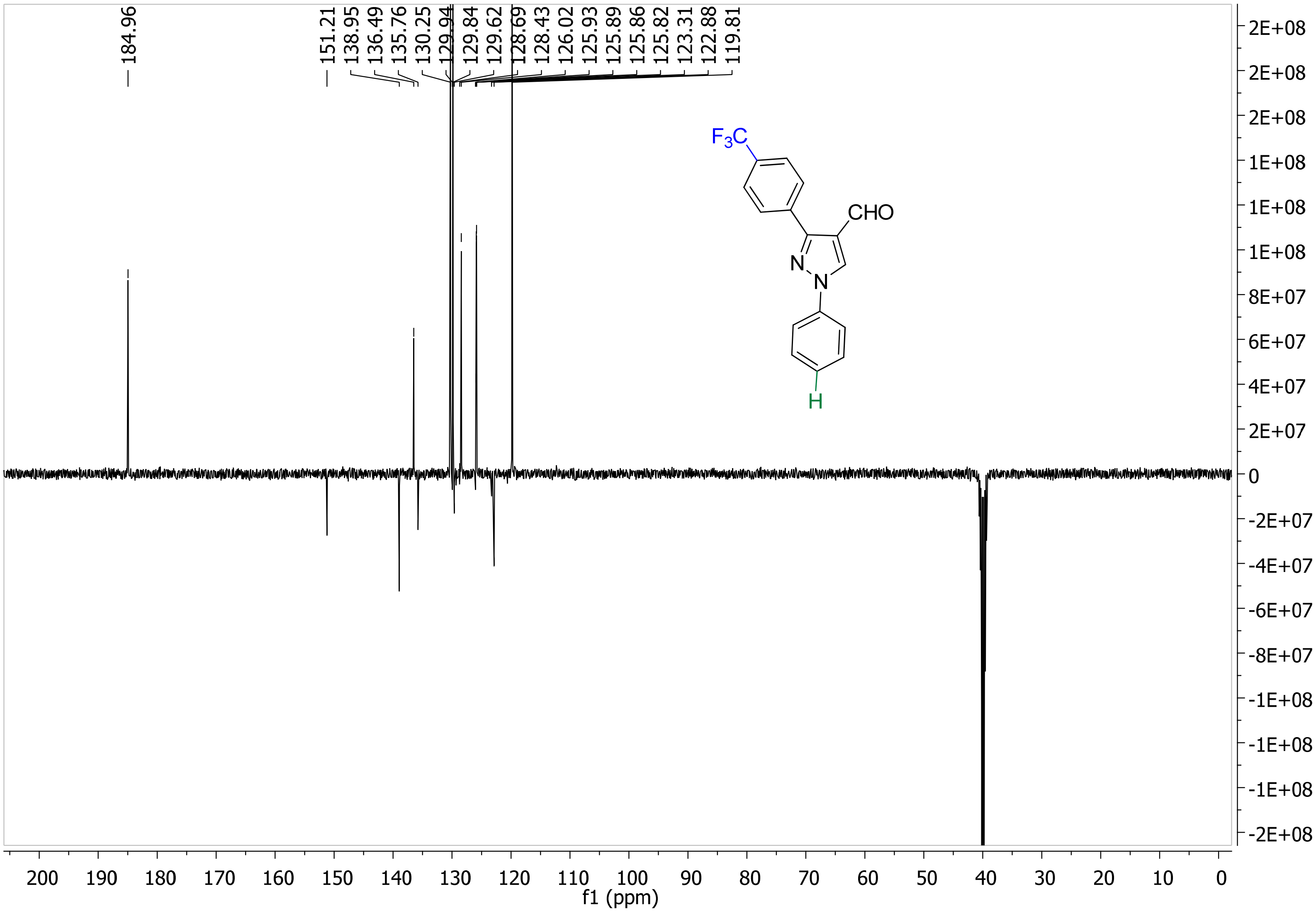

^{13}C APT NMR (DMSO-*d6*, 101 MHz) spectrum of 1-phenyl-3-(4-(trifluoromethyl)phenyl)-1*H*-pyrazole-4-carbaldehyde **2d**

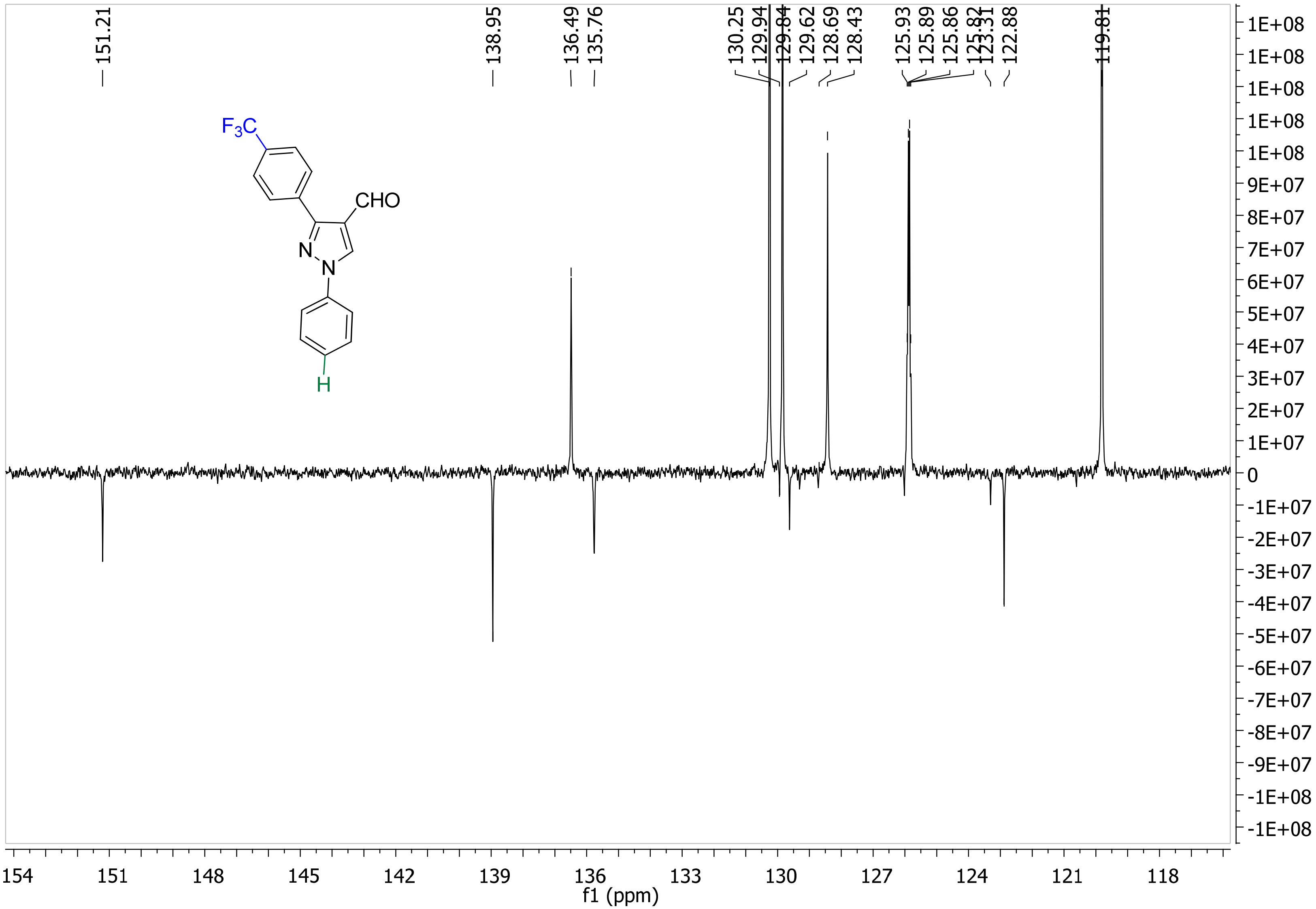

^{13}C APT NMR (DMSO-*d6*, 101 MHz) spectrum of 1-phenyl-3-(4-(trifluoromethyl)phenyl)-1*H*-pyrazole-4-carbaldehyde **2d**

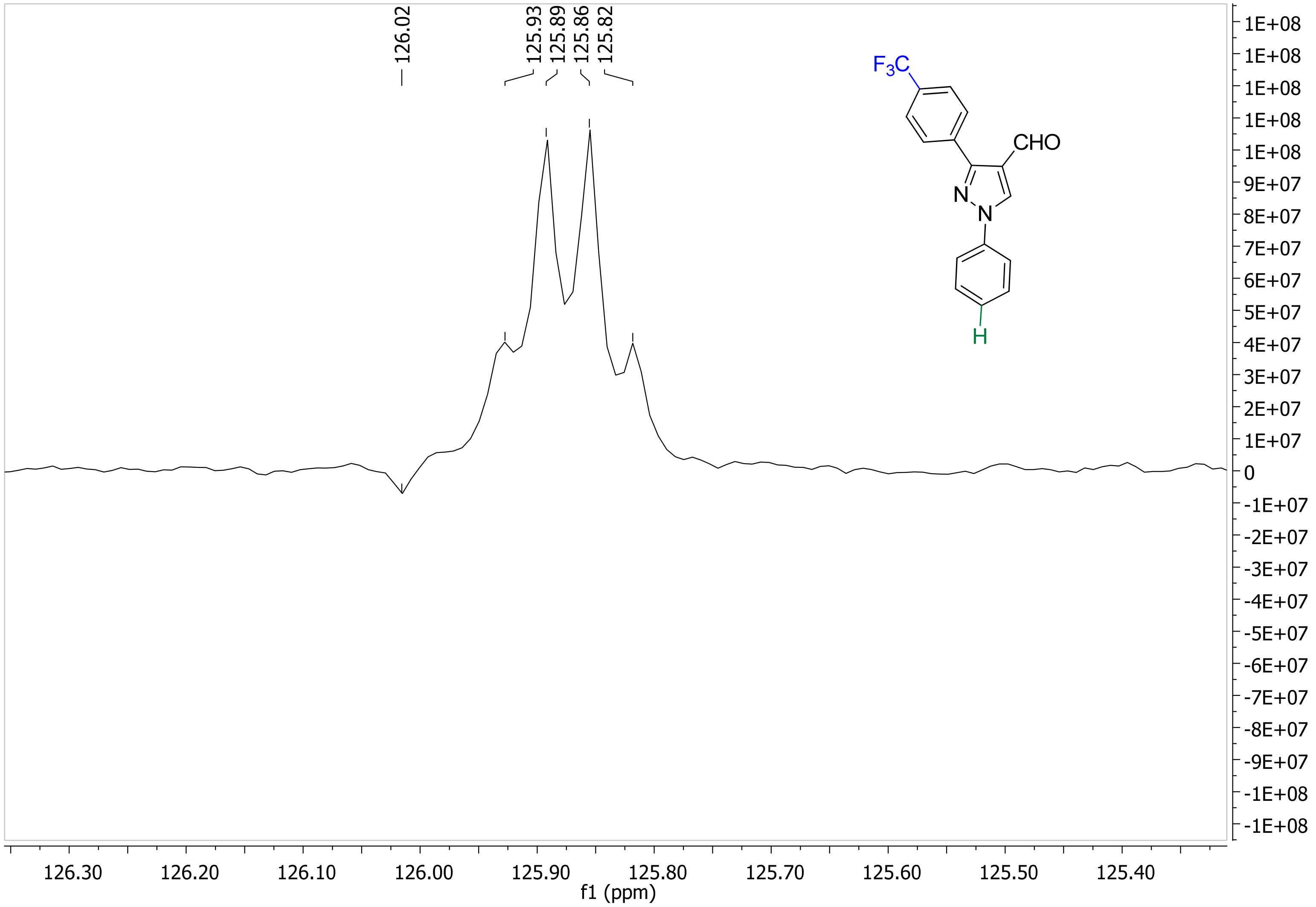

^{13}C APT NMR (DMSO-*d6*, 101 MHz) spectrum of 1-phenyl-3-(4-(trifluoromethyl)phenyl)-1*H*-pyrazole-4-carbaldehyde **2d**

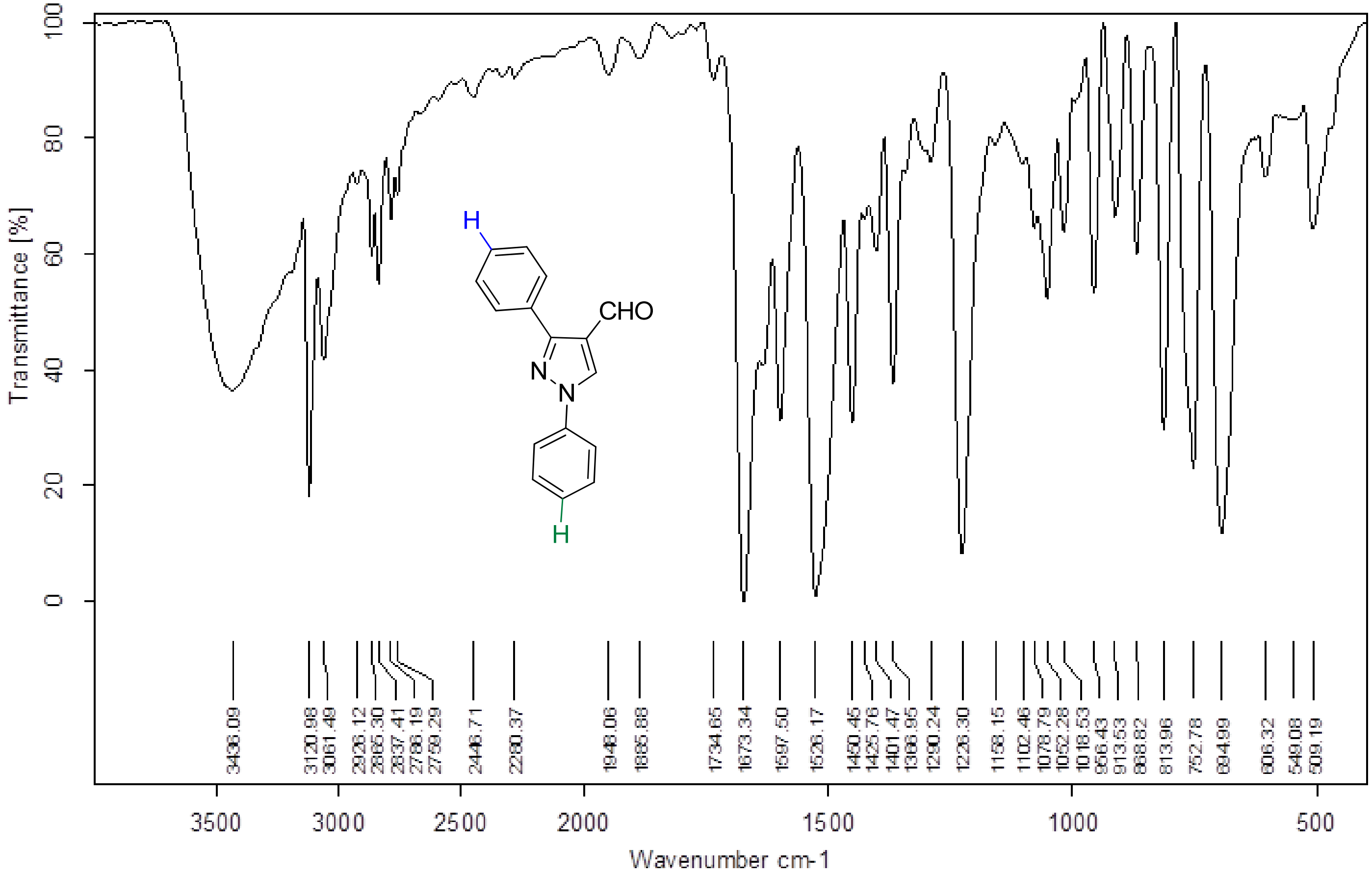

IR (KBr) spectrum of 1,3-diphenyl-1*H*-pyrazole-4-carbaldehyde **2e**

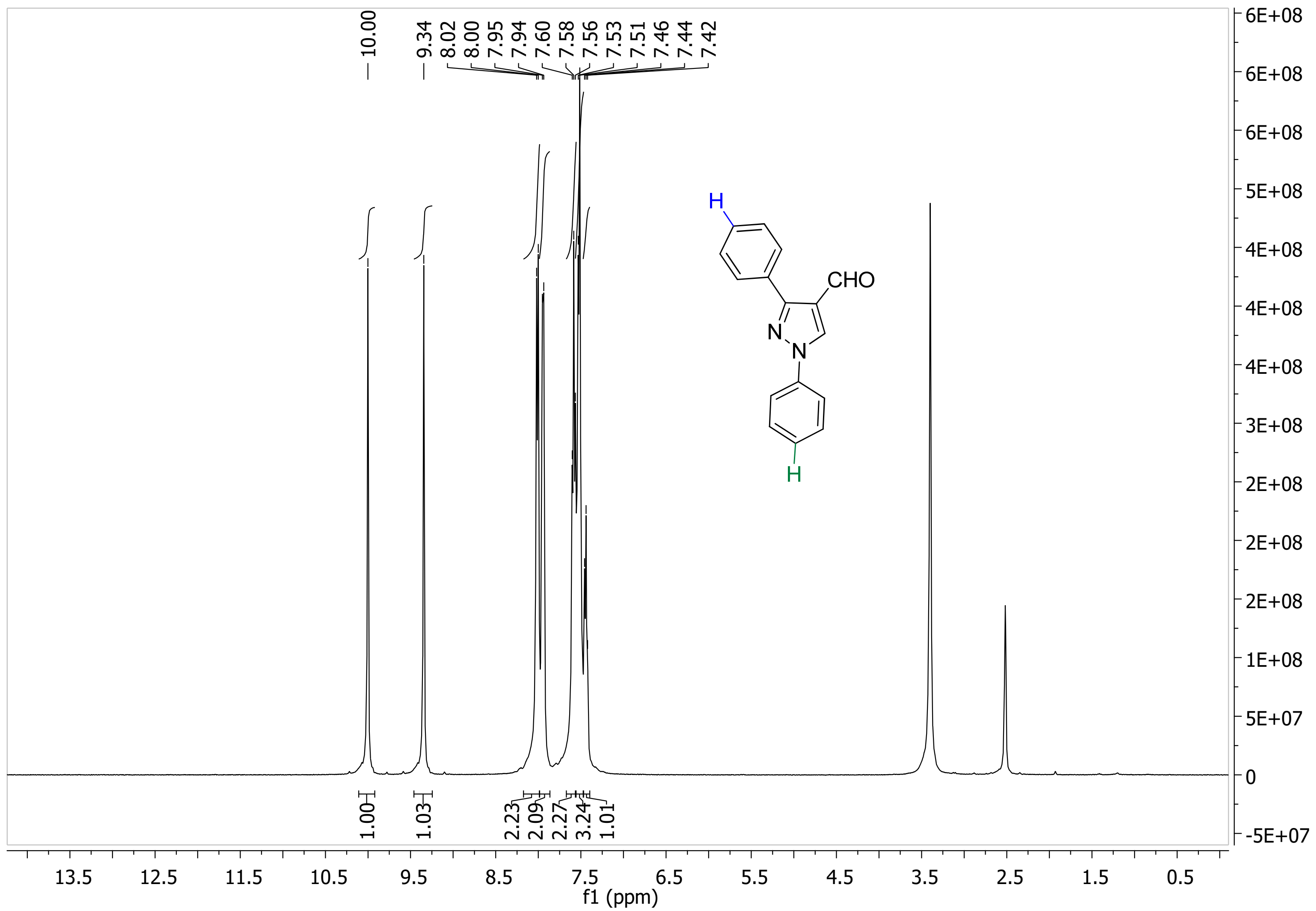

^{1}H NMR (DMSO-*d6*, 400 MHz) spectrum of 1,3-diphenyl-1*H*-pyrazole-4-carbaldehyde **2e**

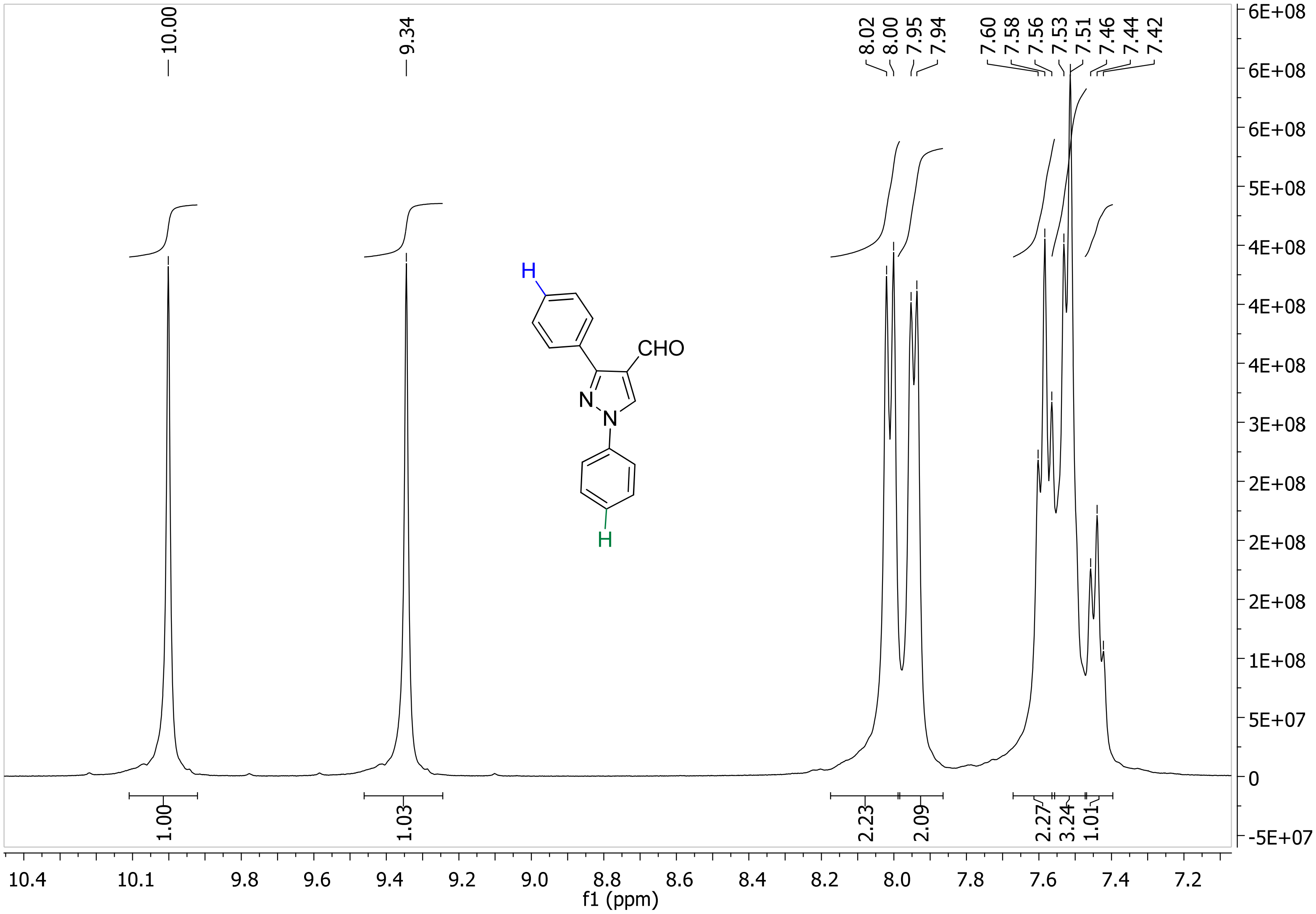

^{1}H NMR (DMSO-*d6*, 400 MHz) spectrum of 1,3-diphenyl-1*H*-pyrazole-4-carbaldehyde **2e**

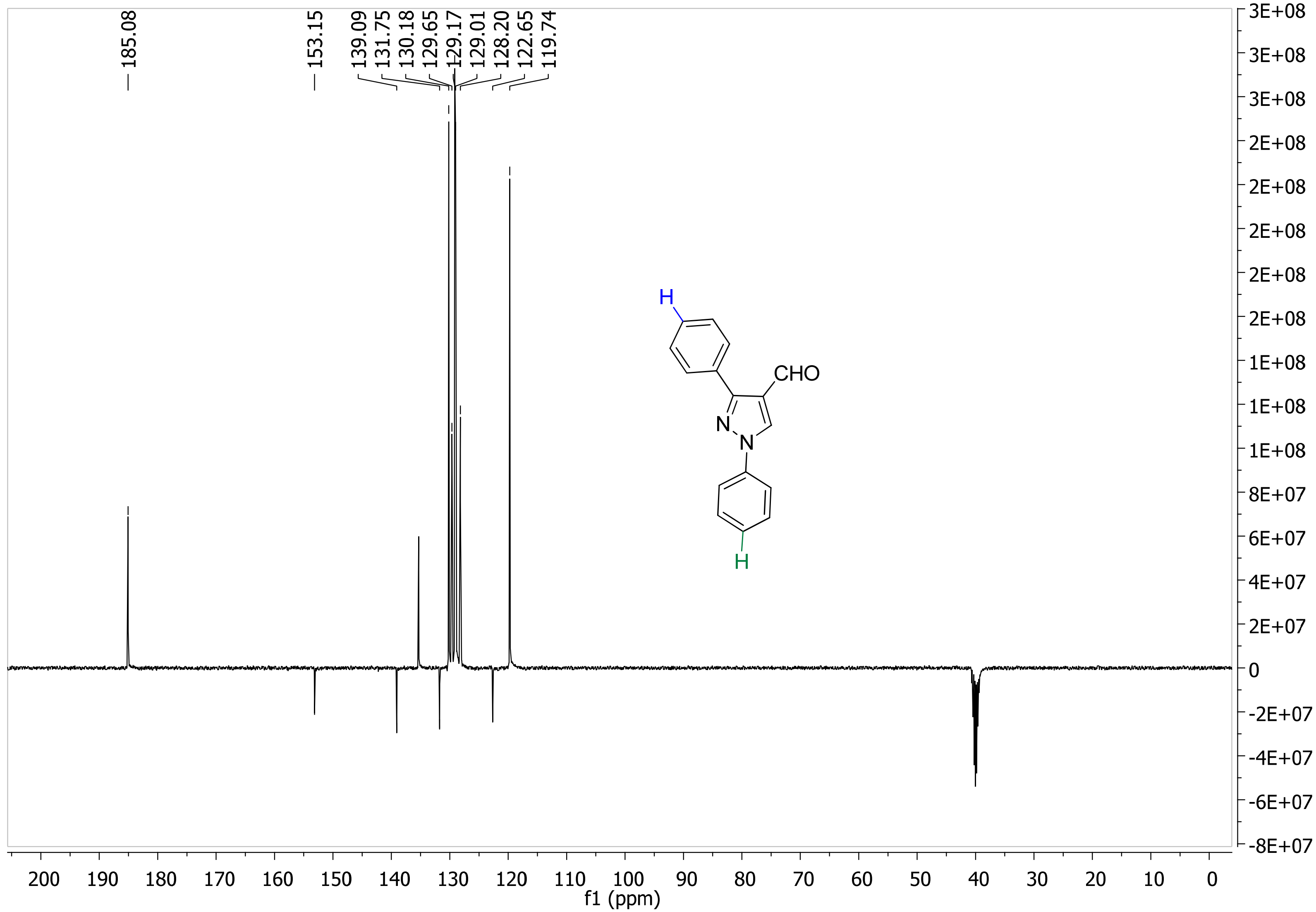

^{13}C APT NMR (DMSO-*d6*, 101 MHz) spectrum of 1,3-diphenyl-1*H*-pyrazole-4-carbaldehyde **2e**

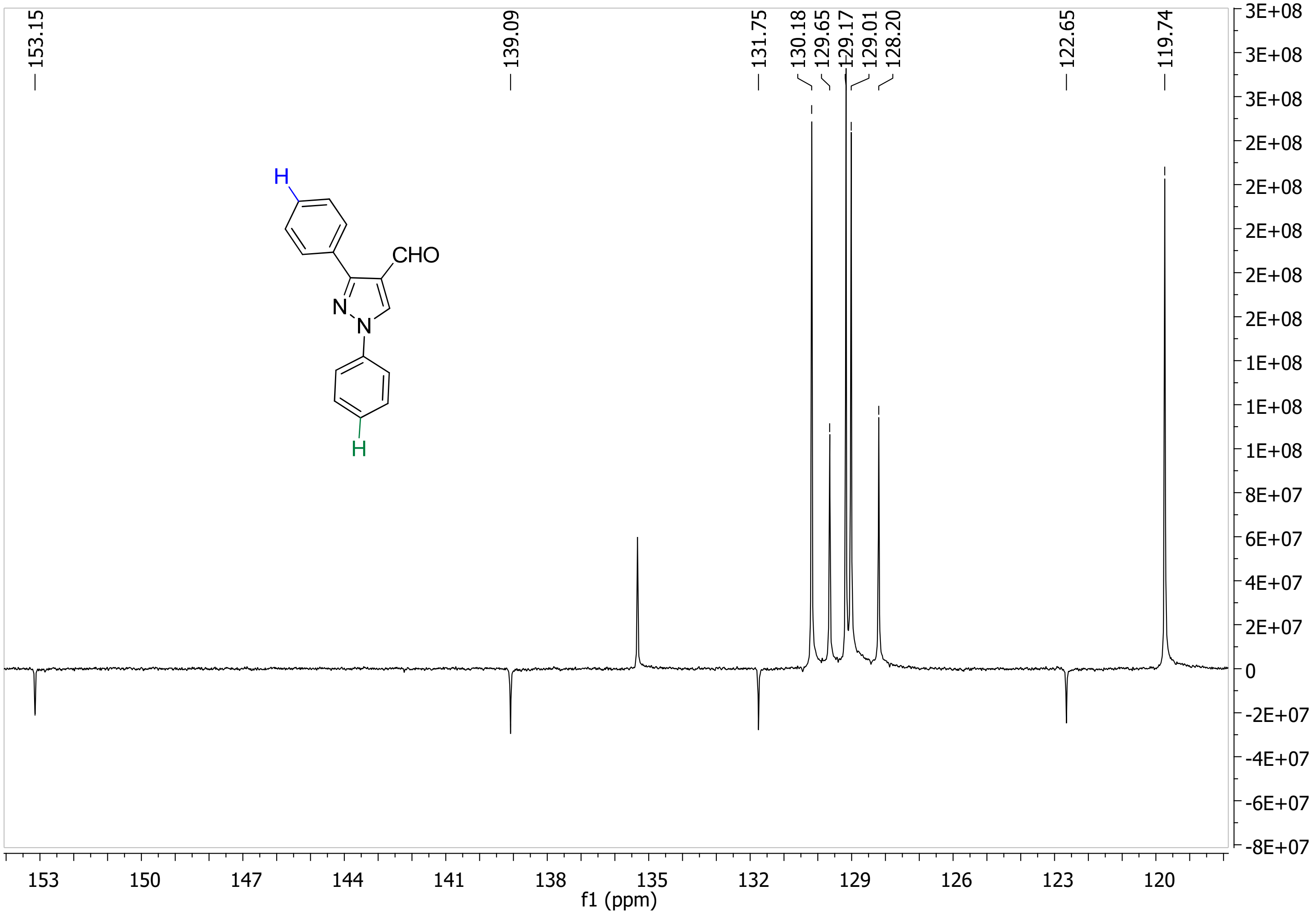

^{13}C APT NMR (DMSO-*d6*, 101 MHz) spectrum of 1,3-diphenyl-1*H*-pyrazole-4-carbaldehyde **2e**

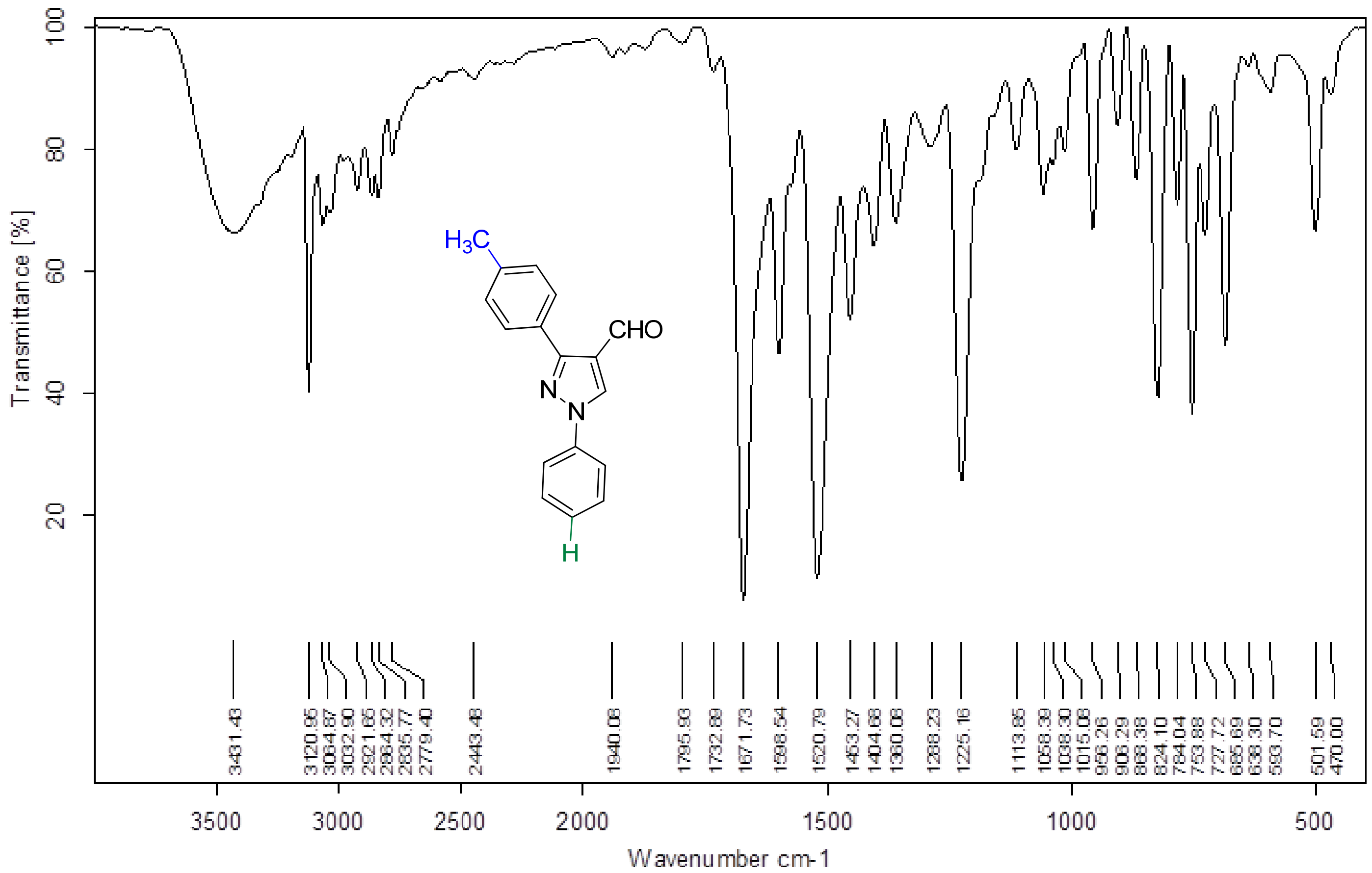

IR (KBr) spectrum of 1-phenyl-3-(*p*-tolyl)-1*H*-pyrazole-4-carbaldehyde **2f**

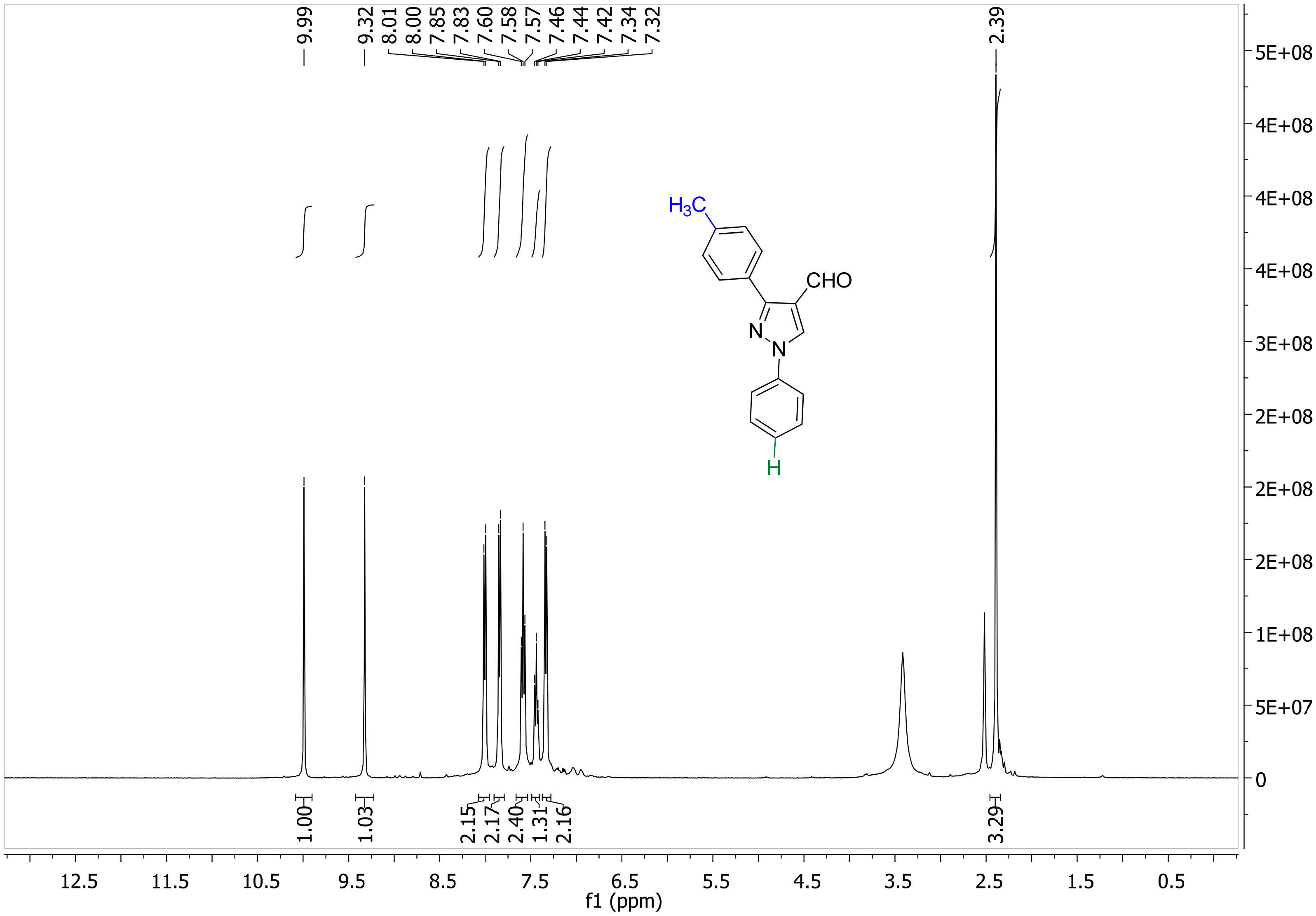

^{1}H NMR (DMSO-*d6*, 400 MHz) spectrum of 1-phenyl-3-(*p*-tolyl)-1*H*-pyrazole-4-carbaldehyde **2f**

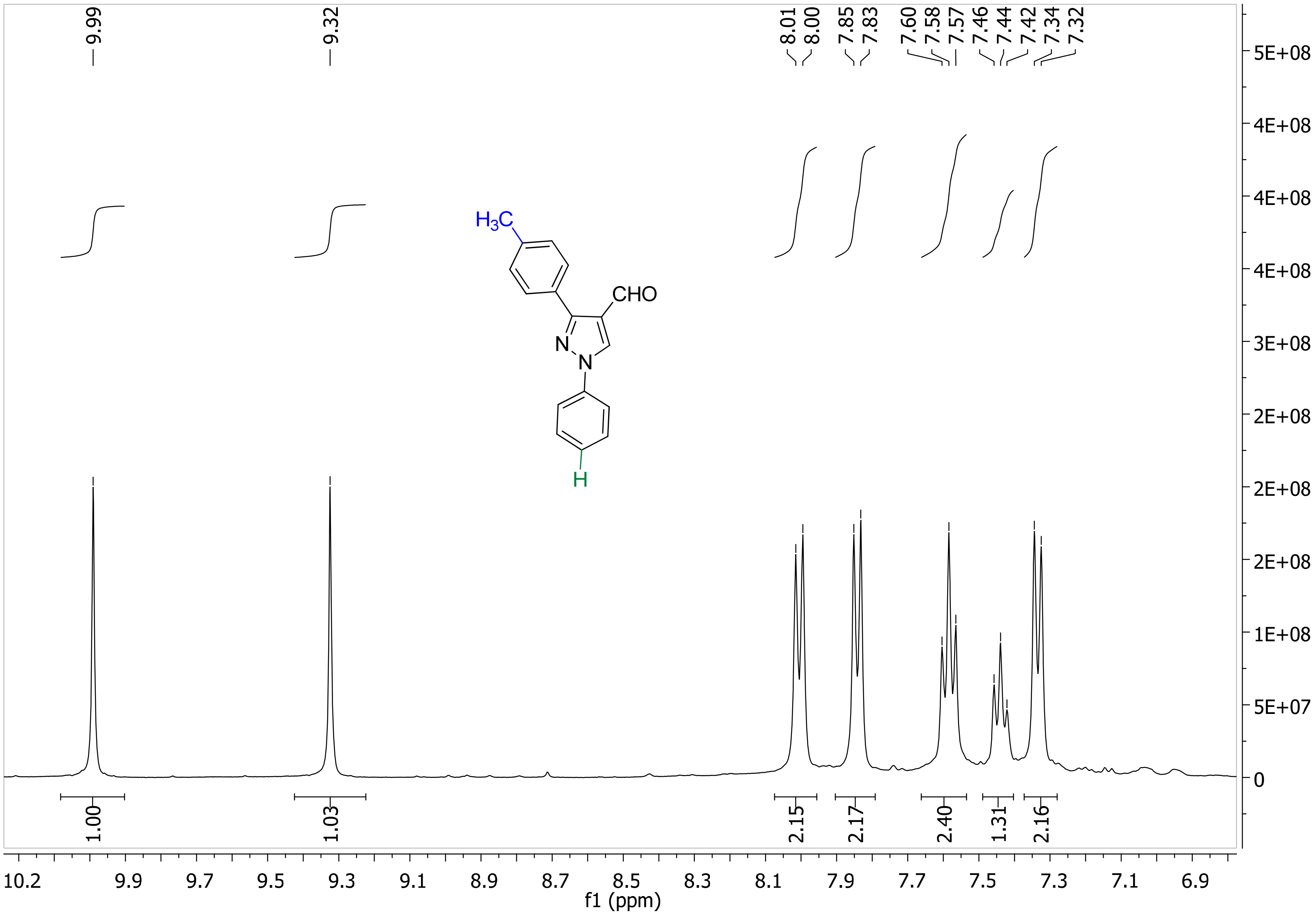

^{1}H NMR (DMSO-*d6*, 400 MHz) spectrum of 1-phenyl-3-(*p*-tolyl)-1*H*-pyrazole-4-carbaldehyde **2f**

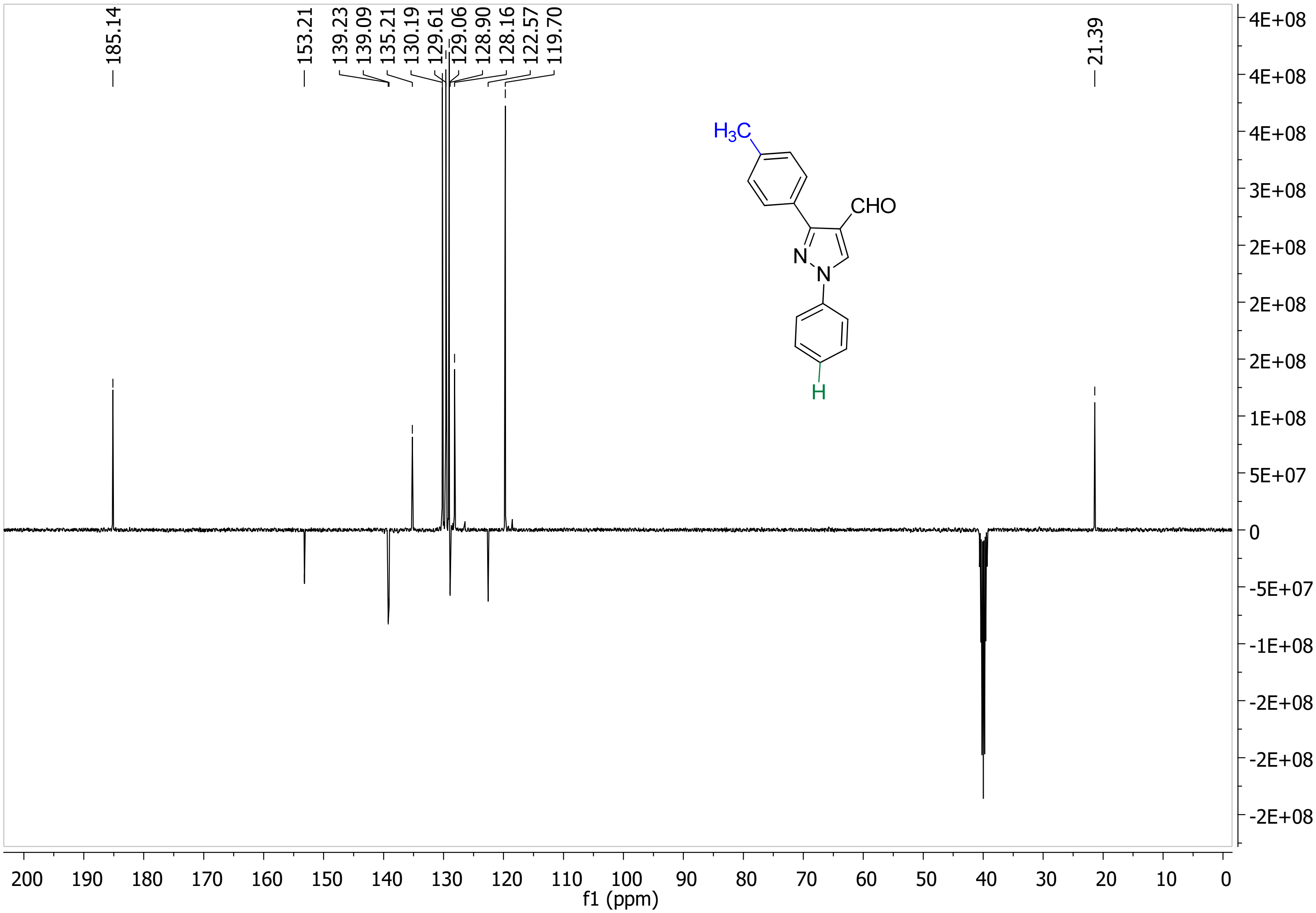

^{13}C APT NMR (DMSO-*d6*, 101 MHz) spectrum of 1-phenyl-3-(*p*-tolyl)-1*H*-pyrazole-4-carbaldehyde **2f**

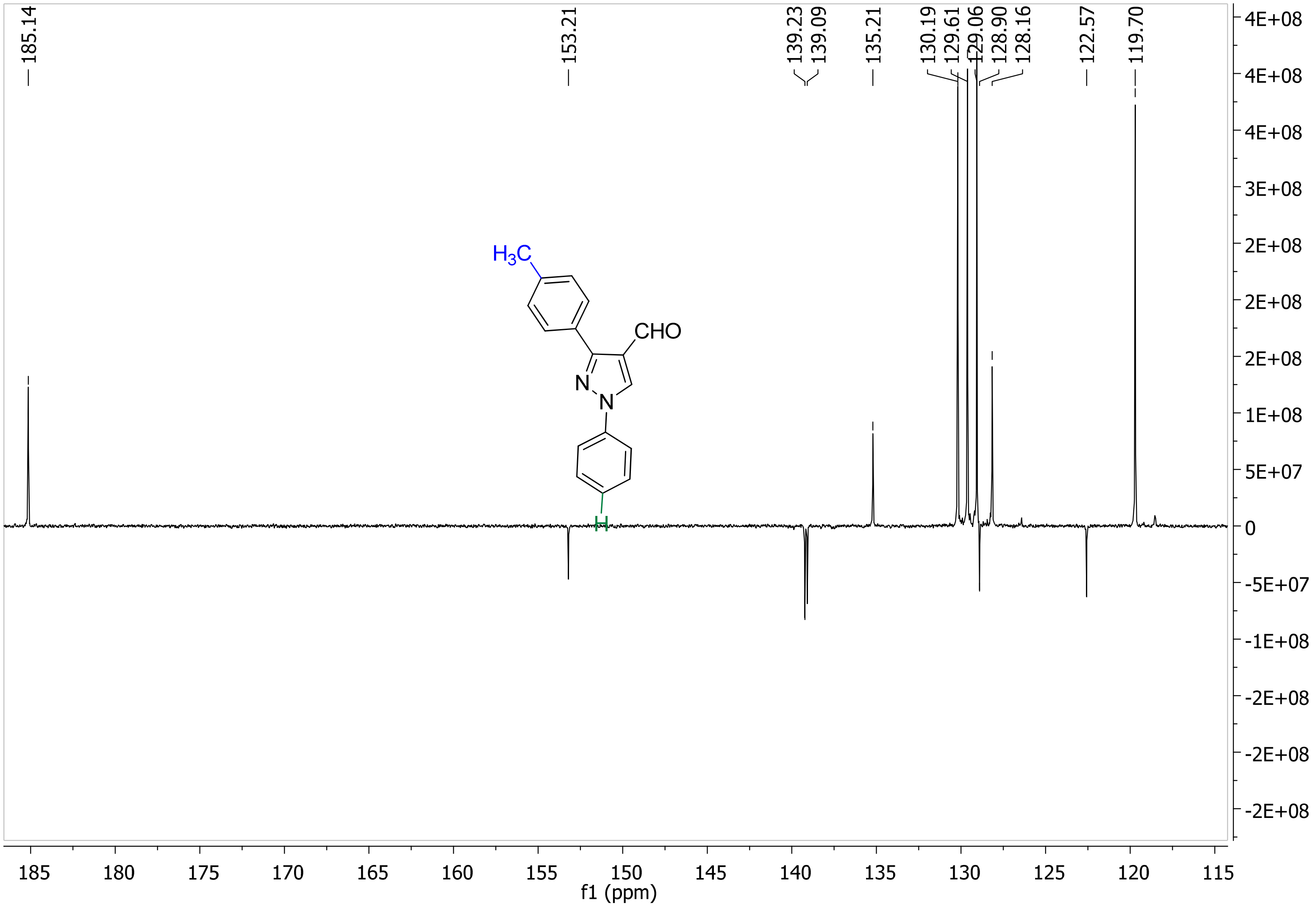

^{13}C APT NMR (DMSO-*d6*, 101 MHz) spectrum of 1-phenyl-3-(*p*-tolyl)-1*H*-pyrazole-4-carbaldehyde **2f**

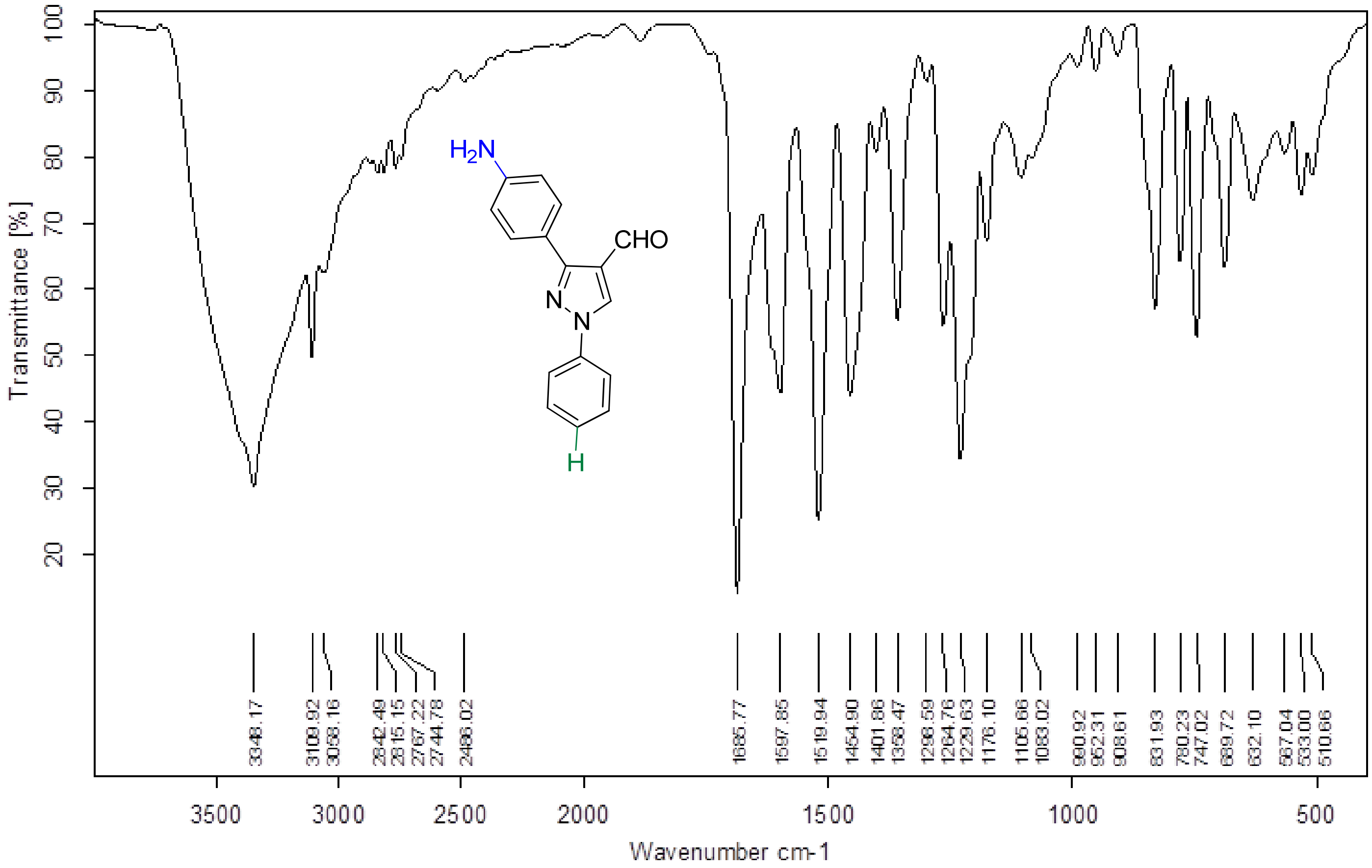

IR (KBr) spectrum of 3-(4-aminophenyl)-1-phenyl-1*H*-pyrazole-4-carbaldehyde **2g**

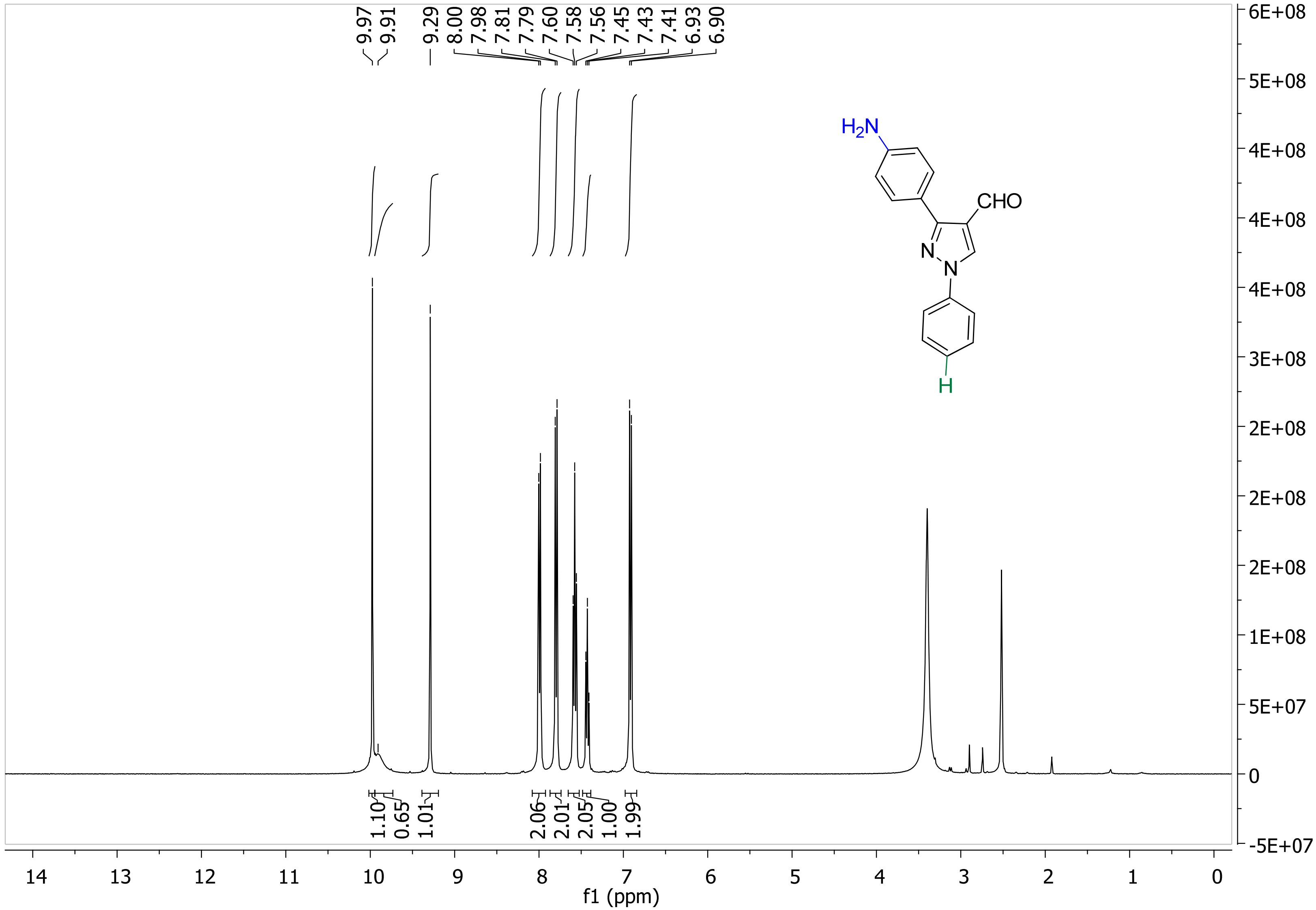

^{1}H NMR (DMSO-*d6*, 400 MHz) spectrum of 3-(4-aminophenyl)-1-phenyl-1*H*-pyrazole-4-carbaldehyde **2g**

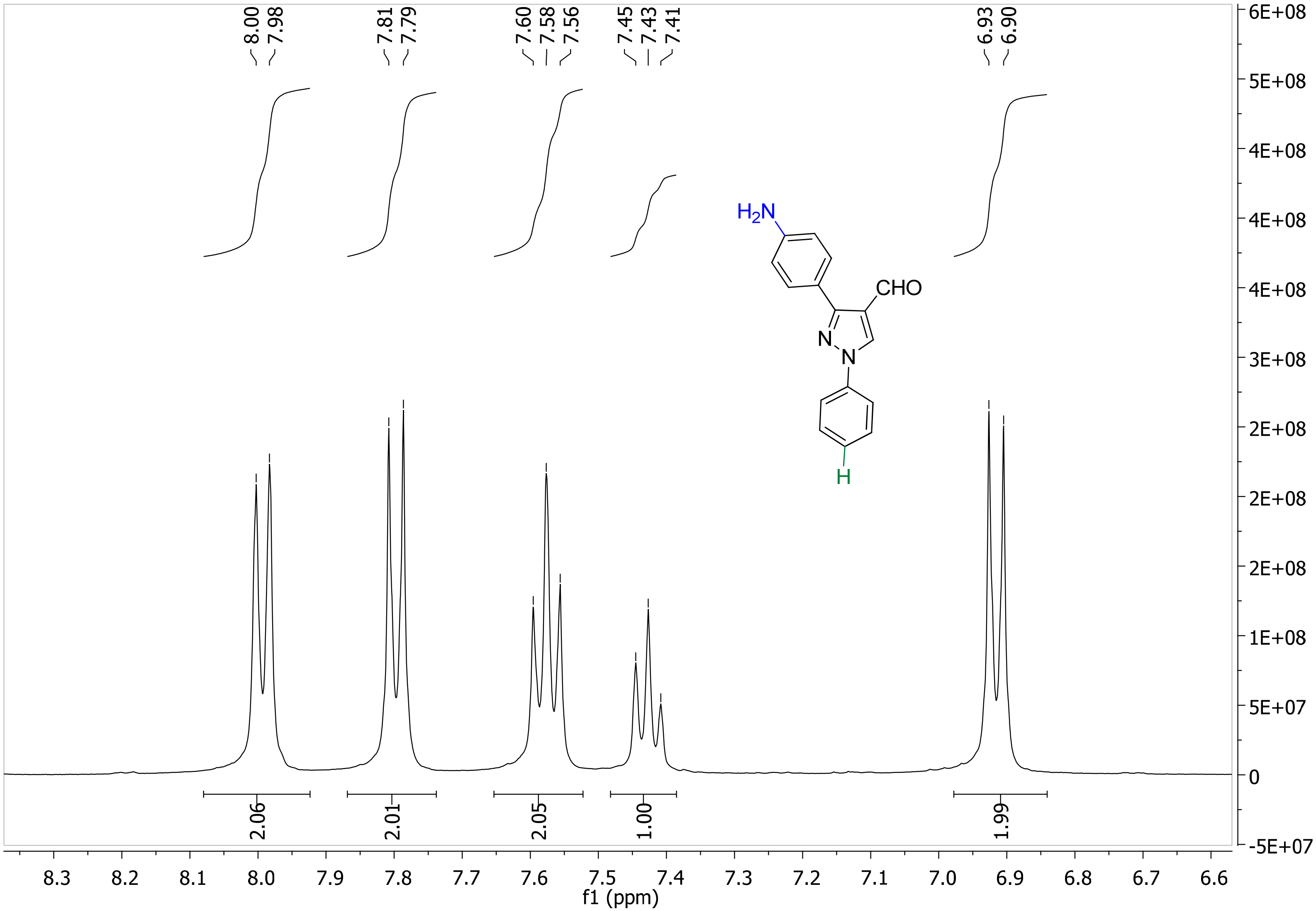

^{1}H NMR (DMSO-*d6*, 400 MHz) spectrum of 3-(4-aminophenyl)-1-phenyl-1*H*-pyrazole-4-carbaldehyde **2g**

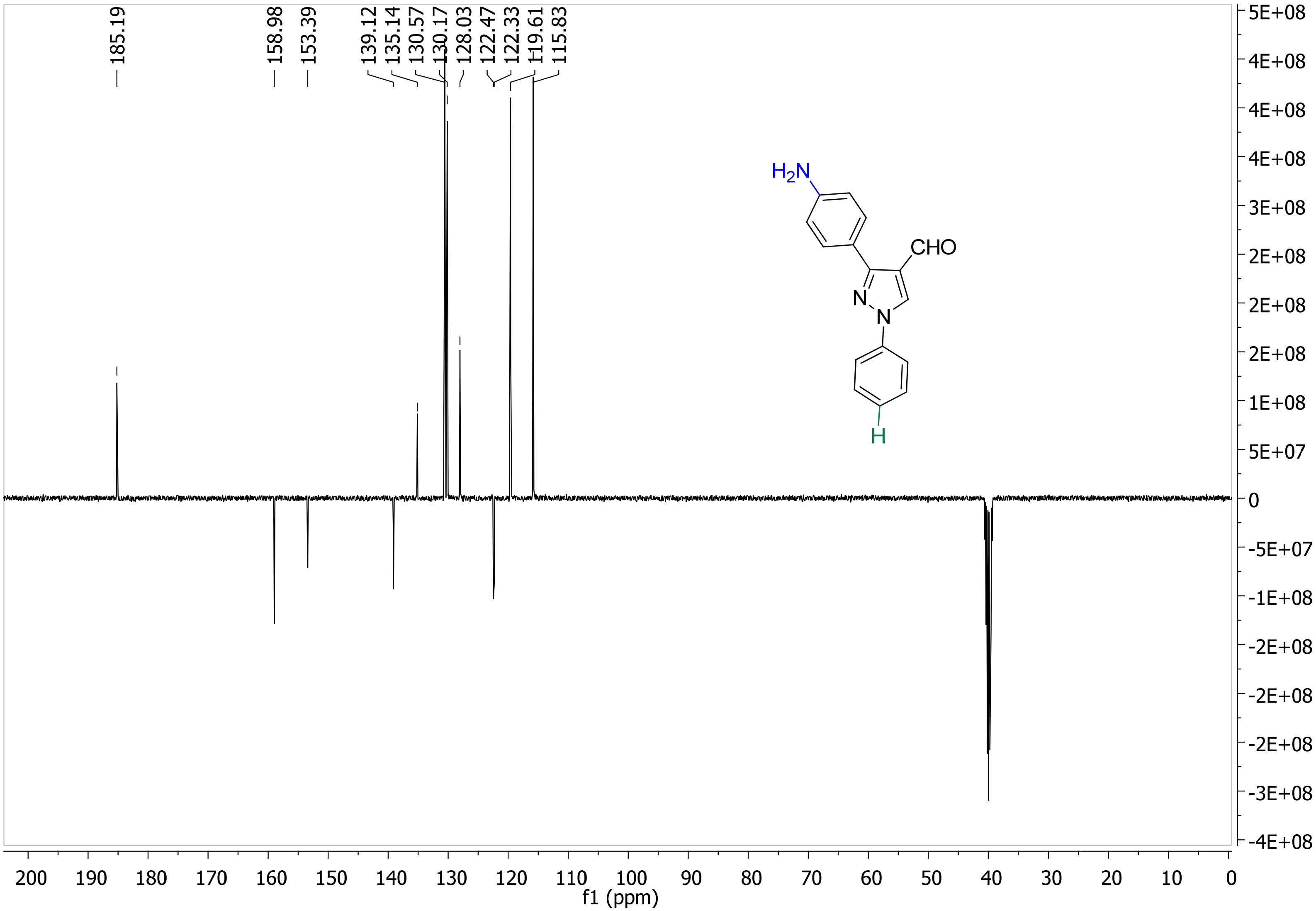

^{13}C APT NMR (DMSO-*d6*, 101 MHz) spectrum of 3-(4-aminophenyl)-1-phenyl-1*H*-pyrazole-4-carbaldehyde **2g**

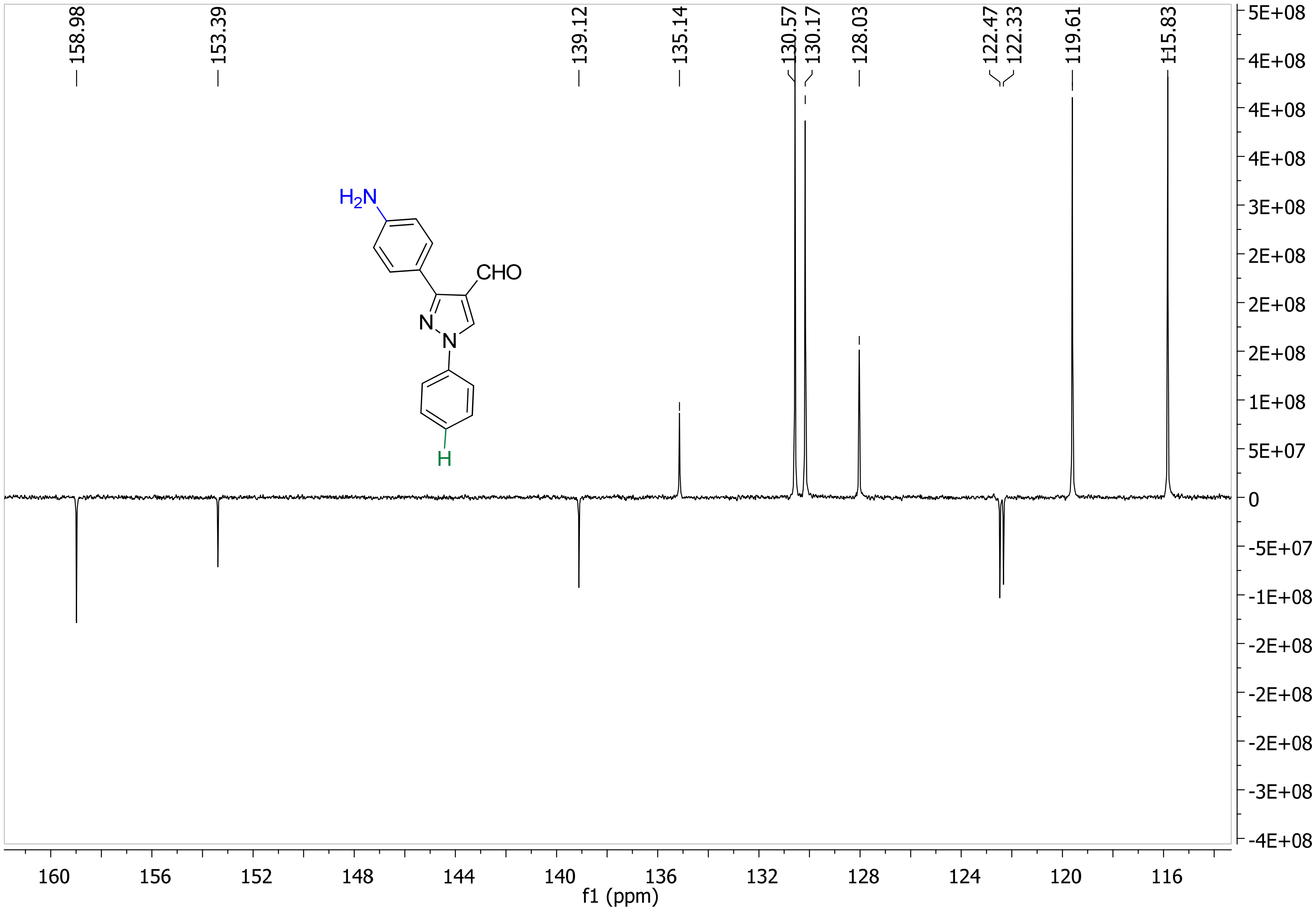

^{13}C APT NMR (DMSO-*d6*, 101 MHz) spectrum of 3-(4-aminophenyl)-1-phenyl-1*H*-pyrazole-4-carbaldehyde **2g**

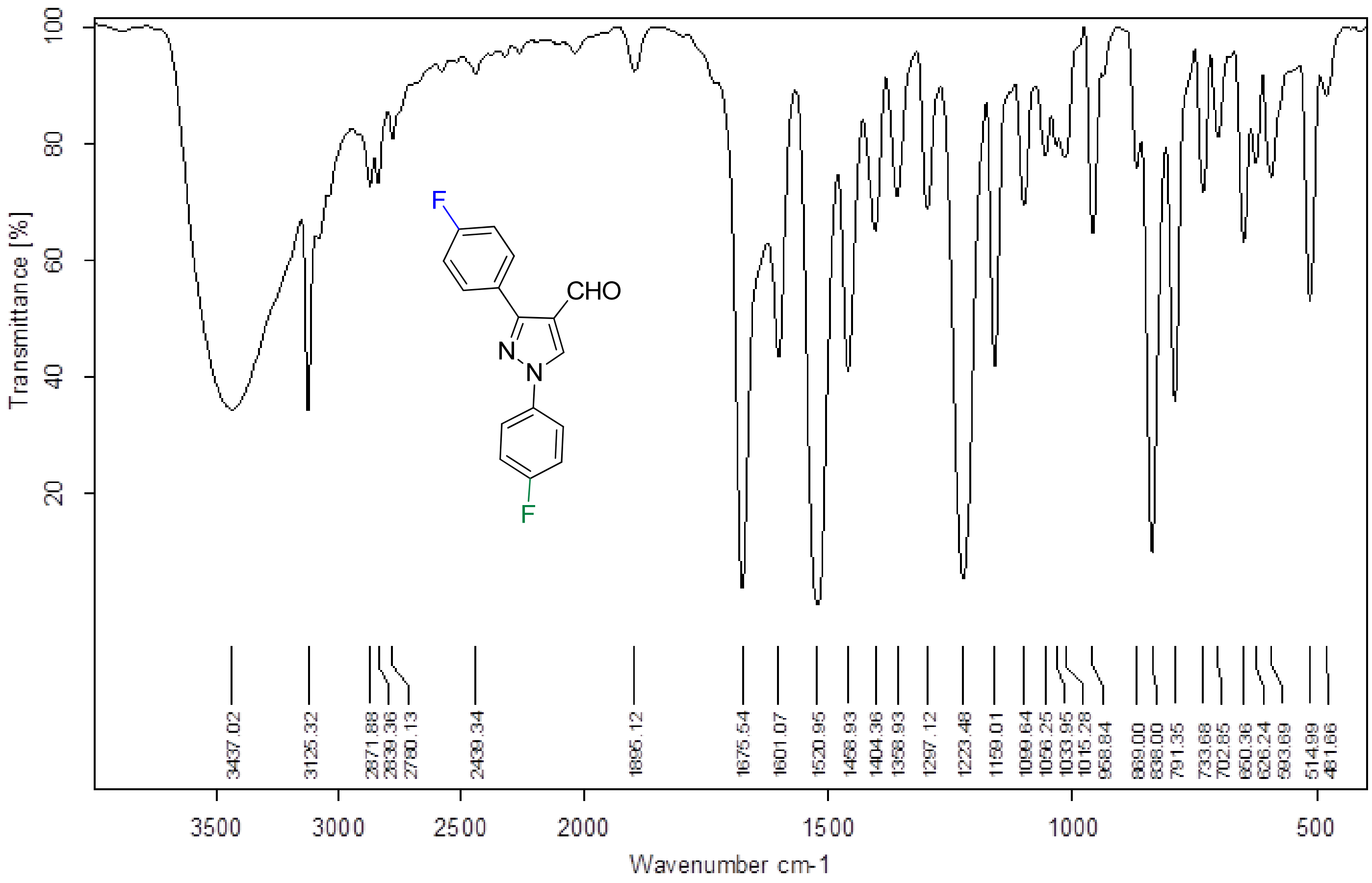

IR (KBr) spectrum of 1,3-bis(4-fluorophenyl)-1*H*-pyrazole-4-carbaldehyde **2h**

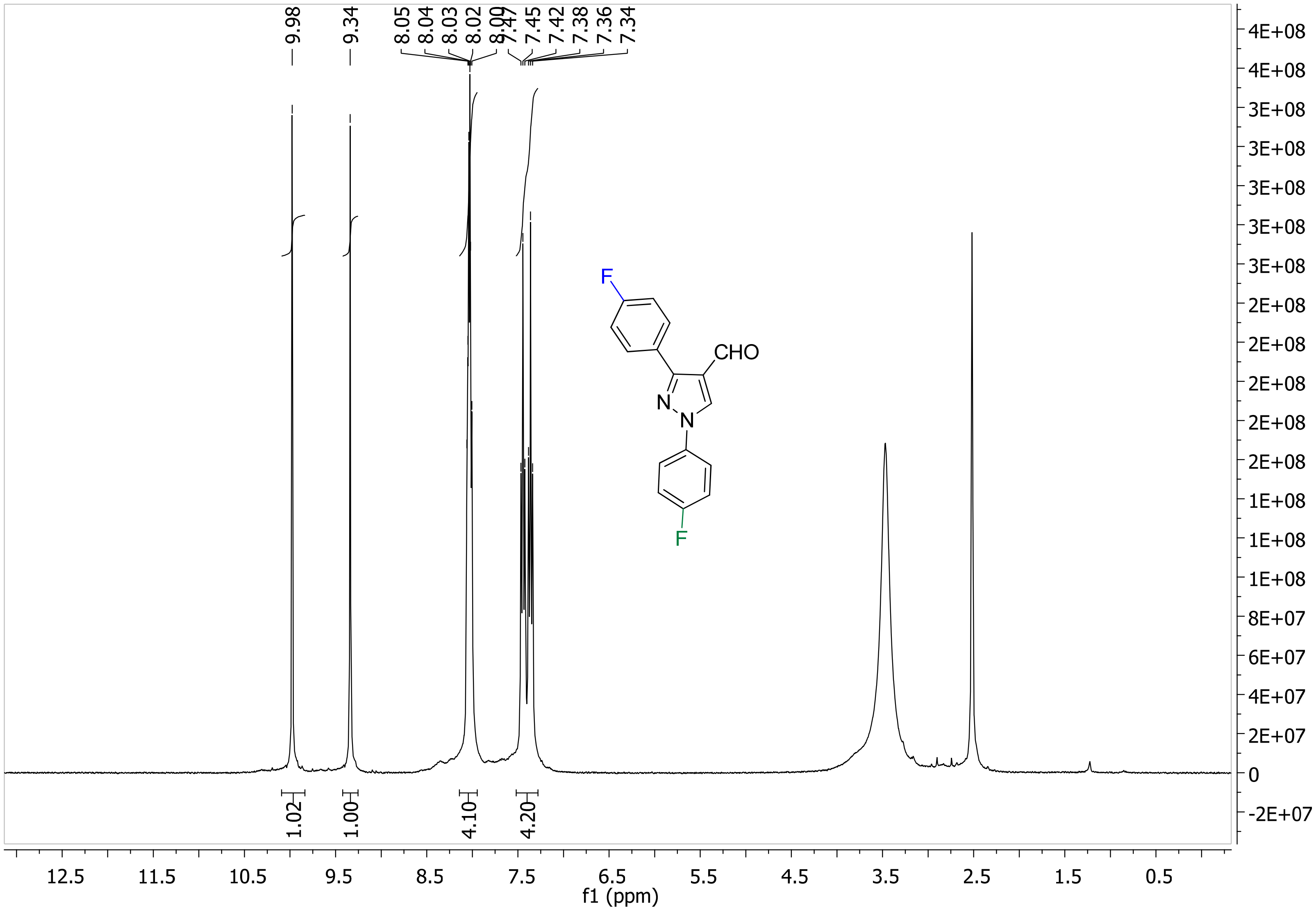

^{1}H NMR (DMSO-*d6*, 400 MHz) spectrum of 1,3-bis(4-fluorophenyl)-1*H*-pyrazole-4-carbaldehyde **2h**

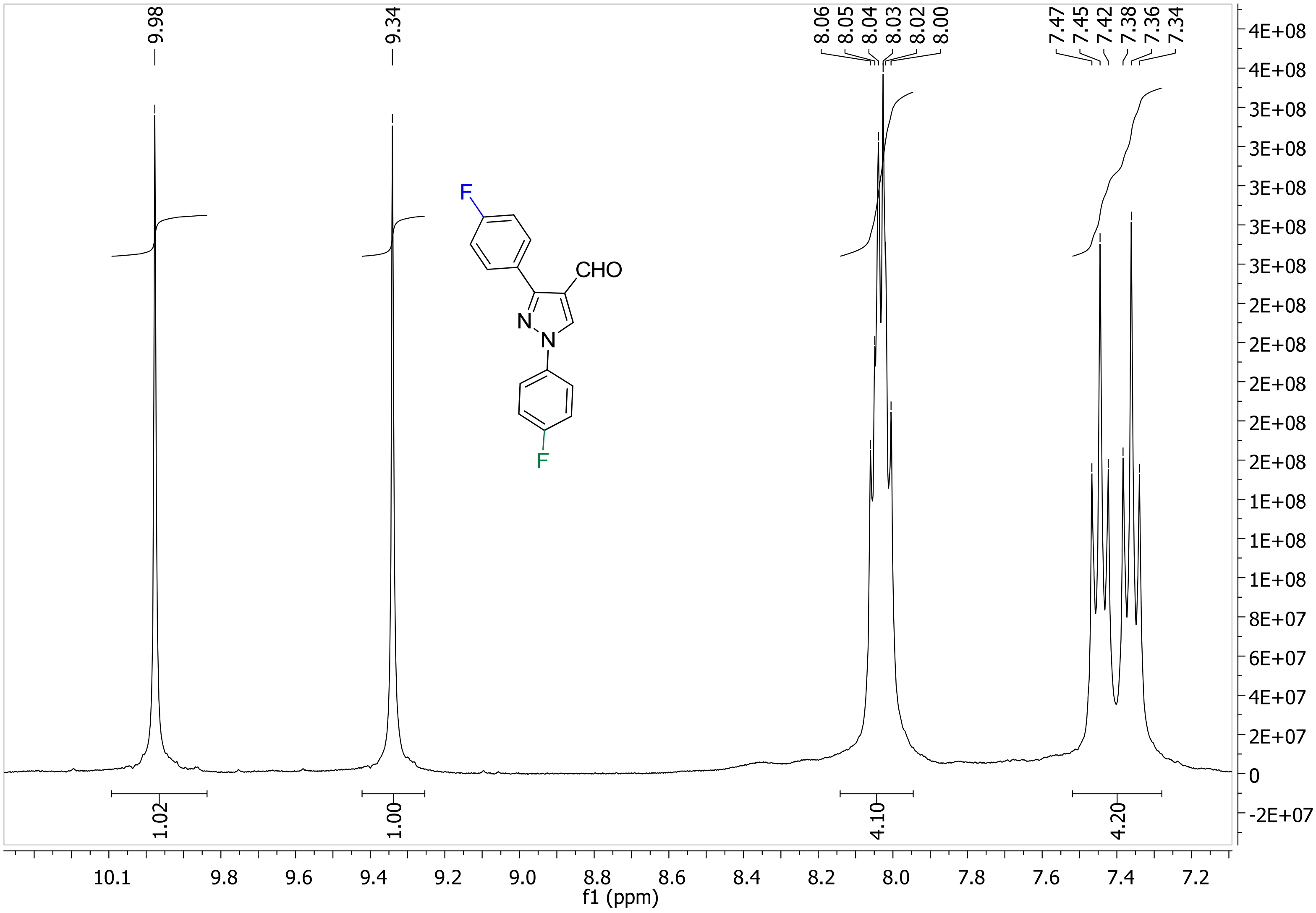

^{1}H NMR (DMSO-*d6*, 400 MHz) spectrum of 1,3-bis(4-fluorophenyl)-1*H*-pyrazole-4-carbaldehyde **2h**

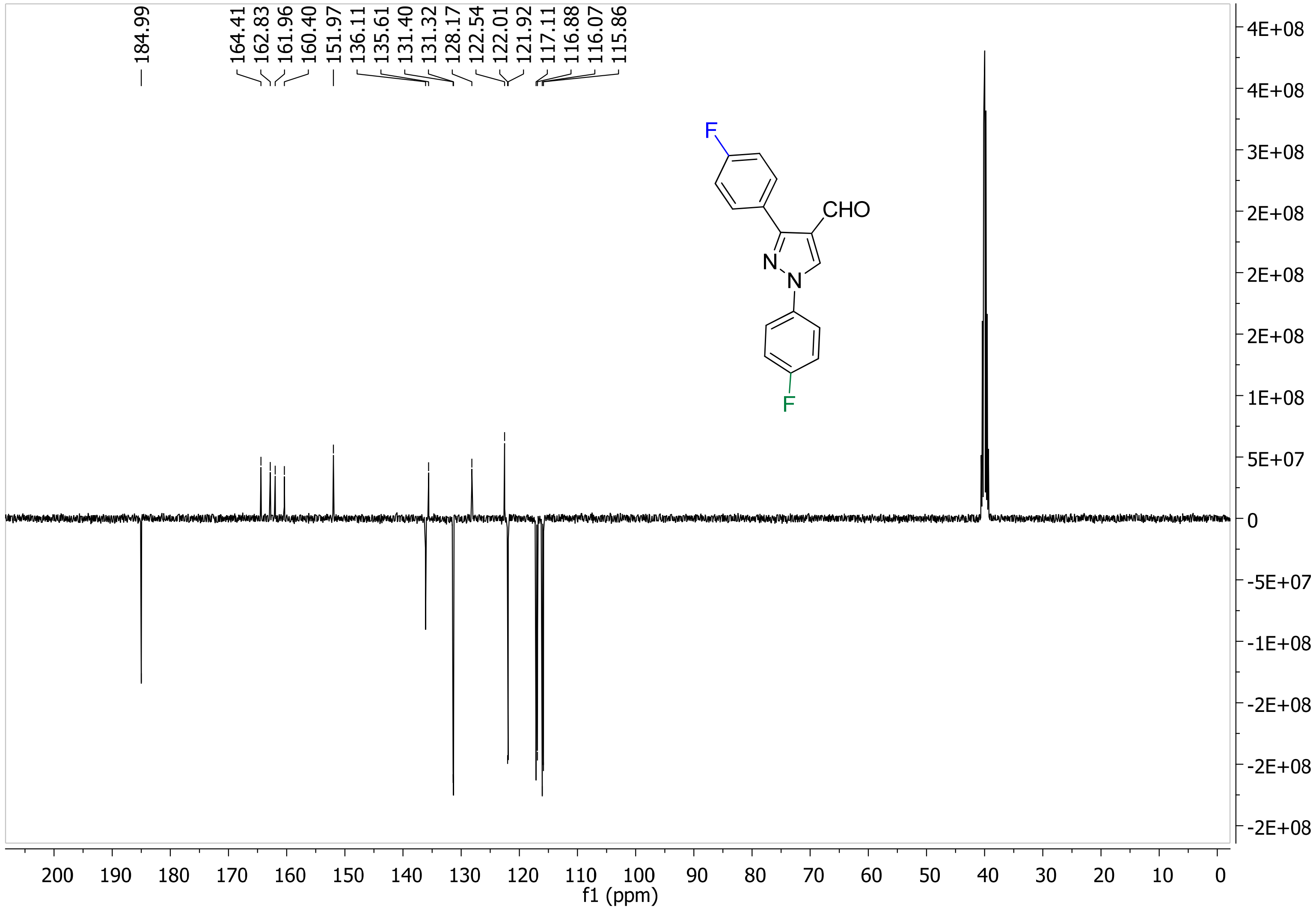

^{13}C APT NMR (DMSO-*d6*, 101 MHz) spectrum of 1,3-bis(4-fluorophenyl)-1*H*-pyrazole-4-carbaldehyde **2h**

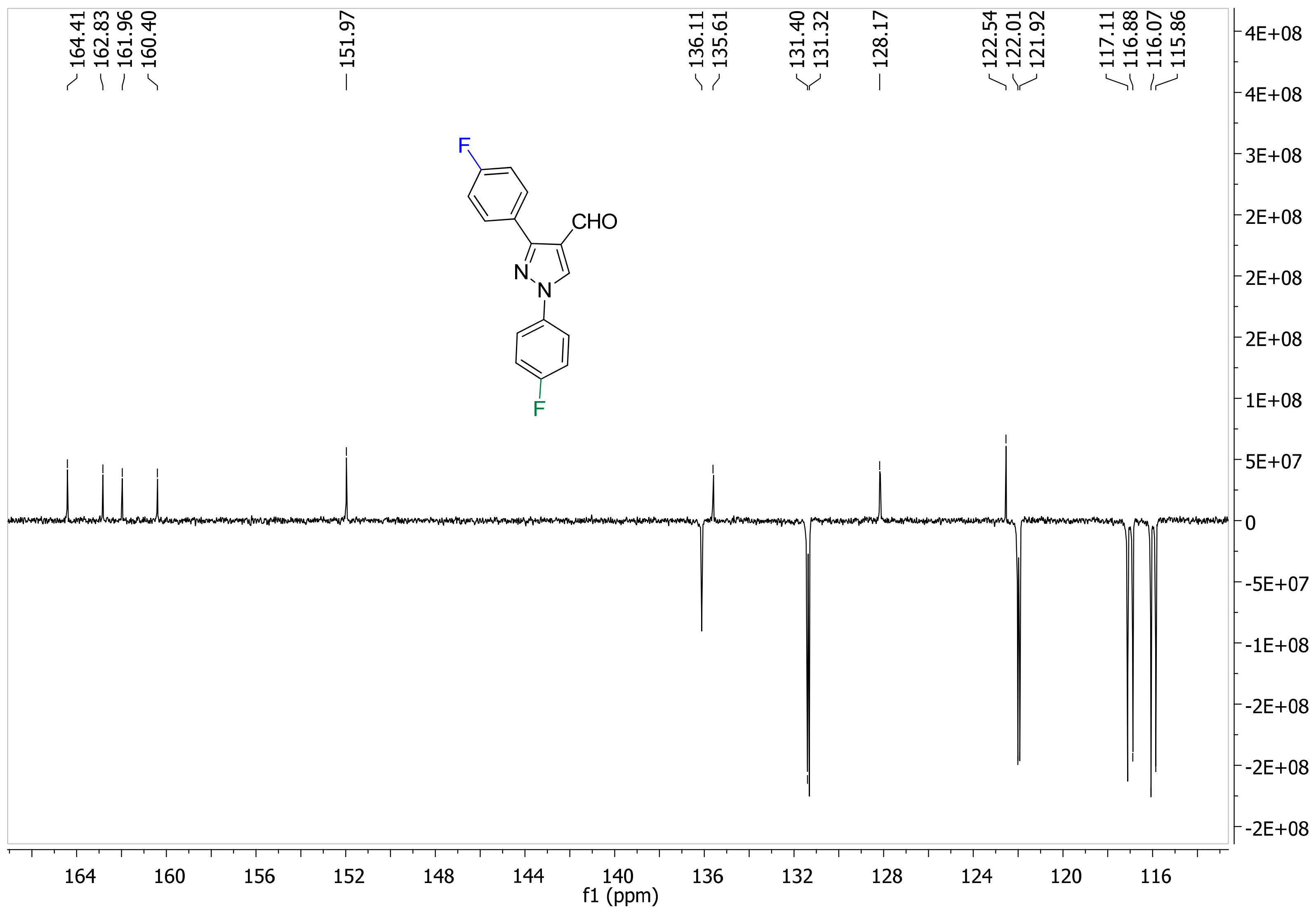

^{13}C APT NMR (DMSO-*d6*, 101 MHz) spectrum of 1,3-bis(4-fluorophenyl)-1*H*-pyrazole-4-carbaldehyde **2h**

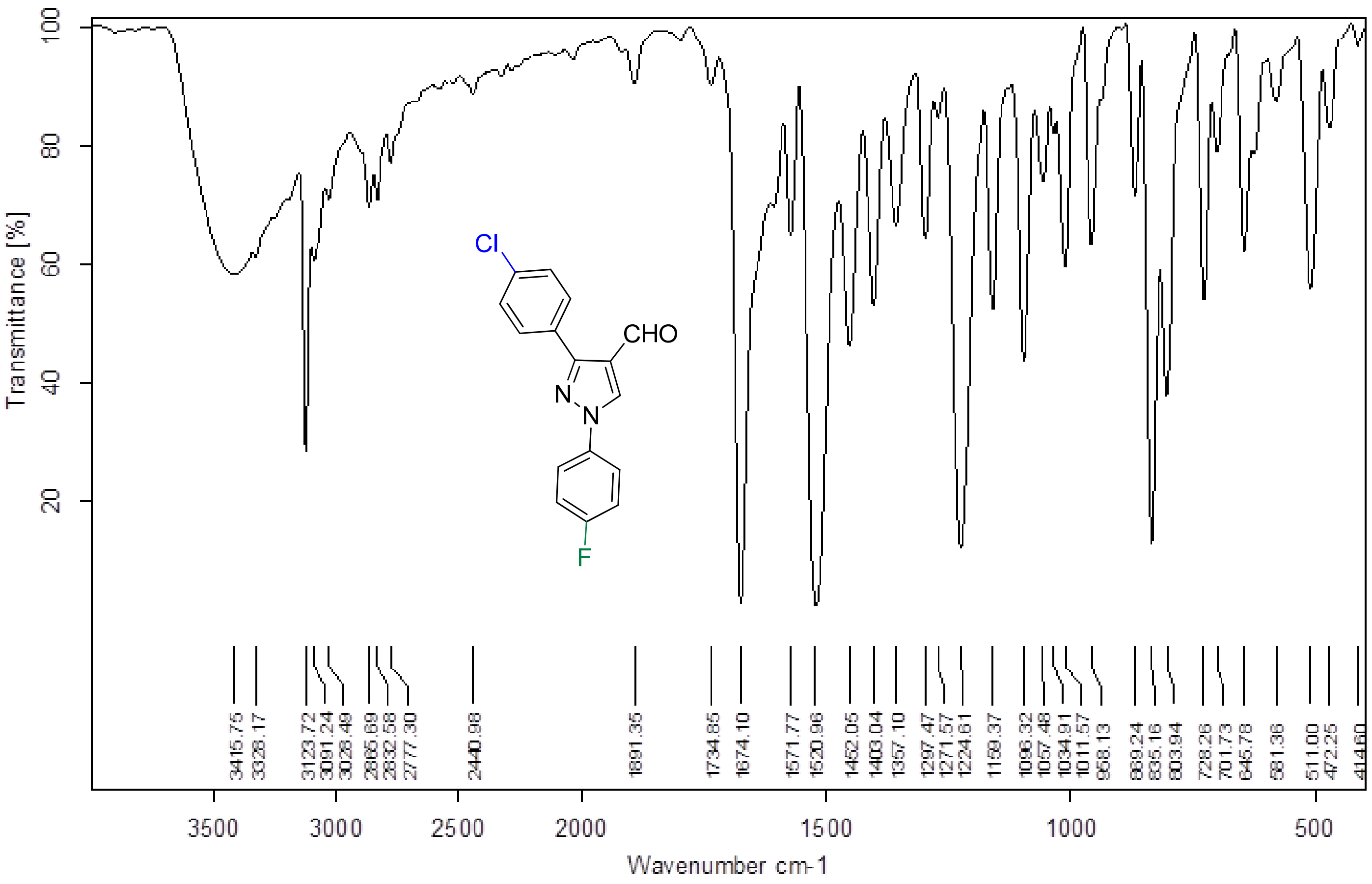

IR (KBr) spectrum of 3-(4-chlorophenyl)-1-(4-fluorophenyl)-1*H*-pyrazole-4-carbaldehyde **2i**

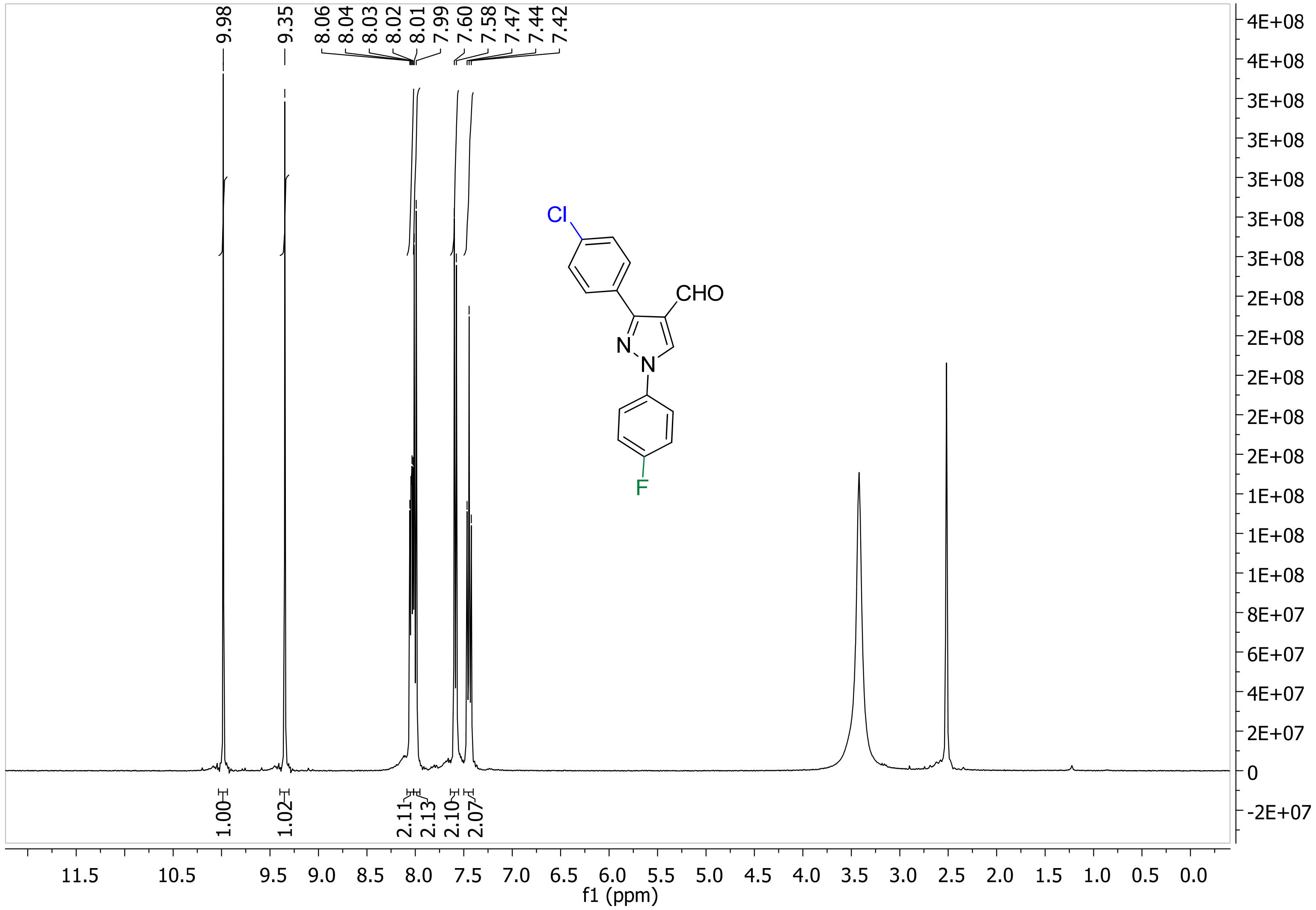

^{1}H NMR (DMSO-*d6*, 400 MHz) spectrum of 3-(4-chlorophenyl)-1-(4-fluorophenyl)-1*H*-pyrazole-4-carbaldehyde **2i**

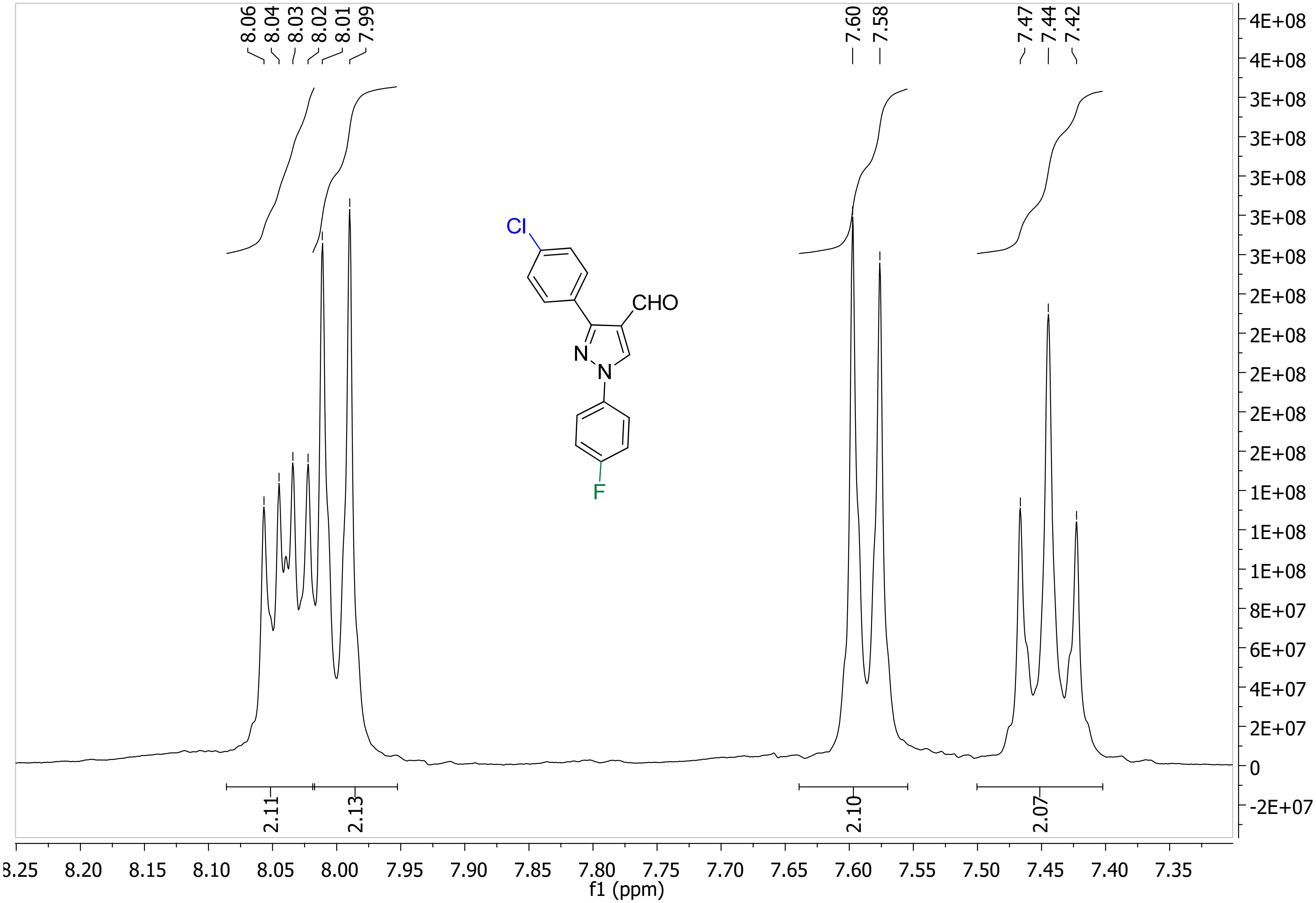

^{1}H NMR (DMSO-*d6*, 400 MHz) spectrum of 3-(4-chlorophenyl)-1-(4-fluorophenyl)-1*H*-pyrazole-4-carbaldehyde **2i**

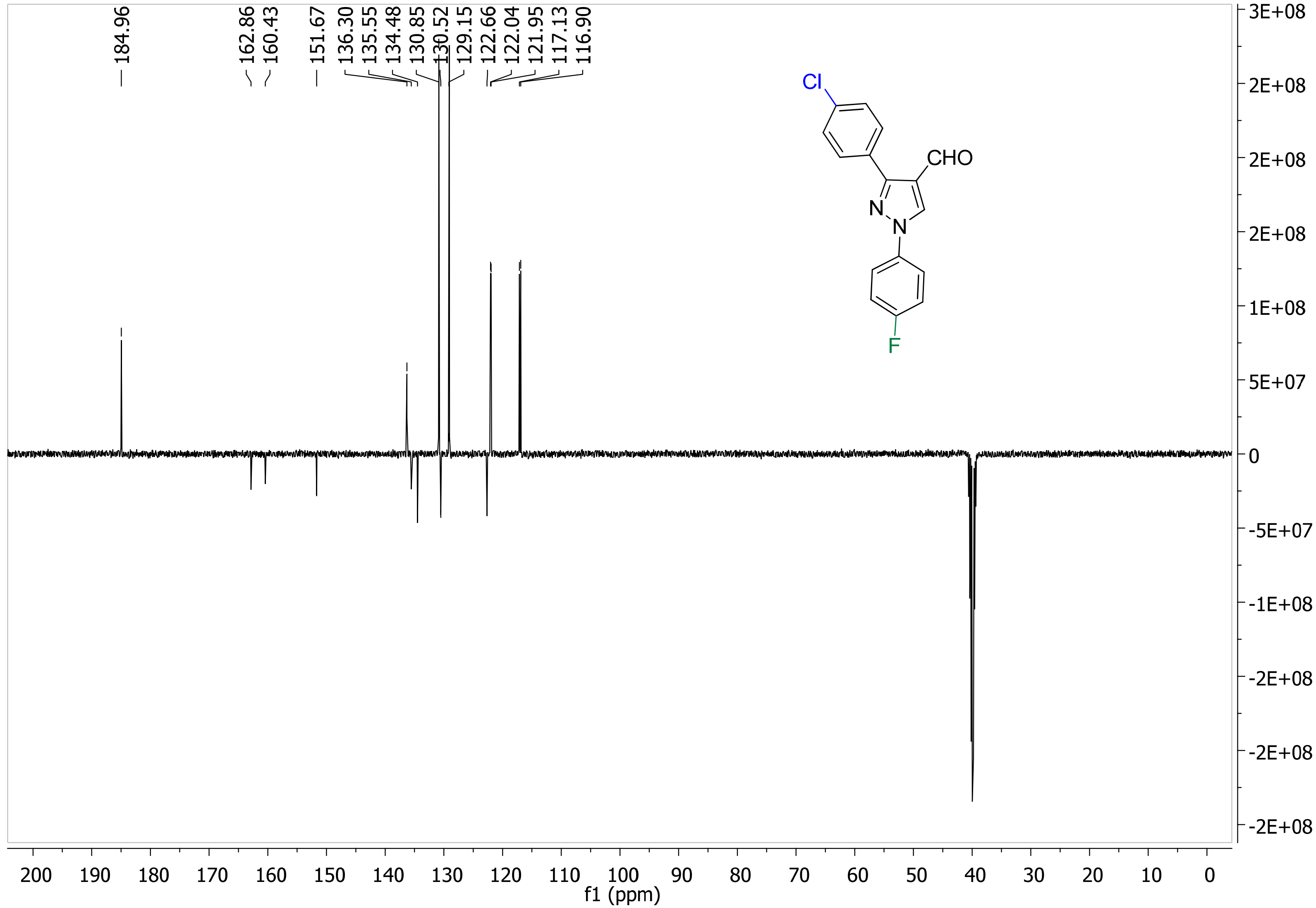

^{13}C APT NMR (DMSO-*d6*, 101 MHz) spectrum of 3-(4-chlorophenyl)-1-(4-fluorophenyl)-1*H*-pyrazole-4-carbaldehyde **2i**

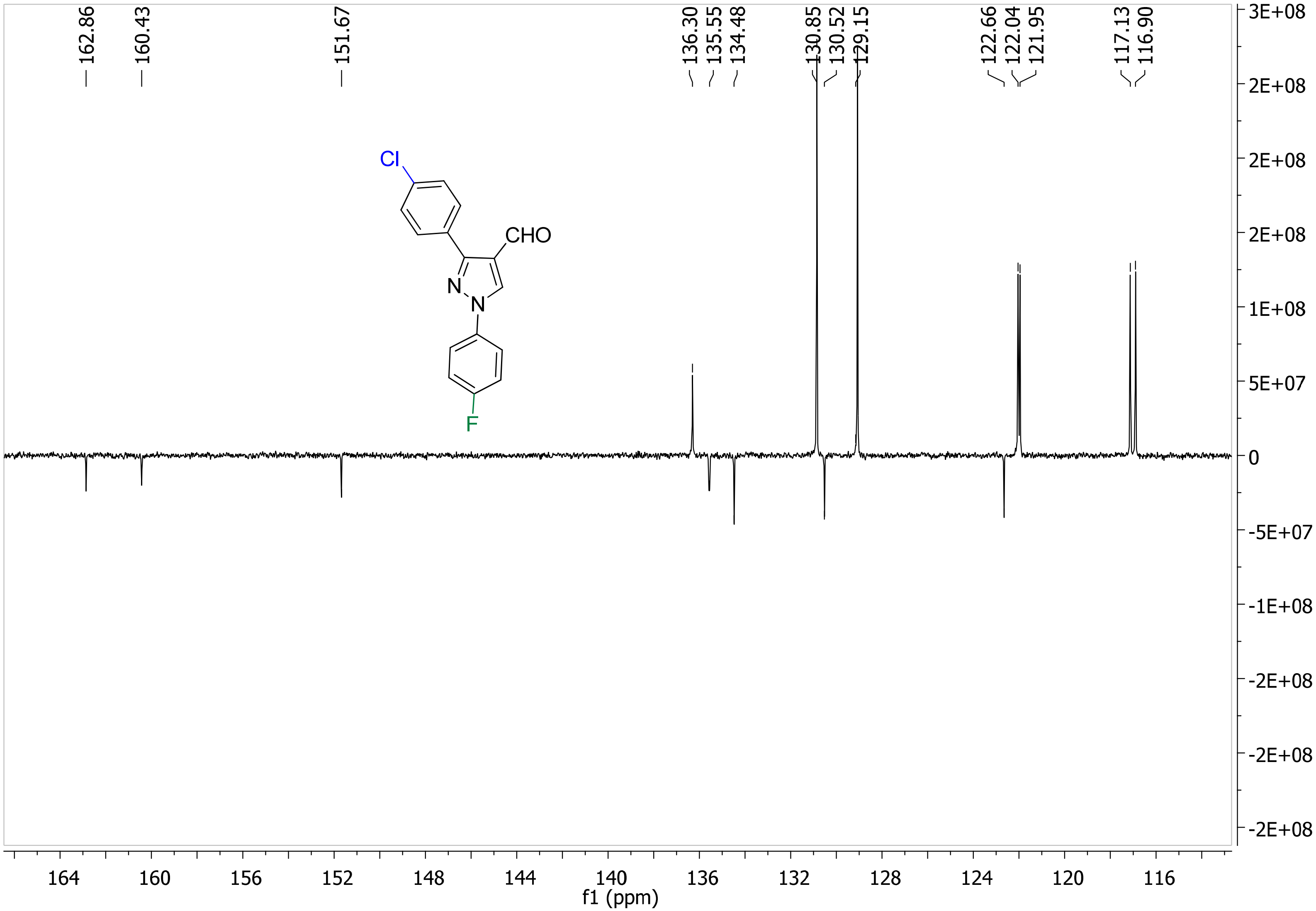

^{13}C APT NMR (DMSO-*d6*, 101 MHz) spectrum of 3-(4-chlorophenyl)-1-(4-fluorophenyl)-1*H*-pyrazole-4-carbaldehyde **2i**

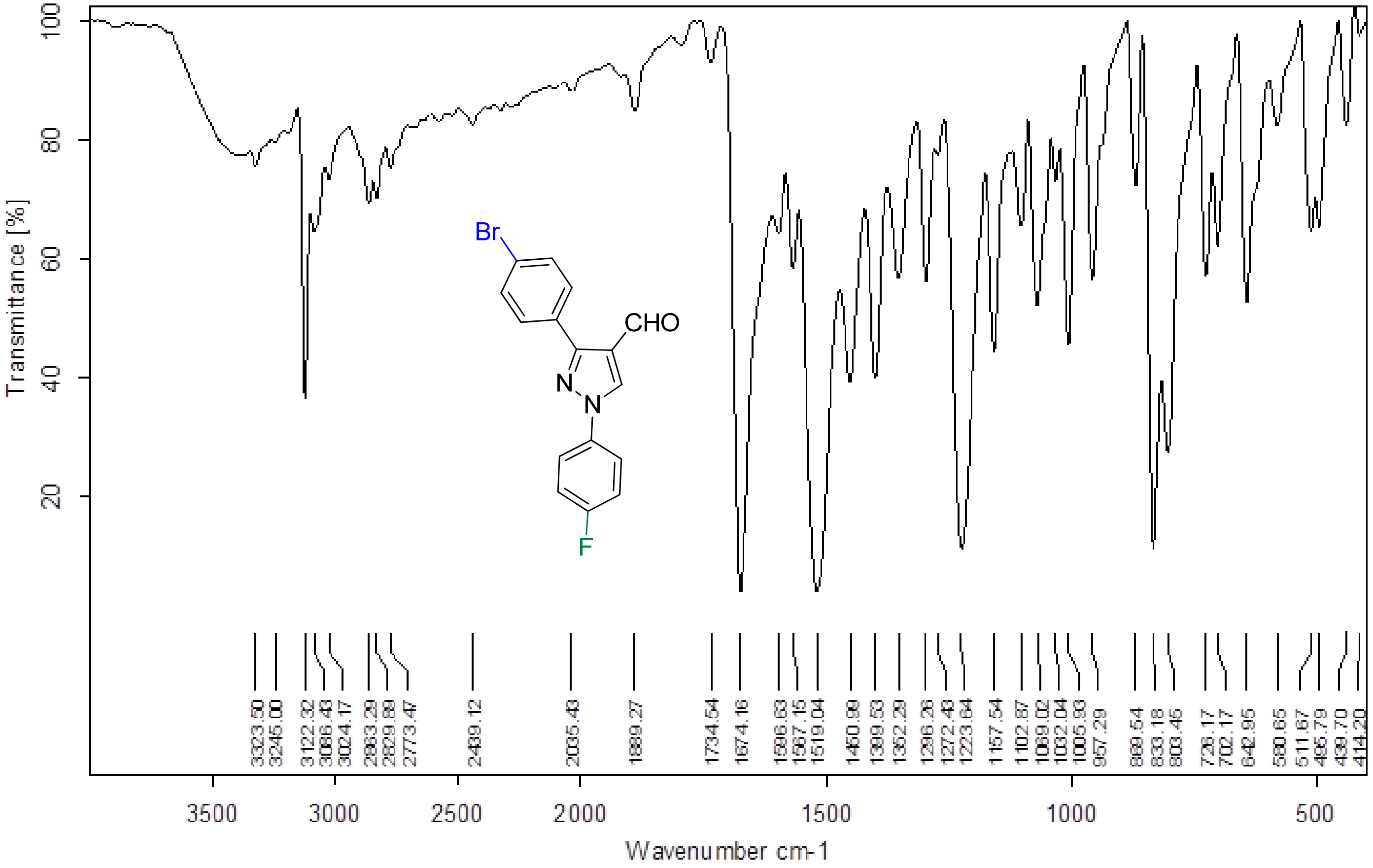

IR (KBr) spectrum of 3-(4-bromophenyl)-1-(4-fluorophenyl)-1*H*-pyrazole-4-carbaldehyde **2j**

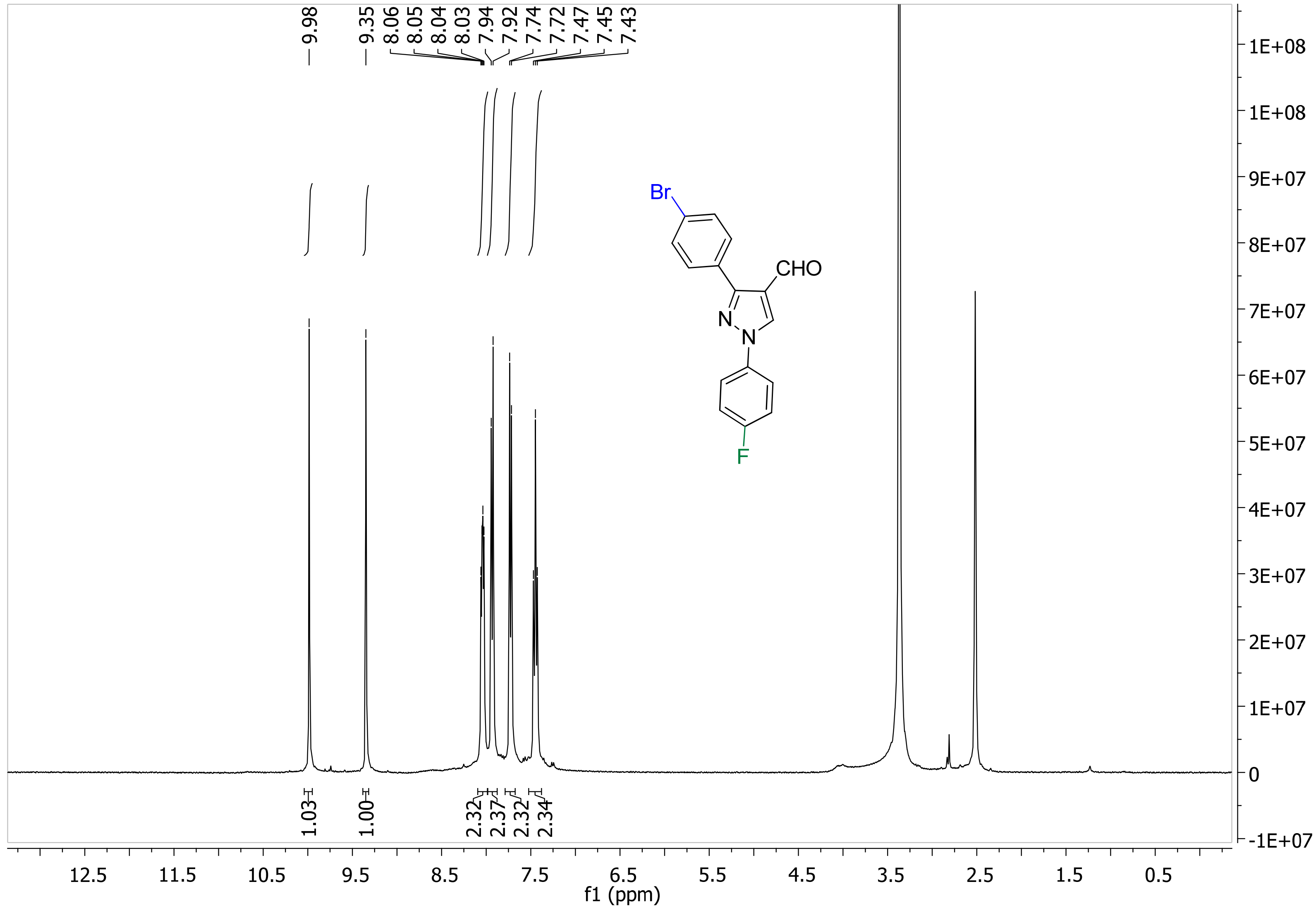

^{1}H NMR (DMSO-*d6*, 400 MHz) spectrum of 3-(4-bromophenyl)-1-(4-fluorophenyl)-1*H*-pyrazole-4-carbaldehyde **2j**

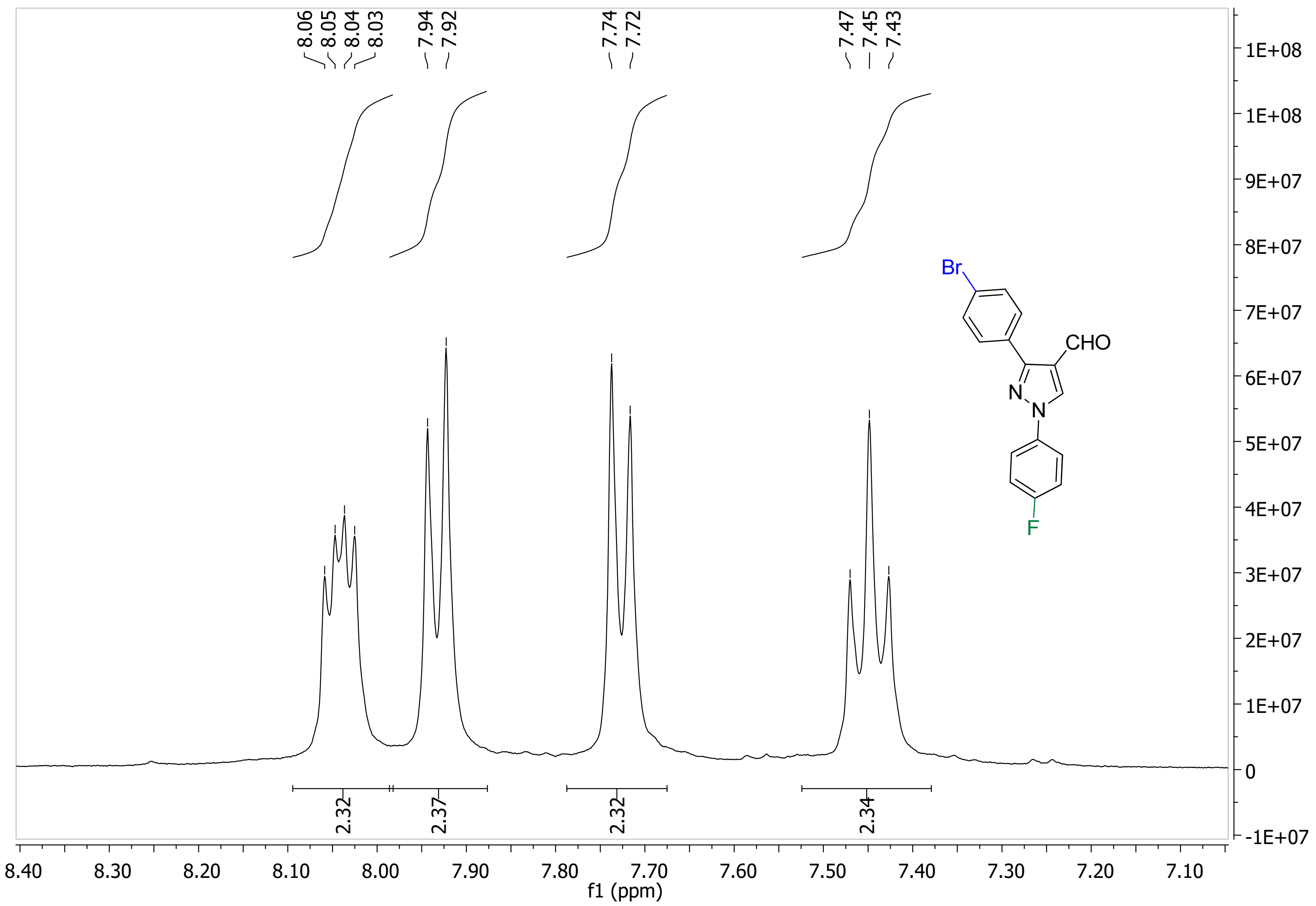

^{1}H NMR (DMSO-*d6*, 400 MHz) spectrum of 3-(4-bromophenyl)-1-(4-fluorophenyl)-1*H*-pyrazole-4-carbaldehyde **2j**

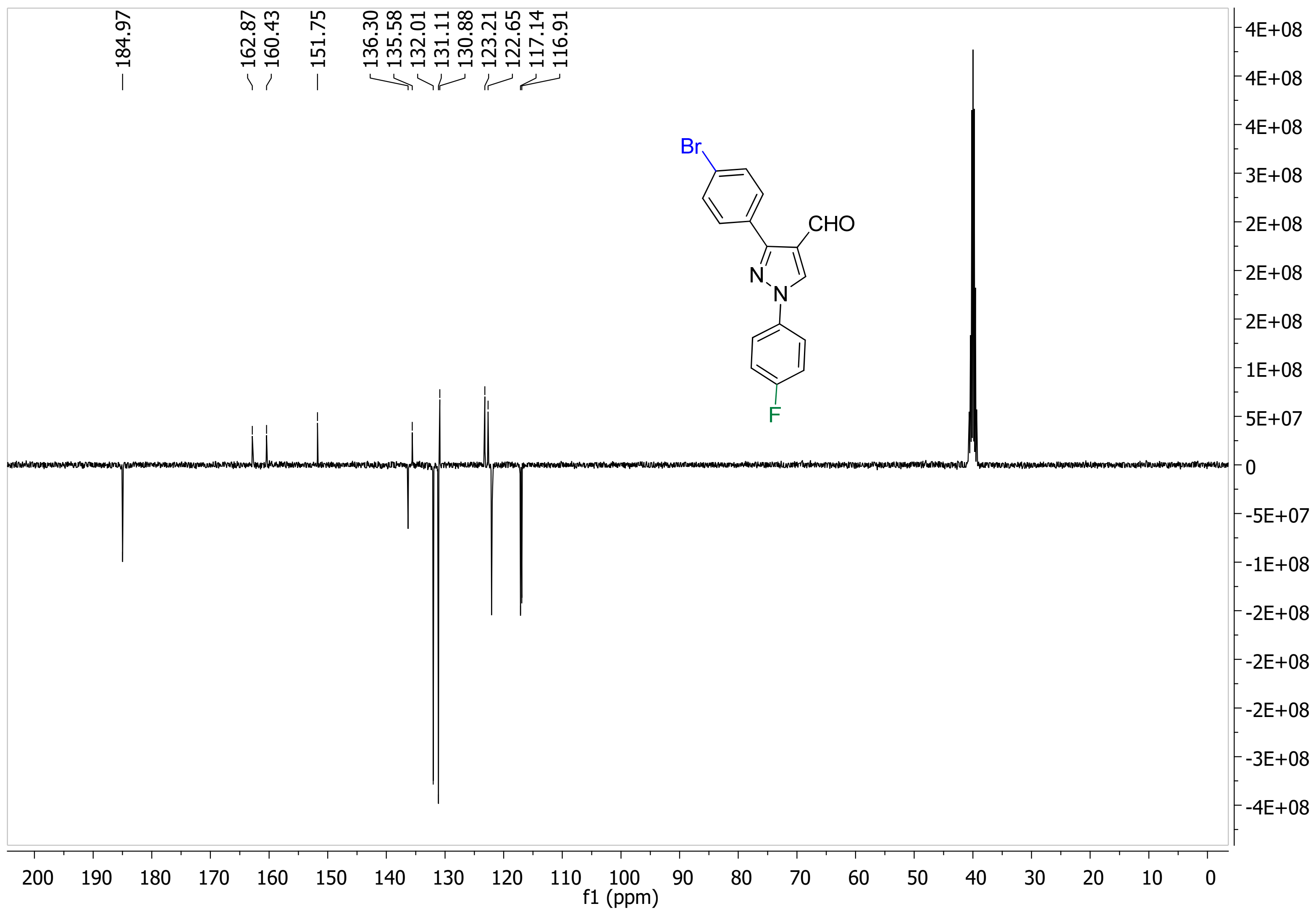

^{13}C APT NMR (DMSO-*d6*, 101 MHz) spectrum of 3-(4-bromophenyl)-1-(4-fluorophenyl)-1*H*-pyrazole-4-carbaldehyde **2j**

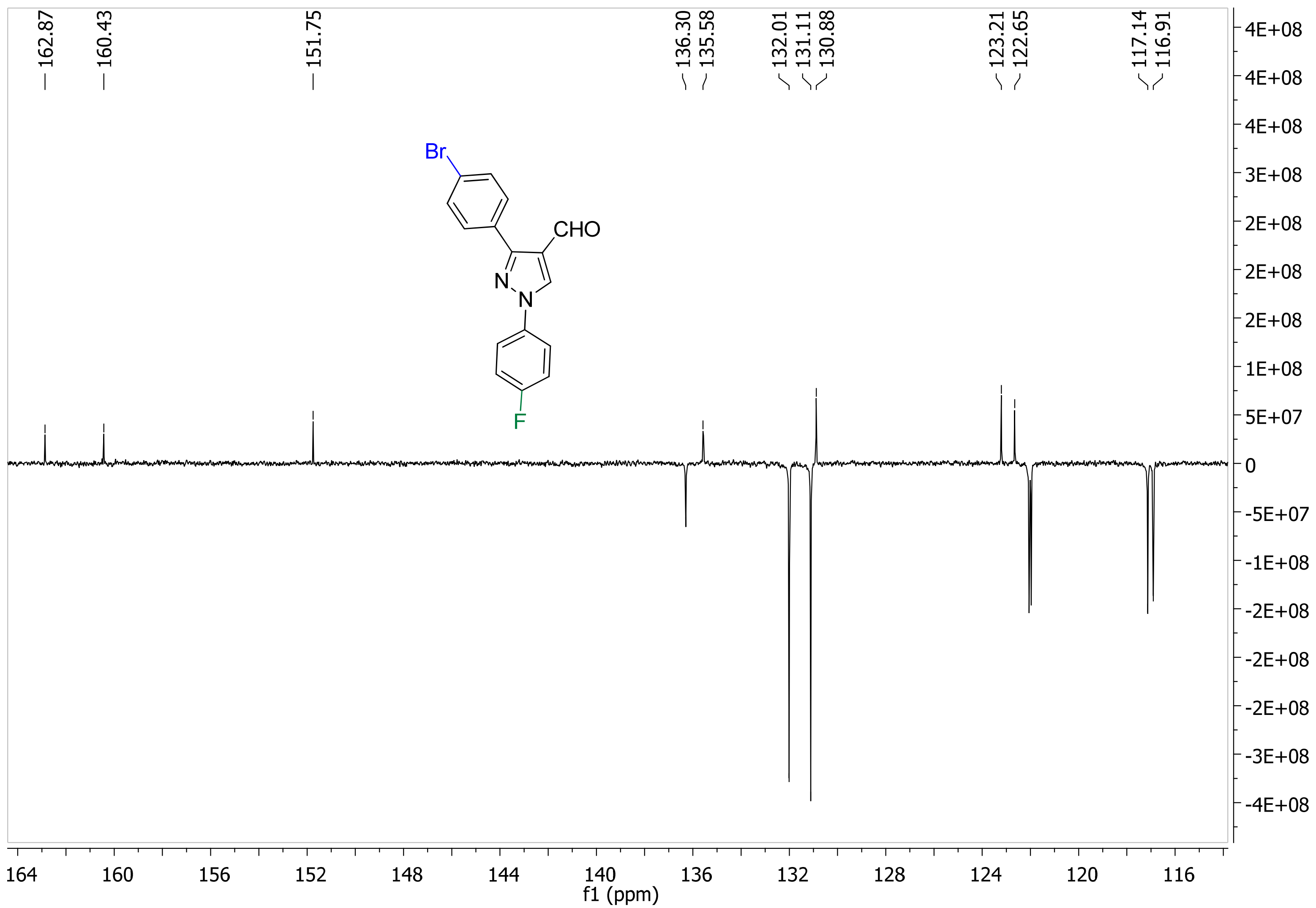

^{13}C APT NMR (DMSO-*d6*, 101 MHz) spectrum of 3-(4-bromophenyl)-1-(4-fluorophenyl)-1*H*-pyrazole-4-carbaldehyde **2j**

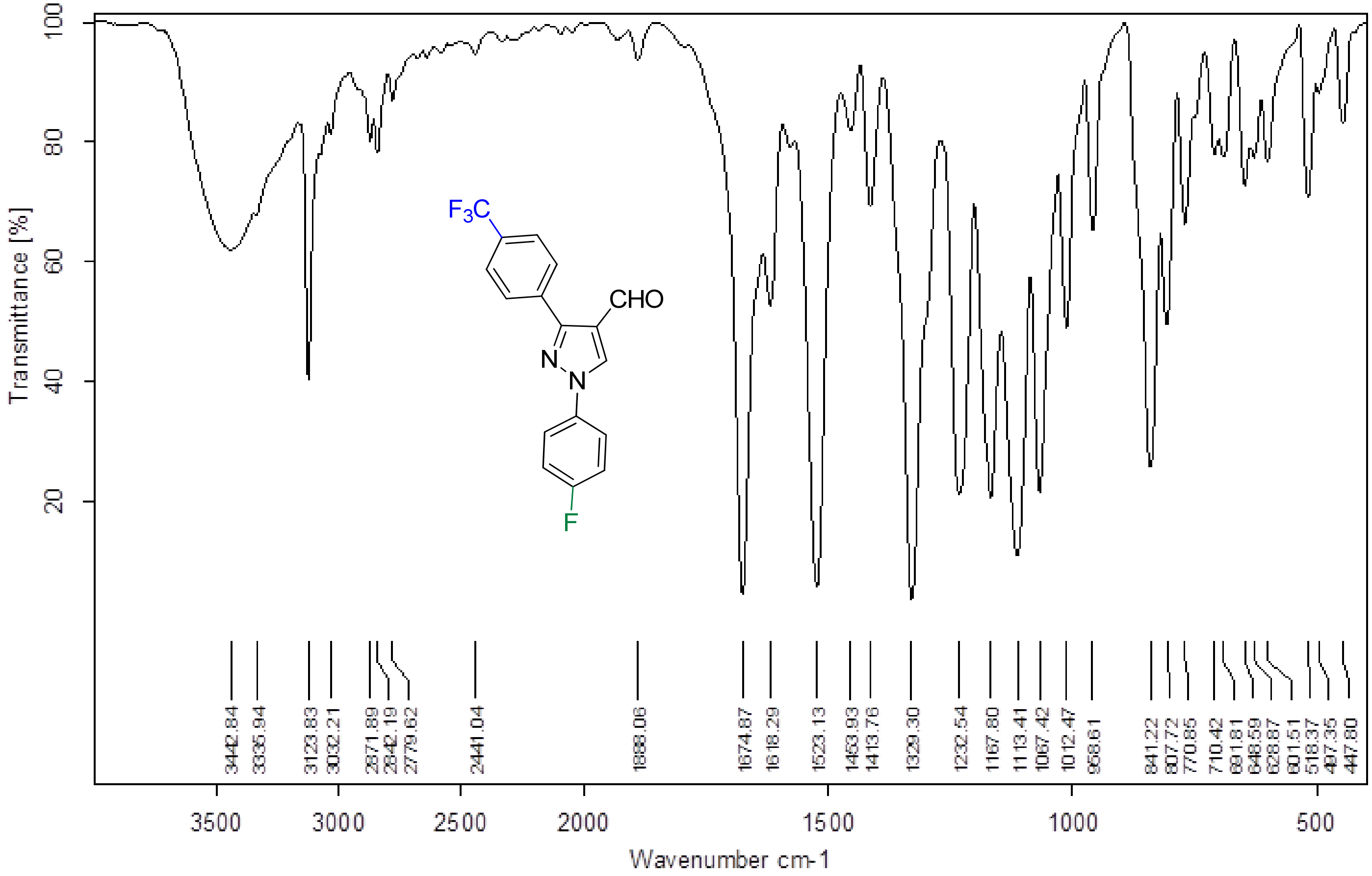

IR (KBr) spectrum of 1-(4-fluorophenyl)-3-(4-(trifluoromethyl)phenyl)-1*H*-pyrazole-4-carbaldehyde **2k**

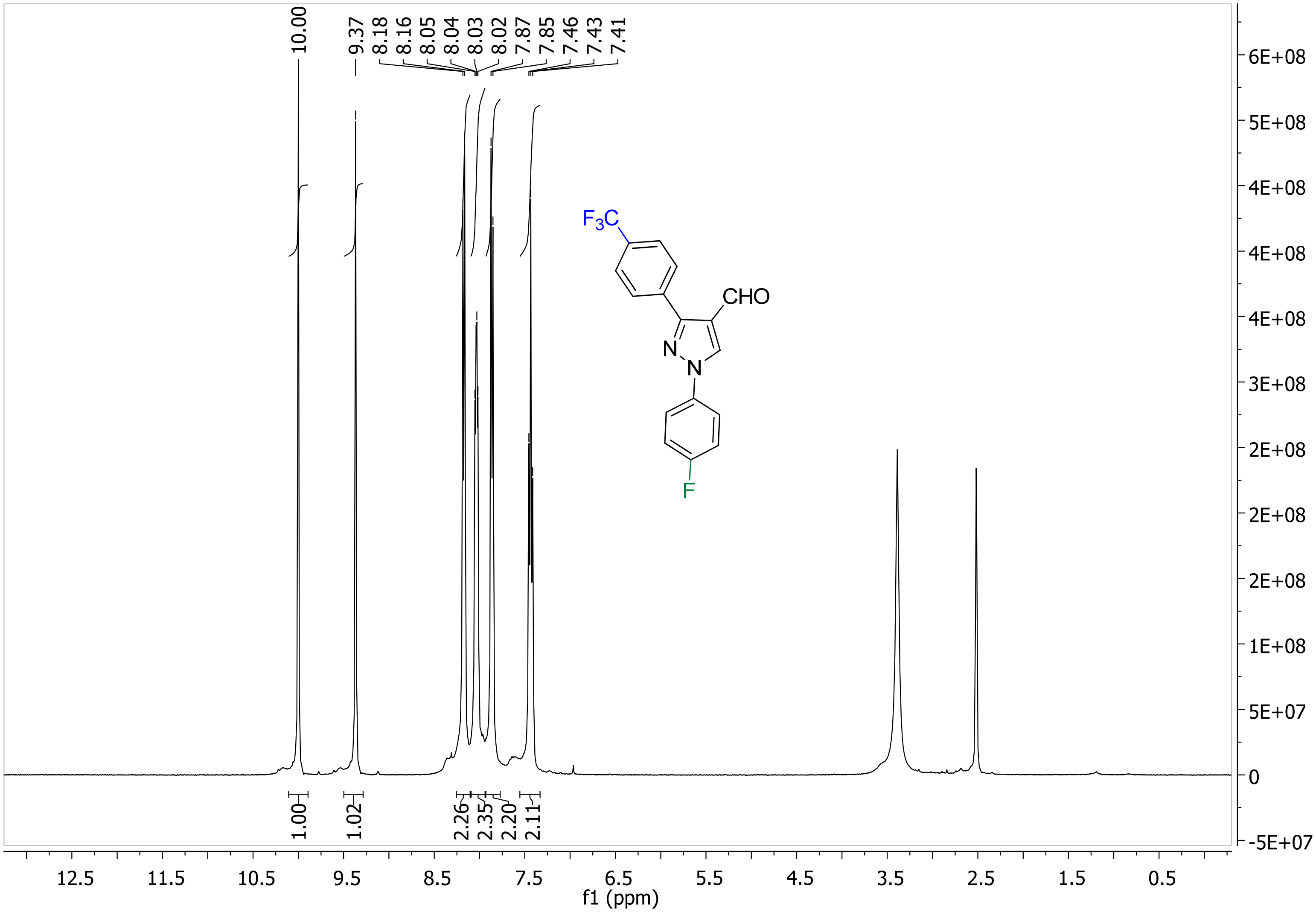

^{1}H NMR (DMSO-*d6*, 400 MHz) spectrum of 1-(4-fluorophenyl)-3-(4-(trifluoromethyl)phenyl)-1*H*-pyrazole-4-carbaldehyde **2k**

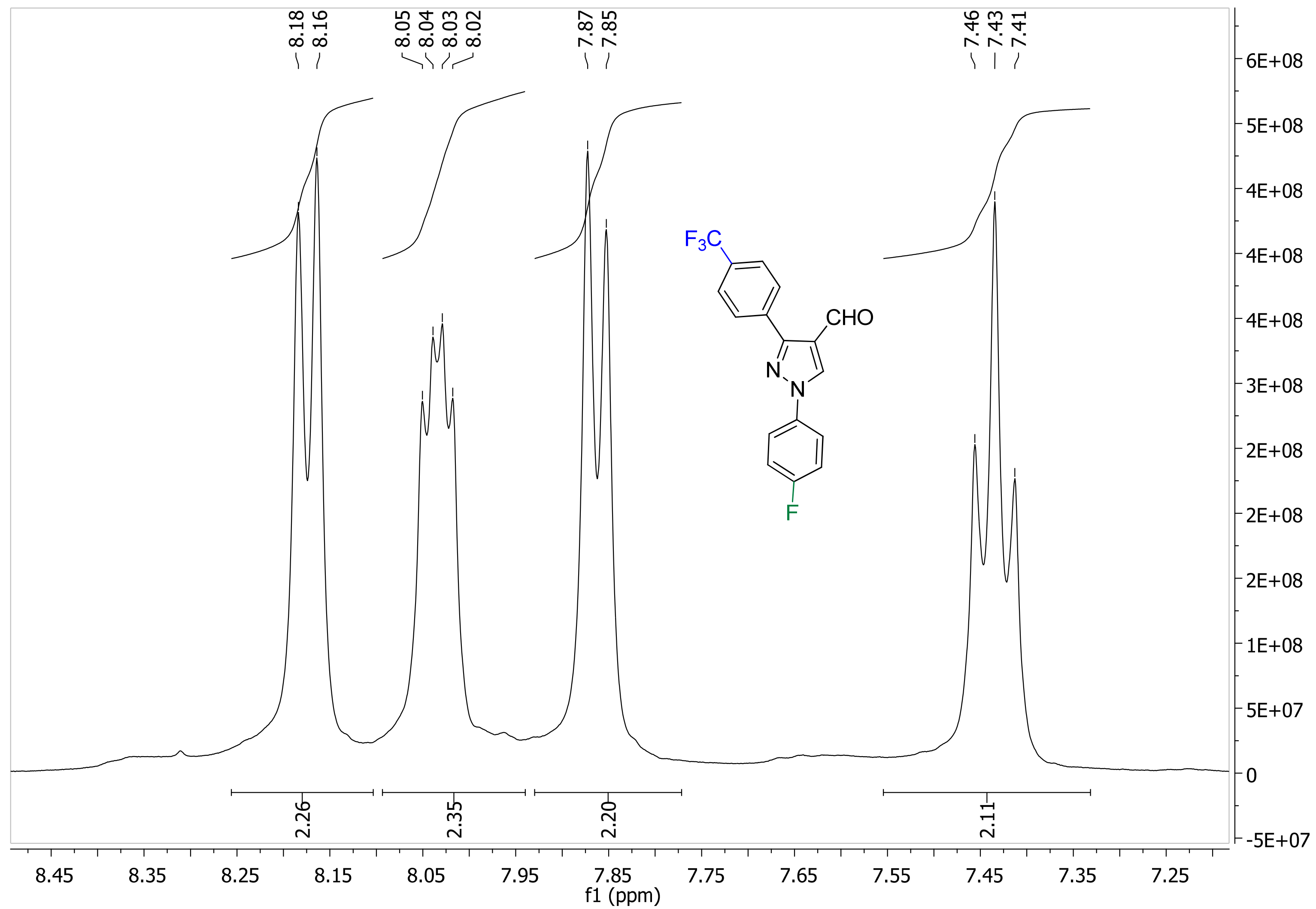

^{1}H NMR (DMSO-*d6*, 400 MHz) spectrum of 1-(4-fluorophenyl)-3-(4-(trifluoromethyl)phenyl)-1*H*-pyrazole-4-carbaldehyde **2k**

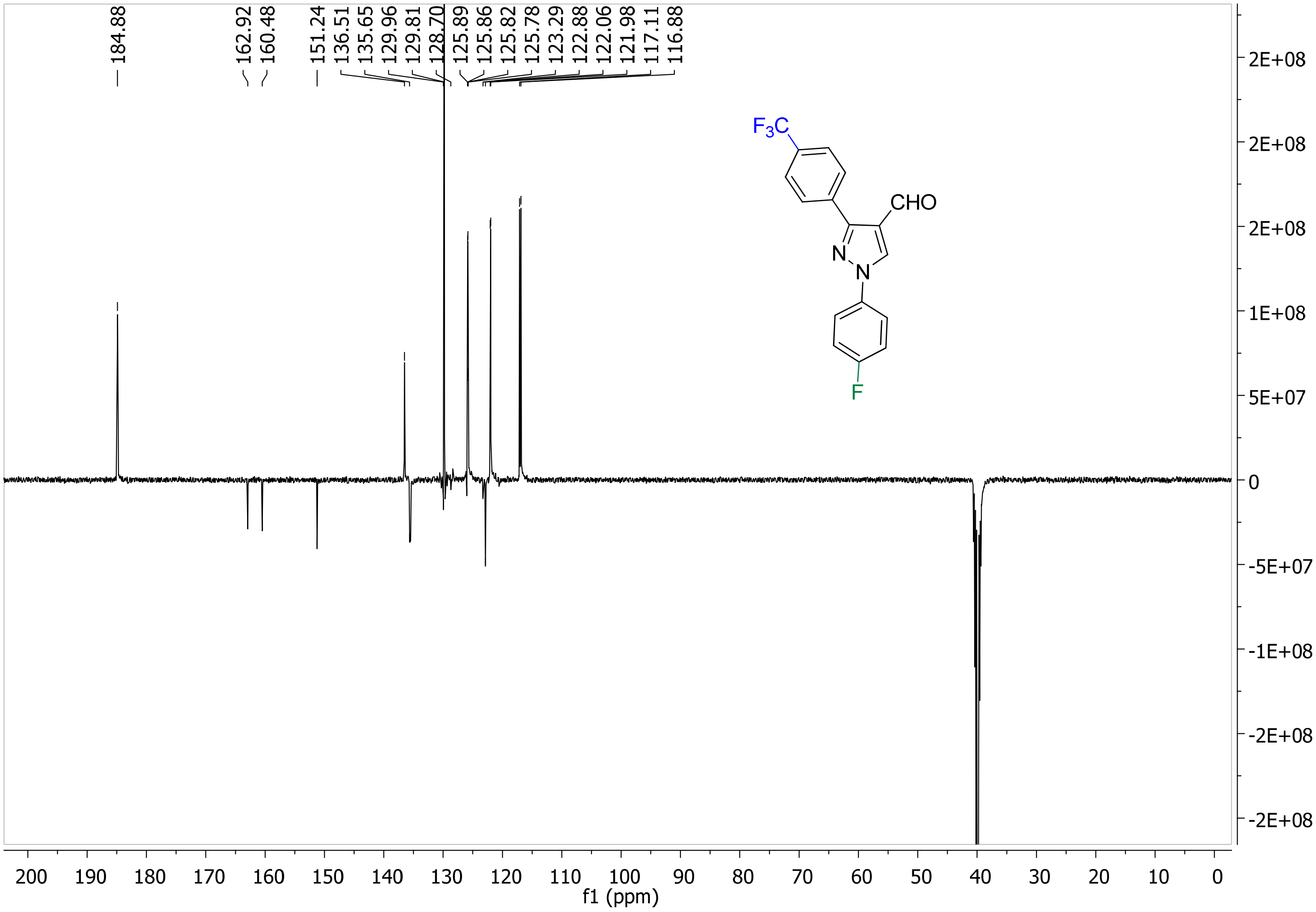

^{13}C APT NMR (DMSO-*d6*, 101 MHz) spectrum of 1-(4-fluorophenyl)-3-(4-(trifluoromethyl)phenyl)-1*H*-pyrazole-4-carbaldehyde **2k**

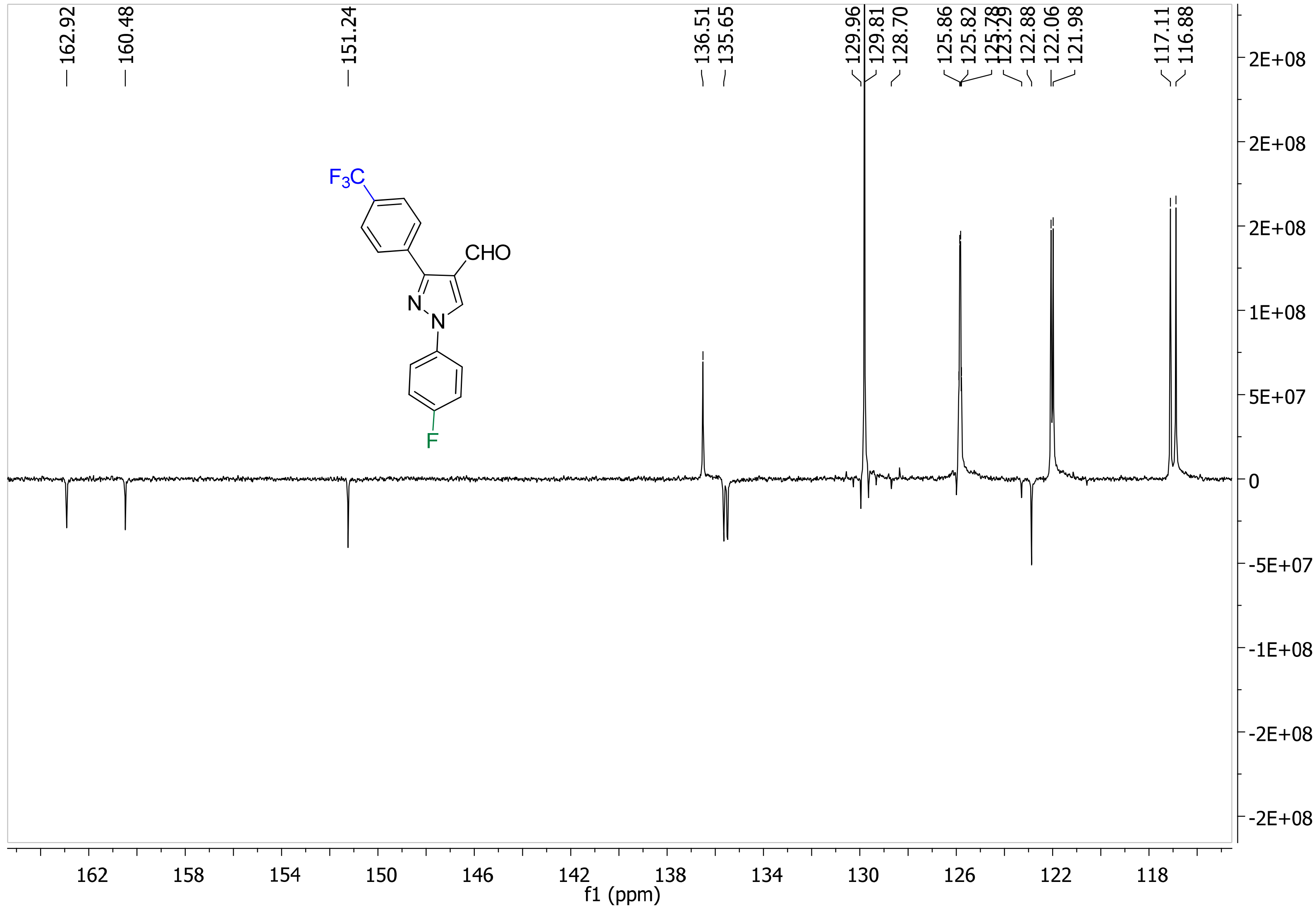

^{13}C APT NMR (DMSO-*d6*, 101 MHz) spectrum of 1-(4-fluorophenyl)-3-(4-(trifluoromethyl)phenyl)-1*H*-pyrazole-4-carbaldehyde **2k**

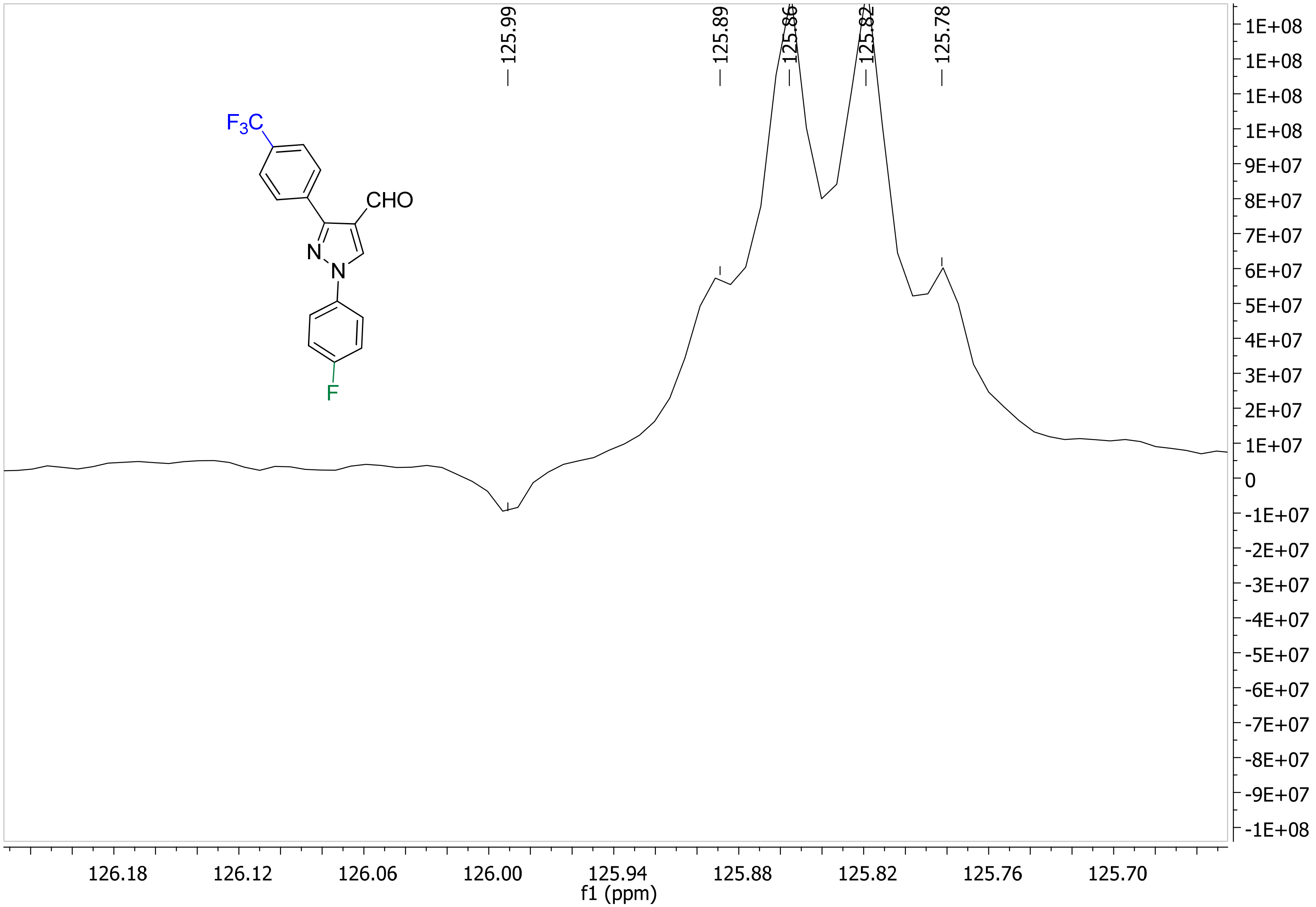

^{13}C APT NMR (DMSO-*d6*, 101 MHz) spectrum of 1-(4-fluorophenyl)-3-(4-(trifluoromethyl)phenyl)-1*H*-pyrazole-4-carbaldehyde **2k**

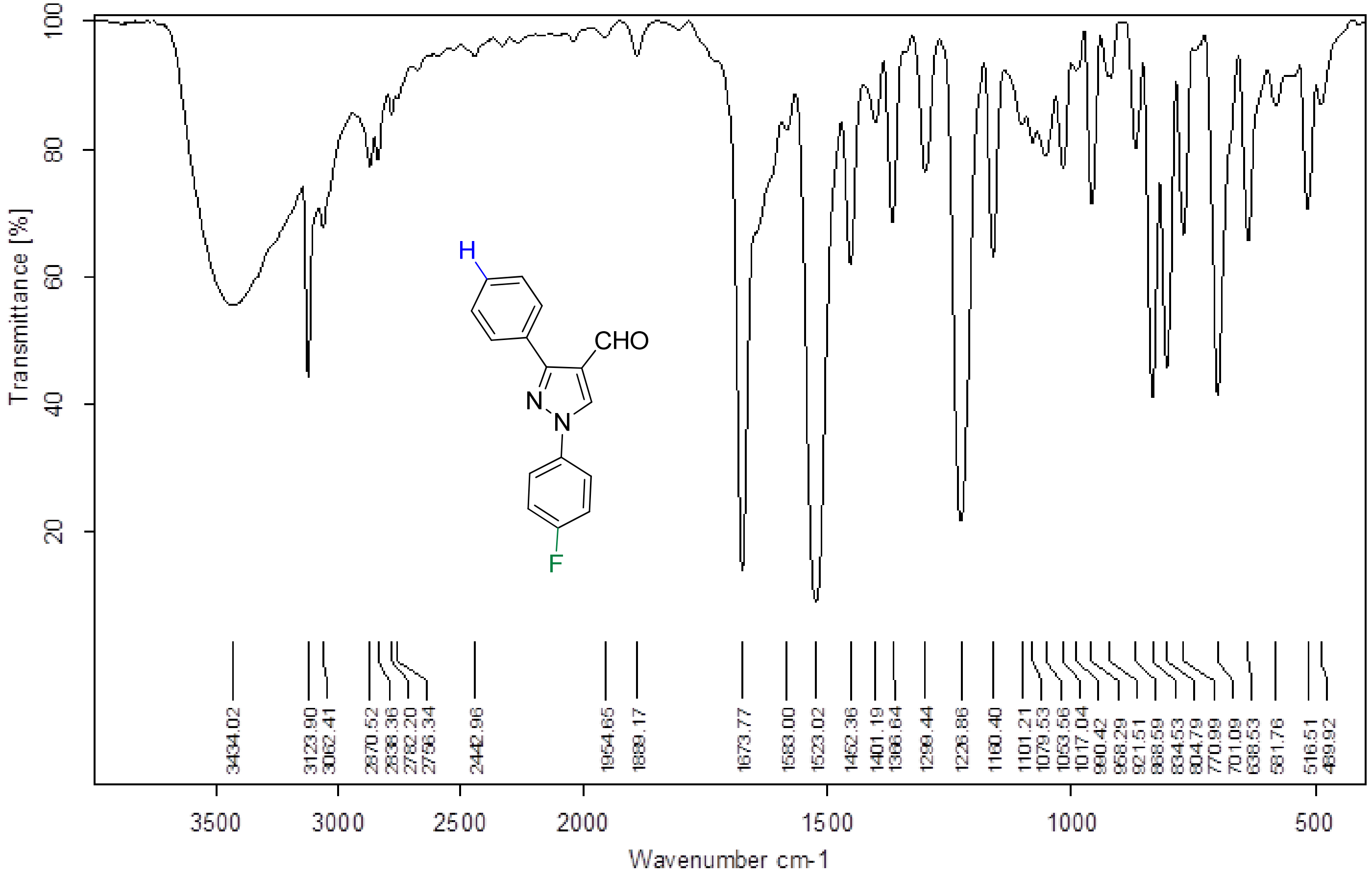

IR (KBr) spectrum of 1-(4-fluorophenyl)-3-phenyl-1*H*-pyrazole-4-carbaldehyde **2l**

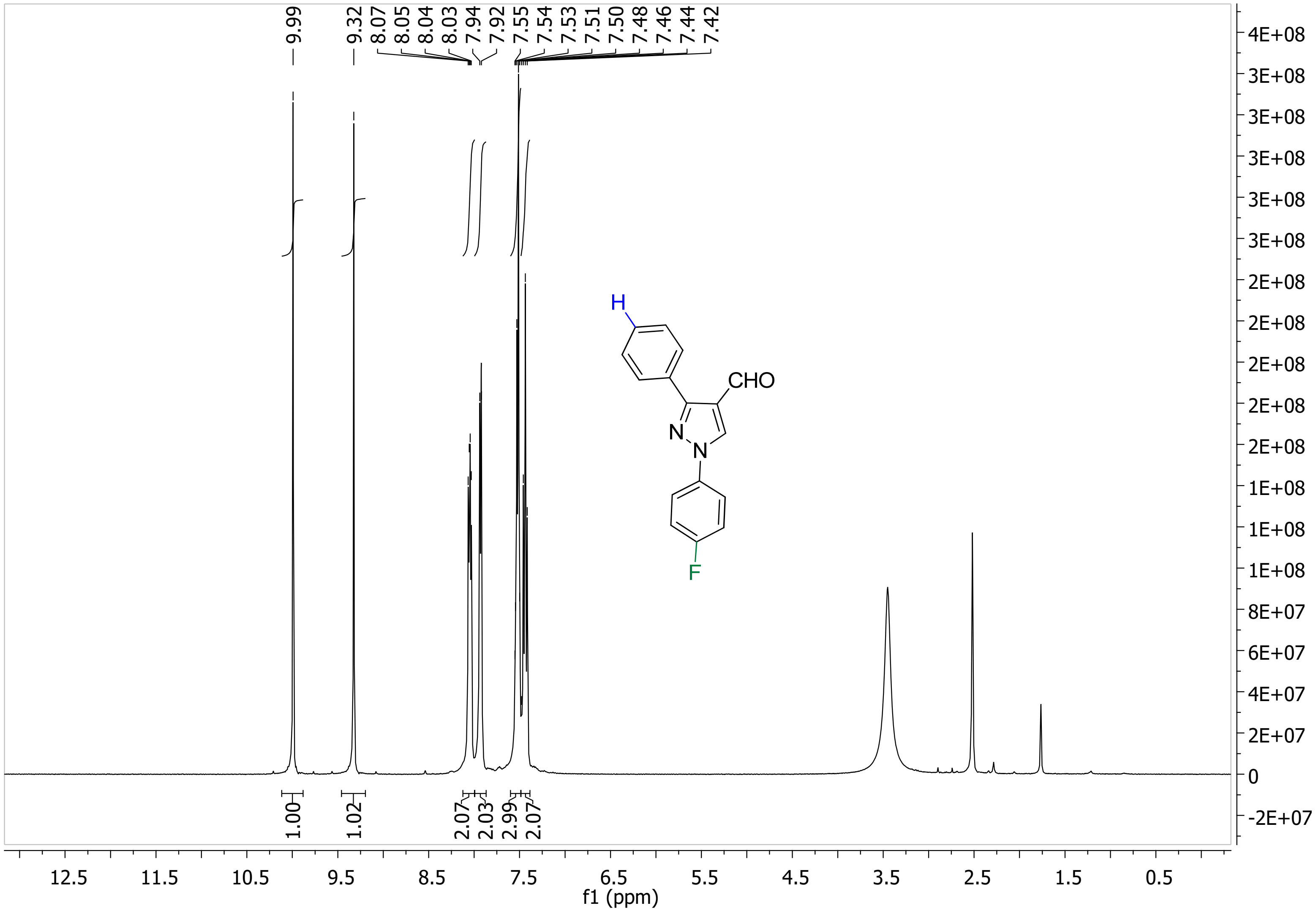

^{1}H NMR (DMSO-*d6*, 400 MHz) spectrum of 1-(4-fluorophenyl)-3-phenyl-1*H*-pyrazole-4-carbaldehyde **2l**

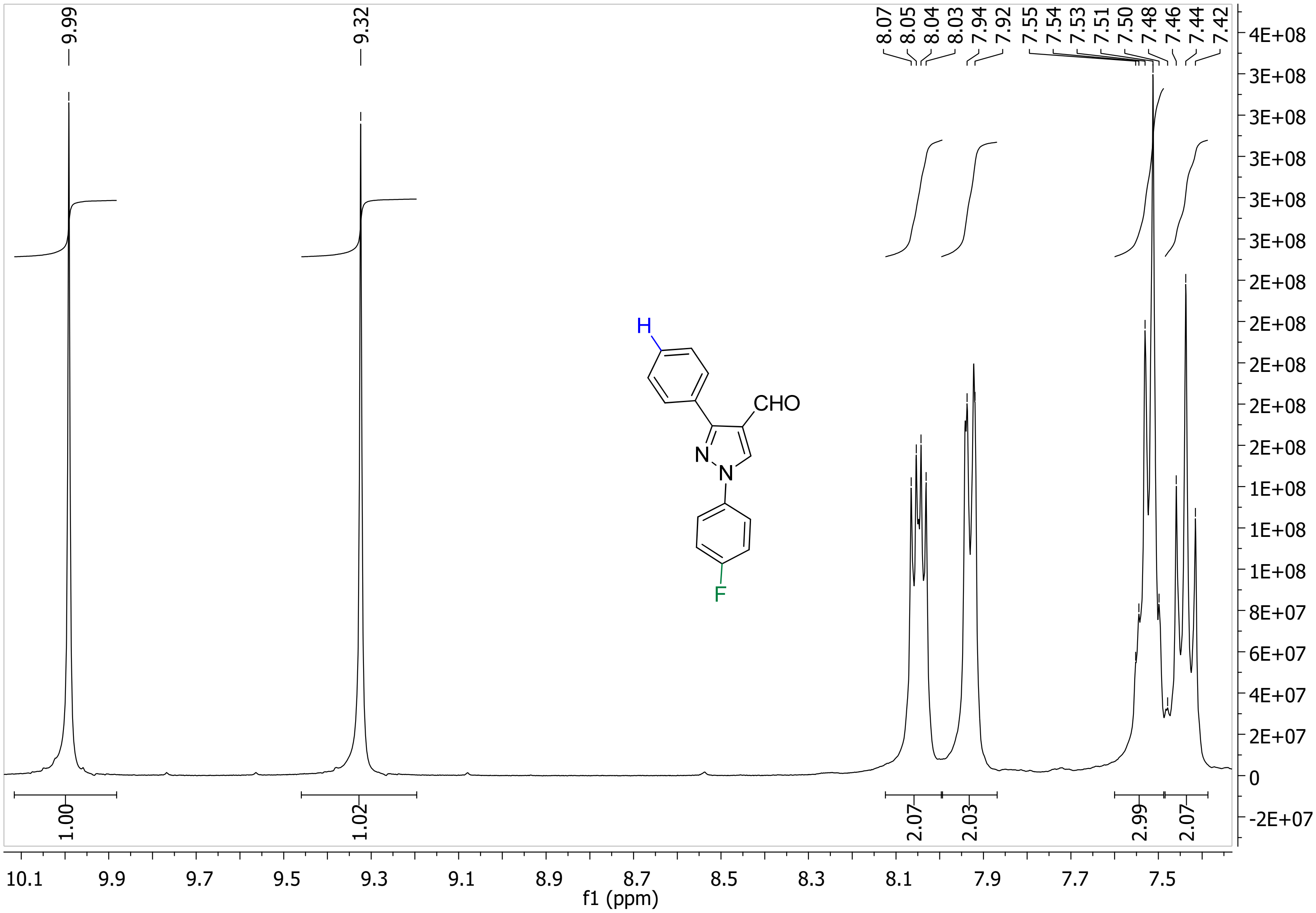

^{1}H NMR (DMSO-*d6*, 400 MHz) spectrum of 1-(4-fluorophenyl)-3-phenyl-1*H*-pyrazole-4-carbaldehyde **2l**

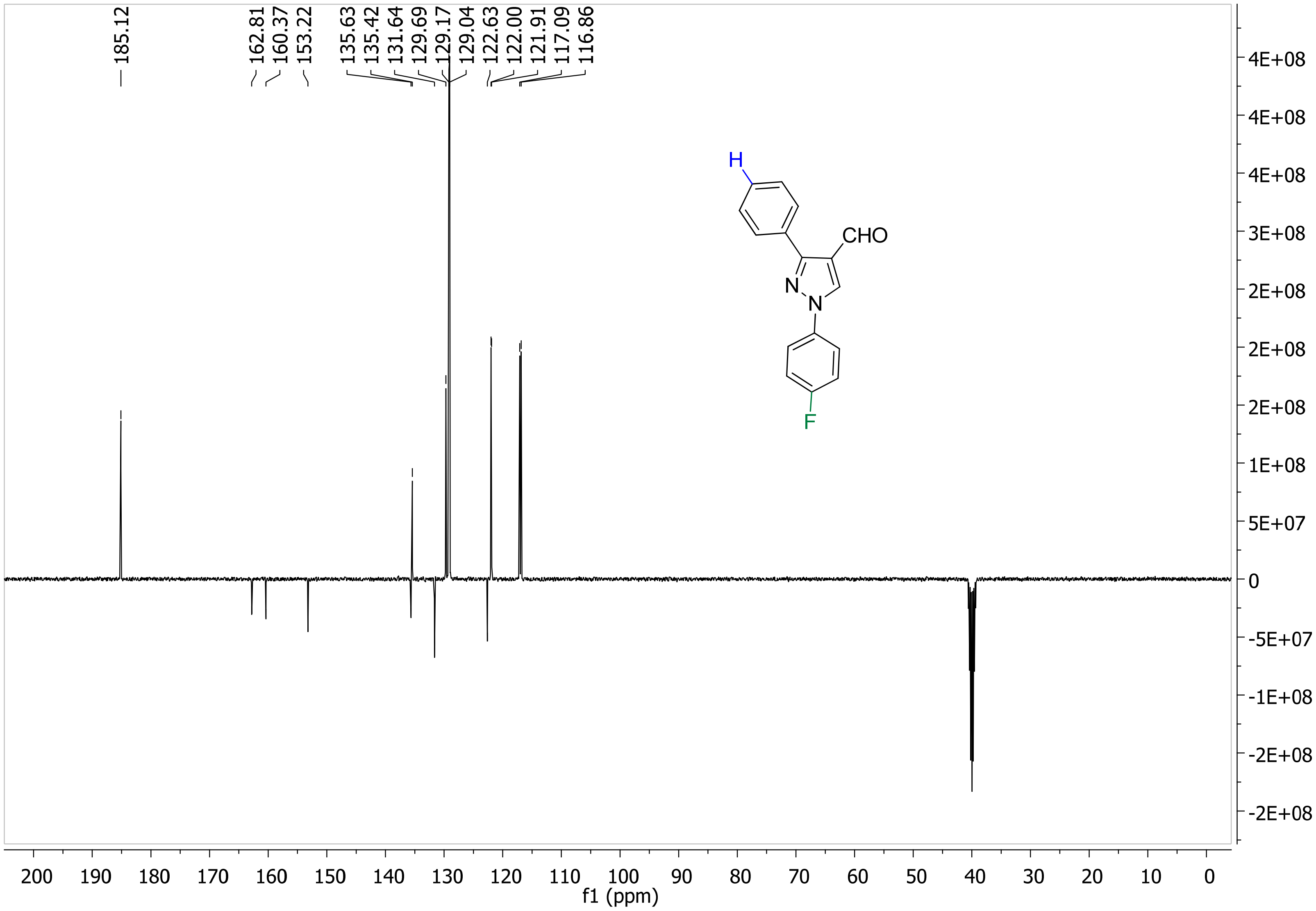

^{13}C APT NMR (DMSO-*d6*, 101 MHz) spectrum of 1-(4-fluorophenyl)-3-phenyl-1*H*-pyrazole-4-carbaldehyde **2l**

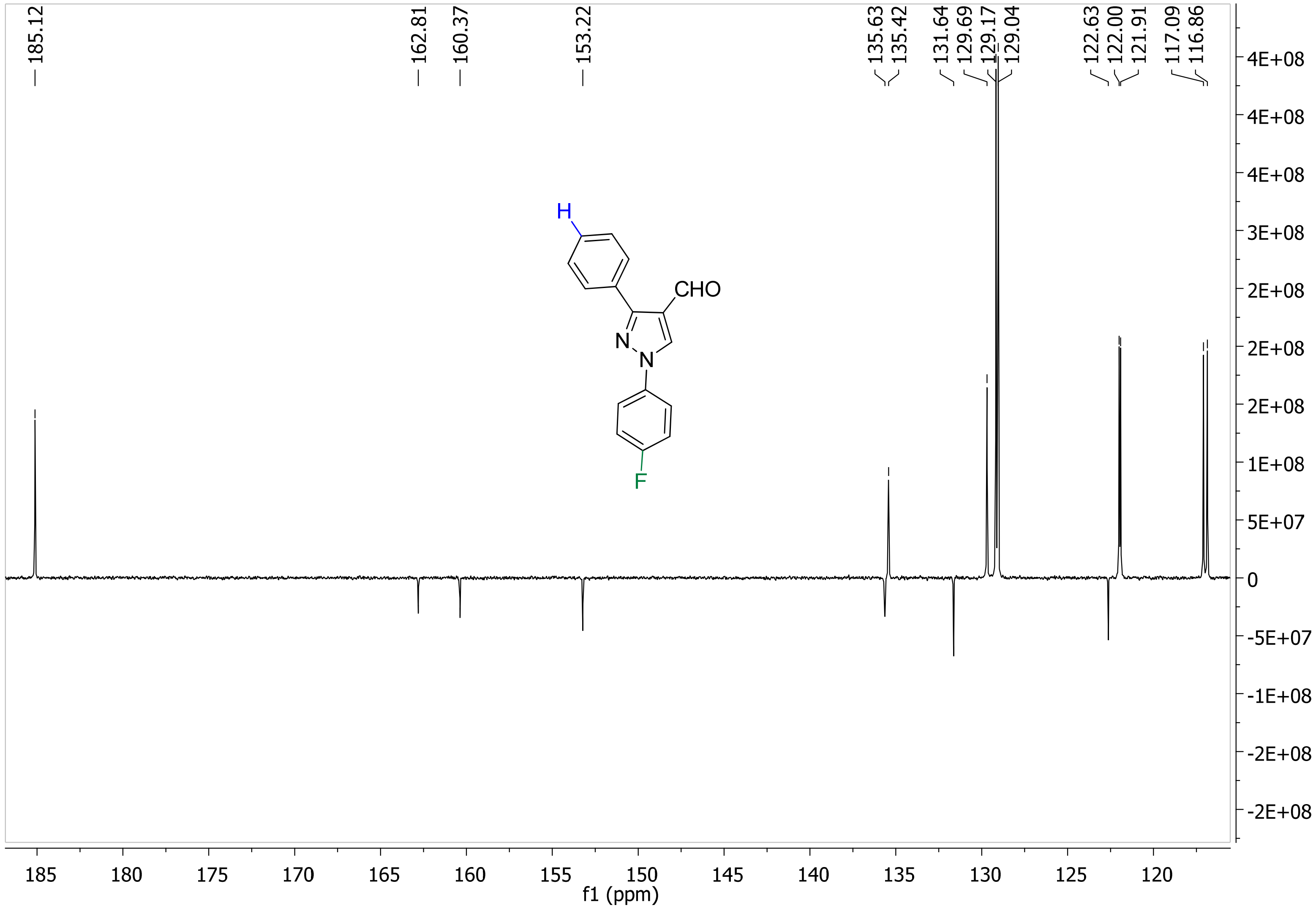

^{13}C APT NMR (DMSO-*d6*, 101 MHz) spectrum of 1-(4-fluorophenyl)-3-phenyl-1*H*-pyrazole-4-carbaldehyde **2l**

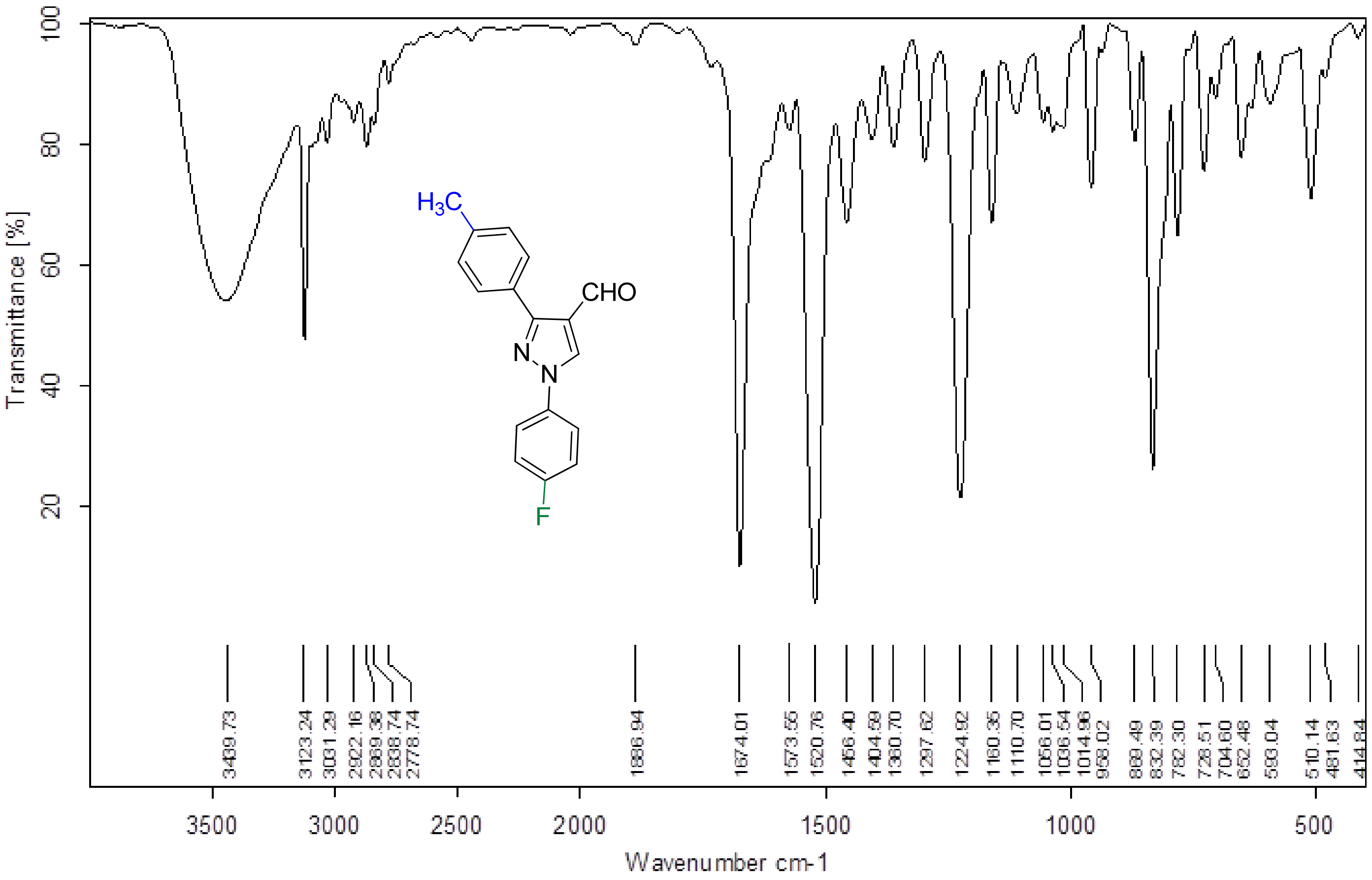

IR (KBr) spectrum of 1-(4-fluorophenyl)-3-(*p*-tolyl)-1*H*-pyrazole-4-carbaldehyde **2m**

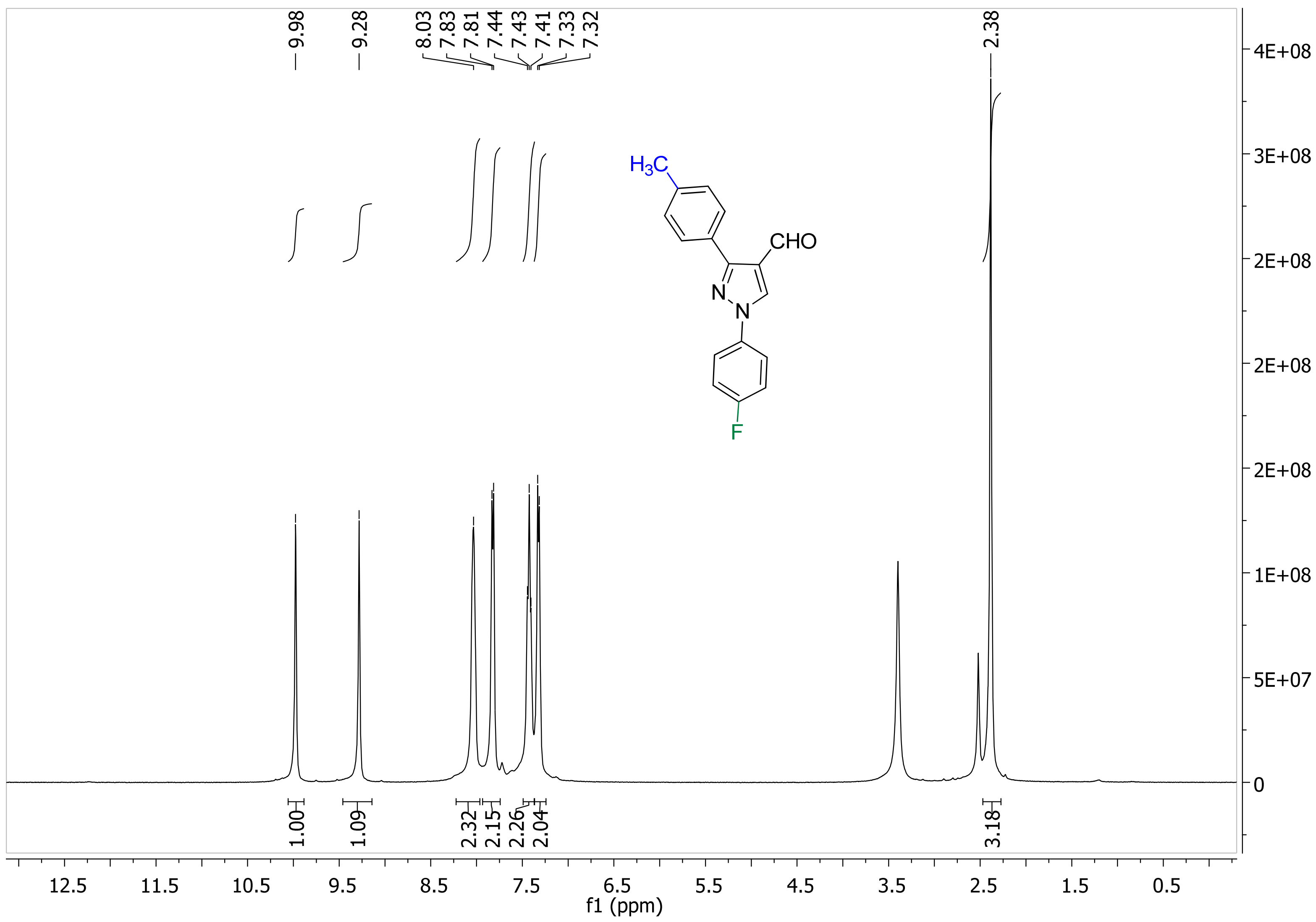

^{1}H NMR (DMSO-*d6*, 400 MHz) spectrum of 1-(4-fluorophenyl)-3-(*p*-tolyl)-1*H*-pyrazole-4-carbaldehyde **2m**

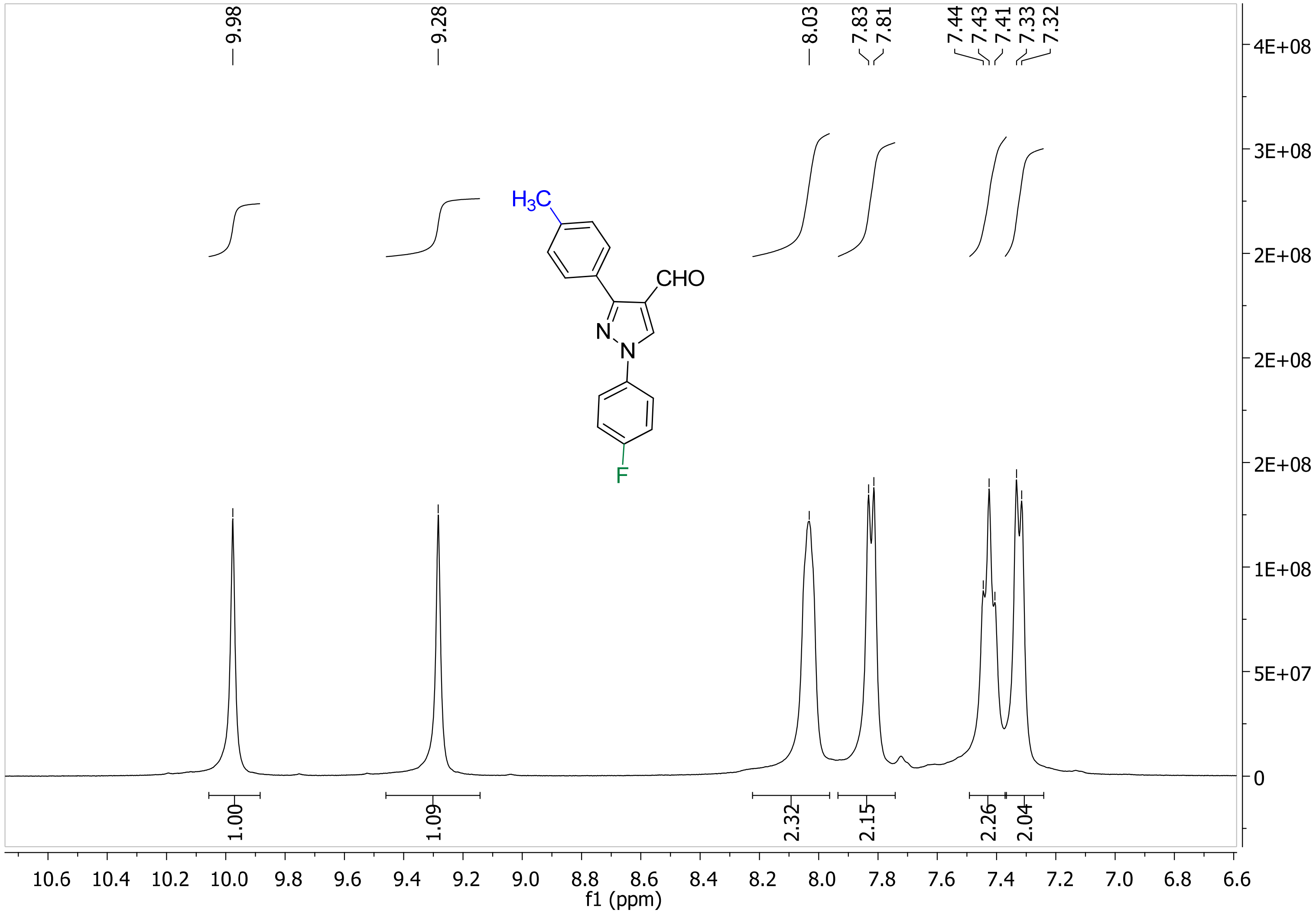

^{1}H NMR (DMSO-*d6*, 400 MHz) spectrum of 1-(4-fluorophenyl)-3-(*p*-tolyl)-1*H*-pyrazole-4-carbaldehyde **2m**

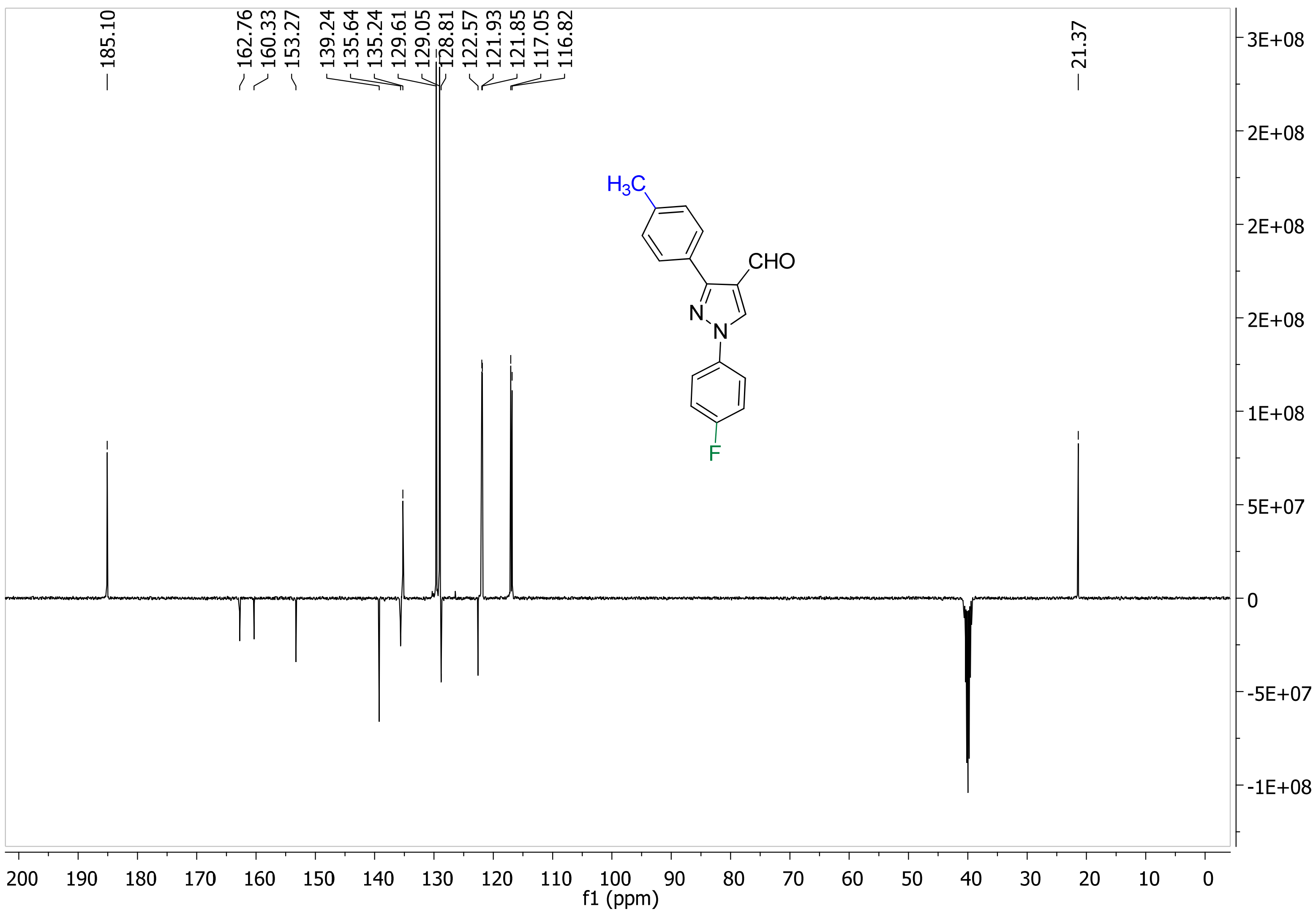

^{13}C APT NMR (DMSO-*d6*, 101 MHz) spectrum of 1-(4-fluorophenyl)-3-(*p*-tolyl)-1*H*-pyrazole-4-carbaldehyde **2m**

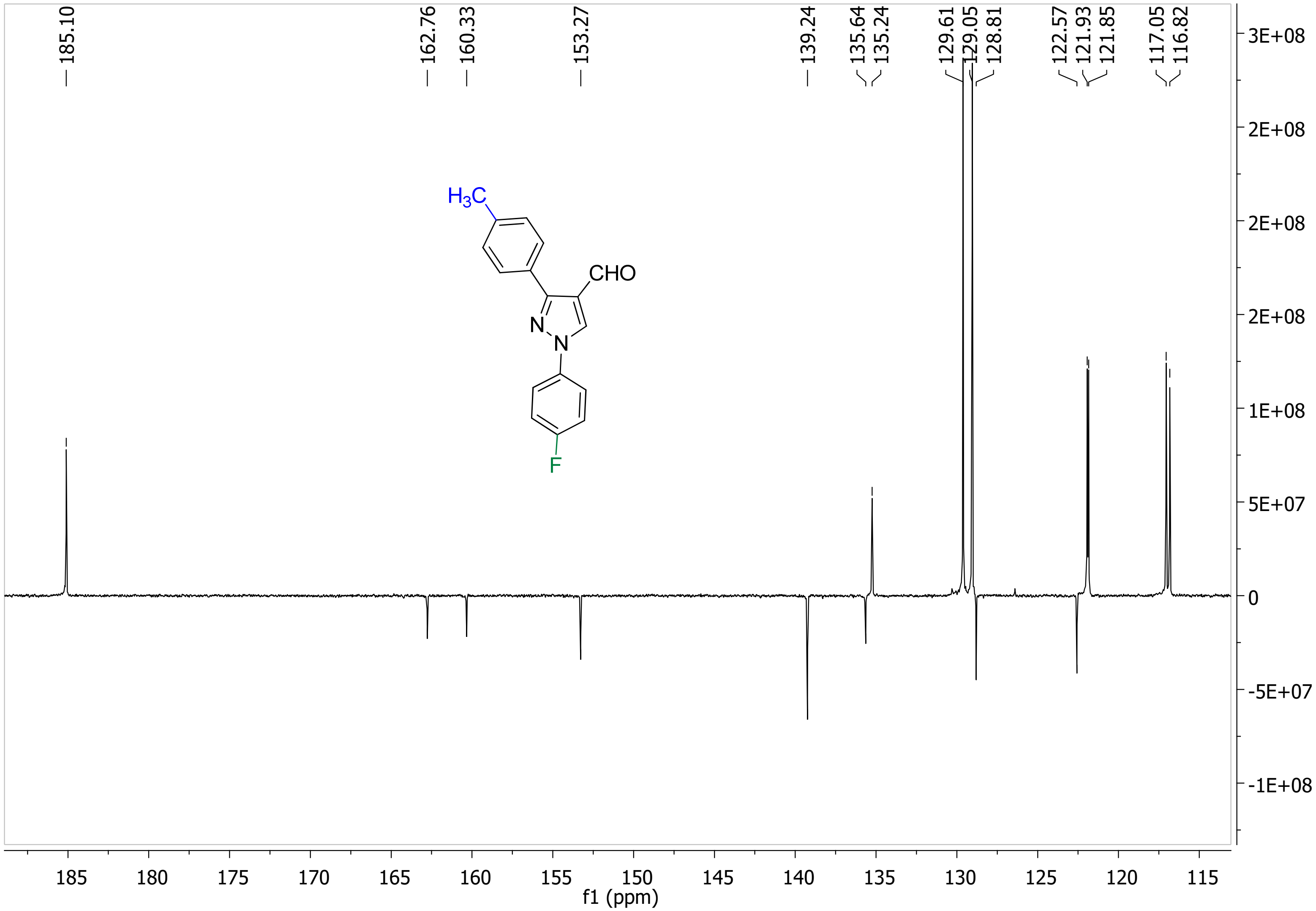

^{13}C APT NMR (DMSO-*d6*, 101 MHz) spectrum of 1-(4-fluorophenyl)-3-(*p*-tolyl)-1*H*-pyrazole-4-carbaldehyde **2m**

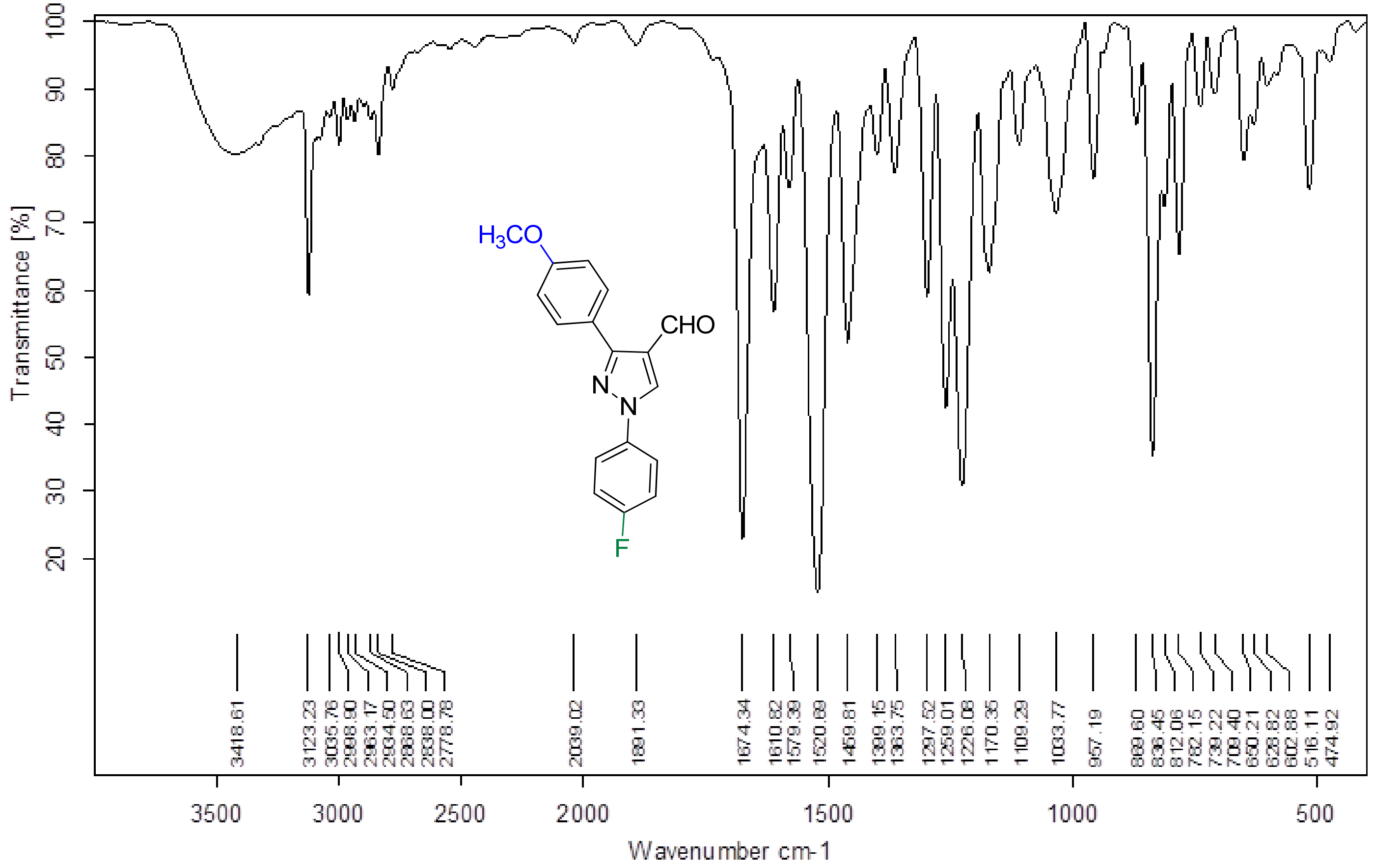

IR (KBr) spectrum of 1-(4-fluorophenyl)-3-(4-methoxyphenyl)-1*H*-pyrazole-4-carbaldehyde **2n**

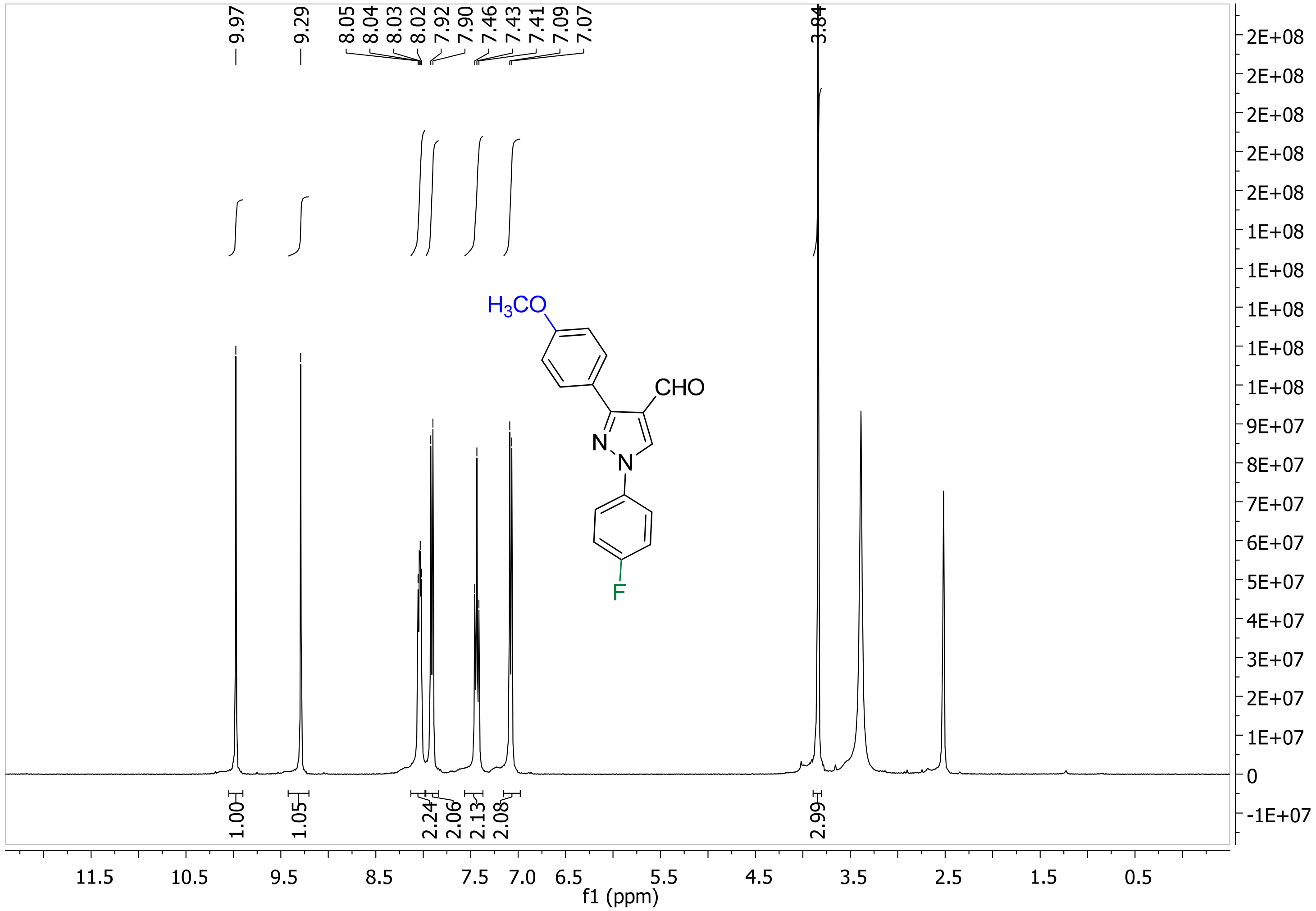

^{1}H NMR (DMSO-*d6*, 400 MHz) spectrum of 1-(4-fluorophenyl)-3-(4-methoxyphenyl)-1*H*-pyrazole-4-carbaldehyde **2n**

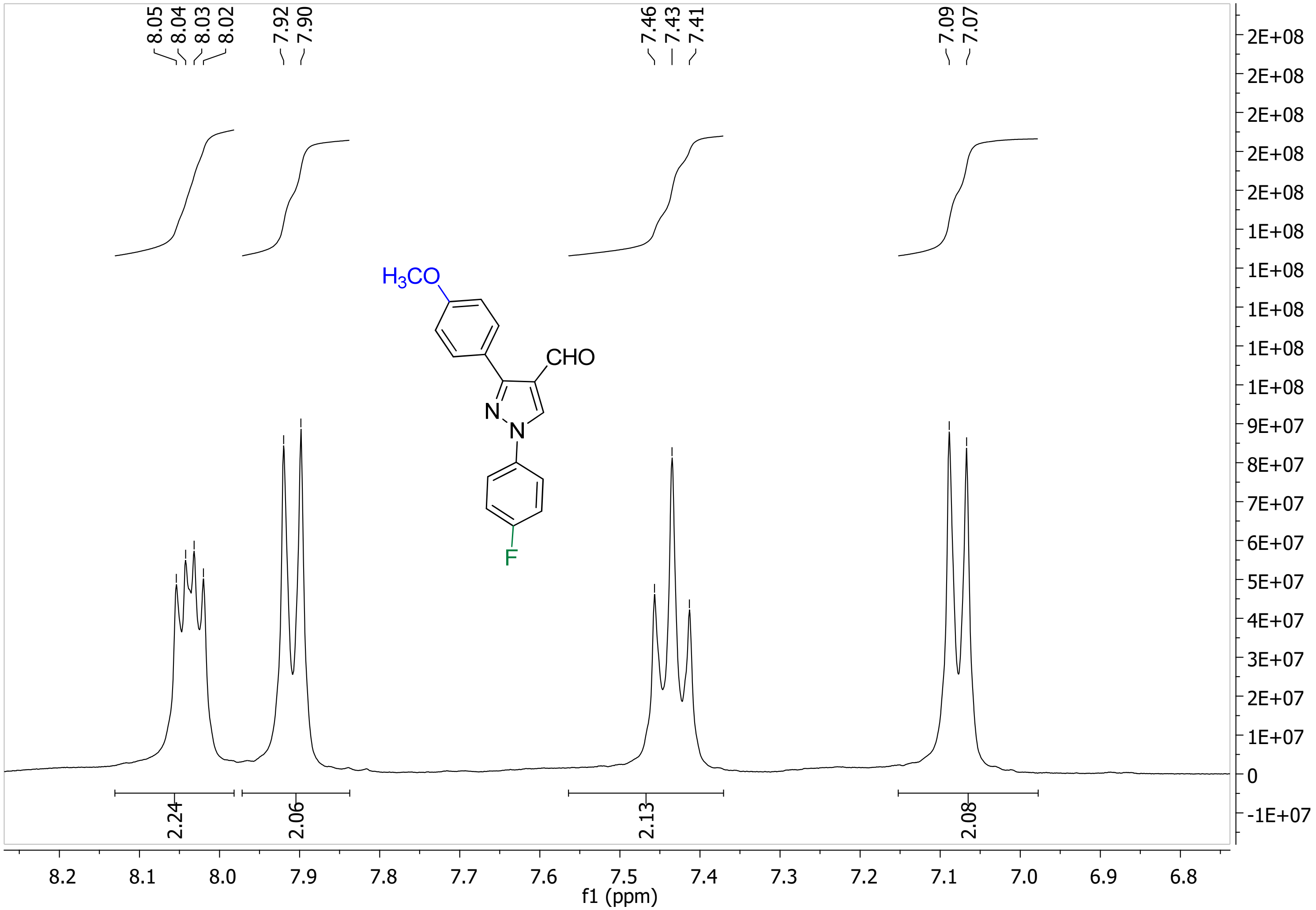

^{1}H NMR (DMSO-*d6*, 400 MHz) spectrum of 1-(4-fluorophenyl)-3-(4-methoxyphenyl)-1*H*-pyrazole-4-carbaldehyde **2n**

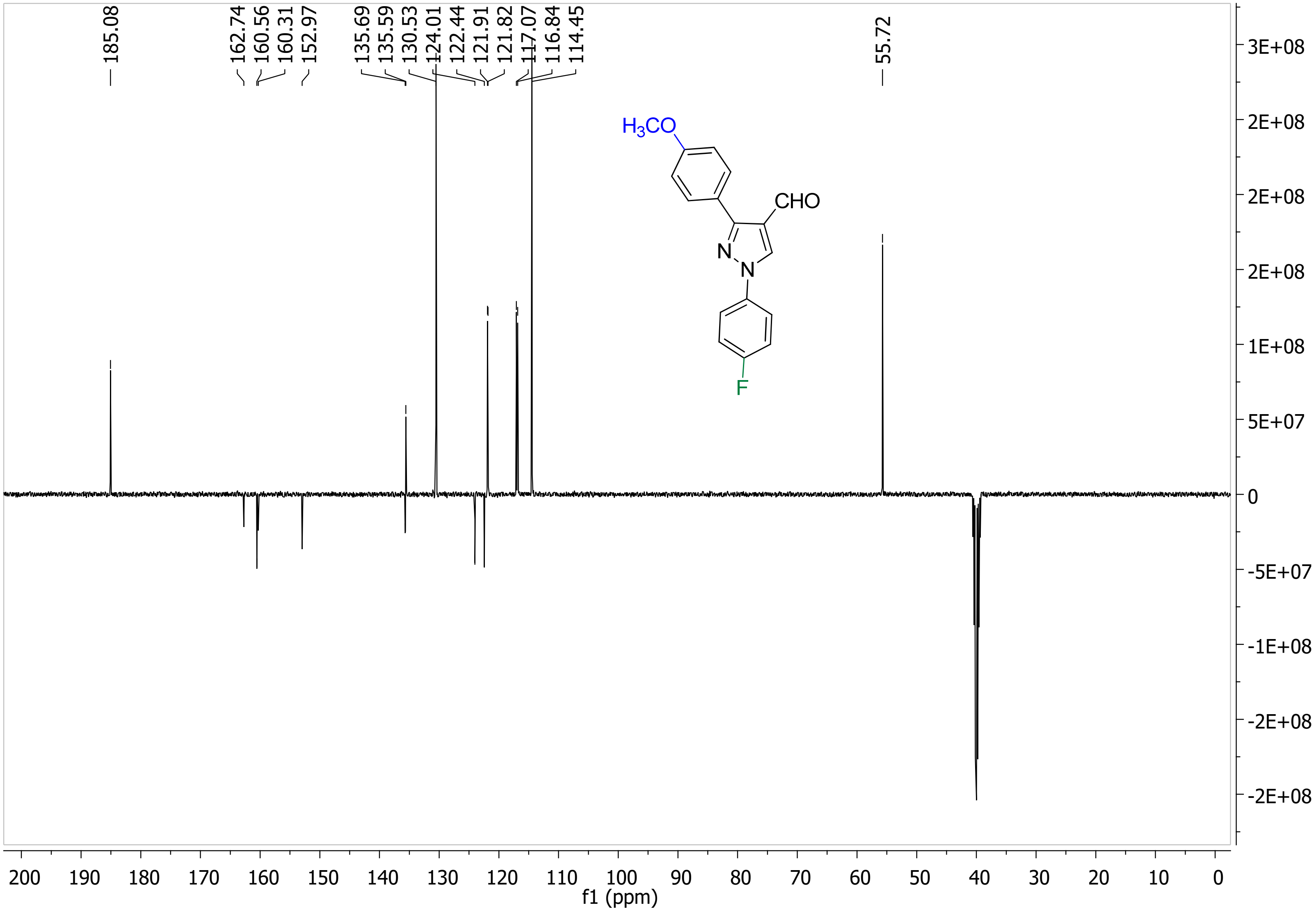

^{13}C APT NMR (DMSO-*d6*, 101 MHz) spectrum of 1-(4-fluorophenyl)-3-(4-methoxyphenyl)-1*H*-pyrazole-4-carbaldehyde **2n**

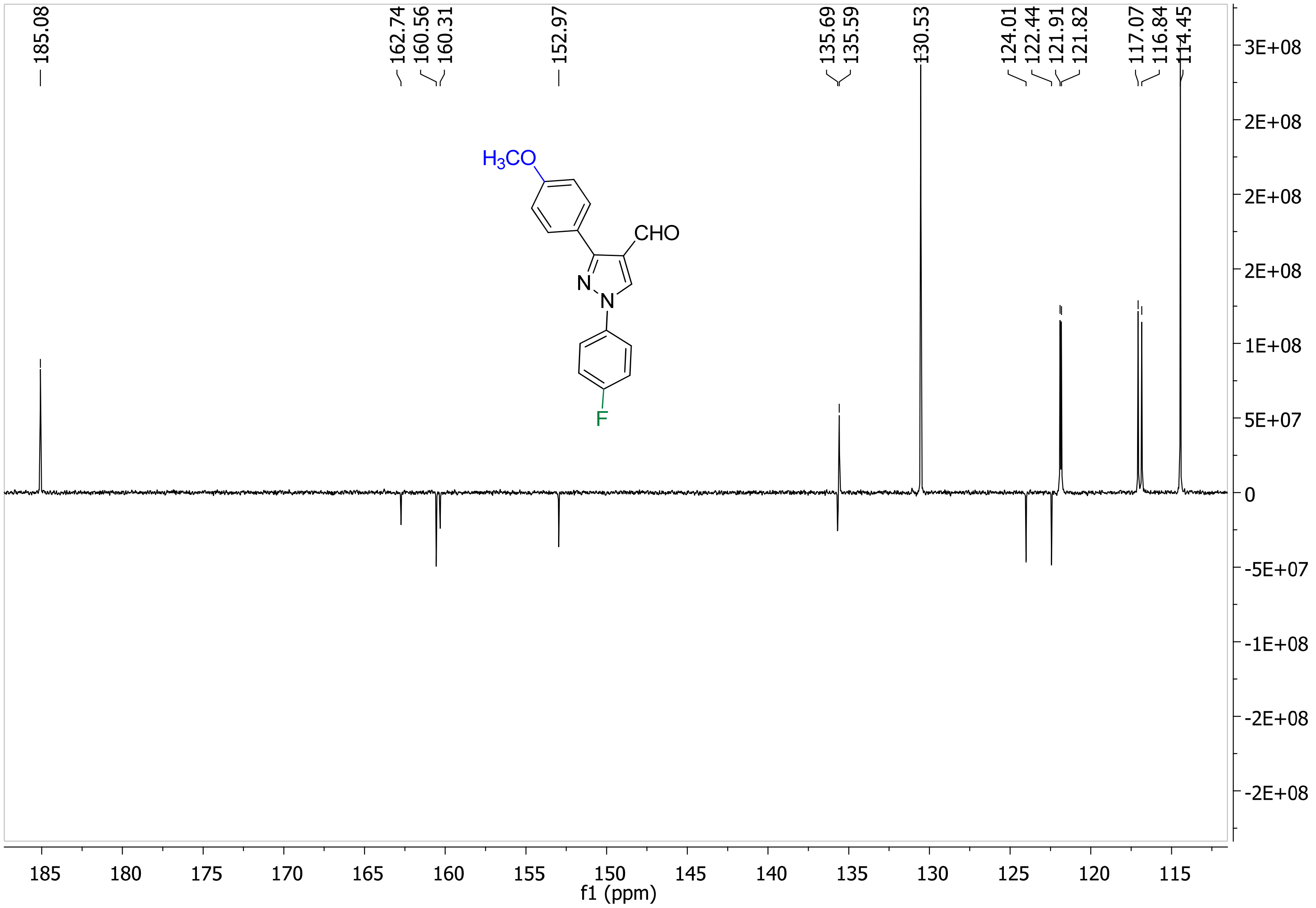

^{13}C APT NMR (DMSO-*d6*, 101 MHz) spectrum of 1-(4-fluorophenyl)-3-(4-methoxyphenyl)-1*H*-pyrazole-4-carbaldehyde **2n**

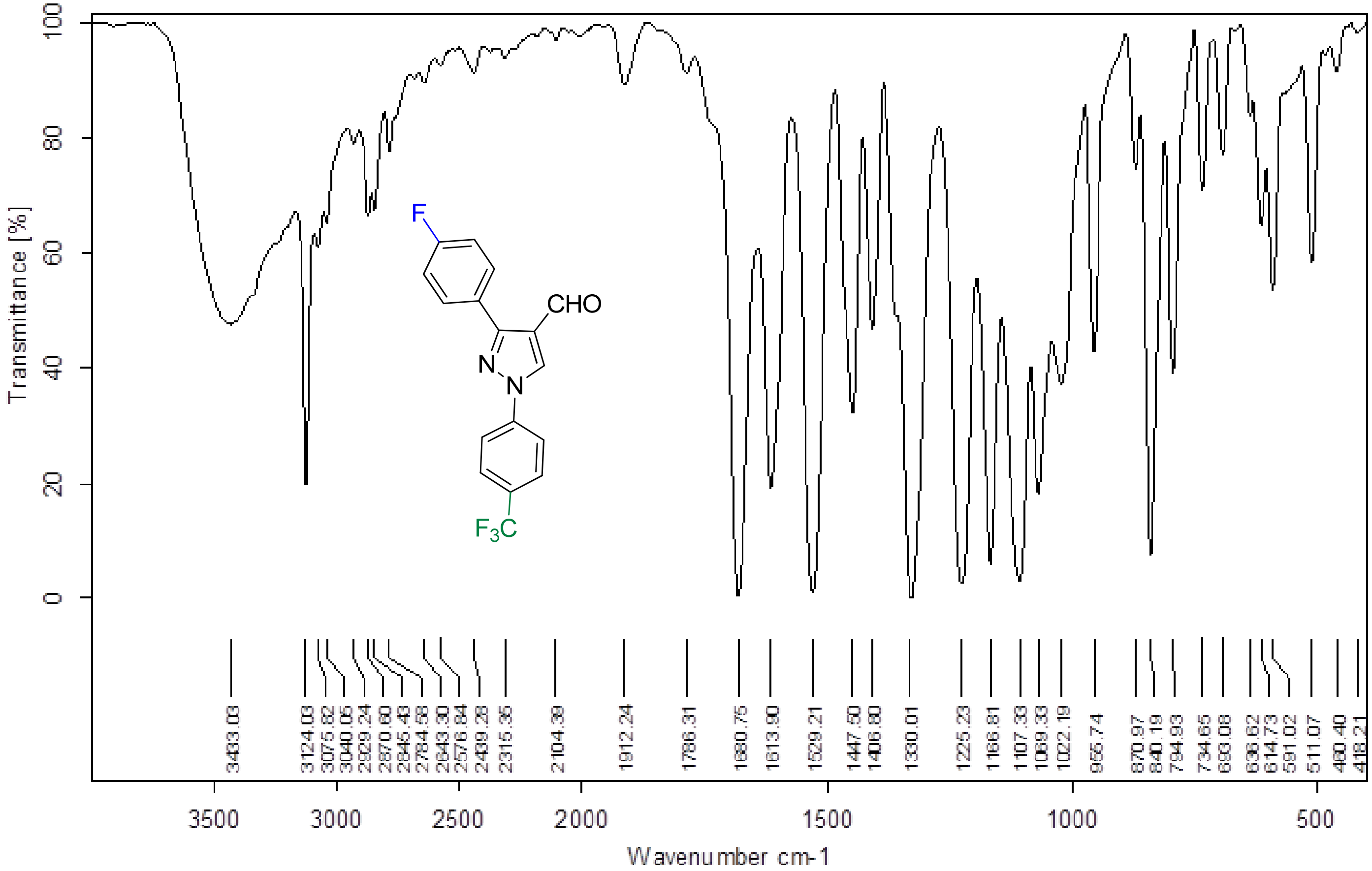

IR (KBr) spectrum of 3-(4-fluorophenyl)-1-(4-(trifluoromethyl)phenyl)-1*H*-pyrazole-4-carbaldehyde **2o**

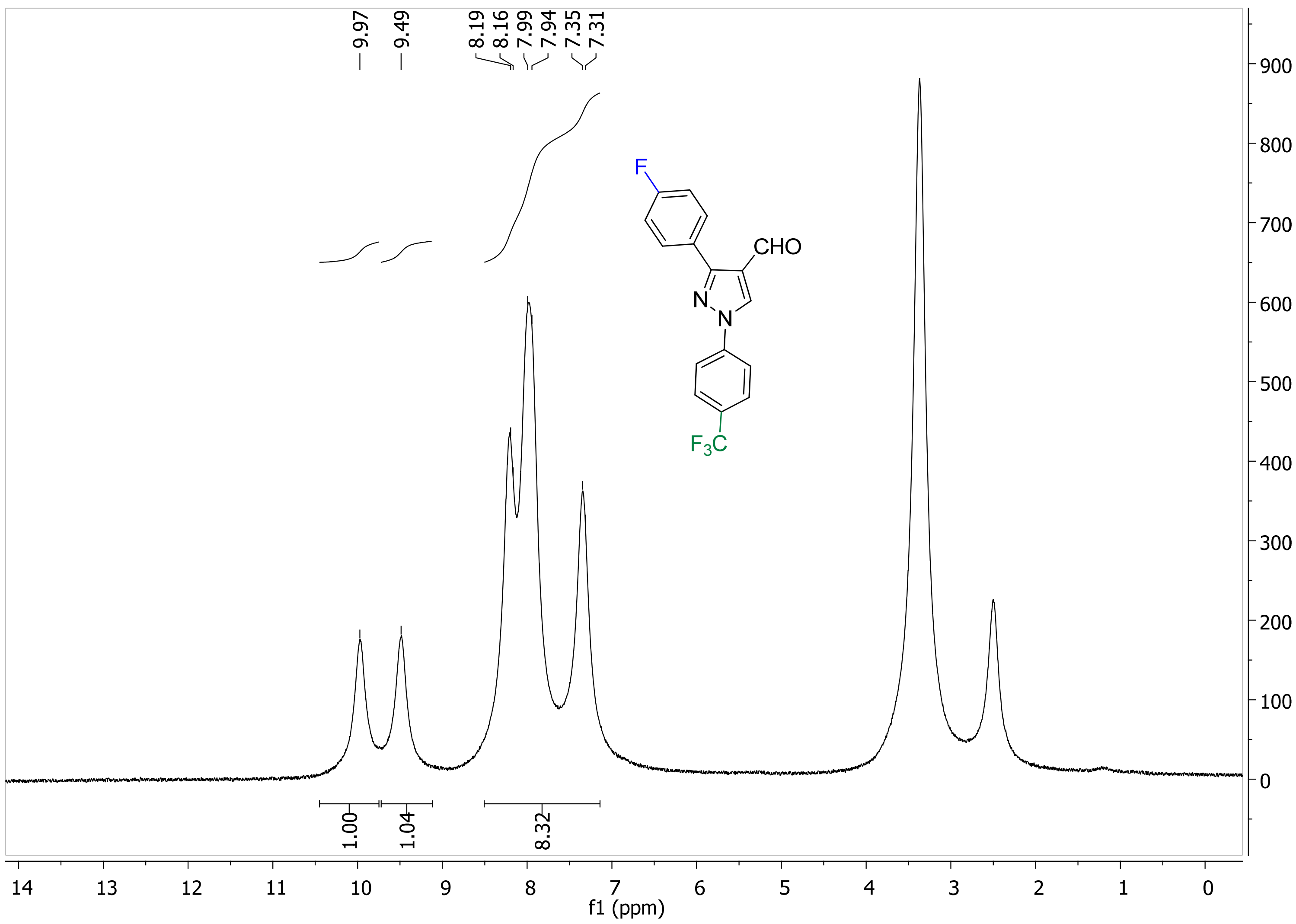

^{1}H NMR (DMSO-*d6*, 400 MHz) spectrum of 3-(4-fluorophenyl)-1-(4-(trifluoromethyl)phenyl)-1*H*-pyrazole-4-carbaldehyde **2o**

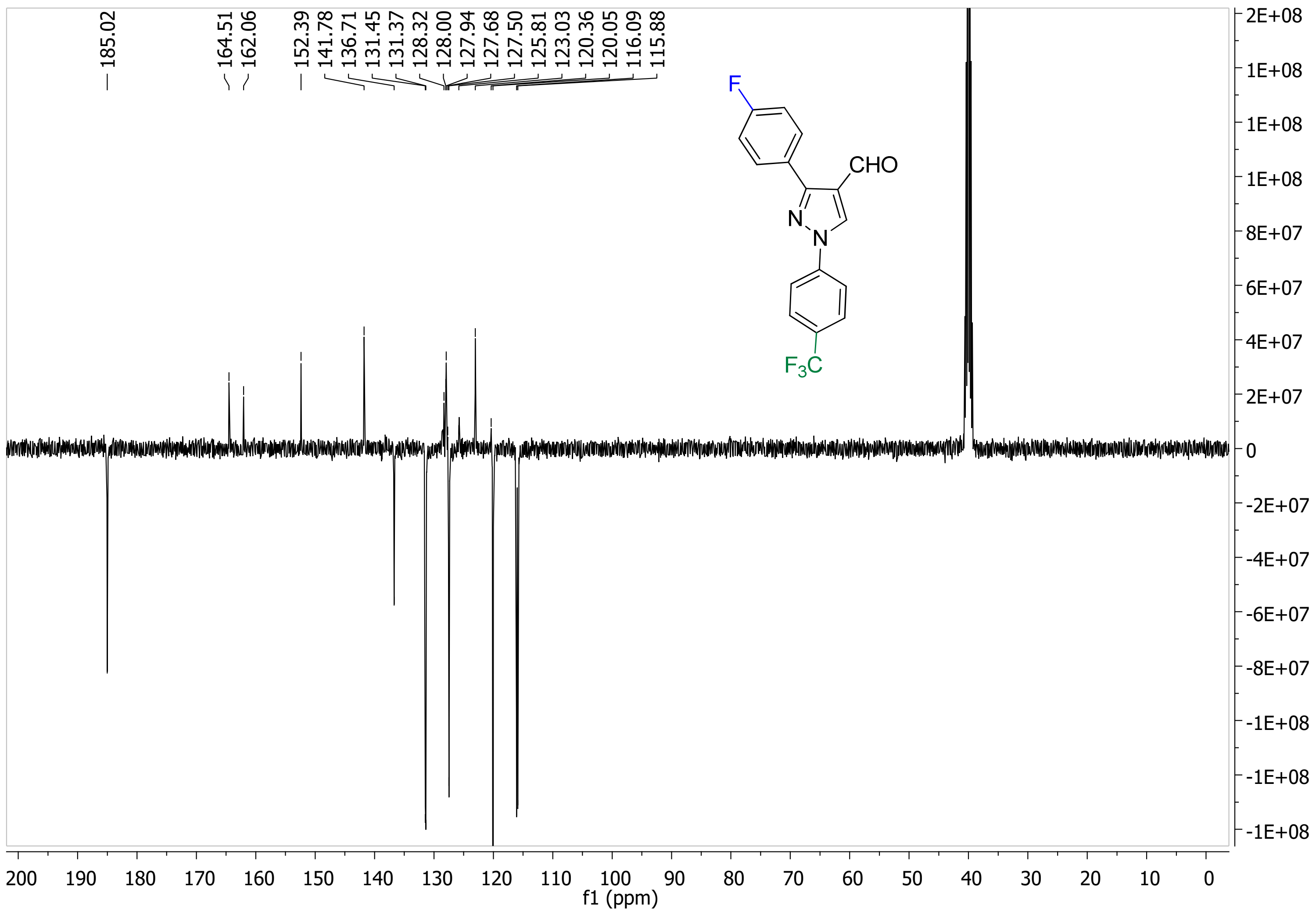

^{13}C APT NMR (DMSO-*d6*, 101 MHz) spectrum of 3-(4-fluorophenyl)-1-(4-(trifluoromethyl)phenyl)-1*H*-pyrazole-4-carbaldehyde **2o**

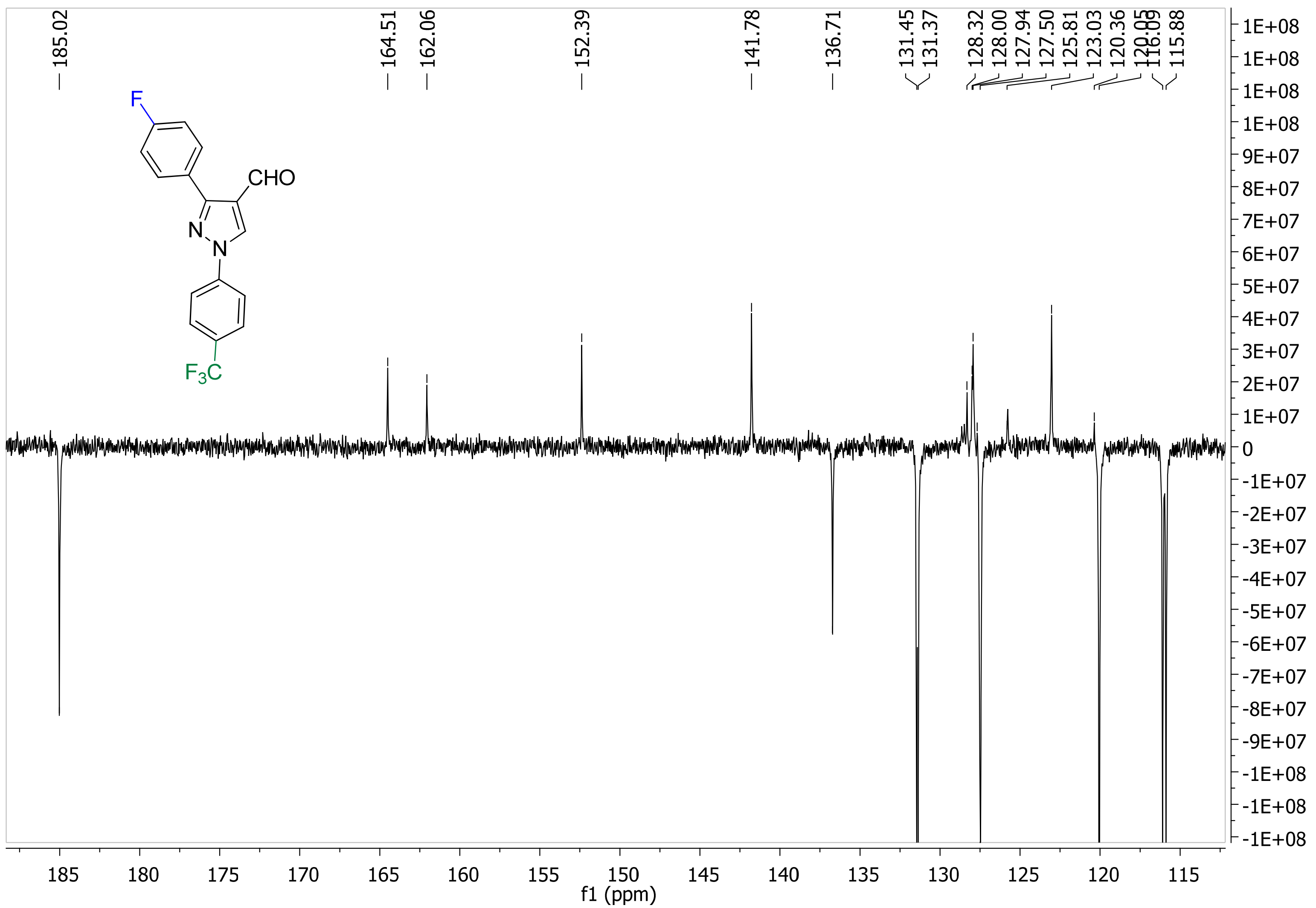

^{13}C APT NMR (DMSO-*d6*, 101 MHz) spectrum of 3-(4-fluorophenyl)-1-(4-(trifluoromethyl)phenyl)-1*H*-pyrazole-4-carbaldehyde **2o**

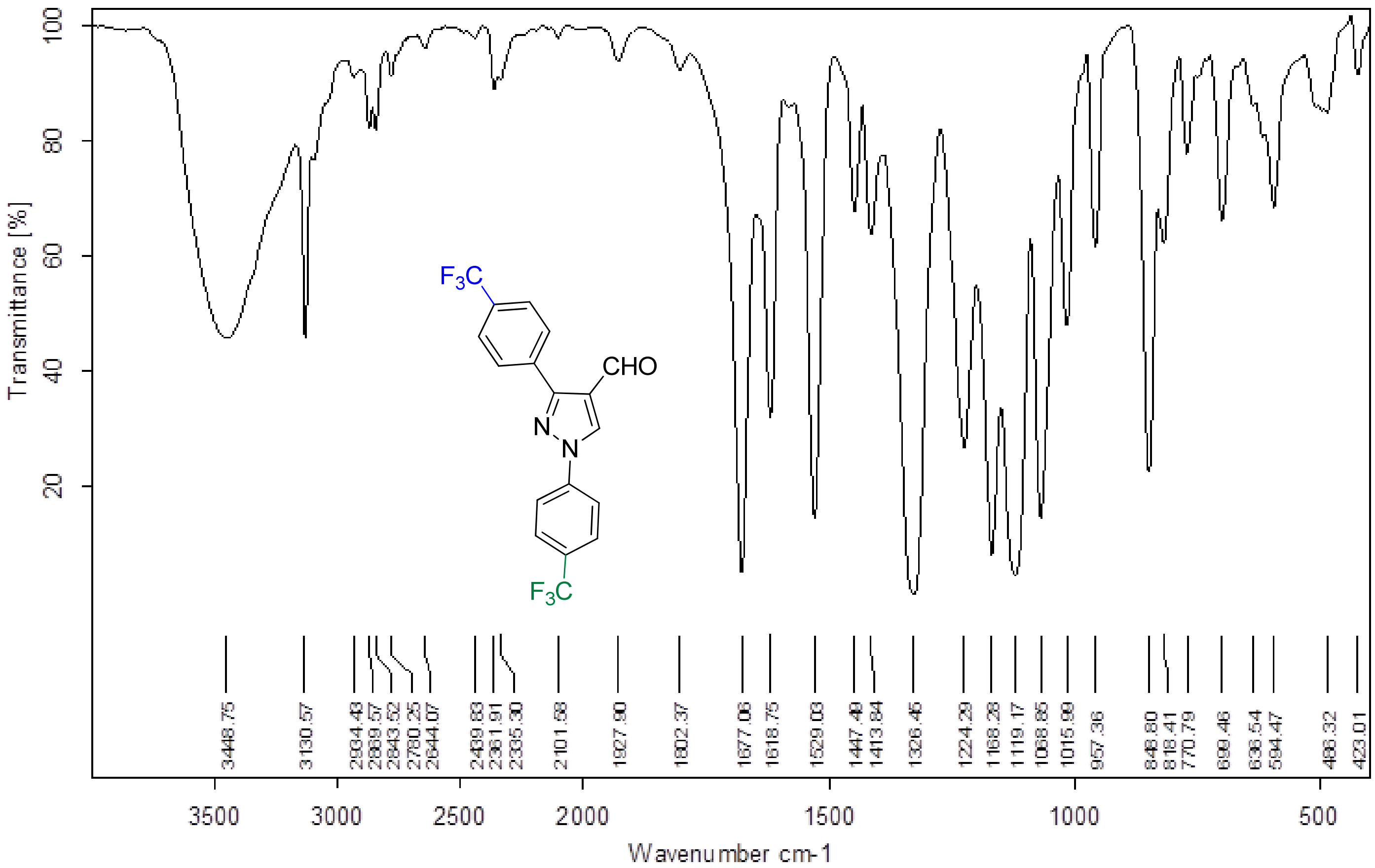

IR (KBr) spectrum of 1,3-bis(4-(trifluoromethyl)phenyl)-1*H*-pyrazole-4-carbaldehyde **2p**

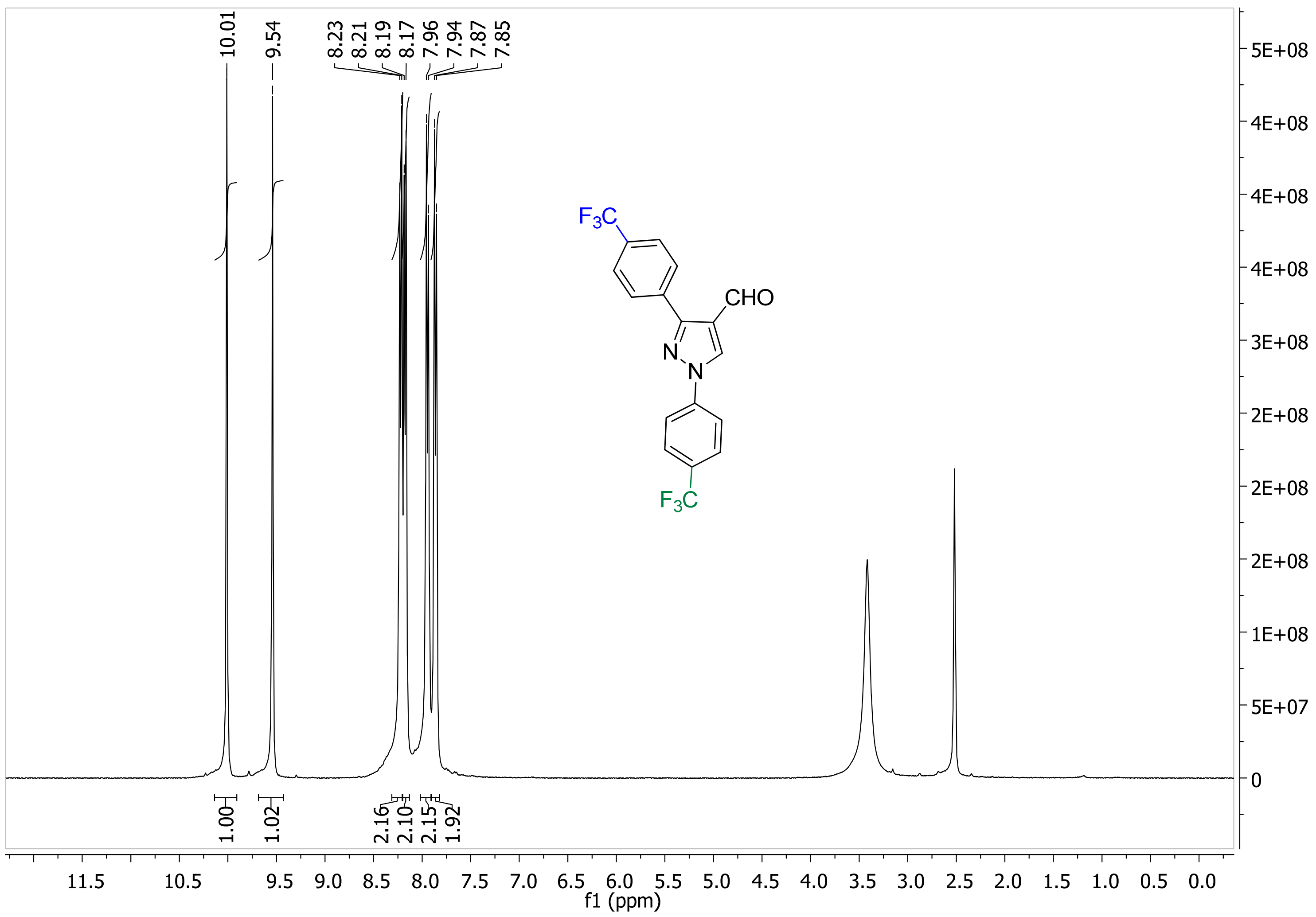

^{1}H NMR (DMSO-*d6*, 400 MHz) spectrum of 1,3-bis(4-(trifluoromethyl)phenyl)-1*H*-pyrazole-4-carbaldehyde **2p**

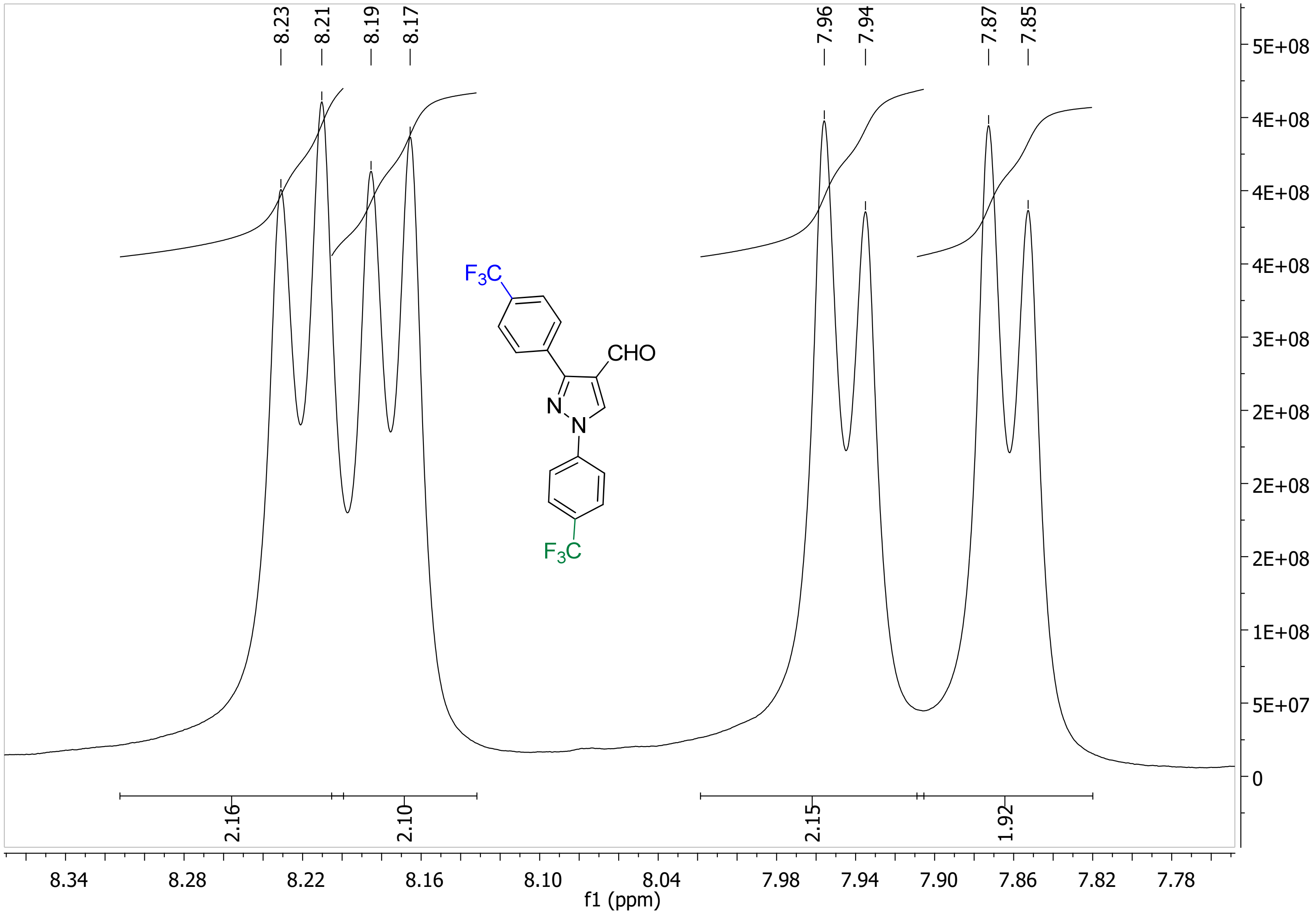

^{1}H NMR (DMSO-*d6*, 400 MHz) spectrum of 1,3-bis(4-(trifluoromethyl)phenyl)-1*H*-pyrazole-4-carbaldehyde **2p**

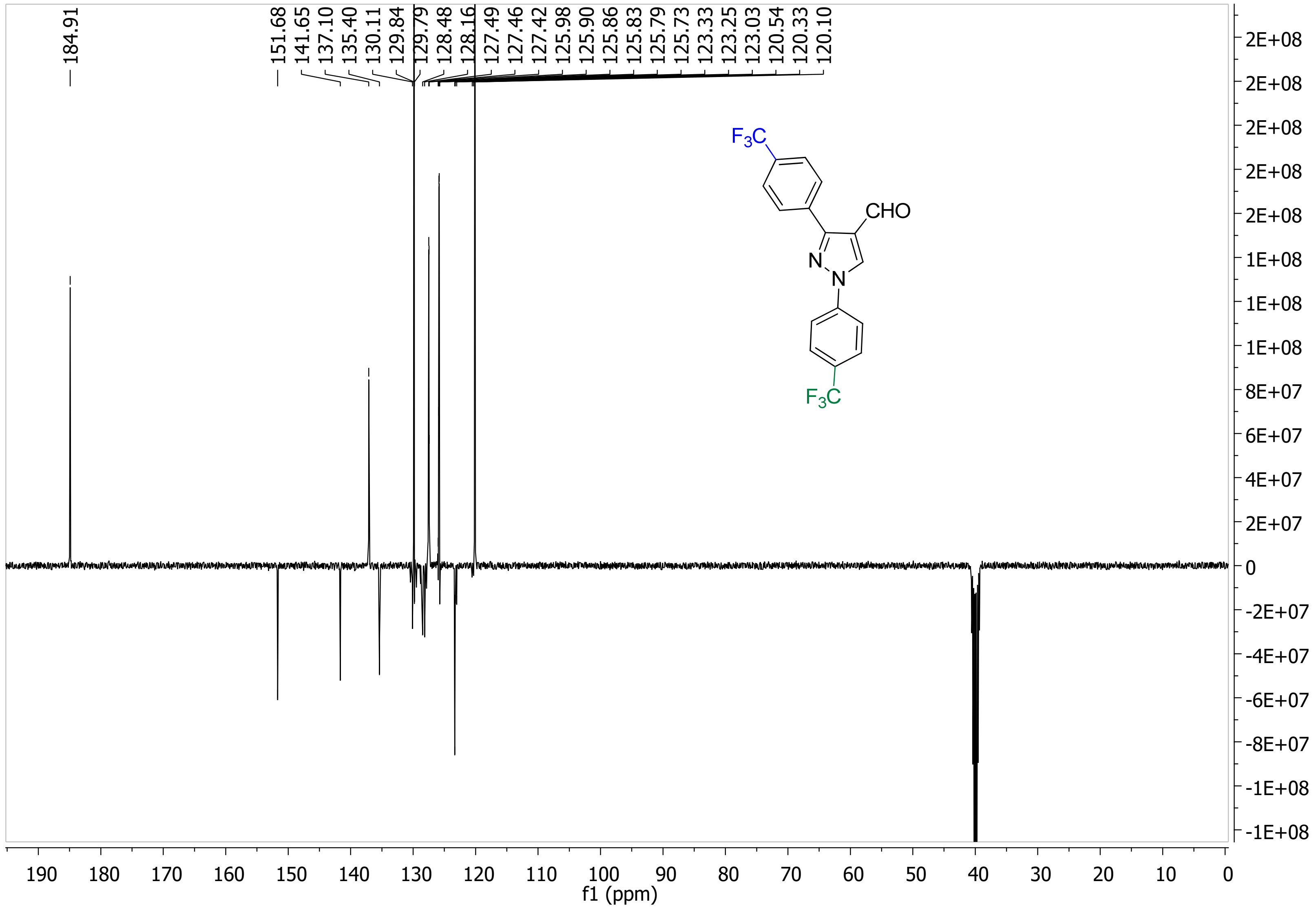

^{13}C APT NMR (DMSO-*d6*, 101 MHz) spectrum of 1,3-bis(4-(trifluoromethyl)phenyl)-1*H*-pyrazole-4-carbaldehyde **2p**

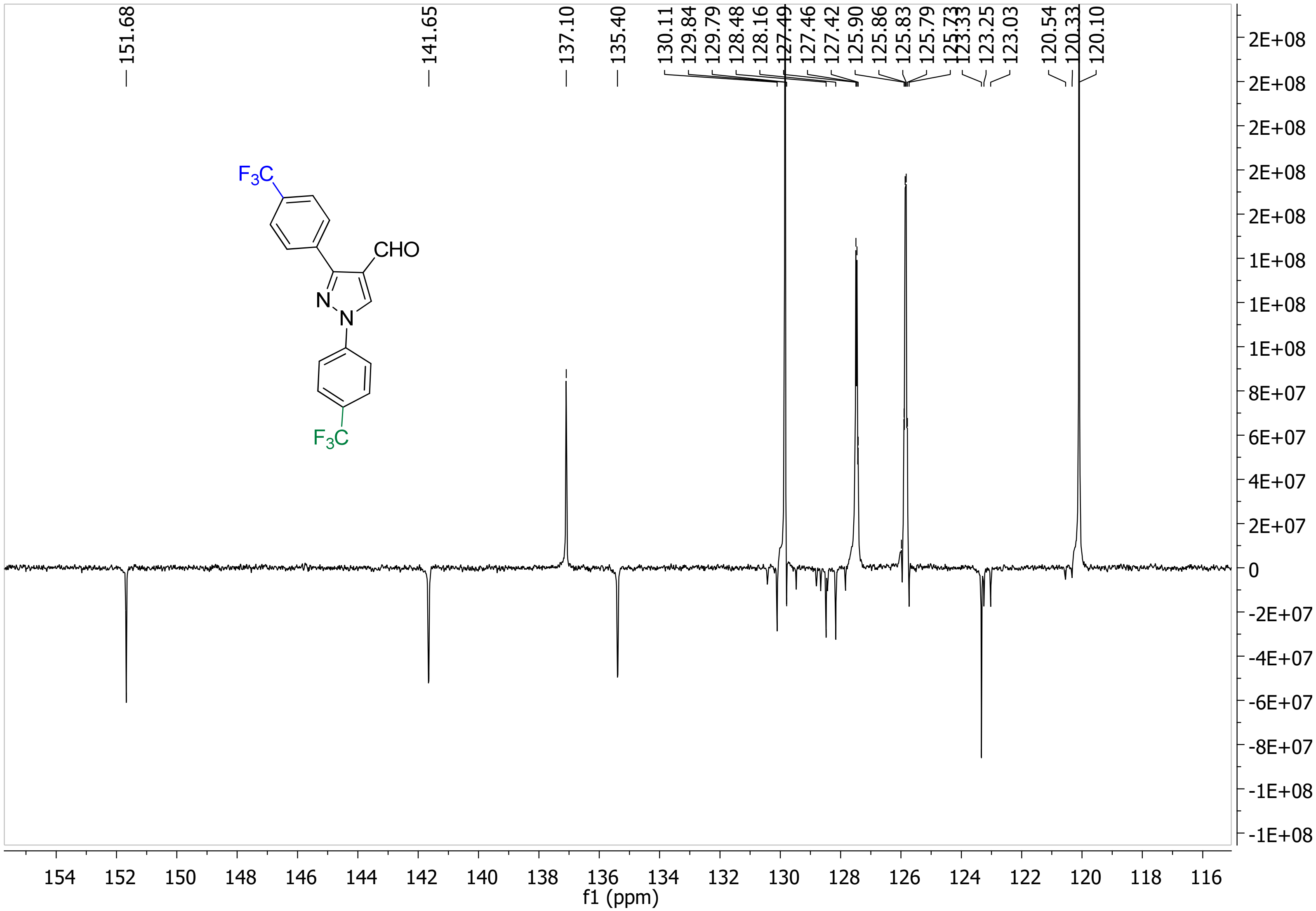

^{13}C APT NMR (DMSO-*d6*, 101 MHz) spectrum of 1,3-bis(4-(trifluoromethyl)phenyl)-1*H*-pyrazole-4-carbaldehyde **2p**

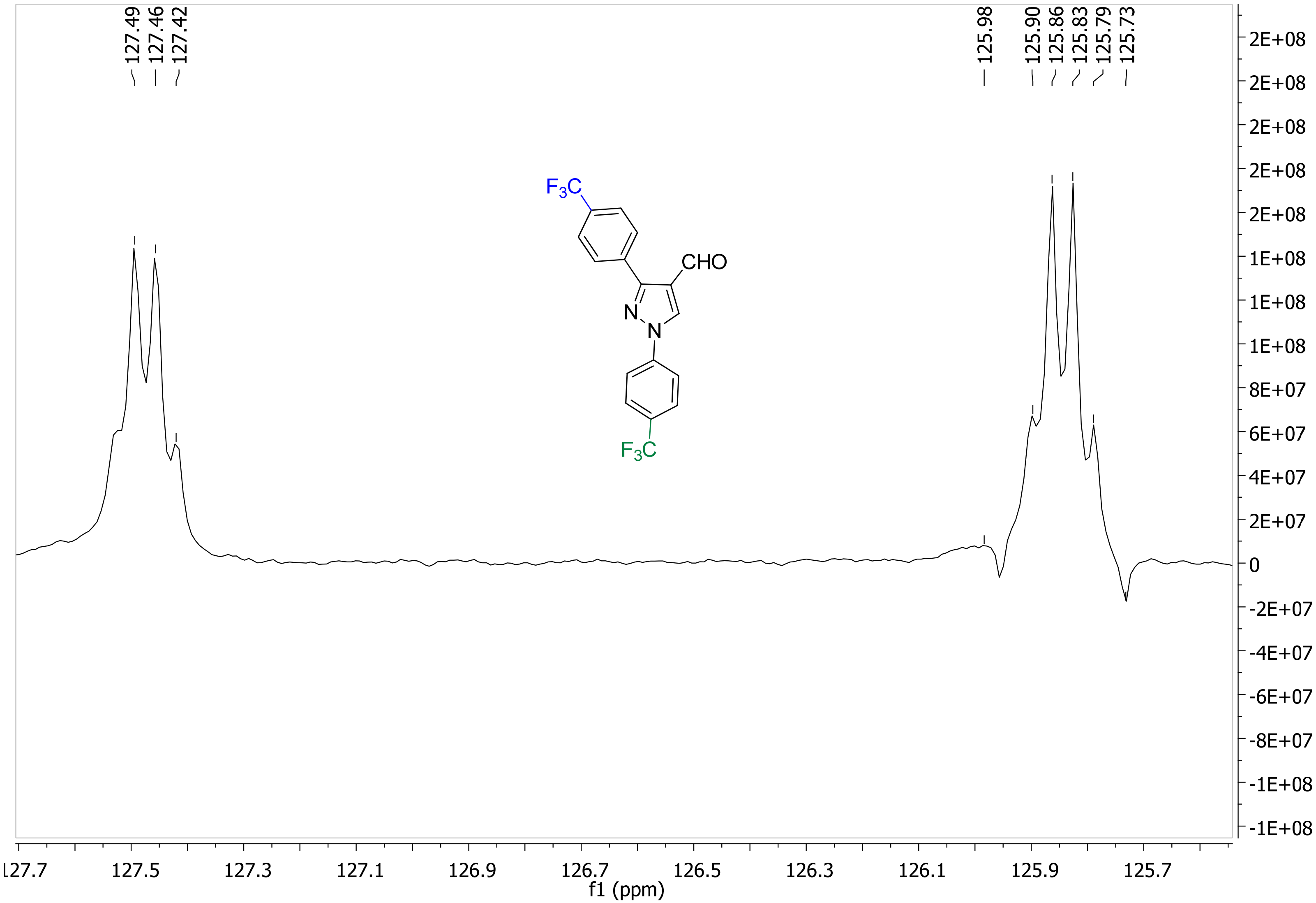

^{13}C APT NMR (DMSO-*d6*, 101 MHz) spectrum of 1,3-bis(4-(trifluoromethyl)phenyl)-1*H*-pyrazole-4-carbaldehyde **2p**

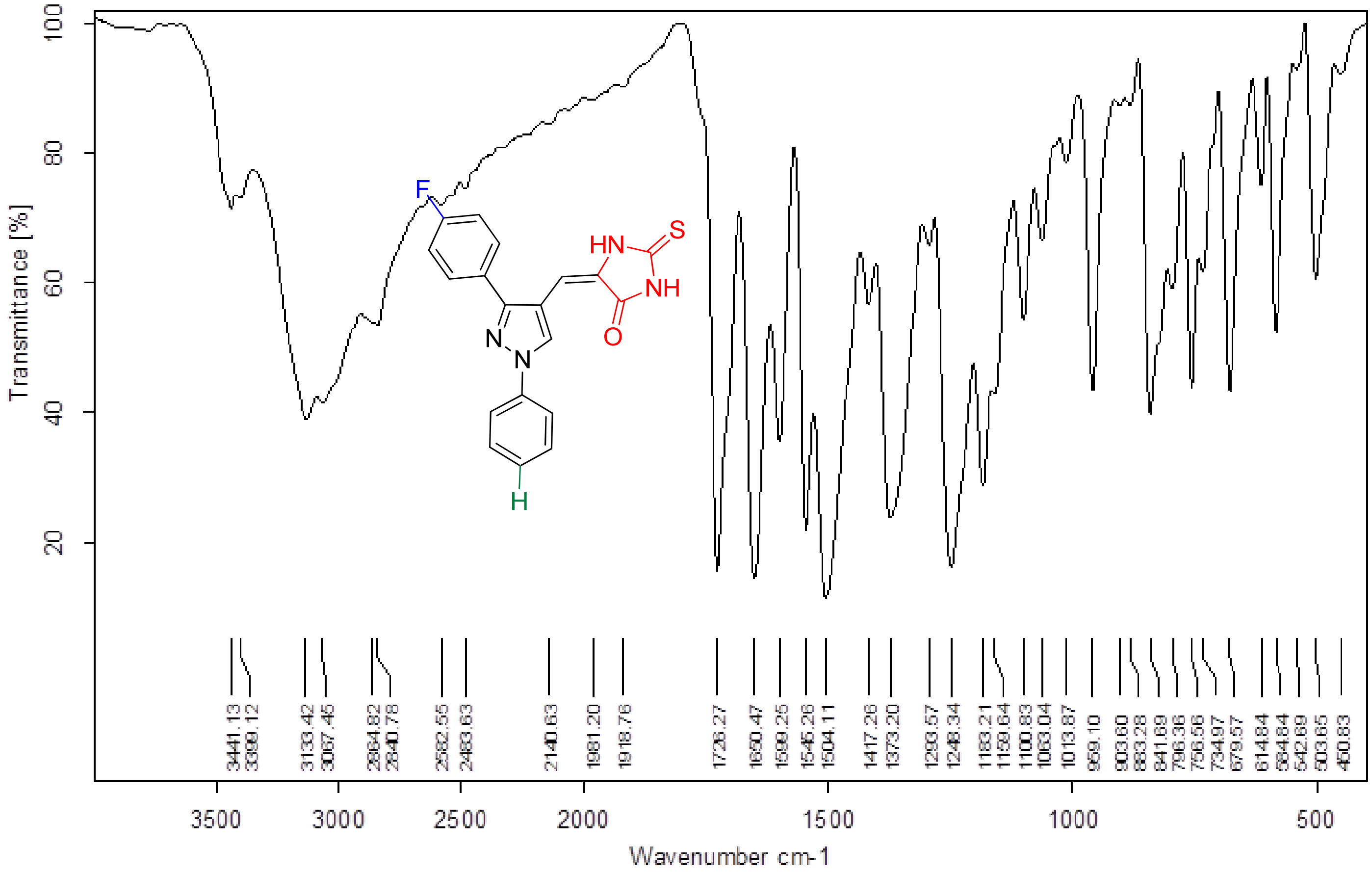

IR (KBr) spectrum of 5-((3-(4-fluorophenyl)-1-phenyl-1*H*-pyrazol-4-yl)methylene)-2-thioxoimidazolidin-4-one **3a**

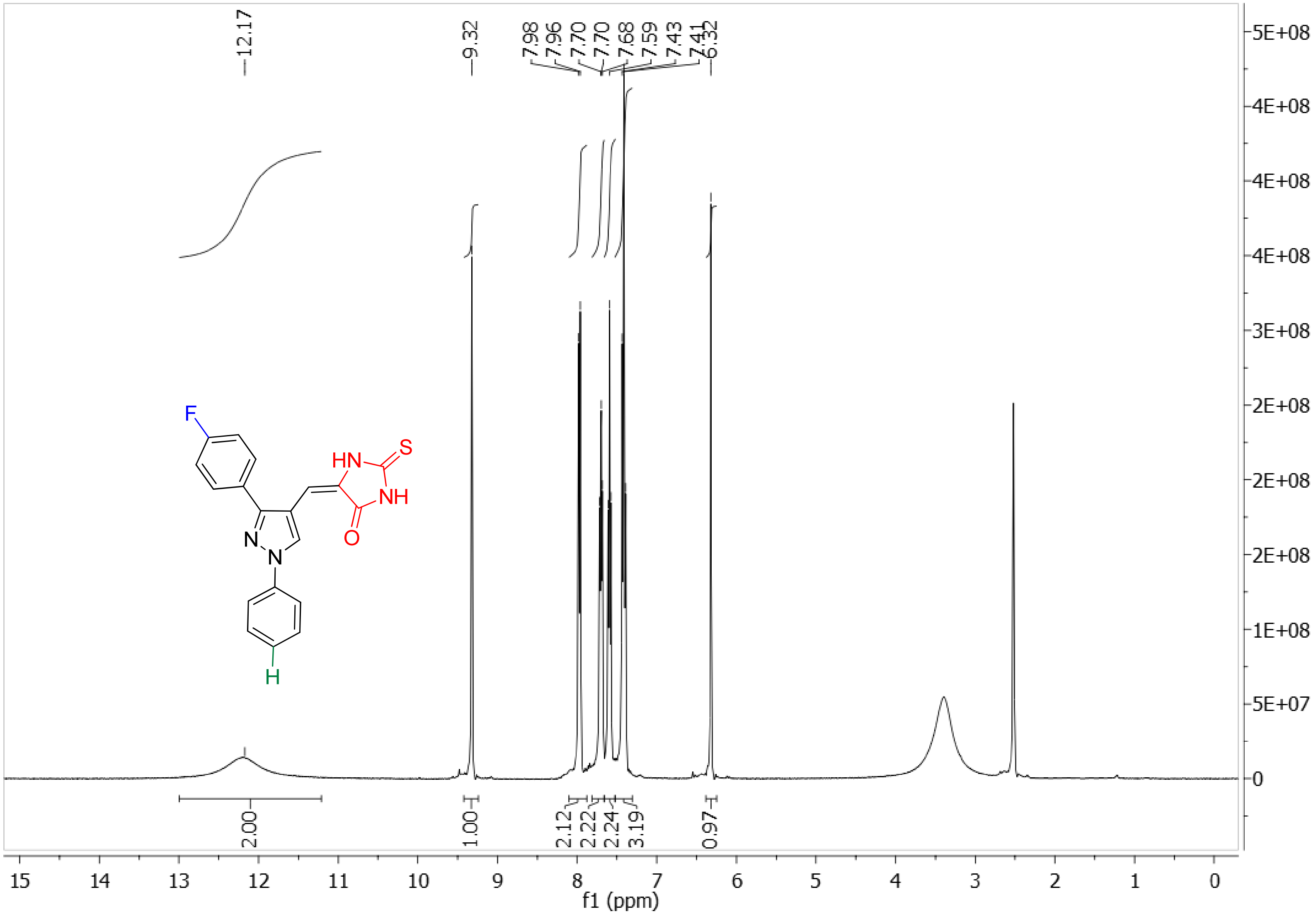

^{1}H NMR (DMSO-*d6*, 400 MHz) spectrum of 5-((3-(4-fluorophenyl)-1-phenyl-1*H*-pyrazol-4-yl)methylene)-2-thioxoimidazolidin-4-one **3a**

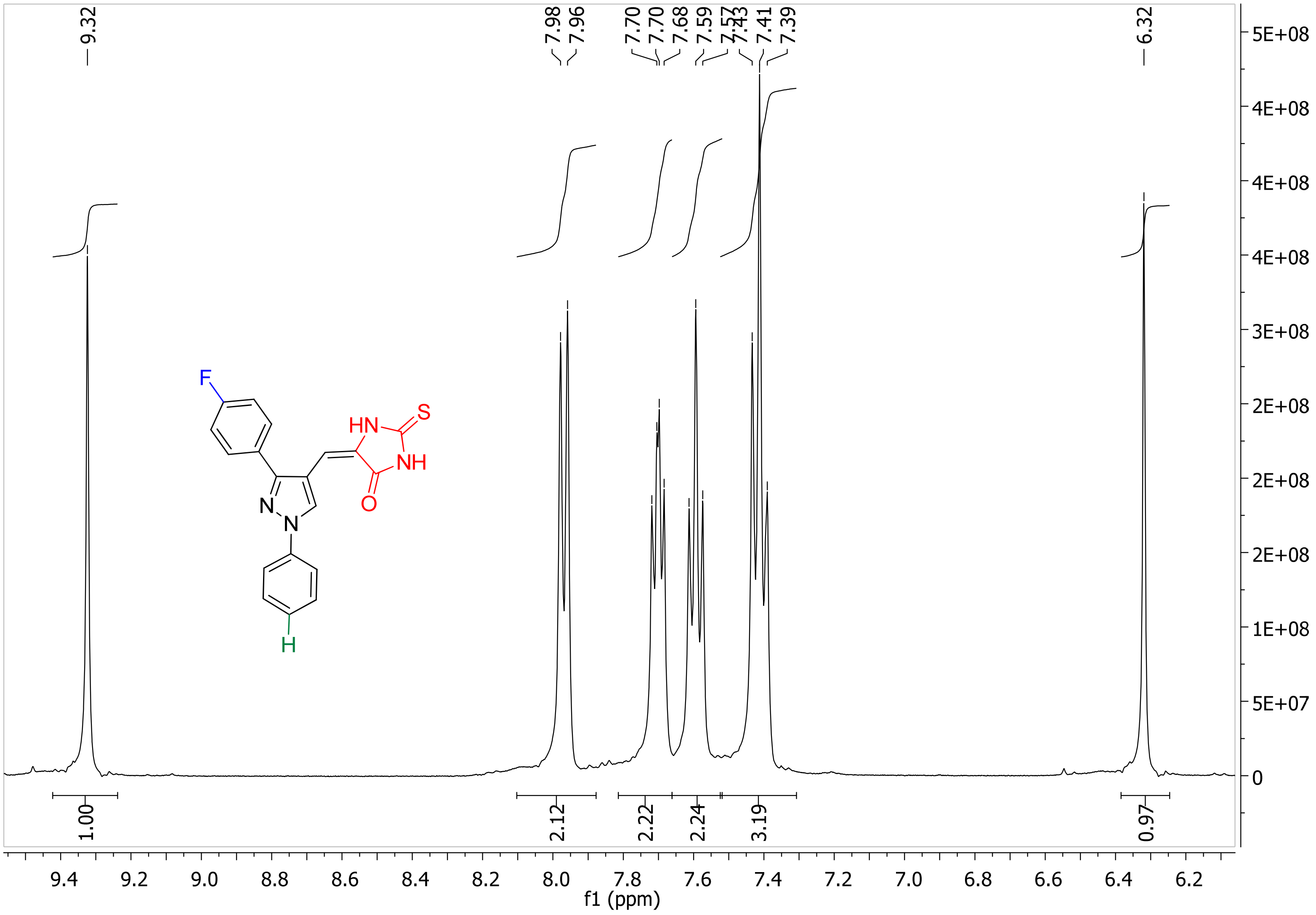

^{1}H NMR (DMSO-*d6*, 400 MHz) spectrum of 5-((3-(4-fluorophenyl)-1-phenyl-1*H*-pyrazol-4-yl)methylene)-2-thioxoimidazolidin-4-one **3a**

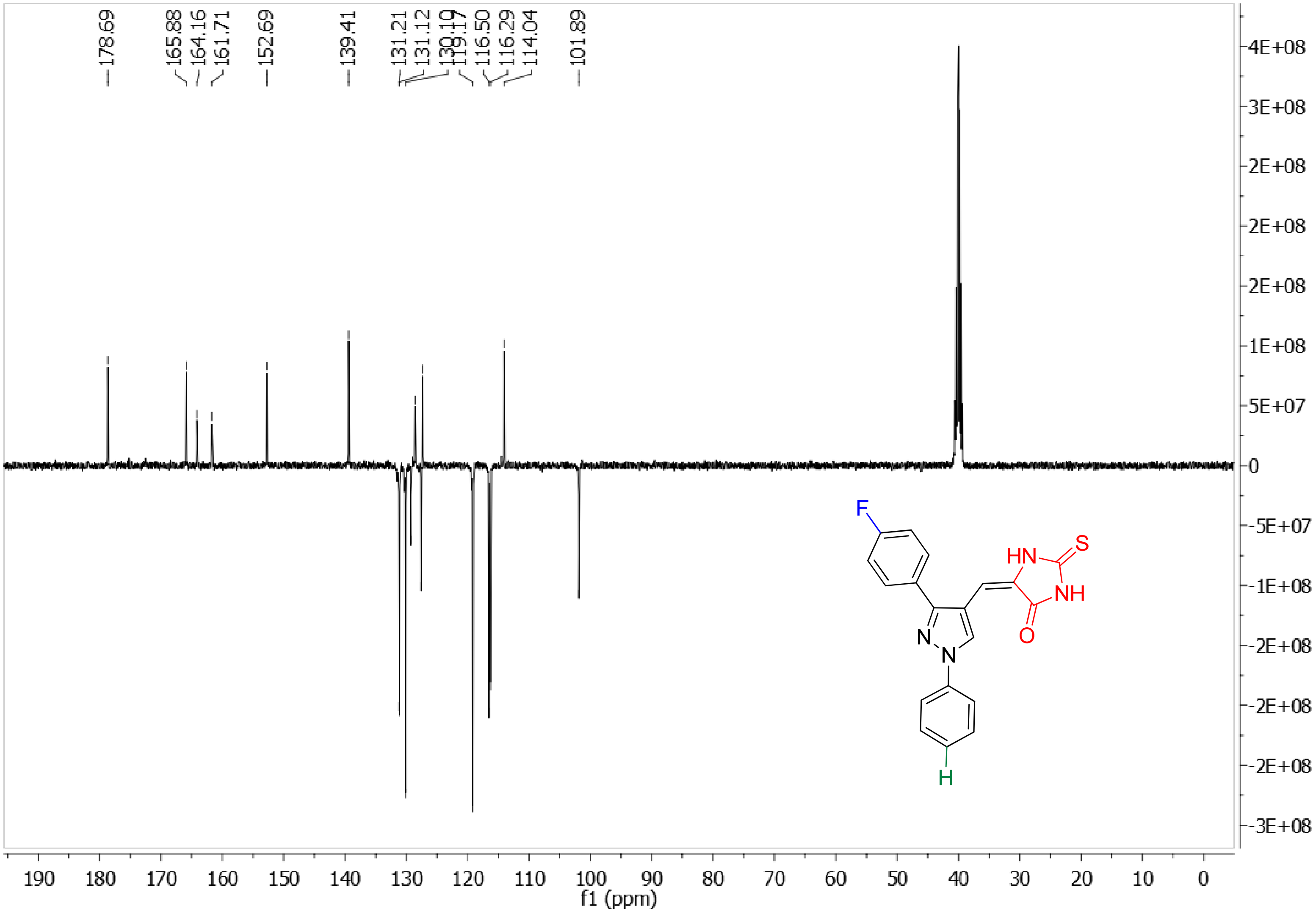

^{13}C APT NMR (DMSO-*d6*, 101 MHz) spectrum of 5-((3-(4-fluorophenyl)-1-phenyl-1*H*-pyrazol-4-yl)methylene)-2-thioxoimidazolidin-4-one **3a**

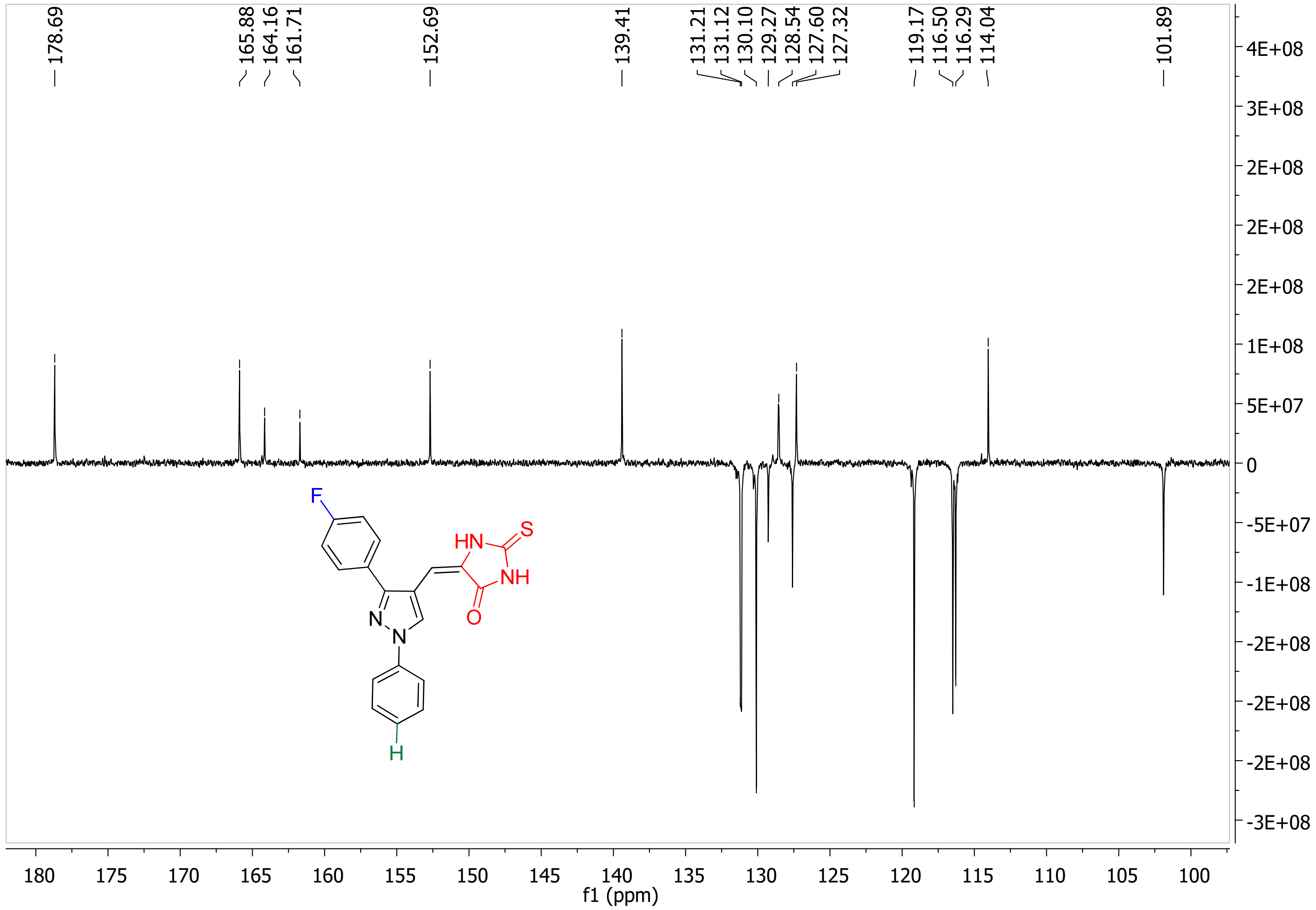

^{13}C APT NMR (DMSO-*d6*, 101 MHz) spectrum of 5-((3-(4-fluorophenyl)-1-phenyl-1*H*-pyrazol-4-yl)methylene)-2-thioxoimidazolidin-4-one **3a**

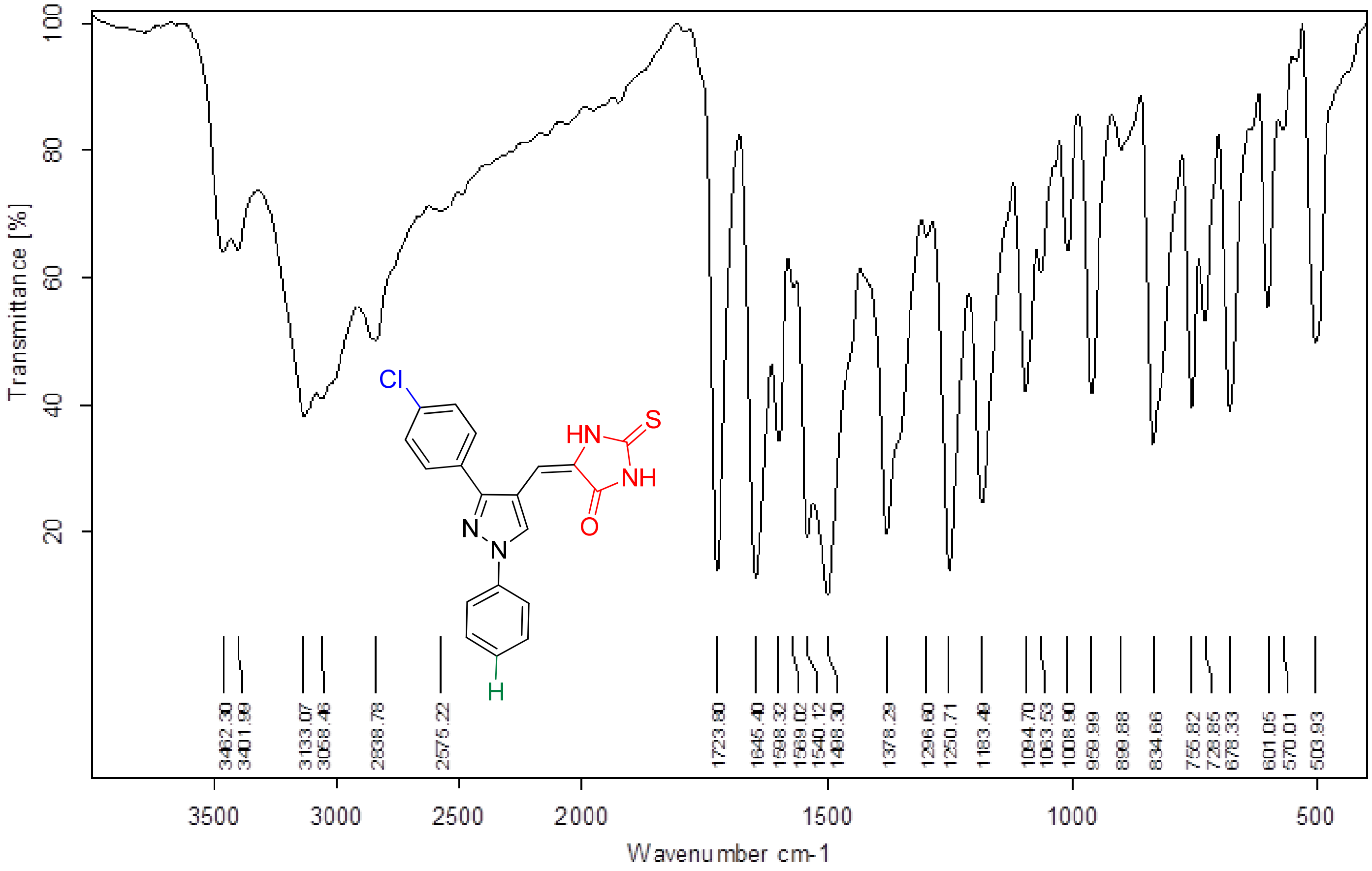

IR (KBr) spectrum of 5-((3-(4-chlorophenyl)-1-phenyl-1*H*-pyrazol-4-yl)methylene)-2-thioxoimidazolidin-4-one **3b**

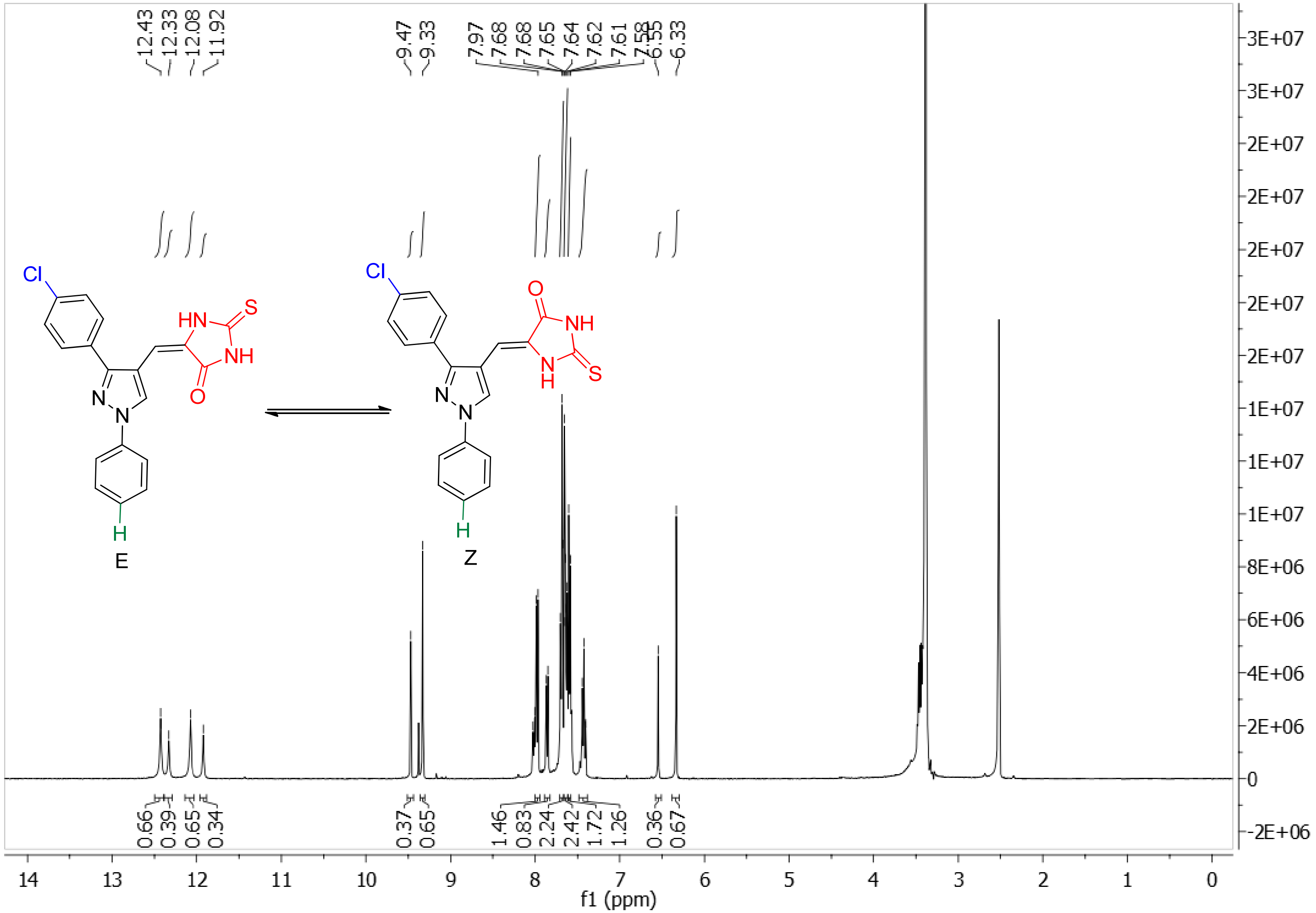

^{1}H NMR (DMSO-*d6*, 400 MHz) spectrum of 5-((3-(4-chlorophenyl)-1-phenyl-1*H*-pyrazol-4-yl)methylene)-2-thioxoimidazolidin-4-one **3b**

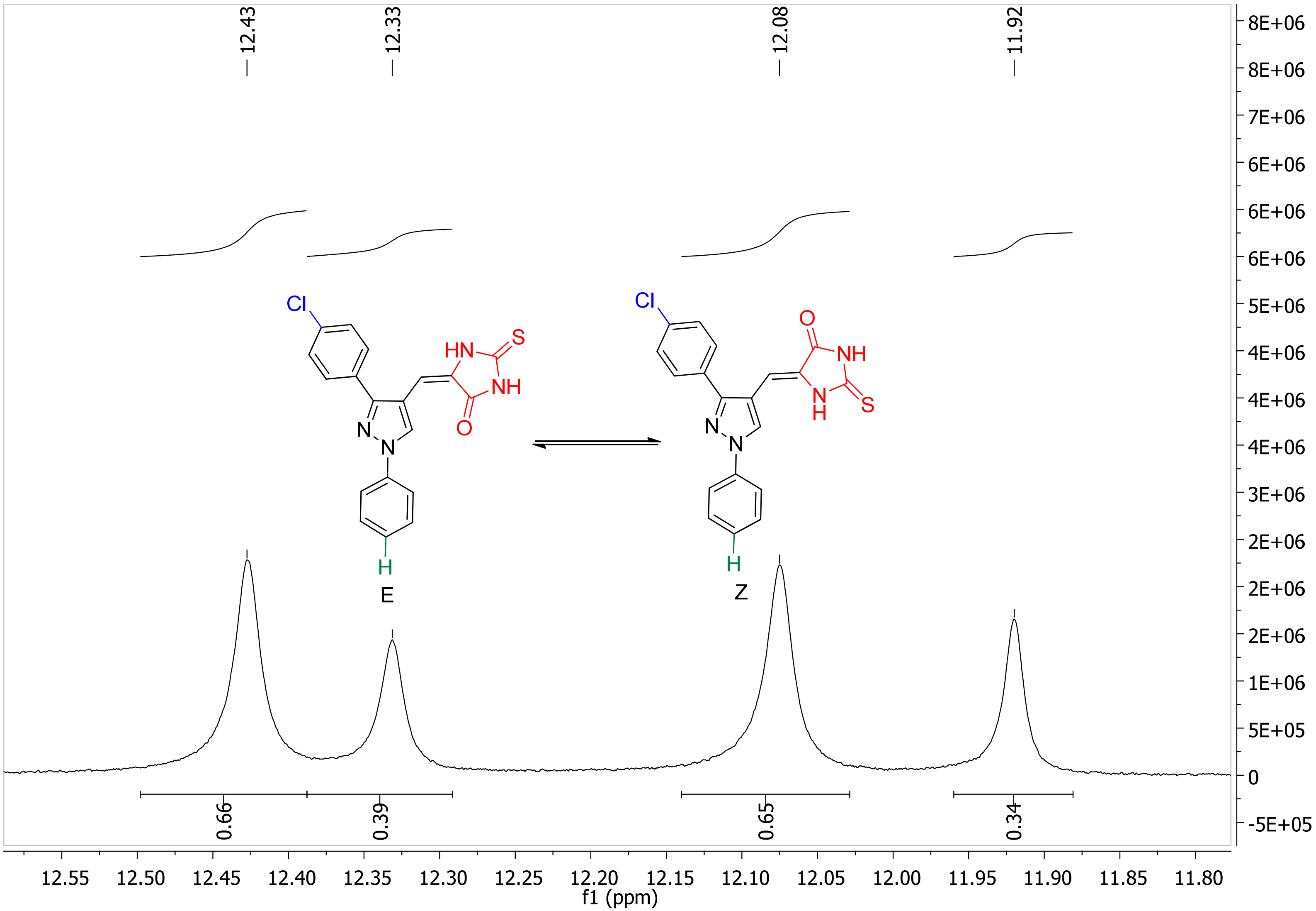

^{1}H NMR (DMSO-*d6*, 400 MHz) spectrum of 5-((3-(4-chlorophenyl)-1-phenyl-1*H*-pyrazol-4-yl)methylene)-2-thioxoimidazolidin-4-one **3b**

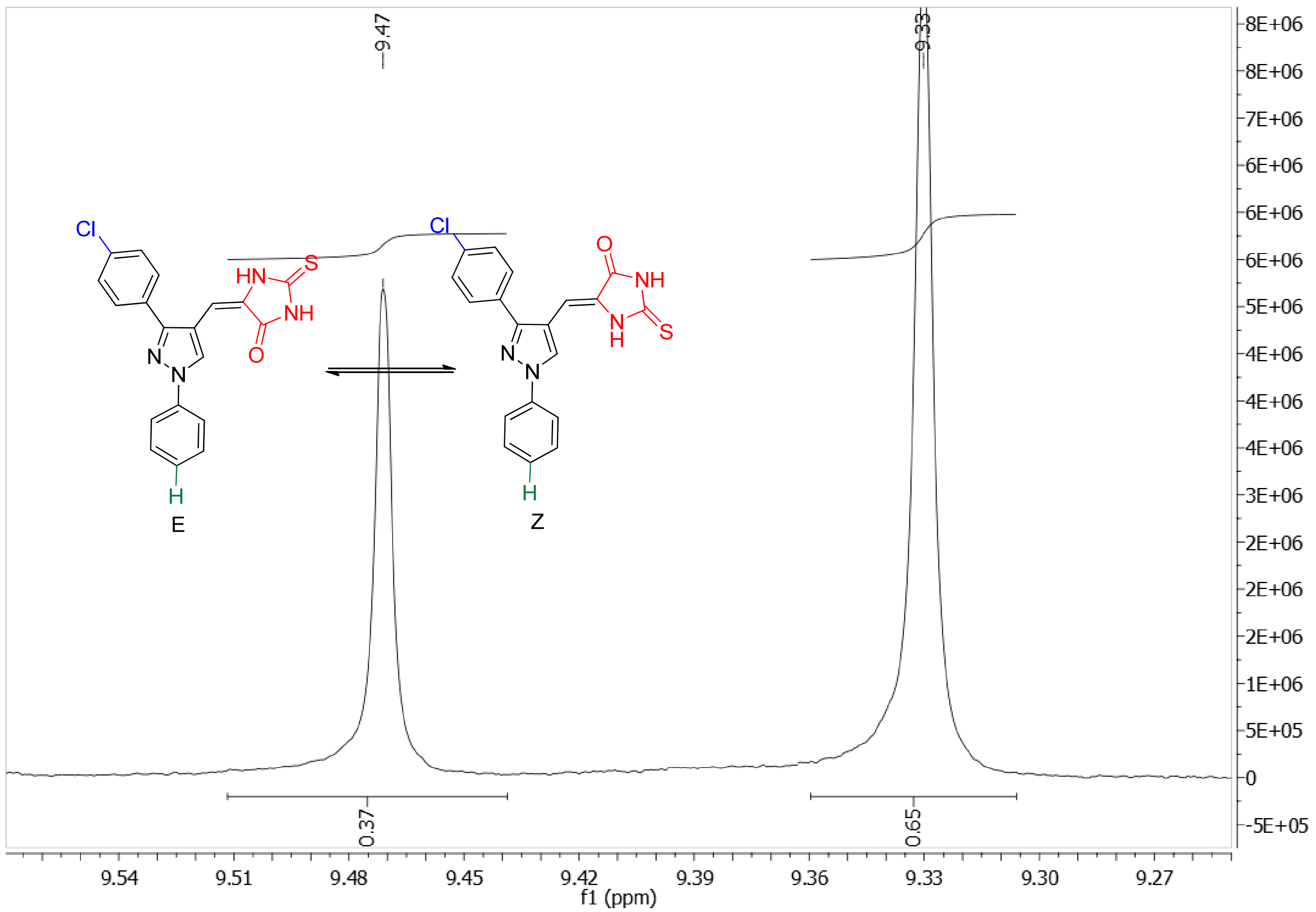

^{1}H NMR (DMSO-*d6*, 400 MHz) spectrum of 5-((3-(4-chlorophenyl)-1-phenyl-1*H*-pyrazol-4-yl)methylene)-2-thioxoimidazolidin-4-one **3b**

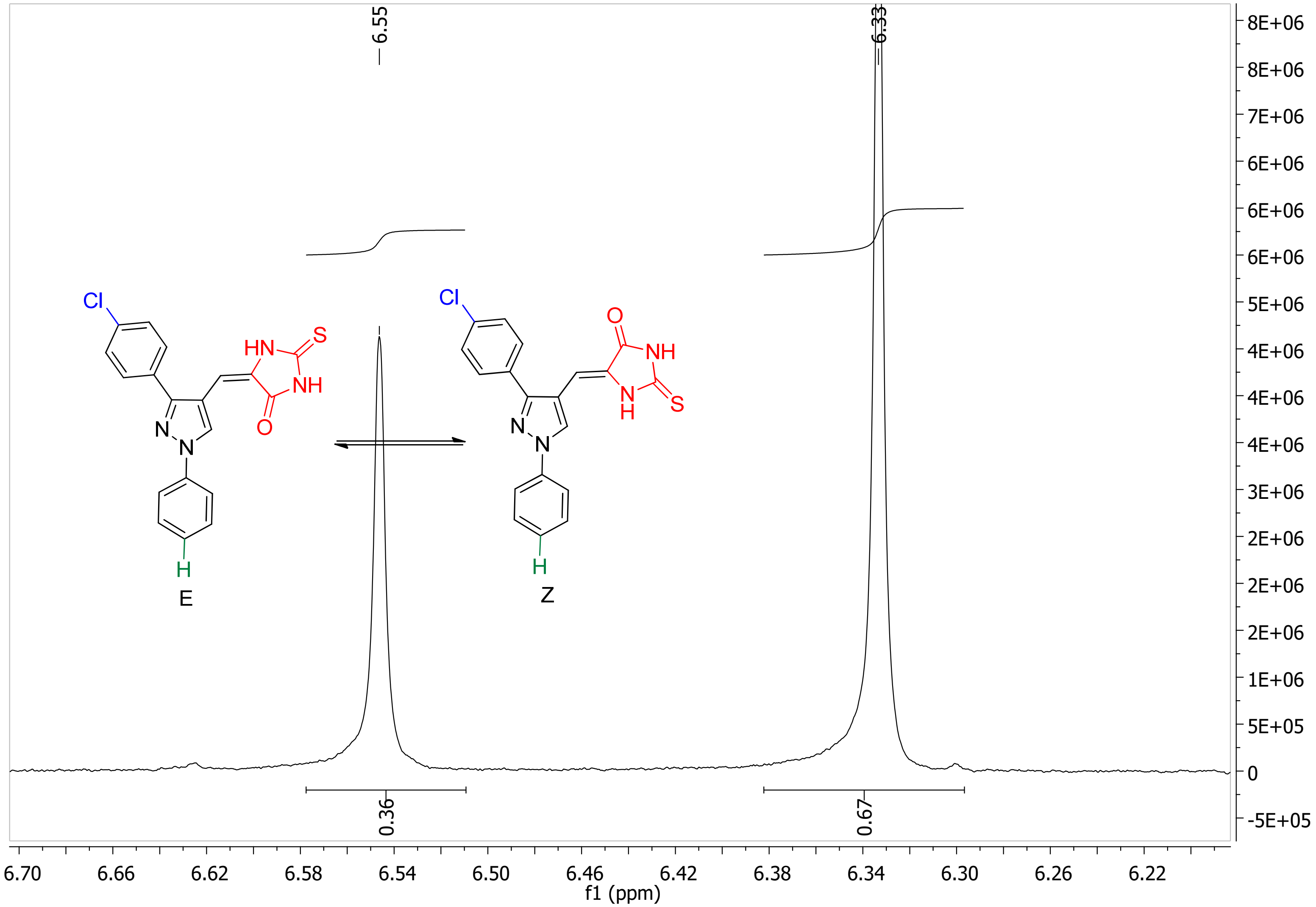

^{1}H NMR (DMSO-*d6*, 400 MHz) spectrum of 5-((3-(4-chlorophenyl)-1-phenyl-1*H*-pyrazol-4-yl)methylene)-2-thioxoimidazolidin-4-one **3b**

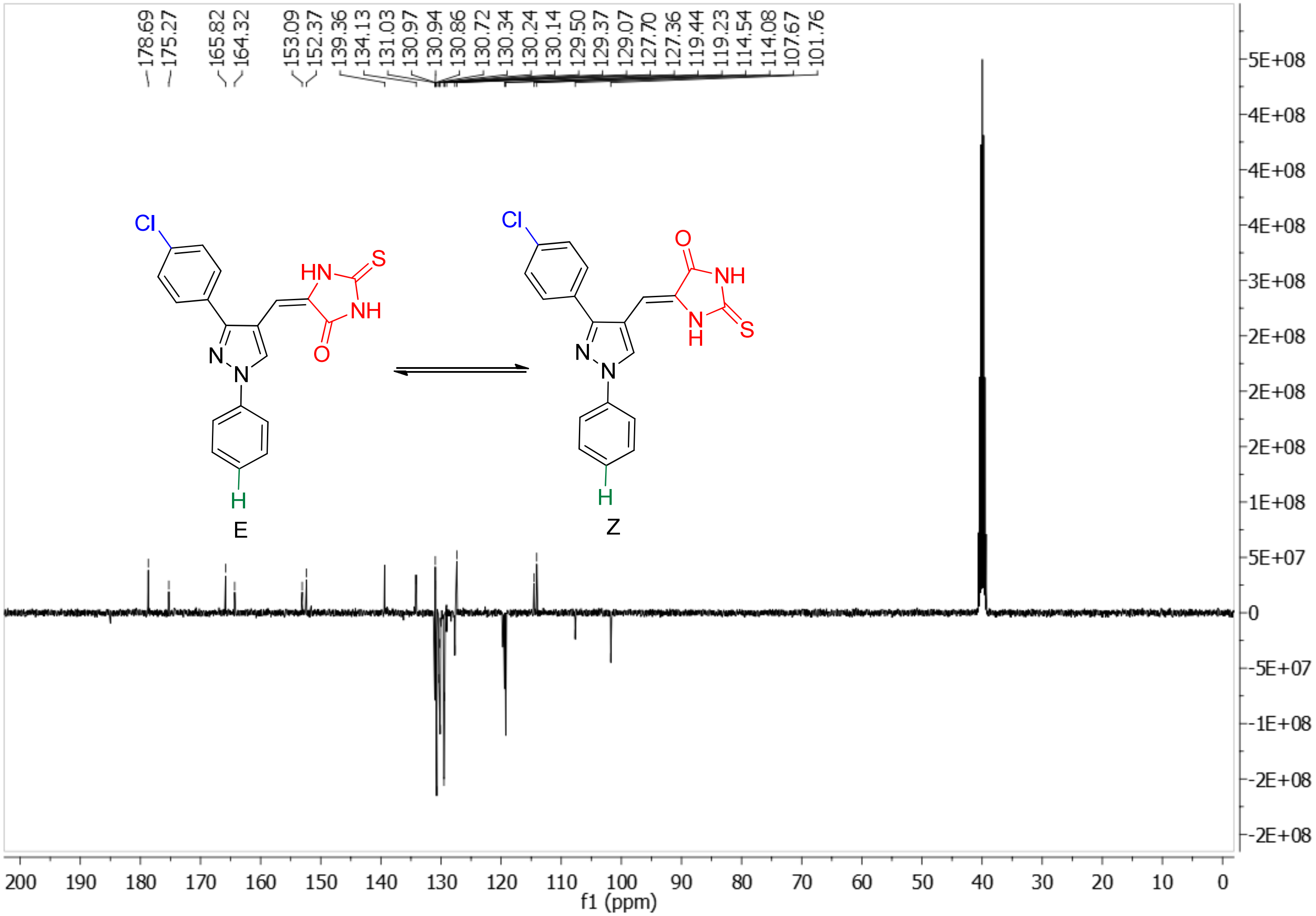

^{13}C APT NMR (DMSO-*d6*, 101 MHz) spectrum of 5-((3-(4-chlorophenyl)-1-phenyl-1*H*-pyrazol-4-yl)methylene)-2-thioxoimidazolidin-4-one

3b

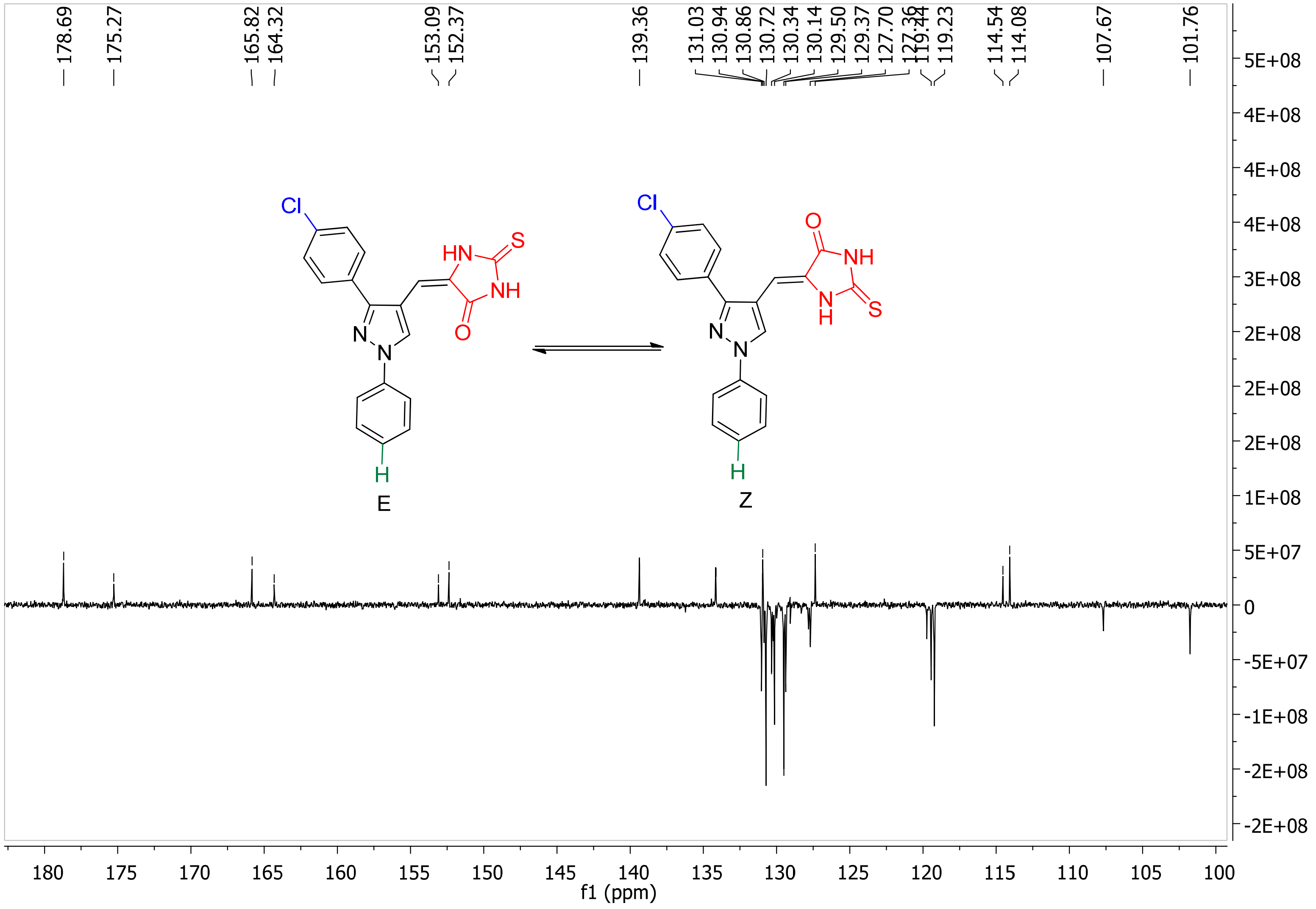

^{13}C APT NMR (DMSO-*d6*, 101 MHz) spectrum of 5-((3-(4-chlorophenyl)-1-phenyl-1*H*-pyrazol-4-yl)methylene)-2-thioxoimidazolidin-4-one **3b**

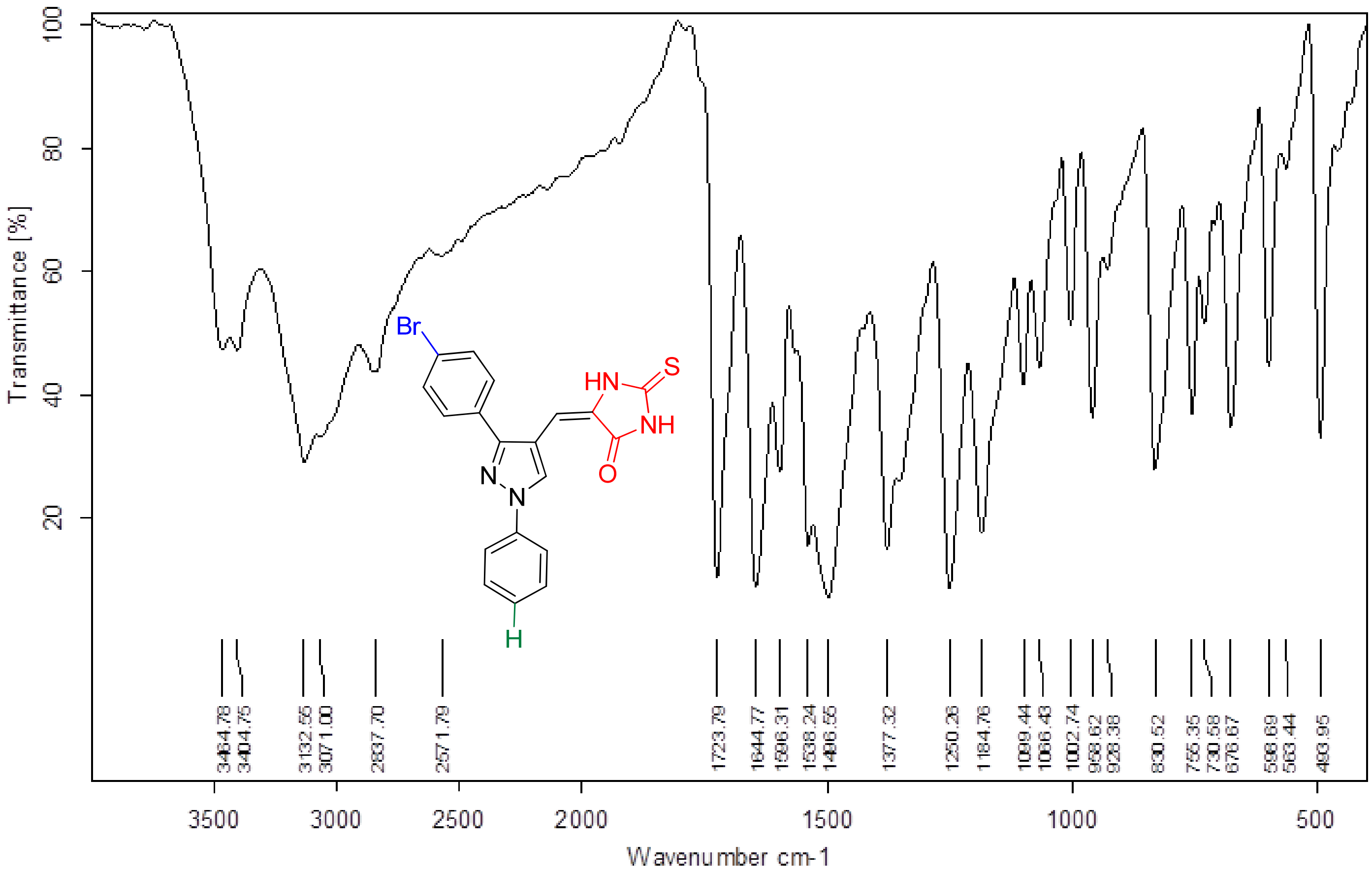

IR (KBr) spectrum of 5-((3-(4-bromophenyl)-1-phenyl-1*H*-pyrazol-4-yl)methylene)-2-thioxoimidazolidin-4-one **3c**

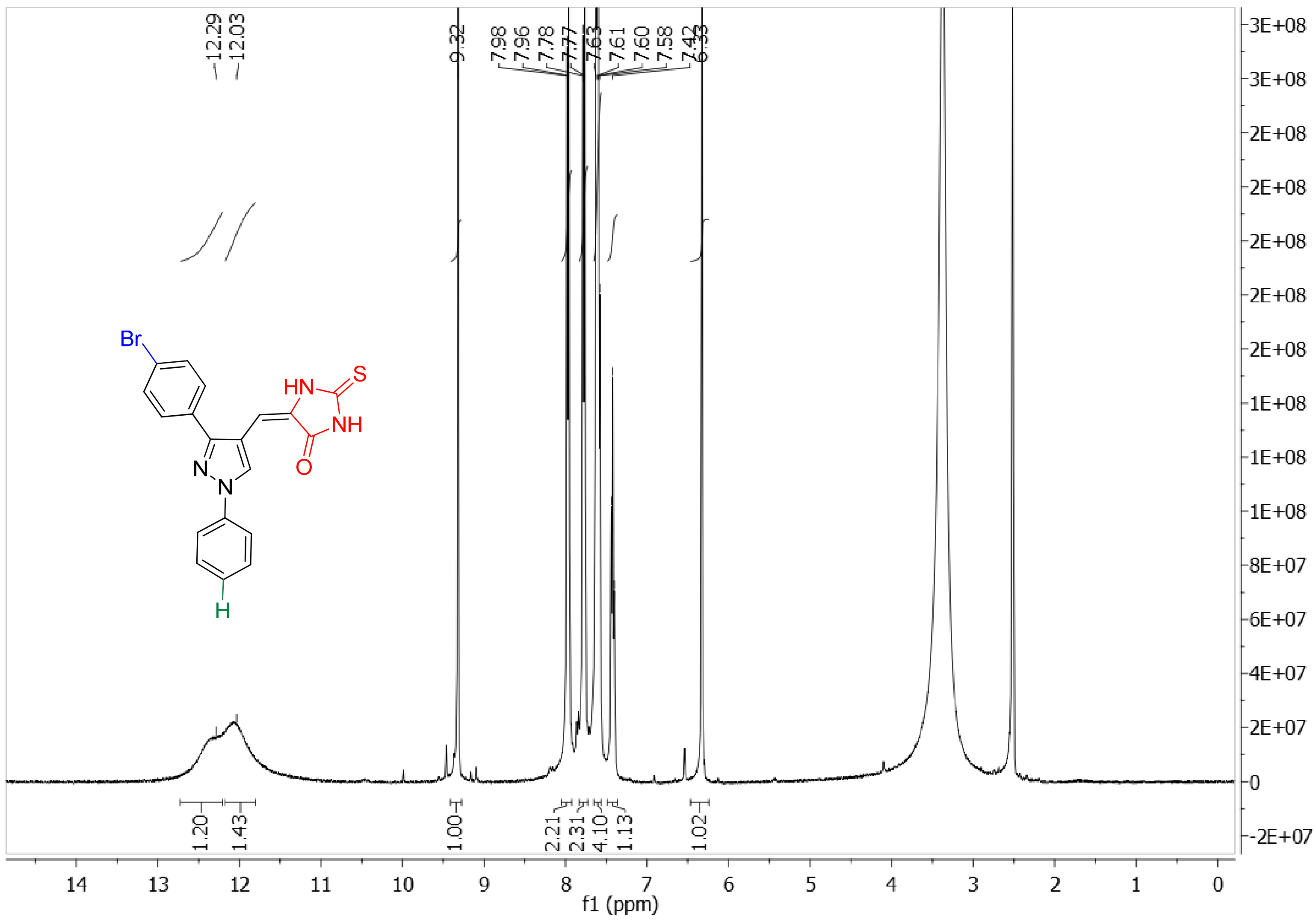

^{1}H NMR (DMSO-*d6*, 400 MHz) spectrum of 5-((3-(4-bromophenyl)-1-phenyl-1*H*-pyrazol-4-yl)methylene)-2-thioxoimidazolidin-4-one **3c**

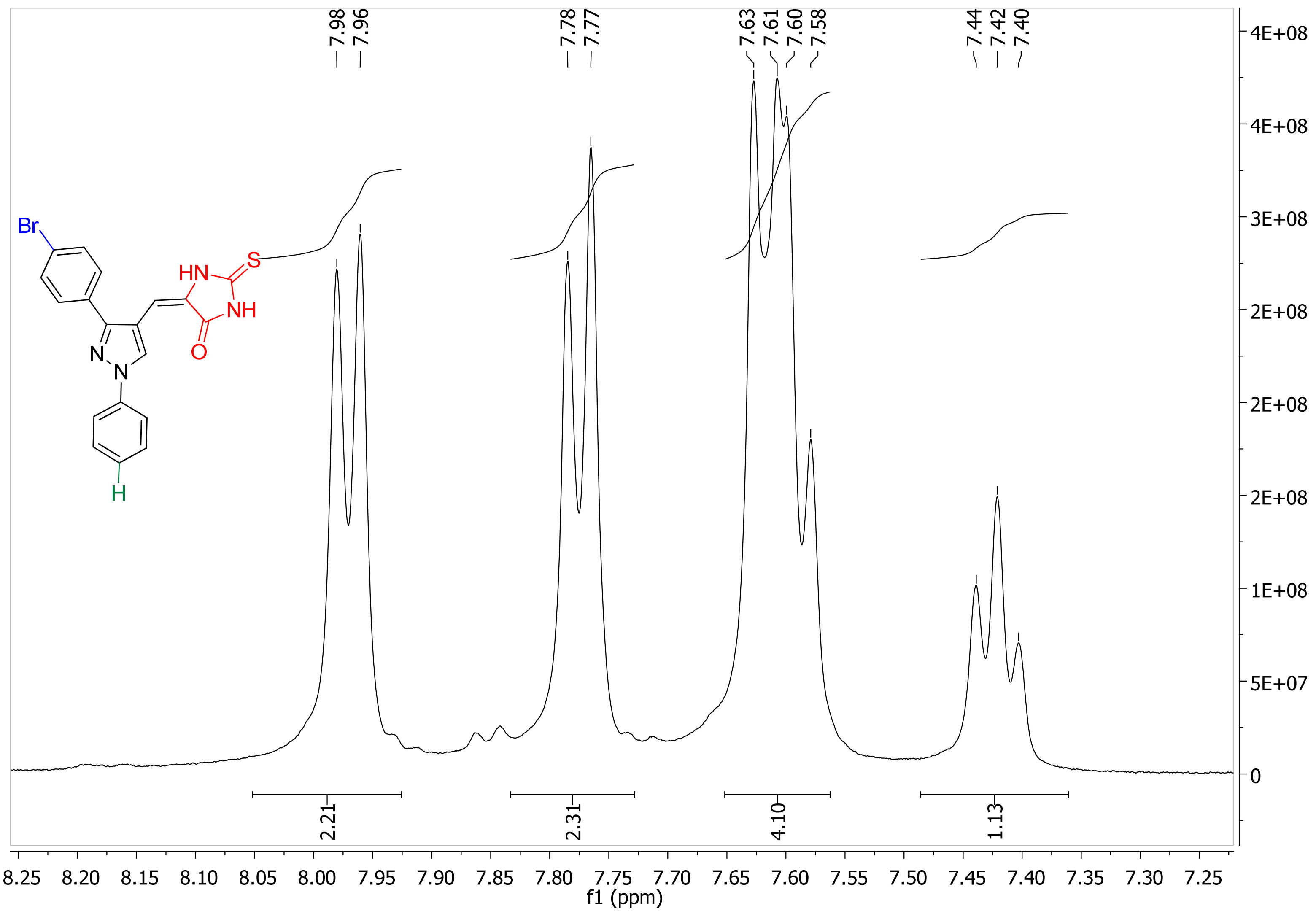

^{1}H NMR (DMSO-*d6*, 400 MHz) spectrum of 5-((3-(4-bromophenyl)-1-phenyl-1*H*-pyrazol-4-yl)methylene)-2-thioxoimidazolidin-4-one **3c**

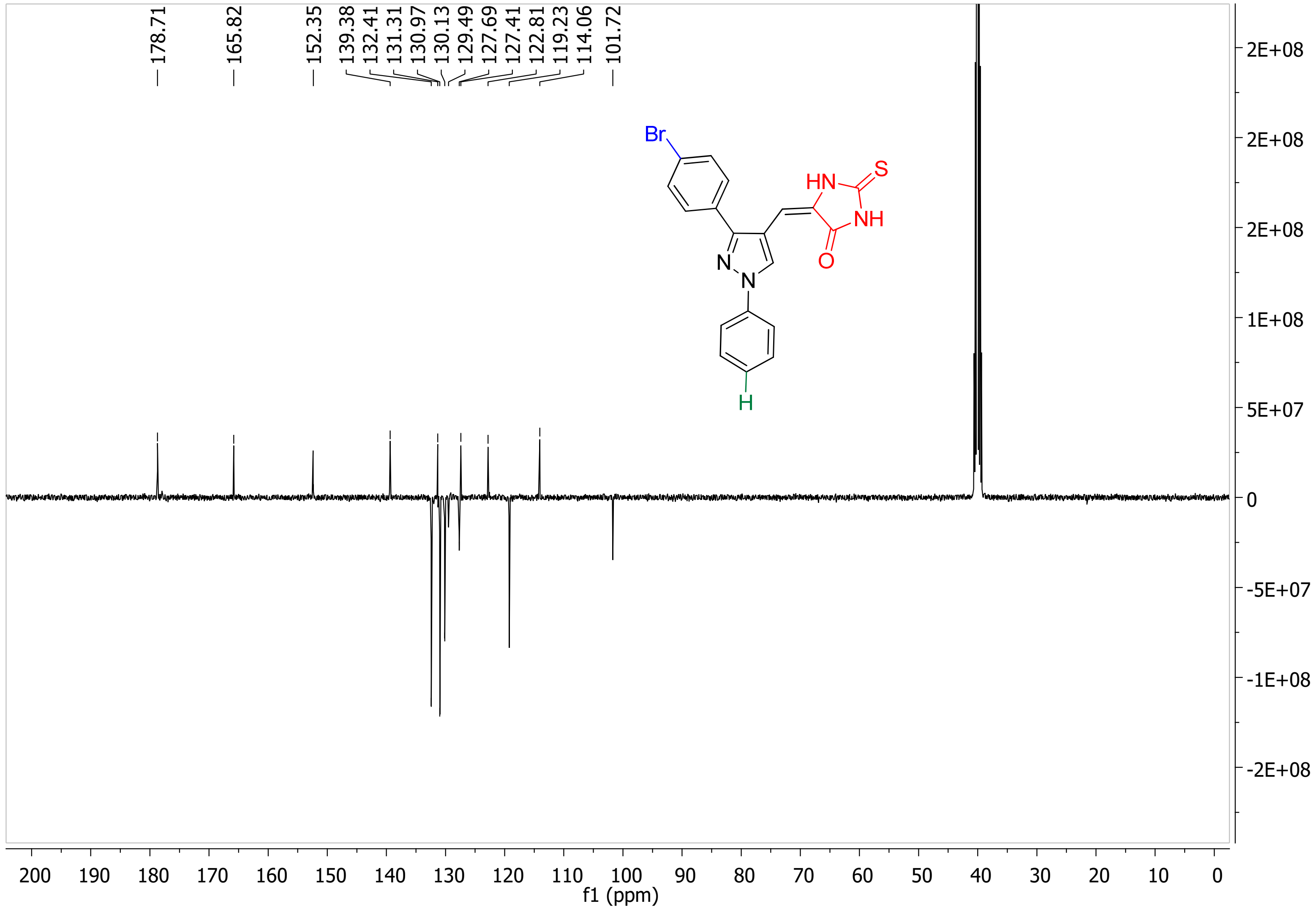

^{13}C APT NMR (DMSO-*d6*, 101 MHz) spectrum of 5-((3-(4-bromophenyl)-1-phenyl-1*H*-pyrazol-4-yl)methylene)-2-thioxoimidazolidin-4-one

3c

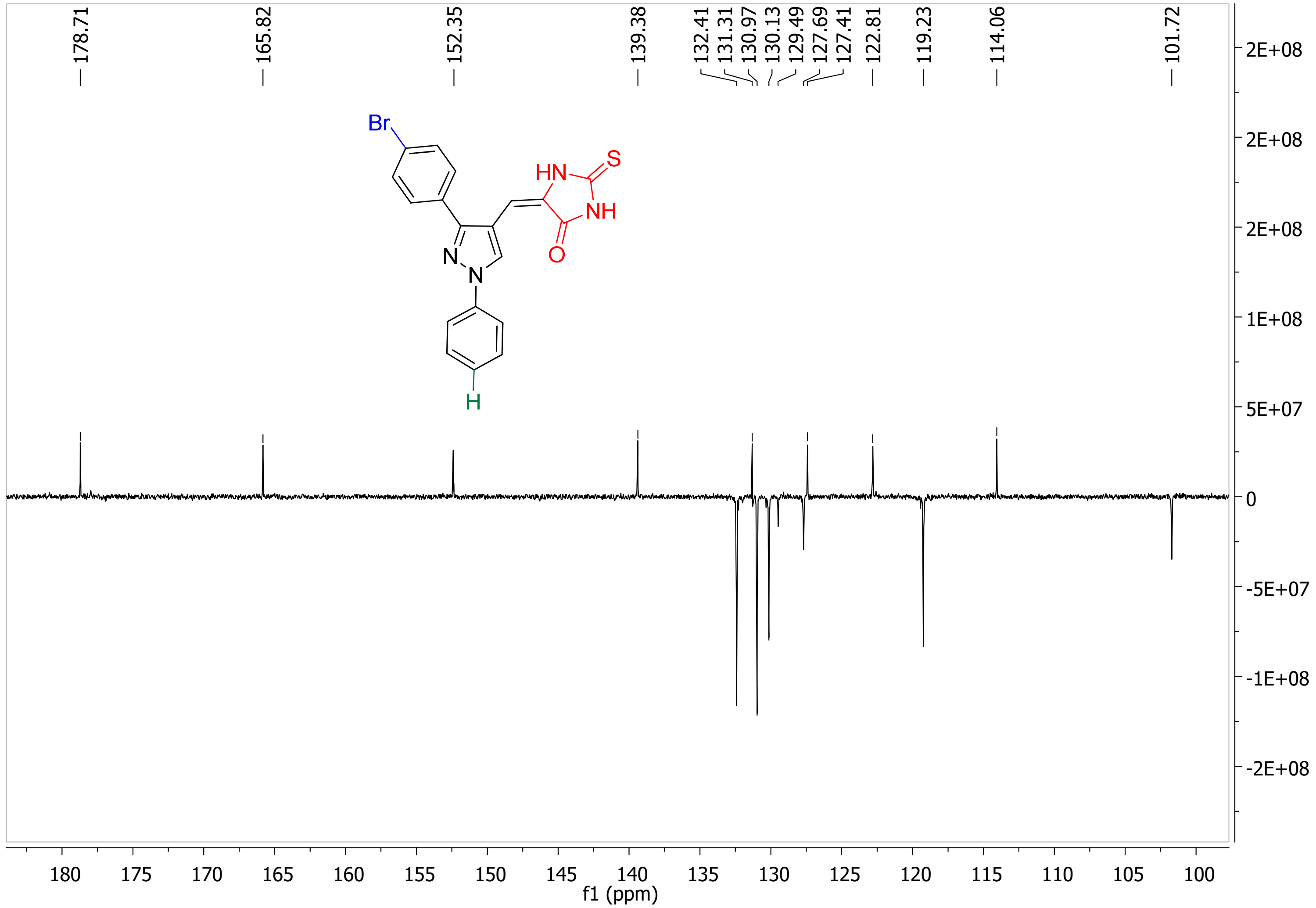

^{13}C APT NMR (DMSO-*d6*, 101 MHz) spectrum of 5-((3-(4-bromophenyl)-1-phenyl-1*H*-pyrazol-4-yl)methylene)-2-thioxoimidazolidin-4-one **3c**

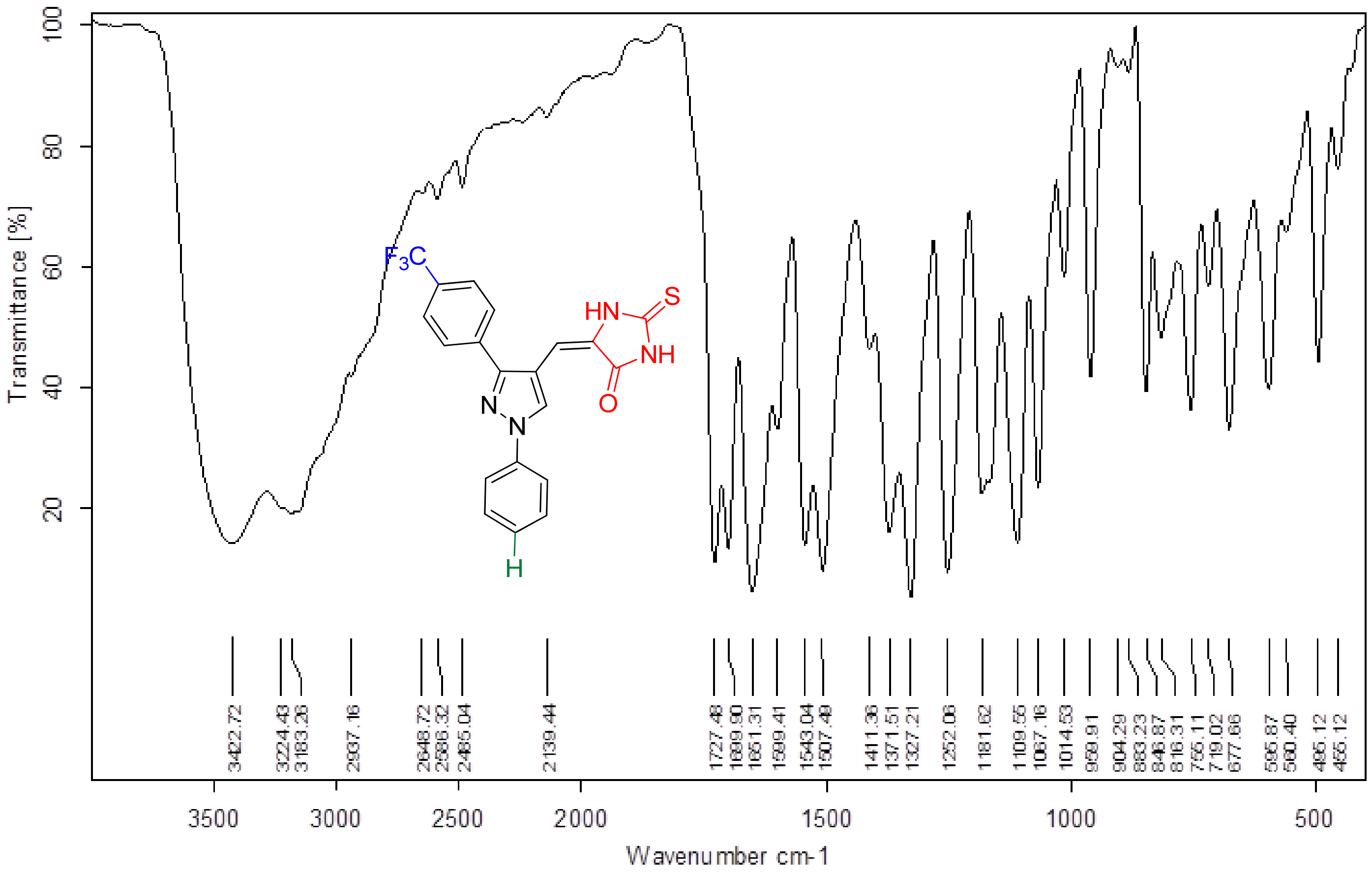

IR (KBr) spectrum of 5-((1-phenyl-3-(4-(trifluoromethyl)phenyl)-1*H*-pyrazol-4-yl)methylene)-2-thioxoimidazolidin-4-one **3d**

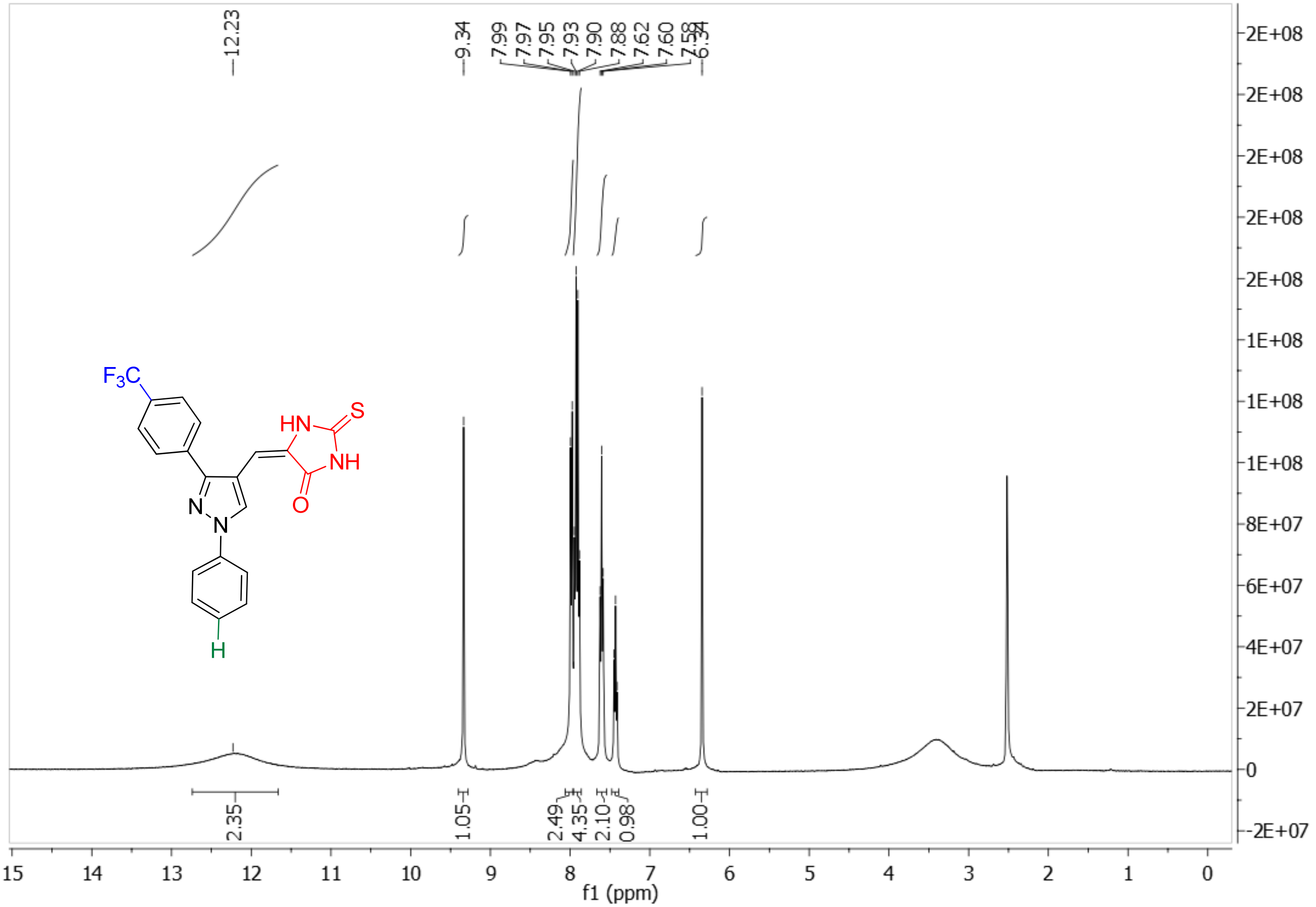

^{1}H NMR (DMSO-*d6*, 400 MHz) spectrum of 5-((1-phenyl-3-(4-(trifluoromethyl)phenyl)-1*H*-pyrazol-4-yl)methylene)-2-thioxoimidazolidin-4-one **3d**

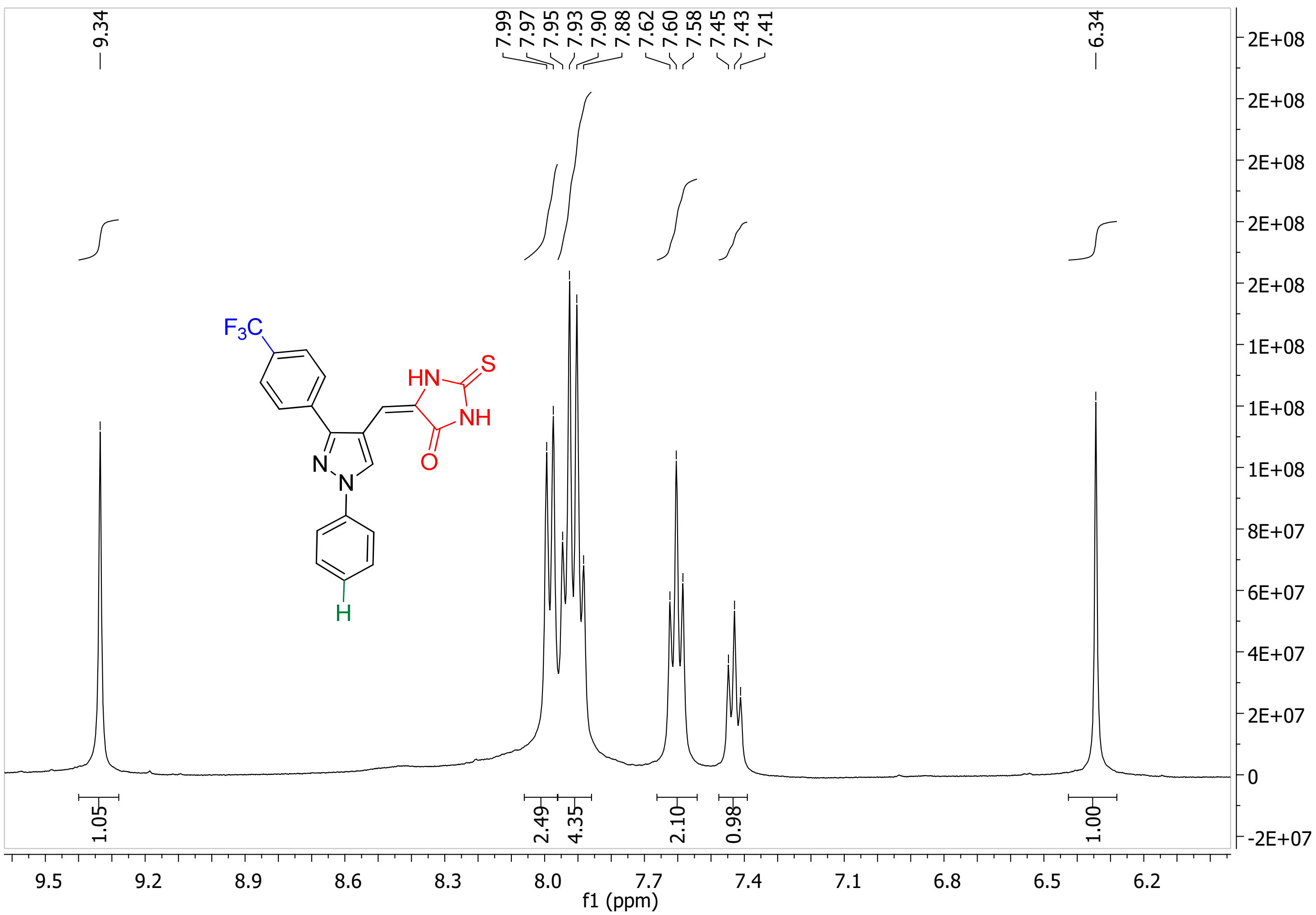

^{1}H NMR (DMSO-*d6*, 400 MHz) spectrum of 5-((1-phenyl-3-(4-(trifluoromethyl)phenyl)-1*H*-pyrazol-4-yl)methylene)-2-thioxoimidazolidin-4-one **3d**

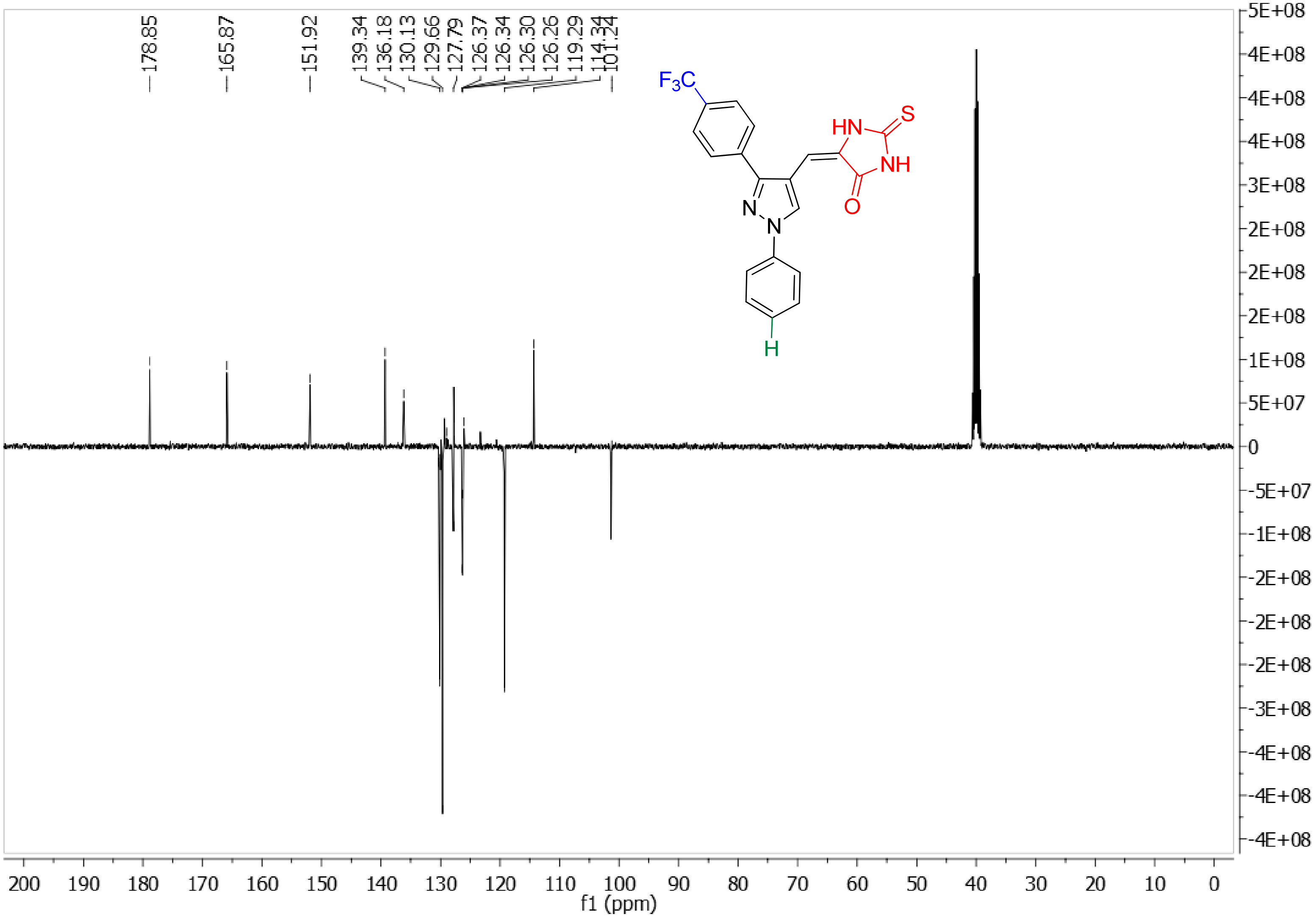

^{13}C APT NMR (DMSO-*d6*, 101 MHz) spectrum of 5-((1-phenyl-3-(4-(trifluoromethyl)phenyl)-1*H*-pyrazol-4-yl)methylene)-2-thioxoimidazolidin-4-one **3d**

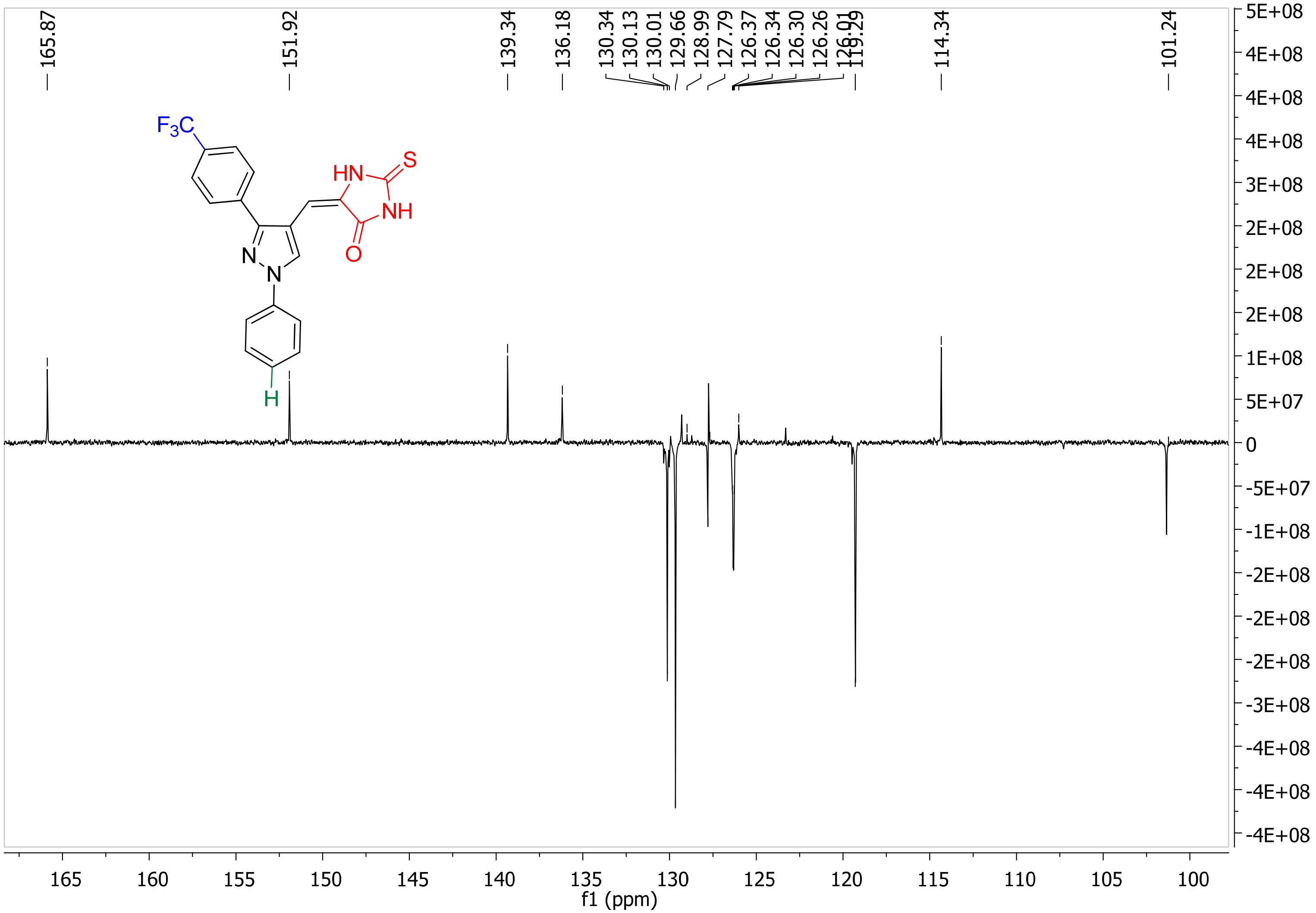

^{13}C APT NMR (DMSO-*d6*, 101 MHz) spectrum of 5-((1-phenyl-3-(4-(trifluoromethyl)phenyl)-1*H*-pyrazol-4-yl)methylene)-2-thioxoimidazolidin-4-one **3d**

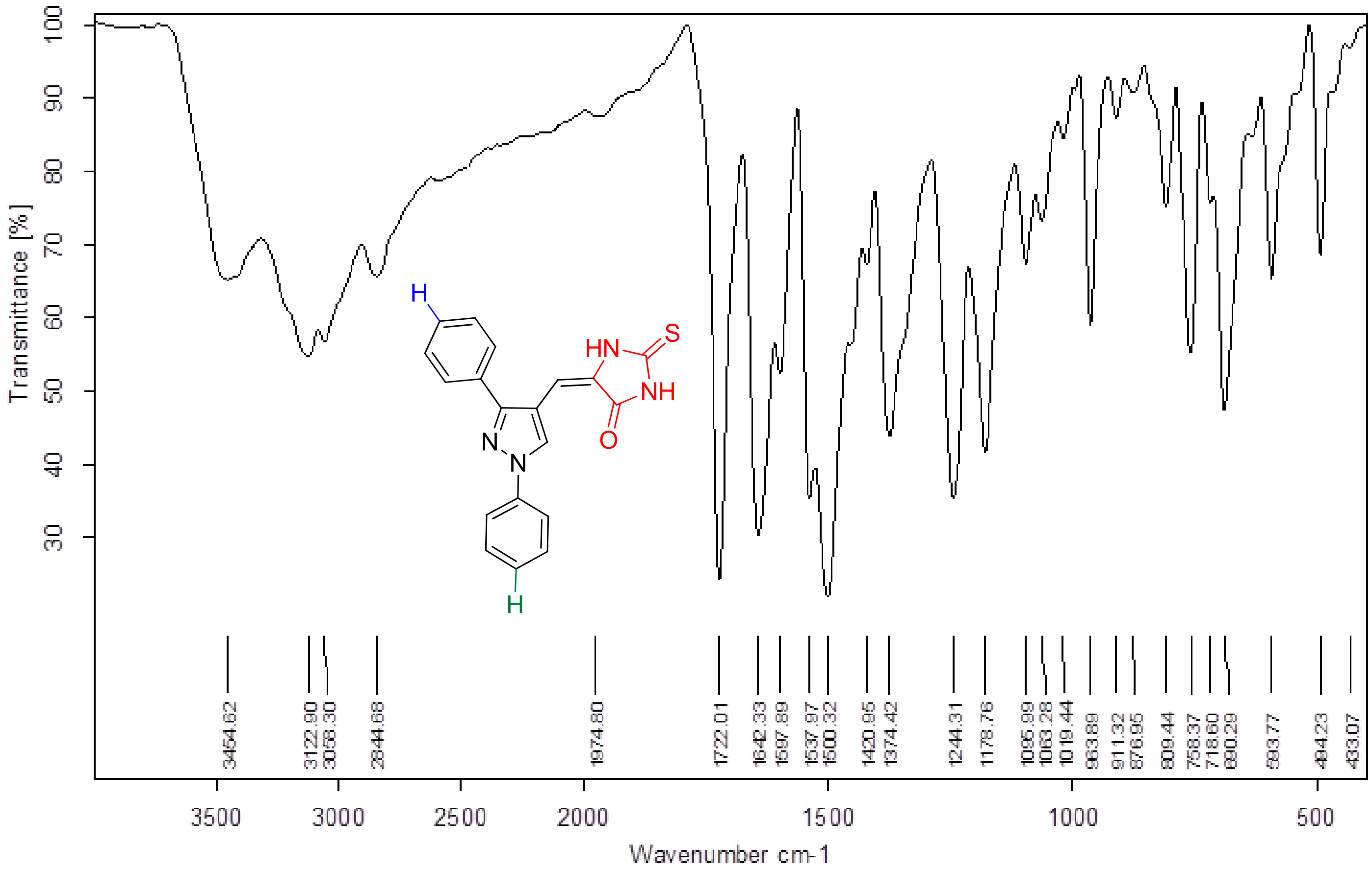

IR (KBr) spectrum of 5-((1,3-diphenyl-1H-pyrazol-4-yl)methylene)-2-thioxoimidazolidin-4-one **3e**

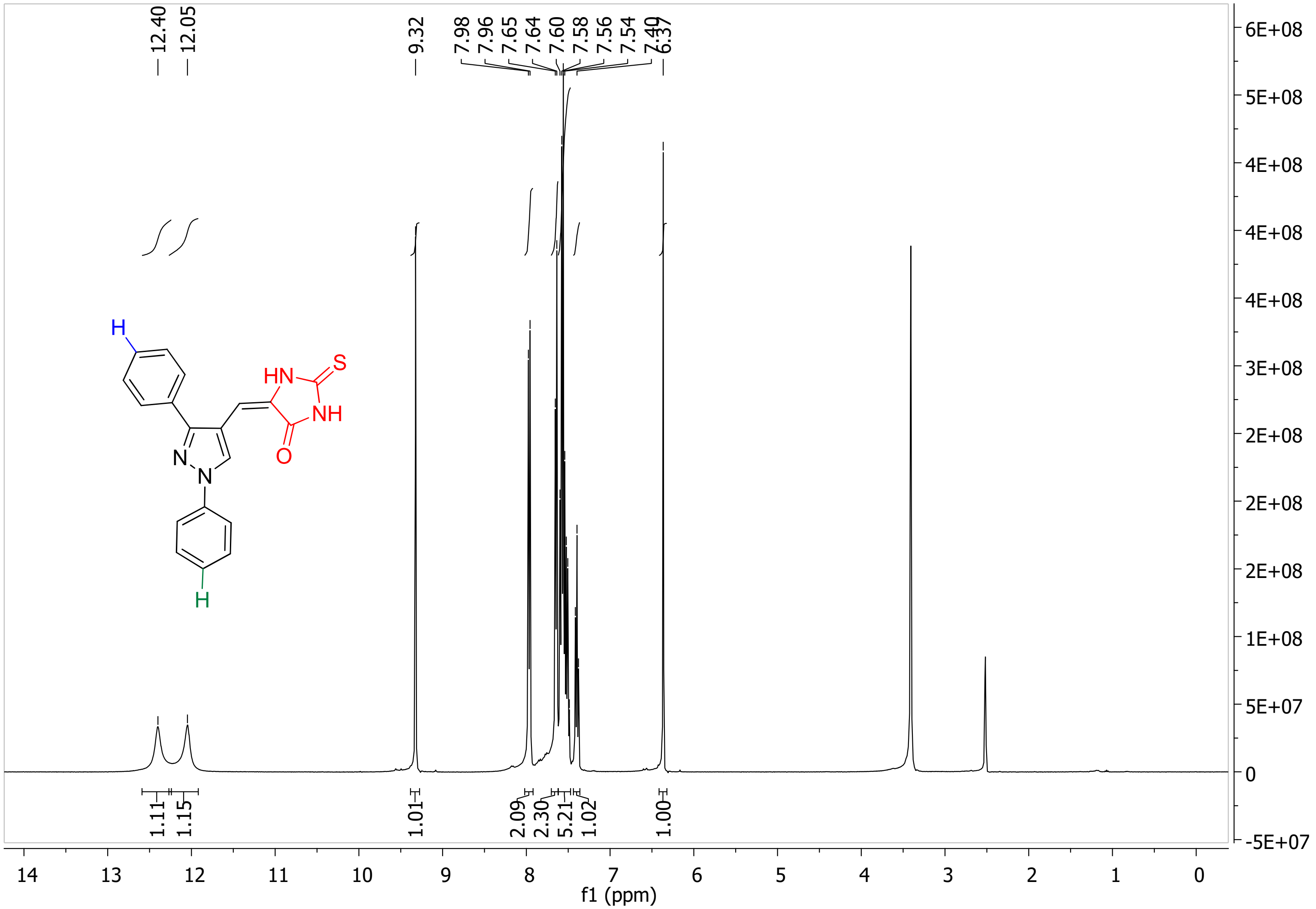

^{1}H NMR (DMSO-*d6*, 400 MHz) spectrum of 5-((1,3-diphenyl-1H-pyrazol-4-yl)methylene)-2-thioxoimidazolidin-4-one **3e**

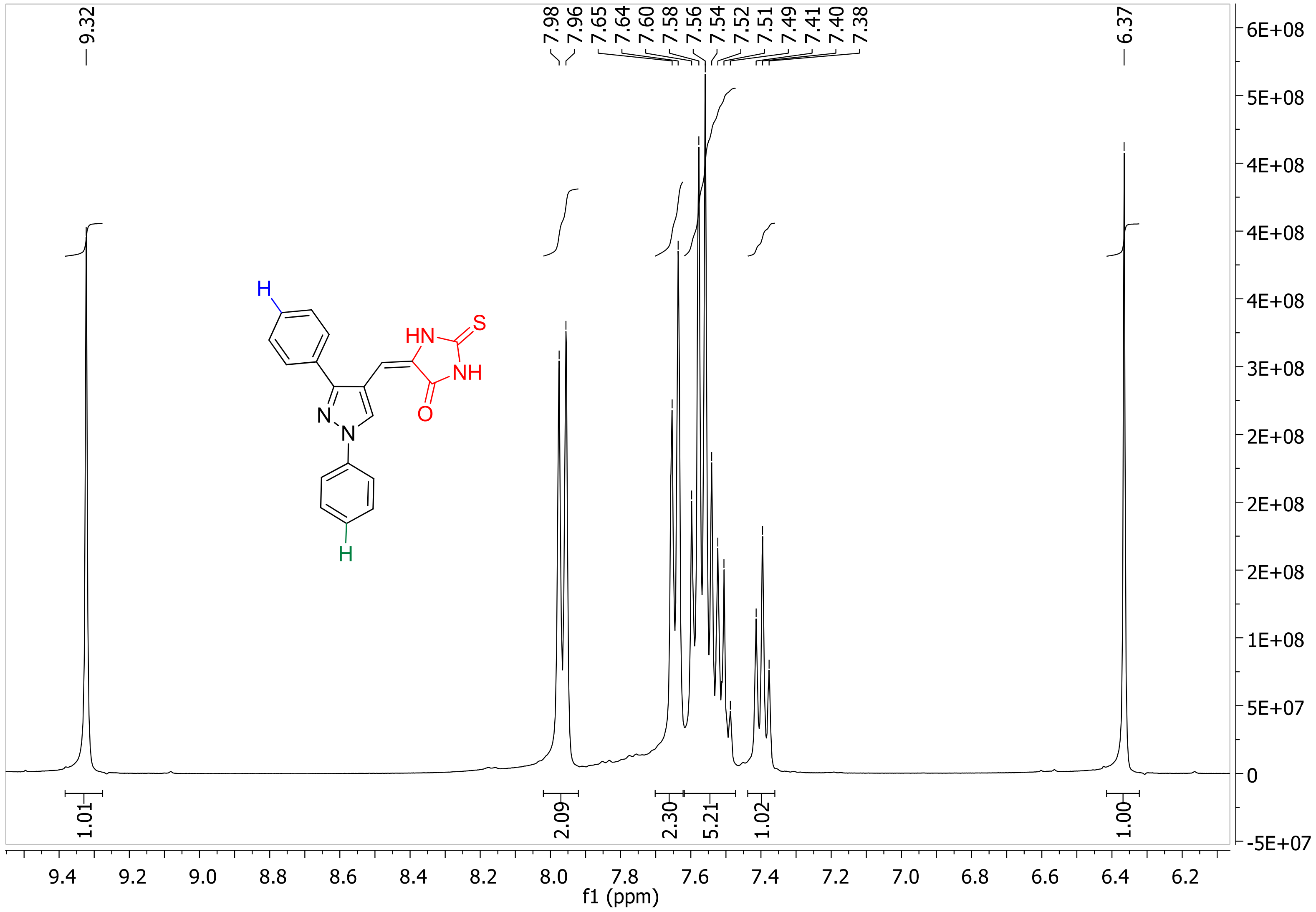

^{1}H NMR (DMSO-*d6*, 400 MHz) spectrum of 5-((1,3-diphenyl-1H-pyrazol-4-yl)methylene)-2-thioxoimidazolidin-4-one **3e**

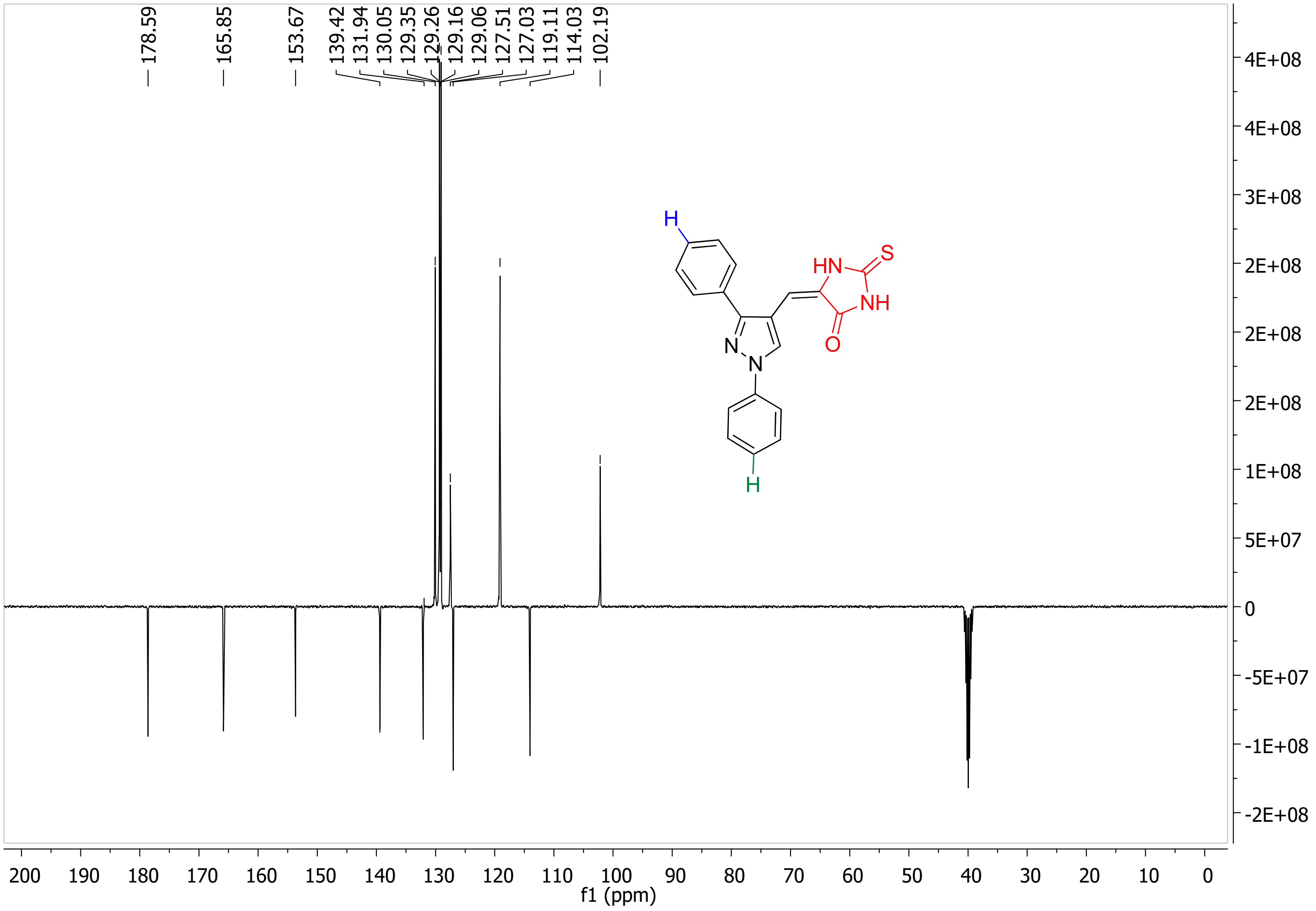

^{13}C APT NMR (DMSO-*d6*, 101 MHz) spectrum of 5-((1,3-diphenyl-1H-pyrazol-4-yl)methylene)-2-thioxoimidazolidin-4-one **3e**

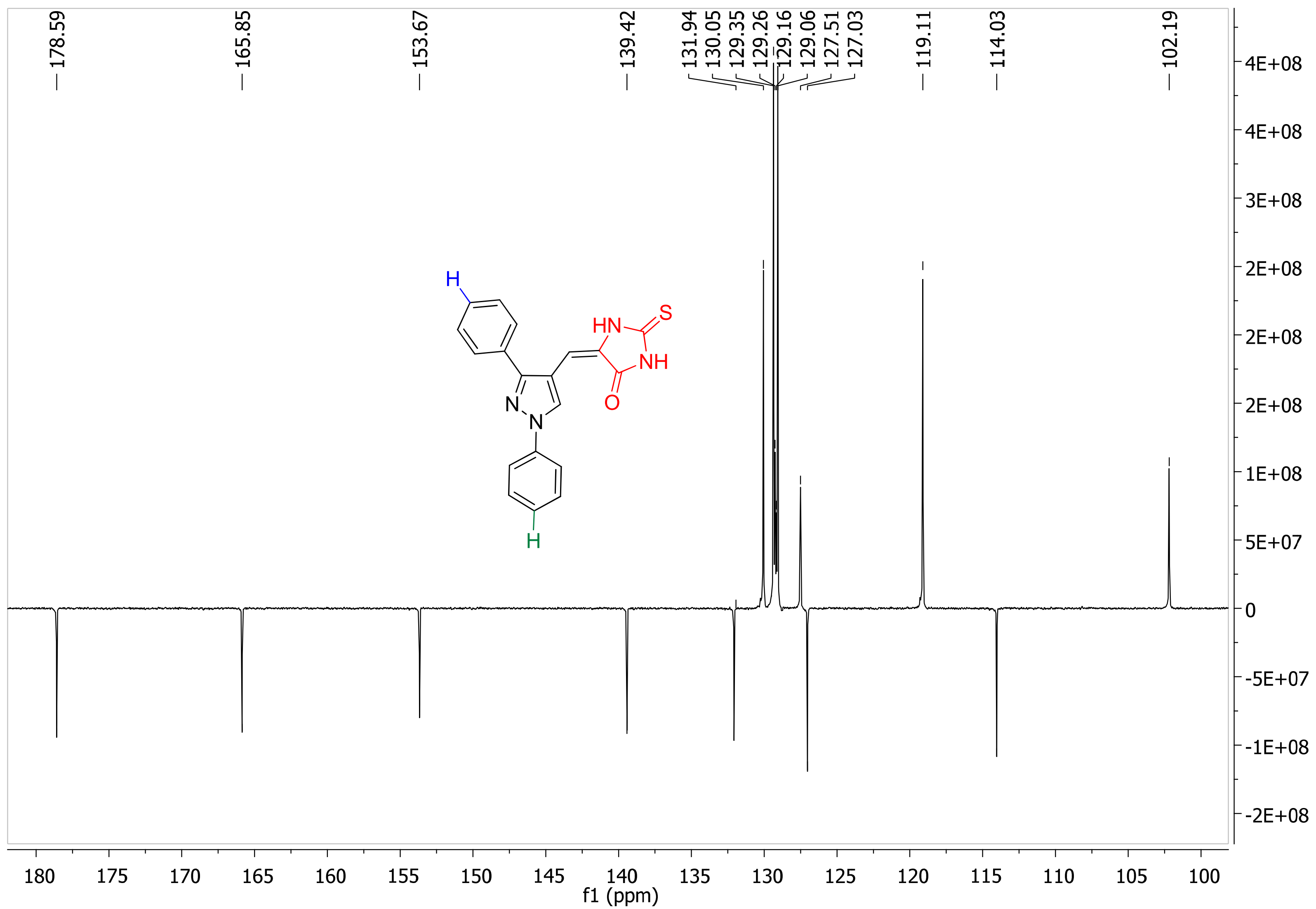

^{13}C APT NMR (DMSO-*d6*, 101 MHz) spectrum of 5-((1,3-diphenyl-1H-pyrazol-4-yl)methylene)-2-thioxoimidazolidin-4-one **3e**

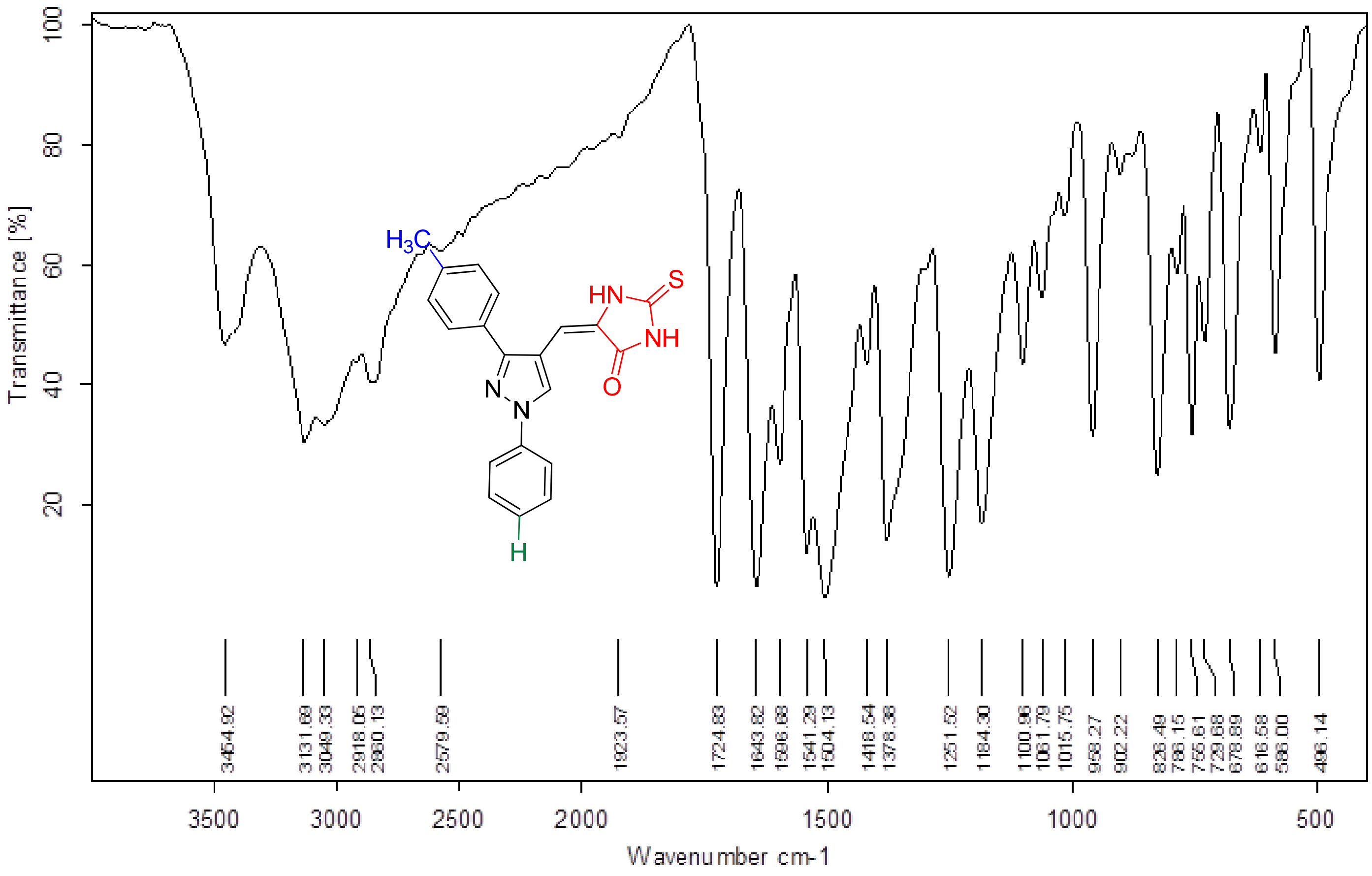

IR (KBr) spectrum of 5-((1-phenyl-3-(p-tolyl)-1*H*-pyrazol-4-yl)methylene)-2-thioxoimidazolidin-4-one **3f**

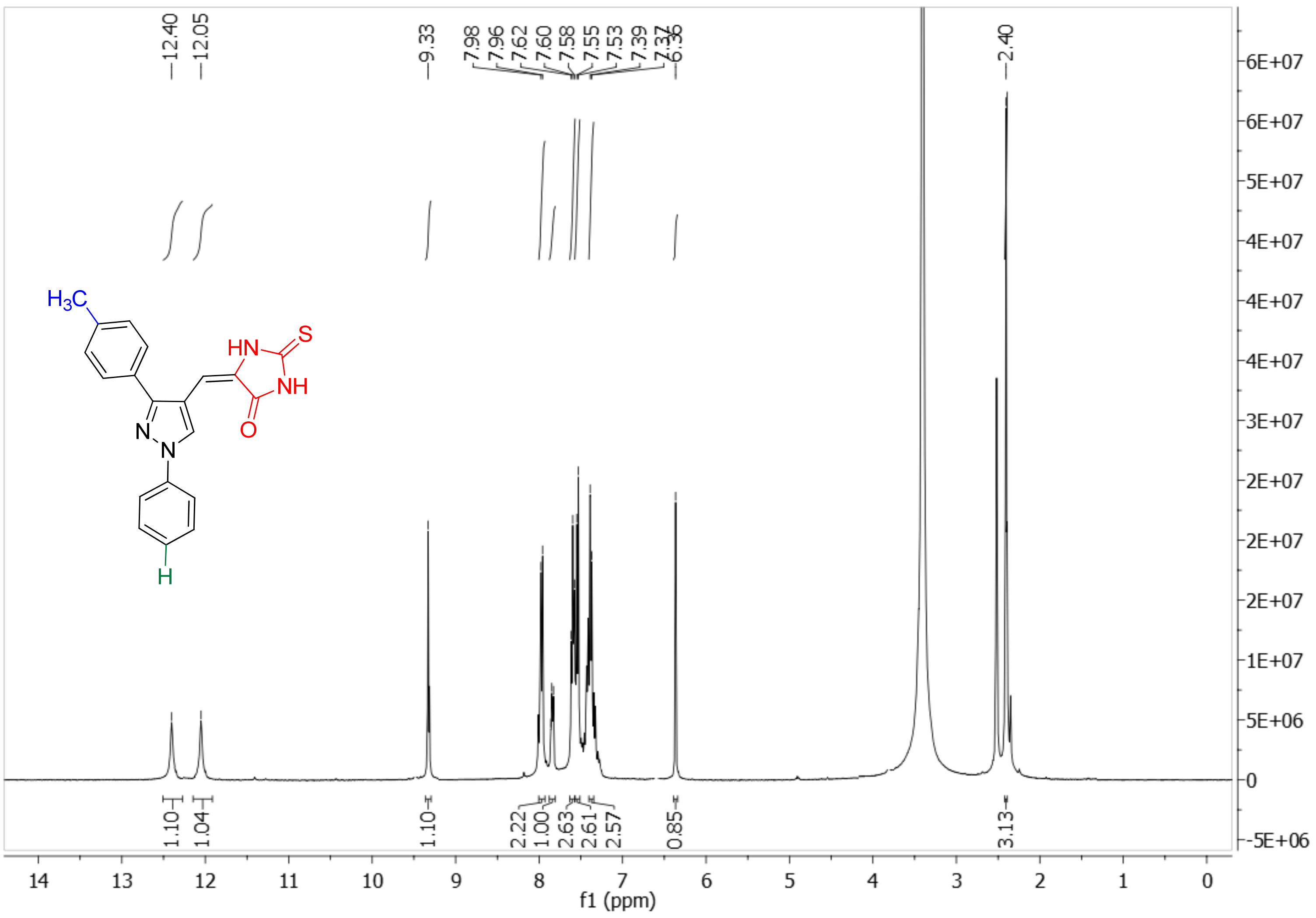

^{1}H NMR (DMSO-*d6*, 400 MHz) spectrum of 5-((1-phenyl-3-(p-tolyl)-1*H*-pyrazol-4-yl)methylene)-2-thioxoimidazolidin-4-one **3f**

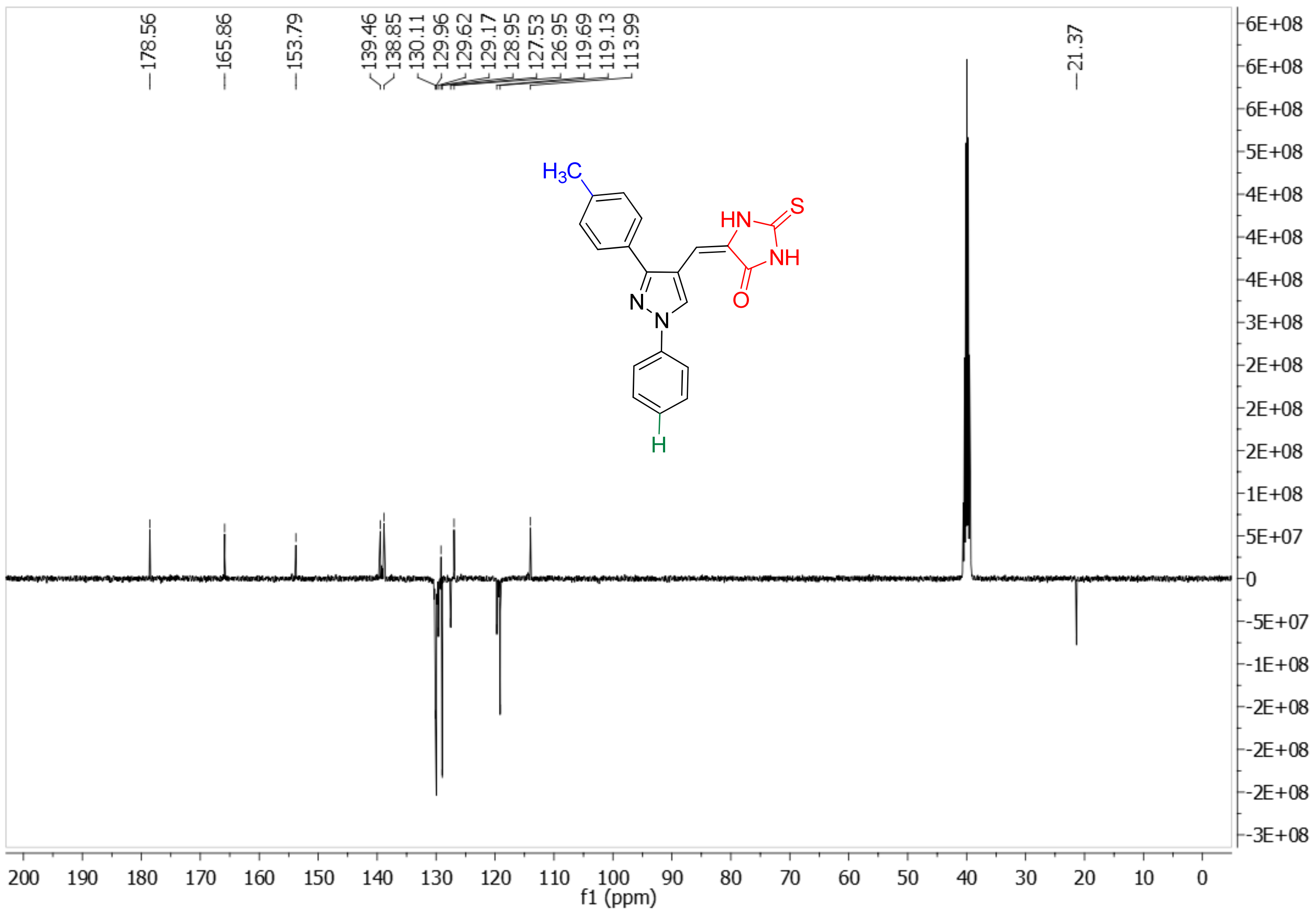

^{13}C APT NMR (DMSO-*d6*, 101 MHz) spectrum of 5-((1-phenyl-3-(p-tolyl)-1*H*-pyrazol-4-yl)methylene)-2-thioxoimidazolidin-4-one **3f**

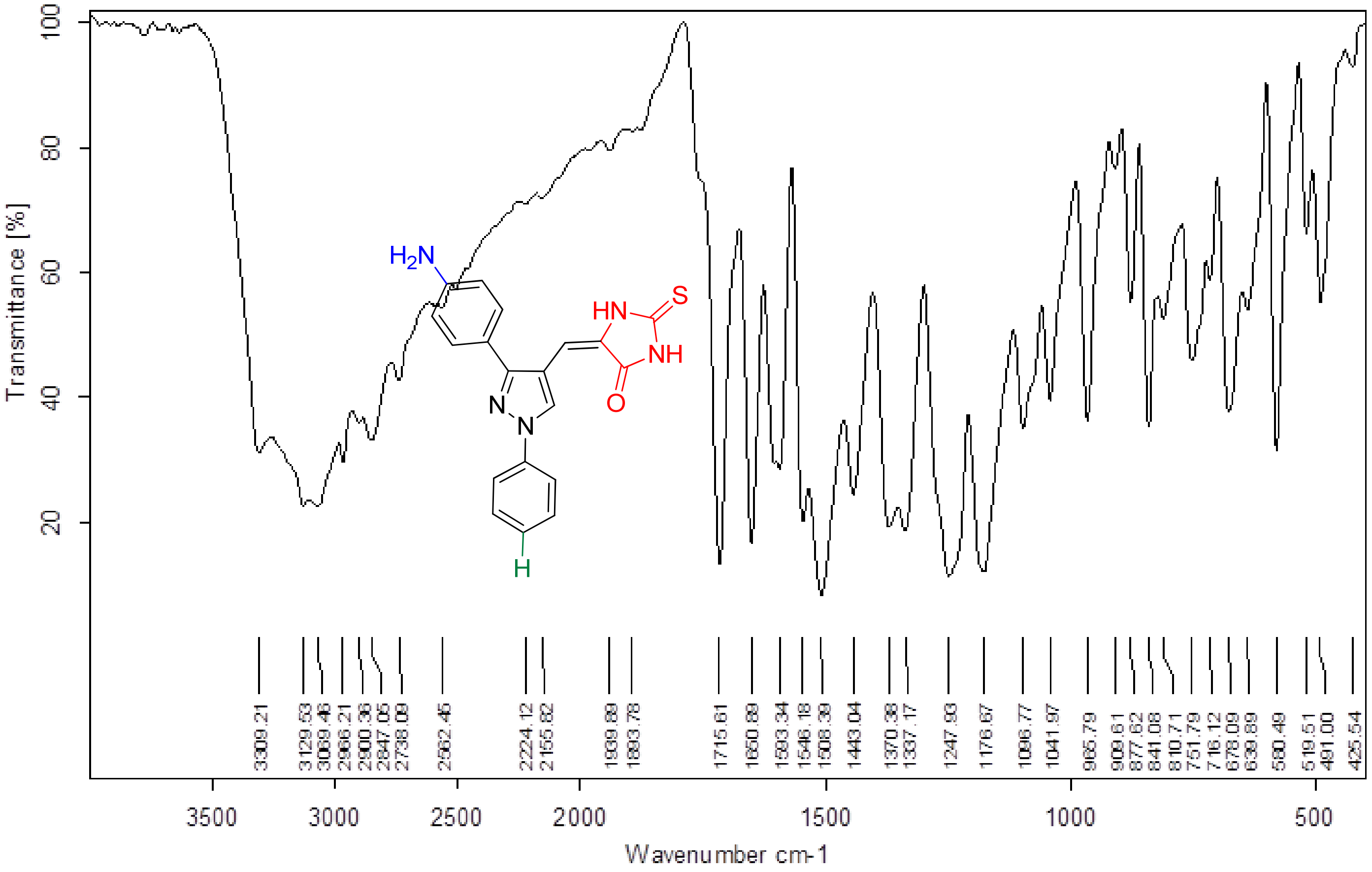

IR (KBr) spectrum of 5-((3-(4-aminophenyl)-1-phenyl-1*H*-pyrazol-4-yl)methylene)-2-thioxoimidazolidin-4-one **3g**

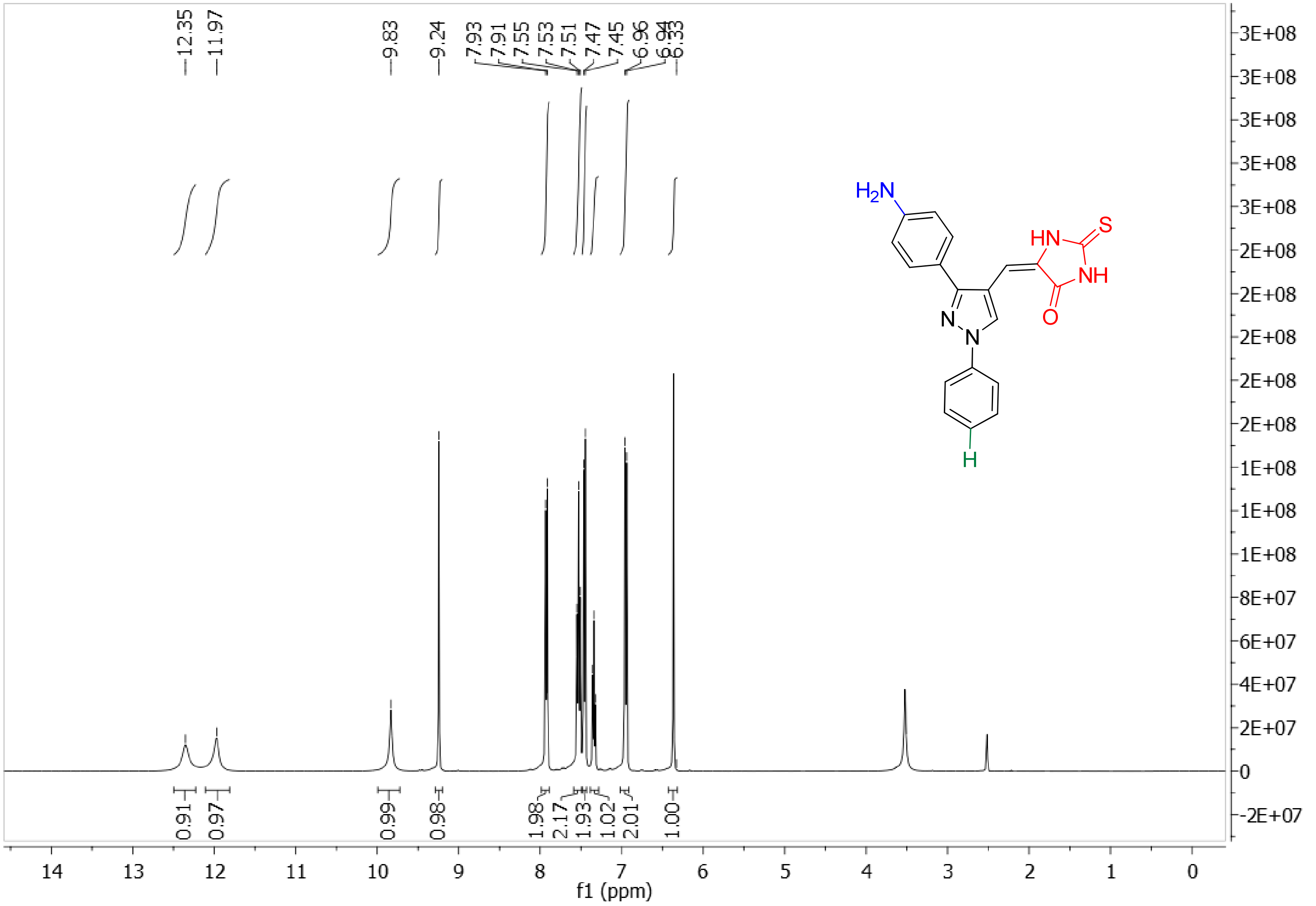

^{1}H NMR (DMSO-*d6*, 400 MHz) spectrum of 5-((3-(4-aminophenyl)-1-phenyl-1*H*-pyrazol-4-yl)methylene)-2-thioxoimidazolidin-4-one **3g**

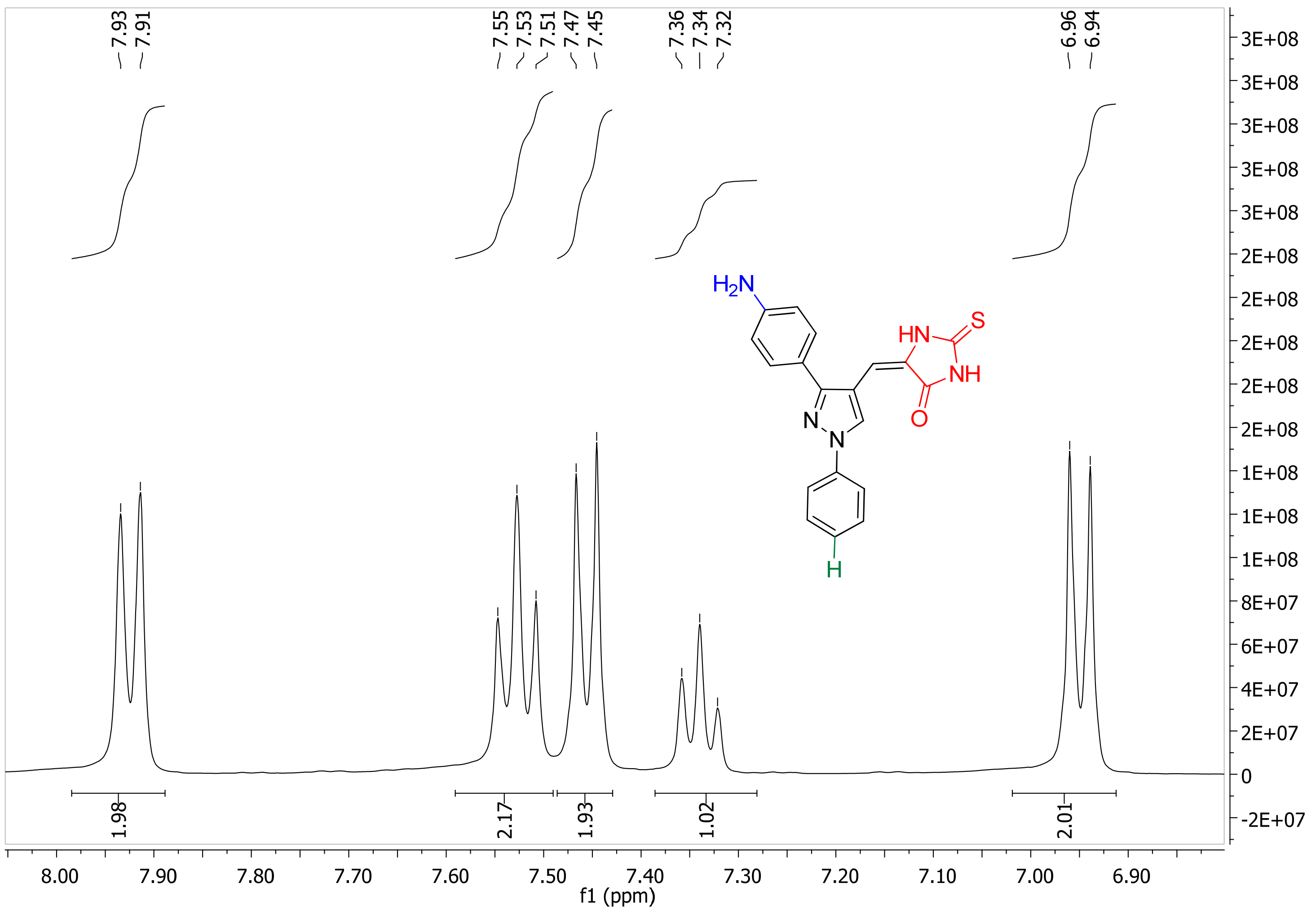

^{1}H NMR (DMSO-*d6*, 400 MHz) spectrum of 5-((3-(4-aminophenyl)-1-phenyl-1*H*-pyrazol-4-yl)methylene)-2-thioxoimidazolidin-4-one **3g**

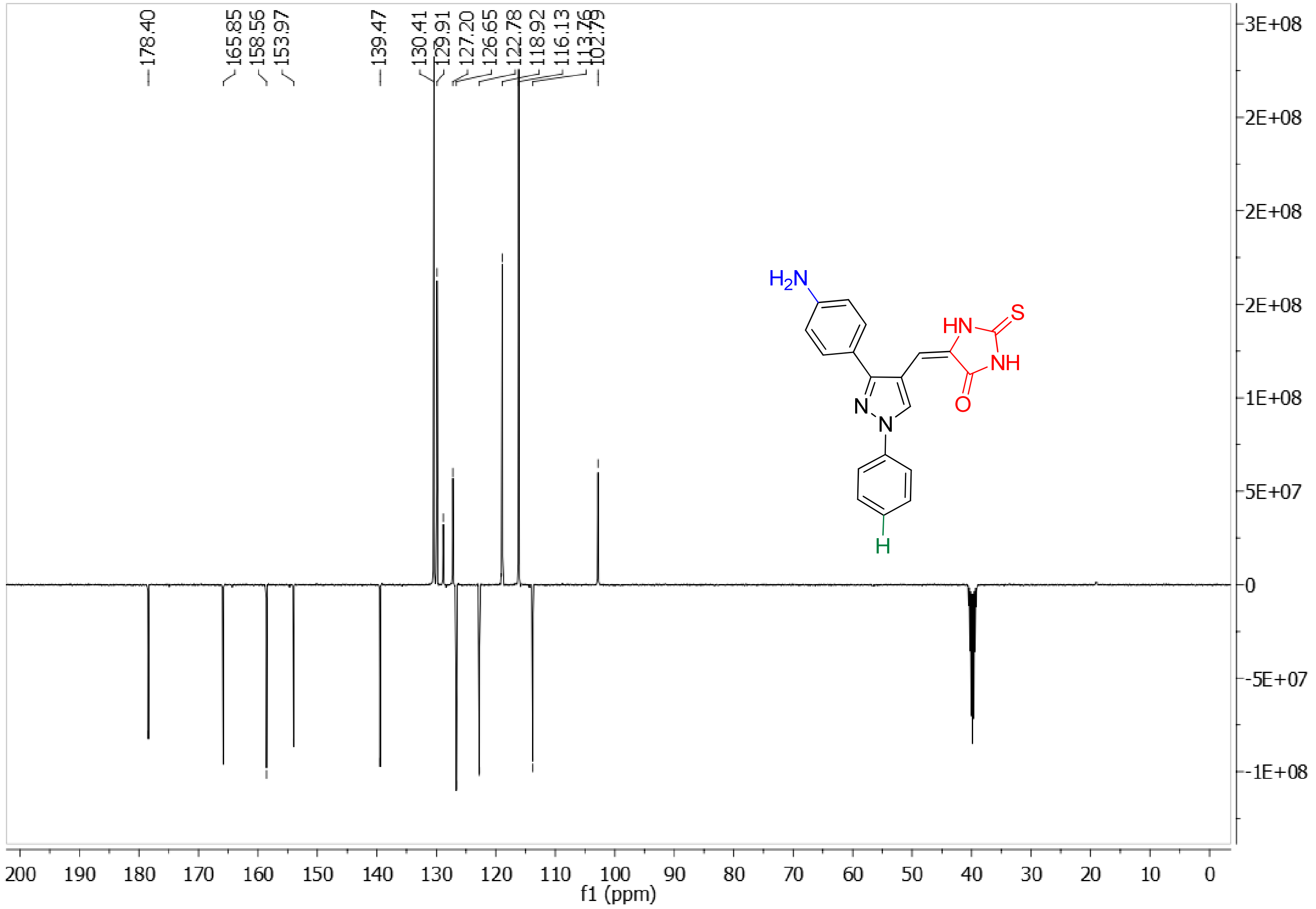

^{13}C APT NMR (DMSO-*d6*, 101 MHz) spectrum of 5-((3-(4-aminophenyl)-1-phenyl-1*H*-pyrazol-4-yl)methylene)-2-thioxoimidazolidin-4-one **3g**

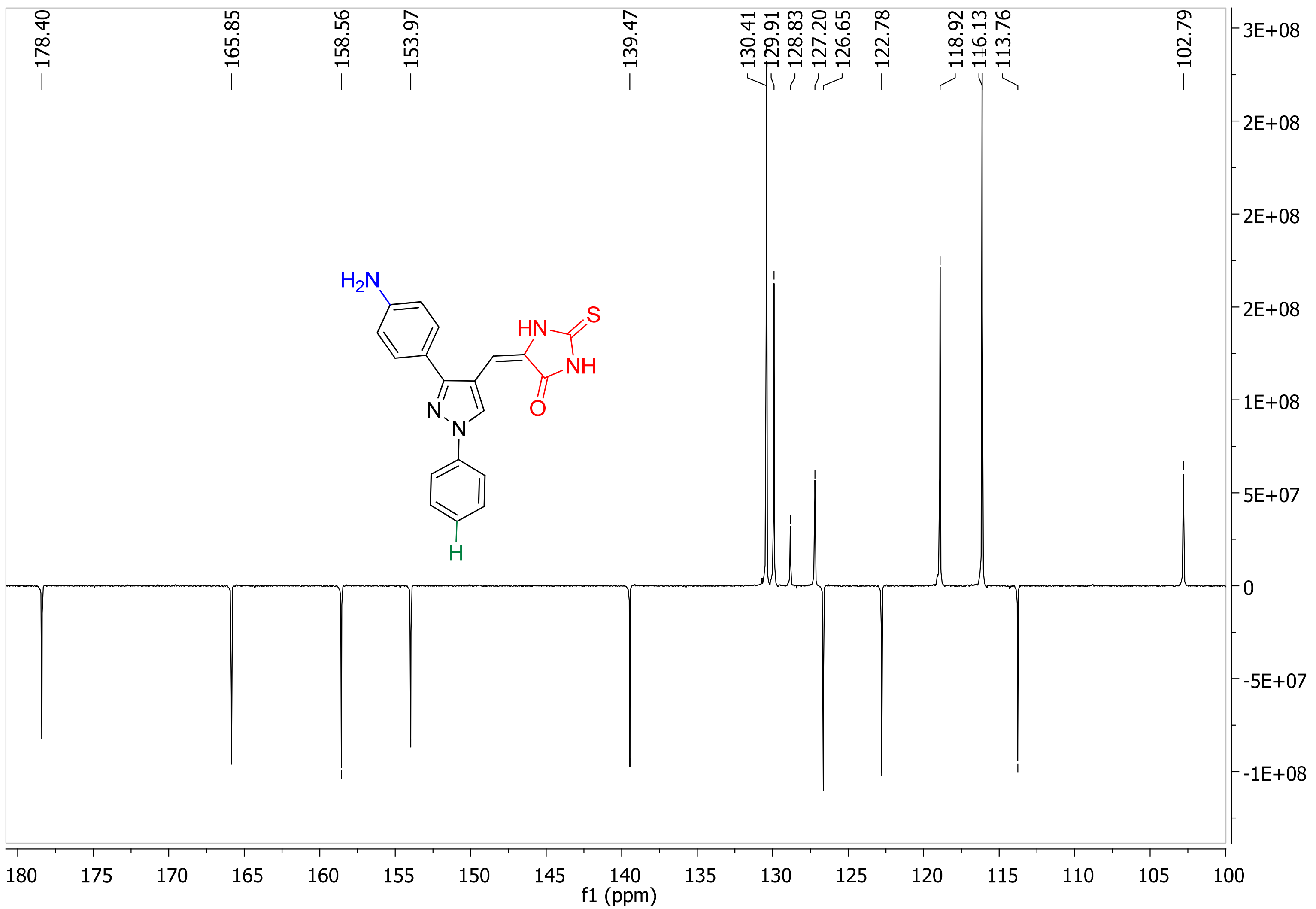

^{13}C APT NMR (DMSO-*d6*, 101 MHz) spectrum of 5-((3-(4-aminophenyl)-1-phenyl-1*H*-pyrazol-4-yl)methylene)-2-thioxoimidazolidin-4-one **3g**

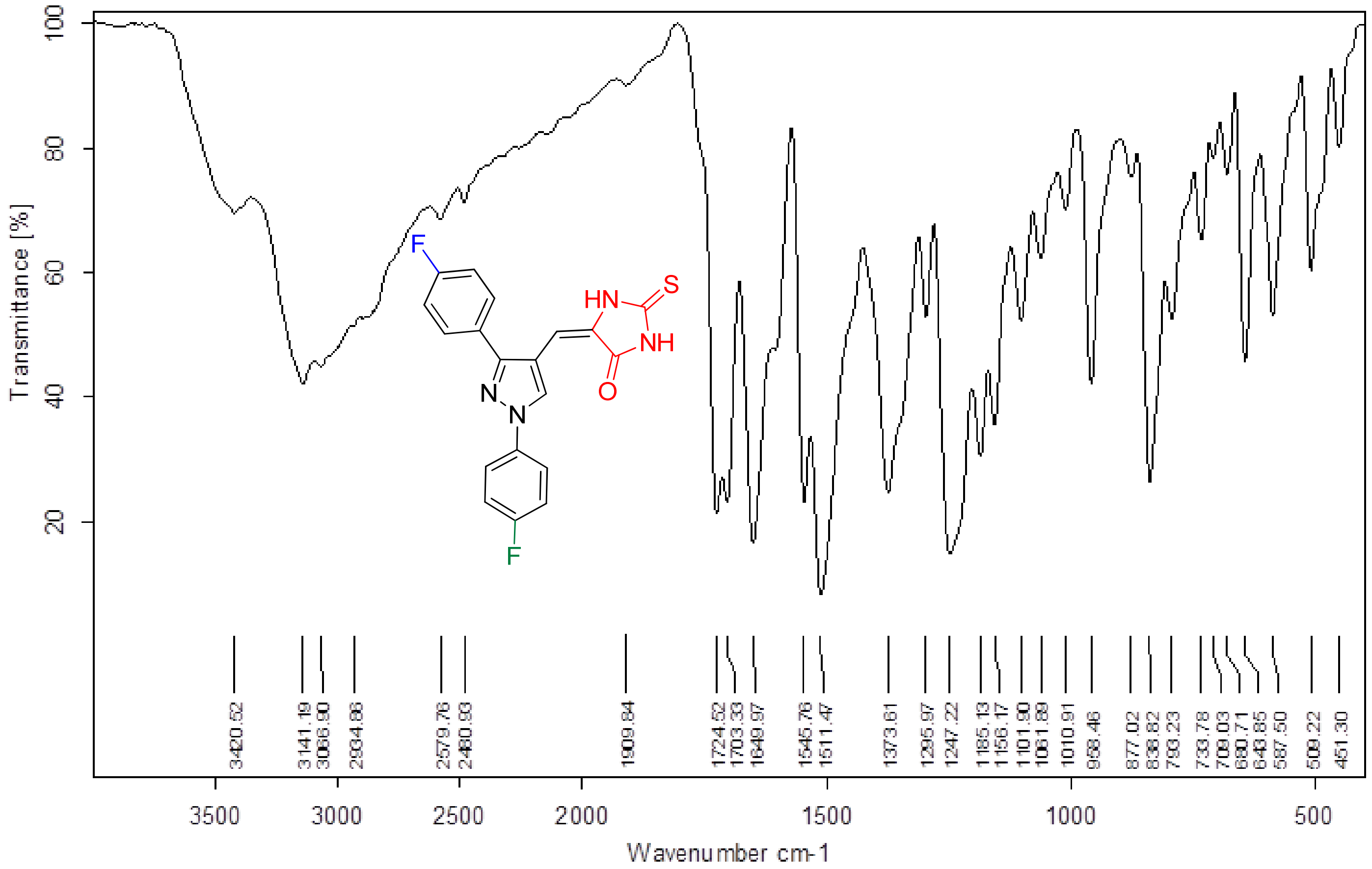

IR (KBr) spectrum of 5-((1,3-bis(4-fluorophenyl)-1*H*-pyrazol-4-yl)methylene)-2-thioxoimidazolidin-4-one **3h**

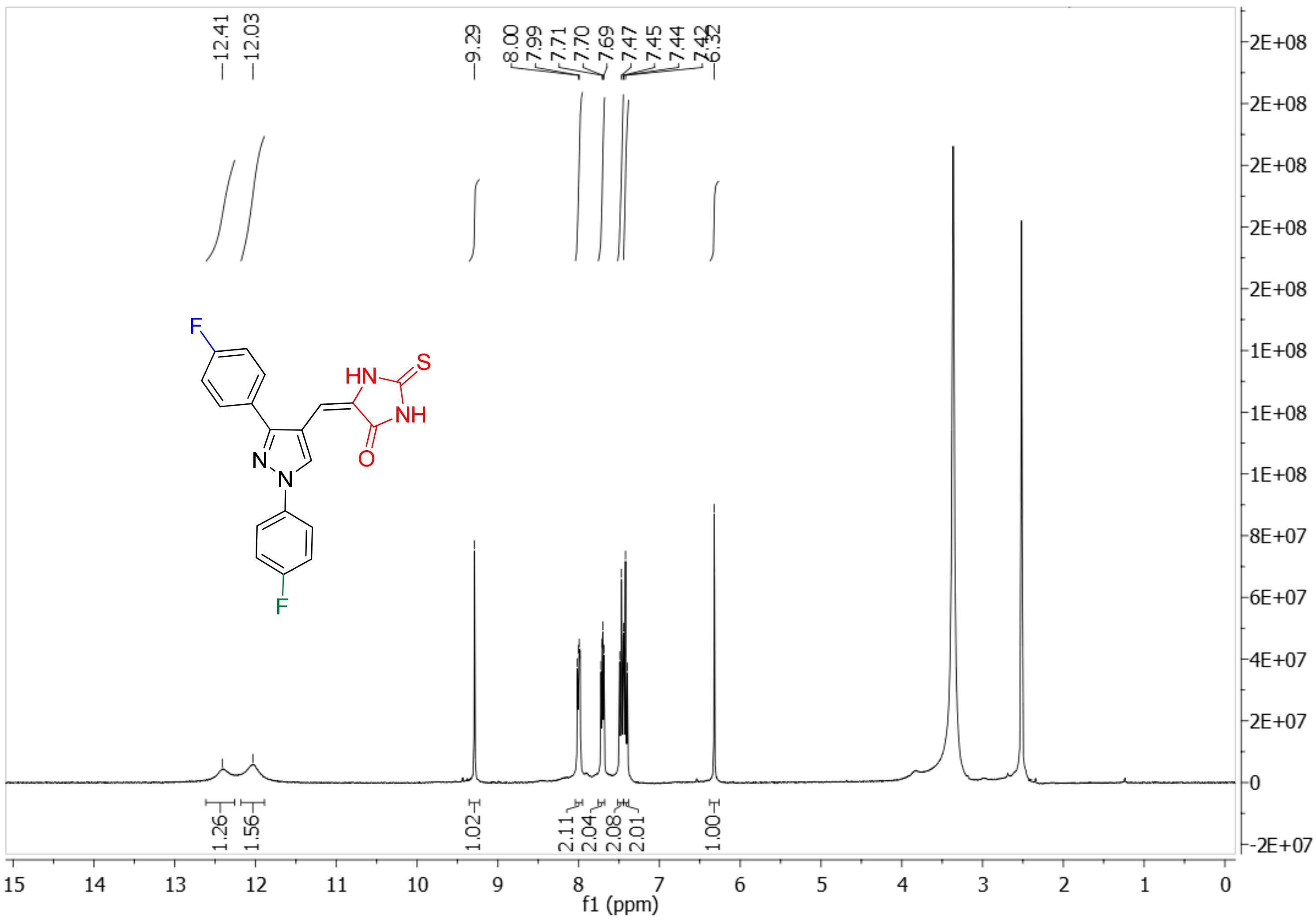

^{1}H NMR (DMSO-*d6*, 400 MHz) spectrum of 5-((1,3-bis(4-fluorophenyl)-1*H*-pyrazol-4-yl)methylene)-2-thioxoimidazolidin-4-one **3h**

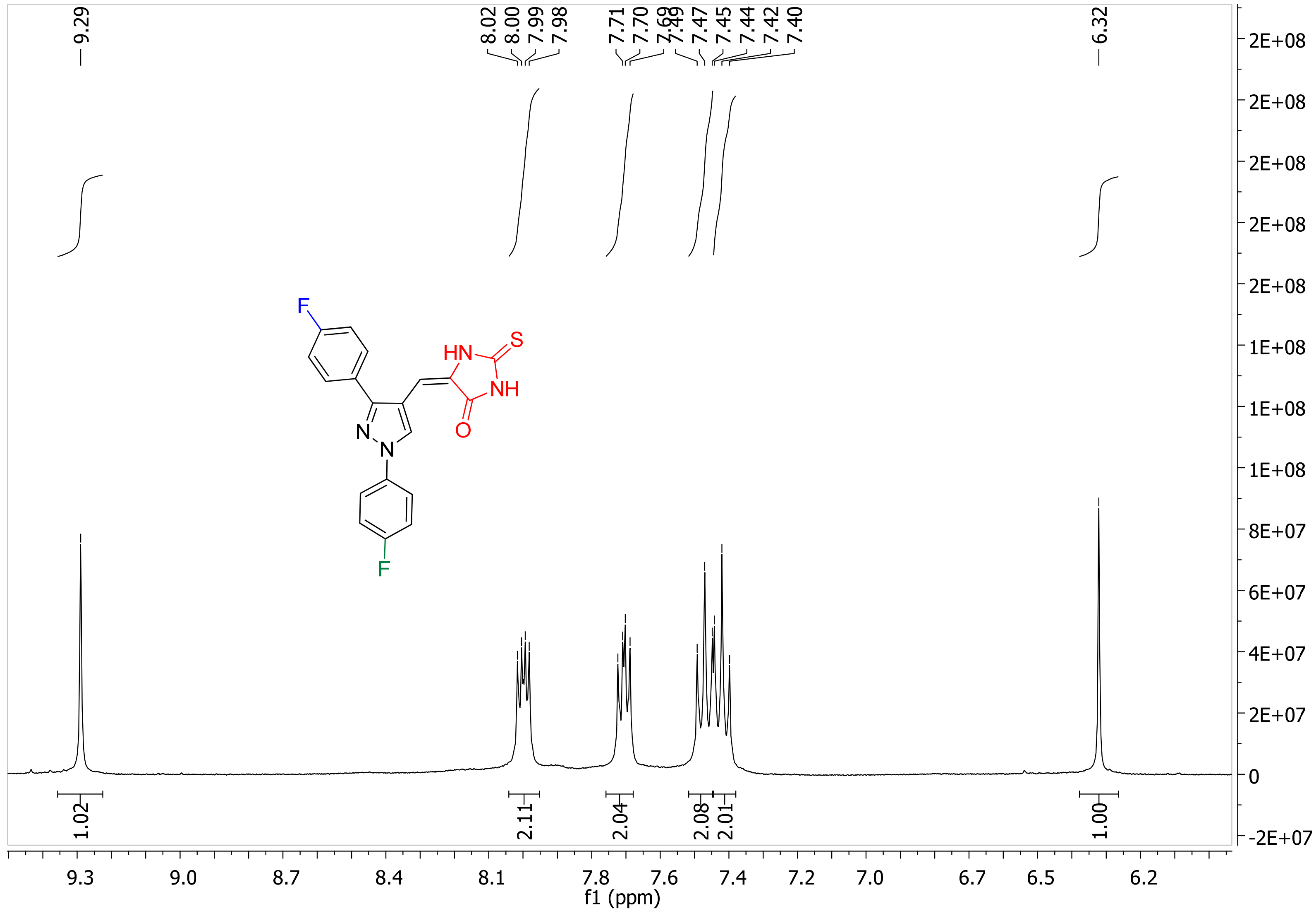

^{1}H NMR (DMSO-*d6*, 400 MHz) spectrum of 5-((1,3-bis(4-fluorophenyl)-1*H*-pyrazol-4-yl)methylene)-2-thioxoimidazolidin-4-one **3h**

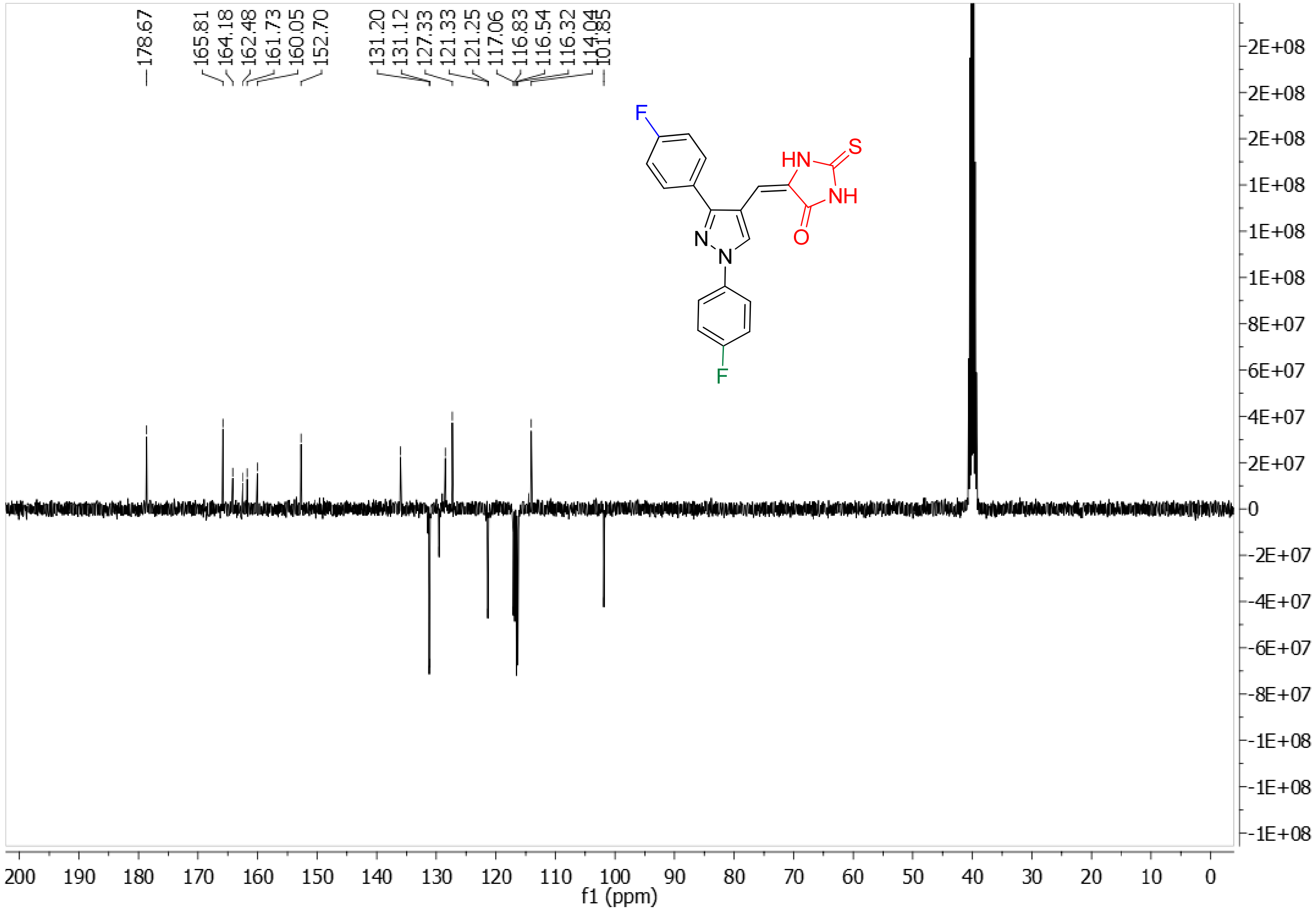

^{13}C APT NMR (DMSO-*d6*, 101 MHz) spectrum of 5-((1,3-bis(4-fluorophenyl)-1*H*-pyrazol-4-yl)methylene)-2-thioxoimidazolidin-4-one **3h**

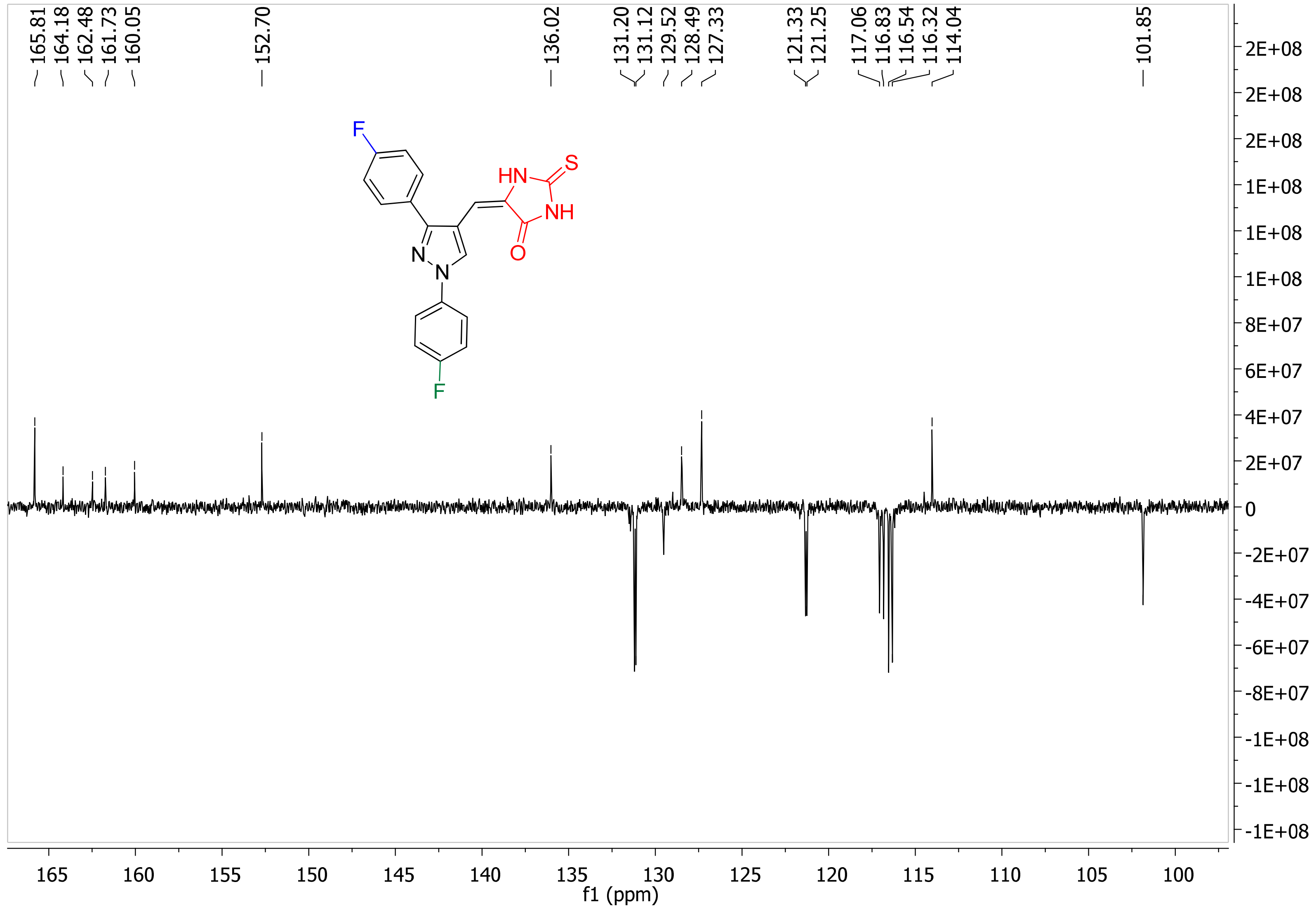

^{13}C APT NMR (DMSO-*d6*, 101 MHz) spectrum of 5-((1,3-bis(4-fluorophenyl)-1*H*-pyrazol-4-yl)methylene)-2-thioxoimidazolidin-4-one **3h**

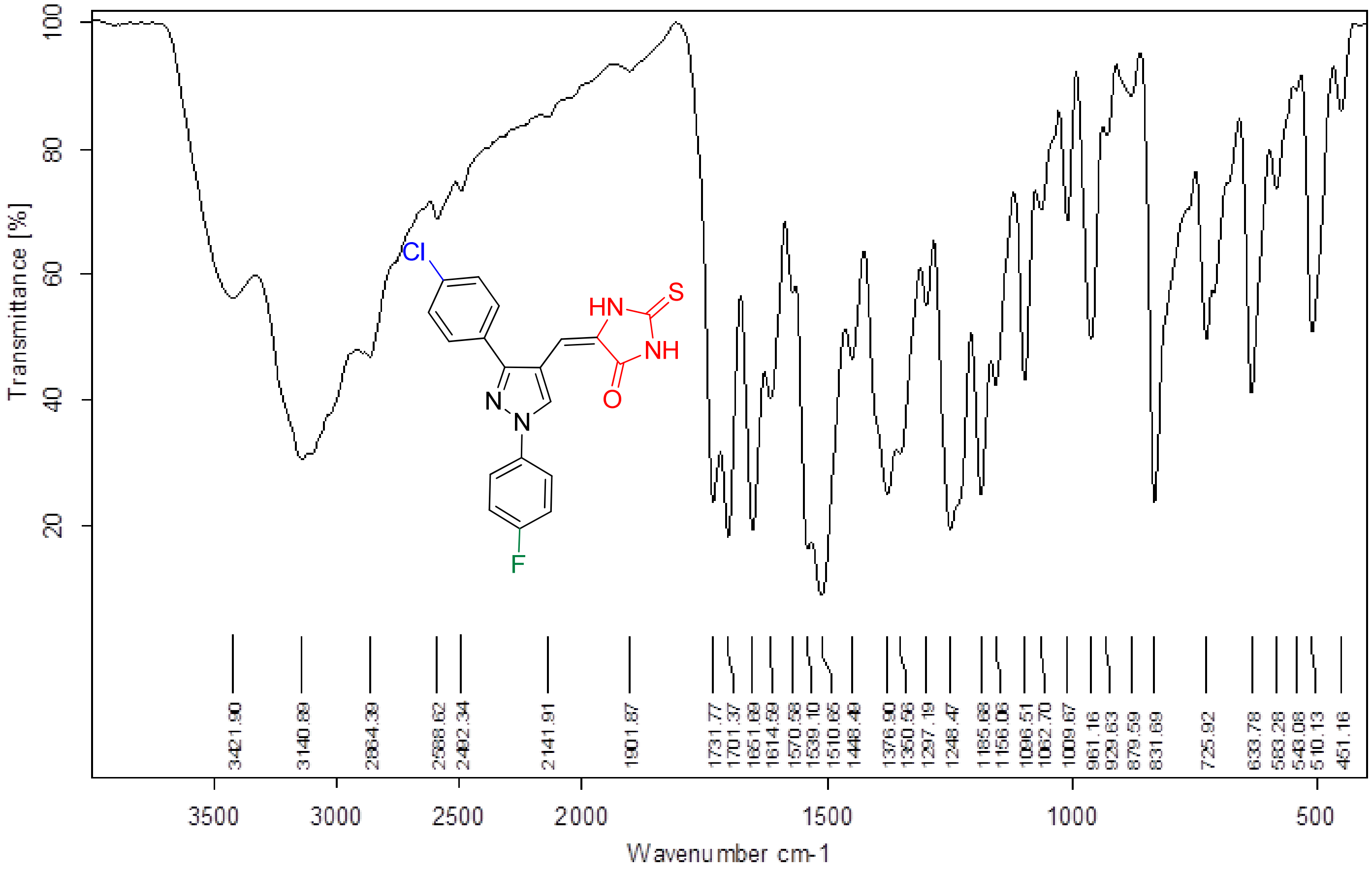

IR (KBr) spectrum of 5-((3-(4-chlorophenyl)-1-(4-fluorophenyl)-1*H*-pyrazol-4-yl)methylene)-2-thioxoimidazolidin-4-one **3i**

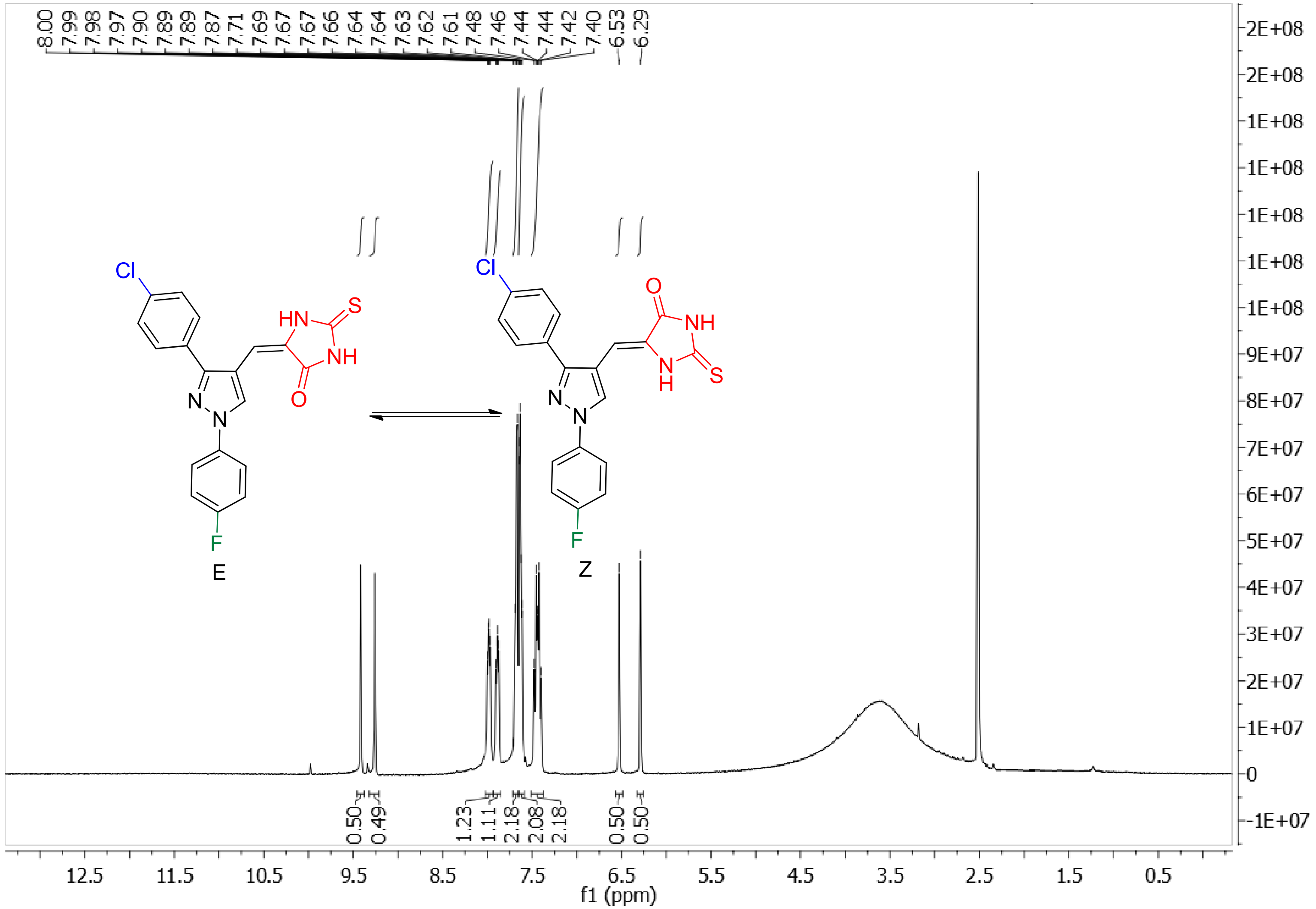

^{1}H NMR (DMSO-*d6*, 400 MHz) spectrum of 5-((3-(4-chlorophenyl)-1-(4-fluorophenyl)-1*H*-pyrazol-4-yl)methylene)-2-thioxoimidazolidin-4-one **3i**

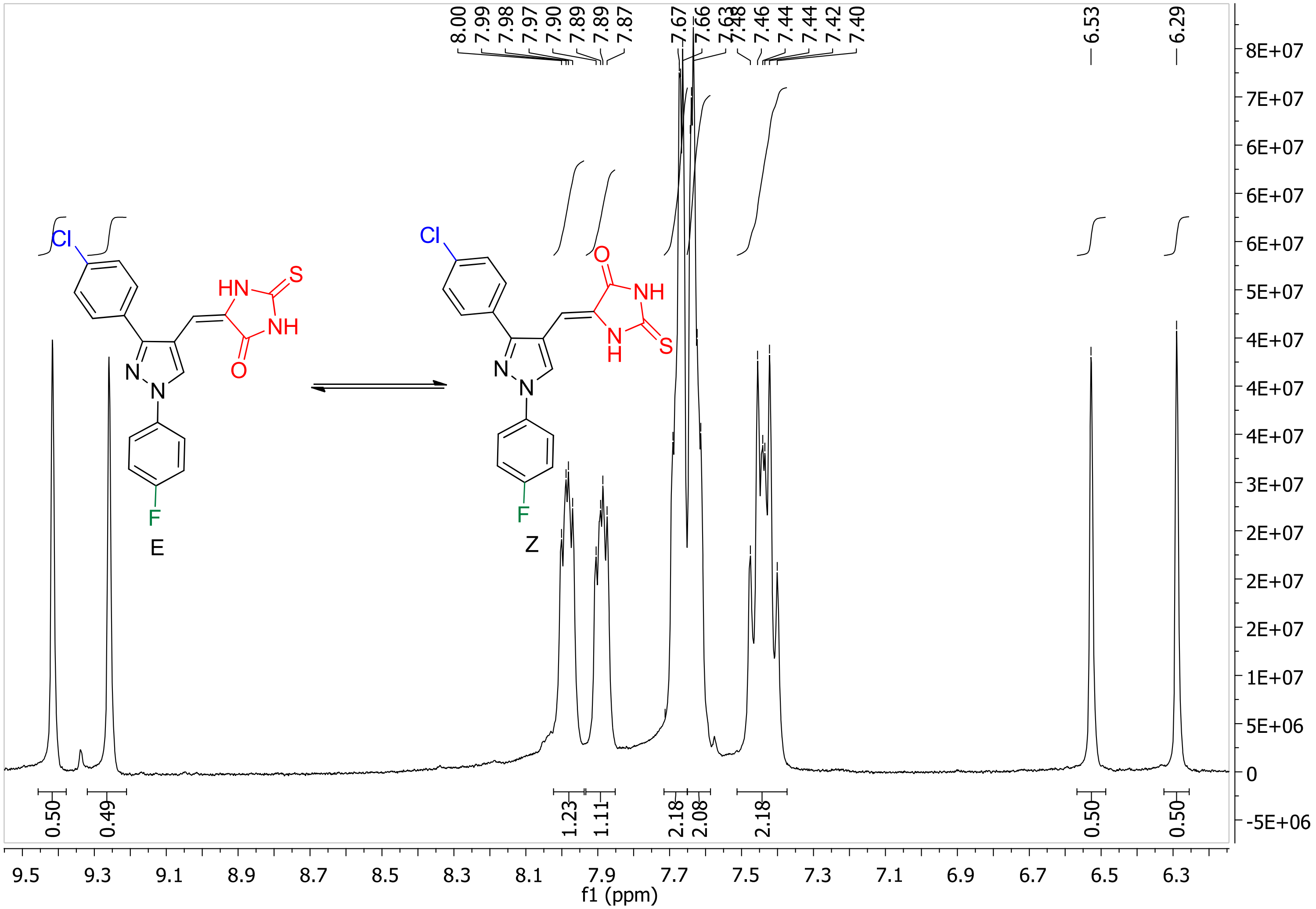

^{1}H NMR (DMSO-*d6*, 400 MHz) spectrum of 5-((3-(4-chlorophenyl)-1-(4-fluorophenyl)-1*H*-pyrazol-4-yl)methylene)-2-thioxoimidazolidin-4-one **3i**

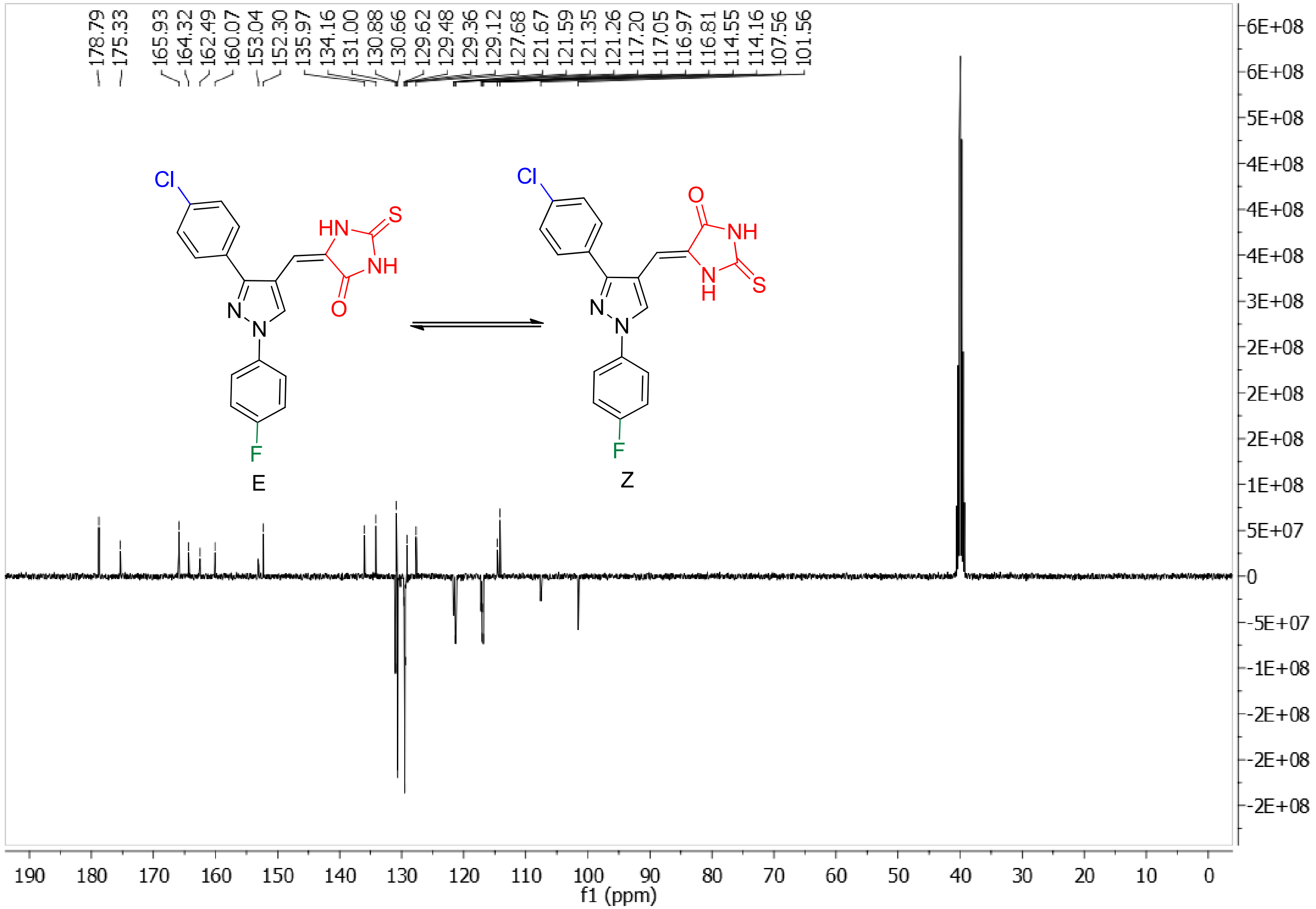

^{13}C APT NMR (DMSO-*d6*, 101 MHz) spectrum of 5-((3-(4-chlorophenyl)-1-(4-fluorophenyl)-1*H*-pyrazol-4-yl)methylene)-2-thioxoimidazolidin-4-one **3i**

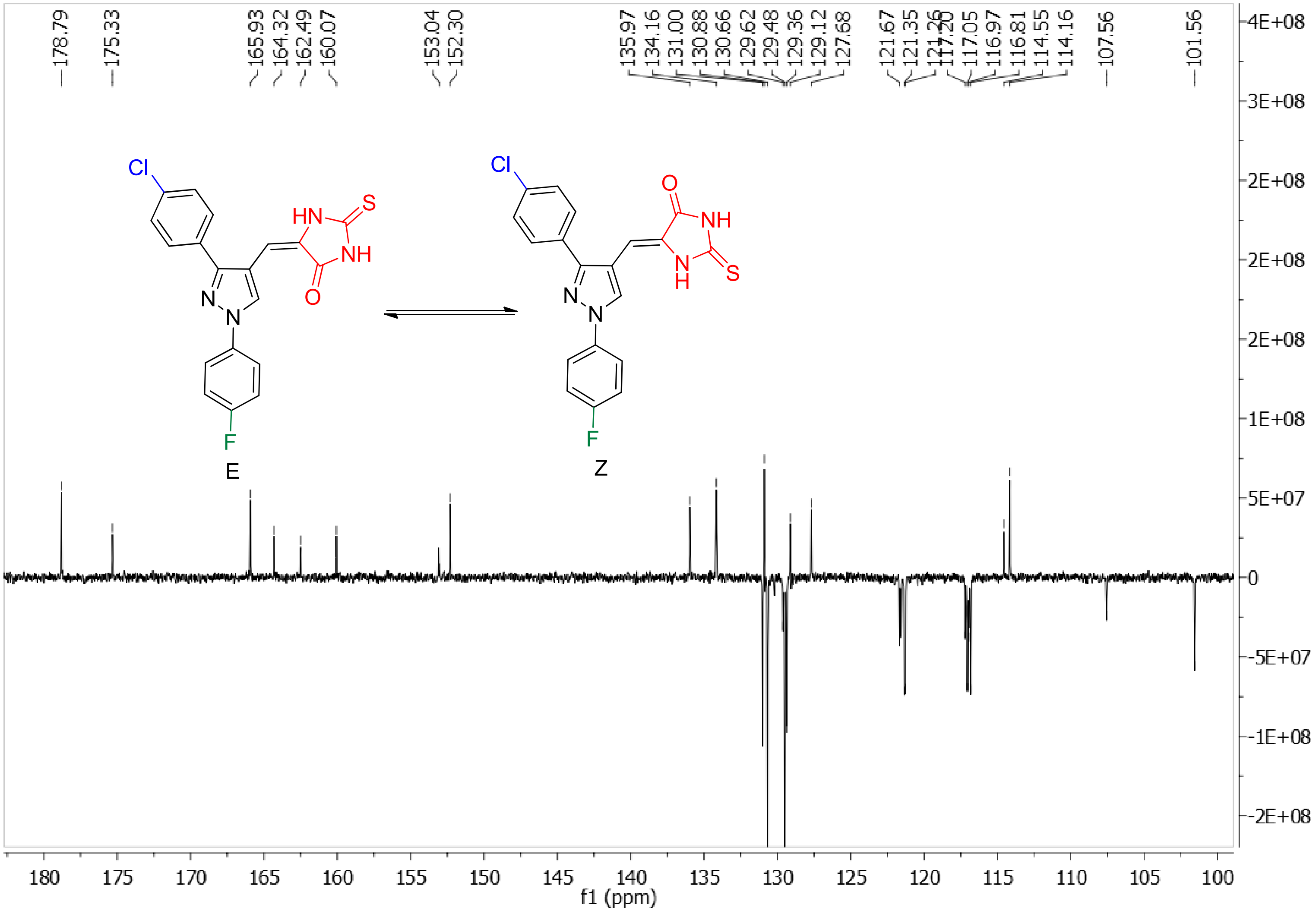

^{13}C APT NMR (DMSO-*d6*, 101 MHz) spectrum of 5-((3-(4-chlorophenyl)-1-(4-fluorophenyl)-1*H*-pyrazol-4-yl)methylene)-2-thioxoimidazolidin-4-one **3i**

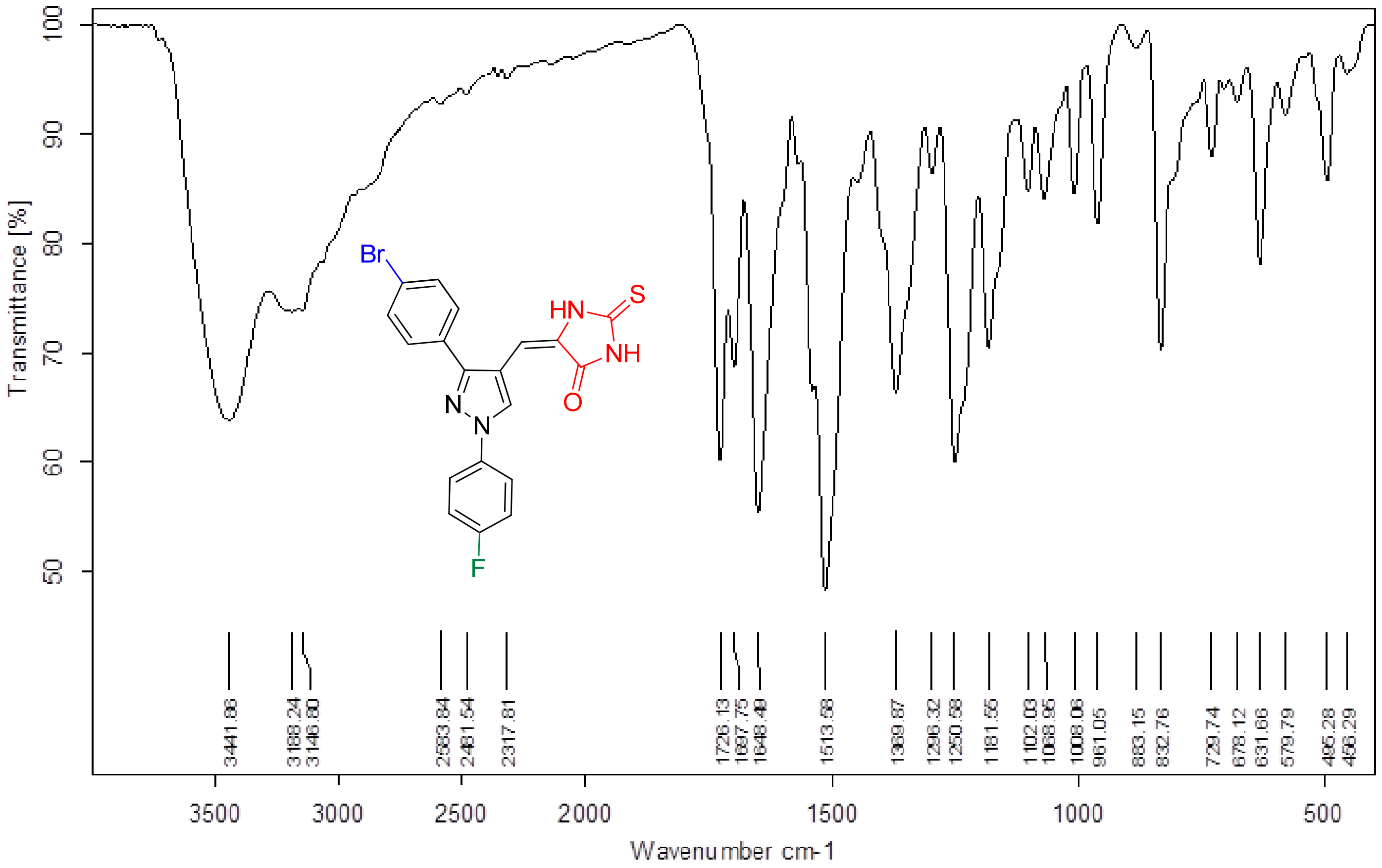

IR (KBr) spectrum of 5-((3-(4-bromophenyl)-1-(4-fluorophenyl)-1*H*-pyrazol-4-yl)methylene)-2-thioxoimidazolidin-4-one **3j**

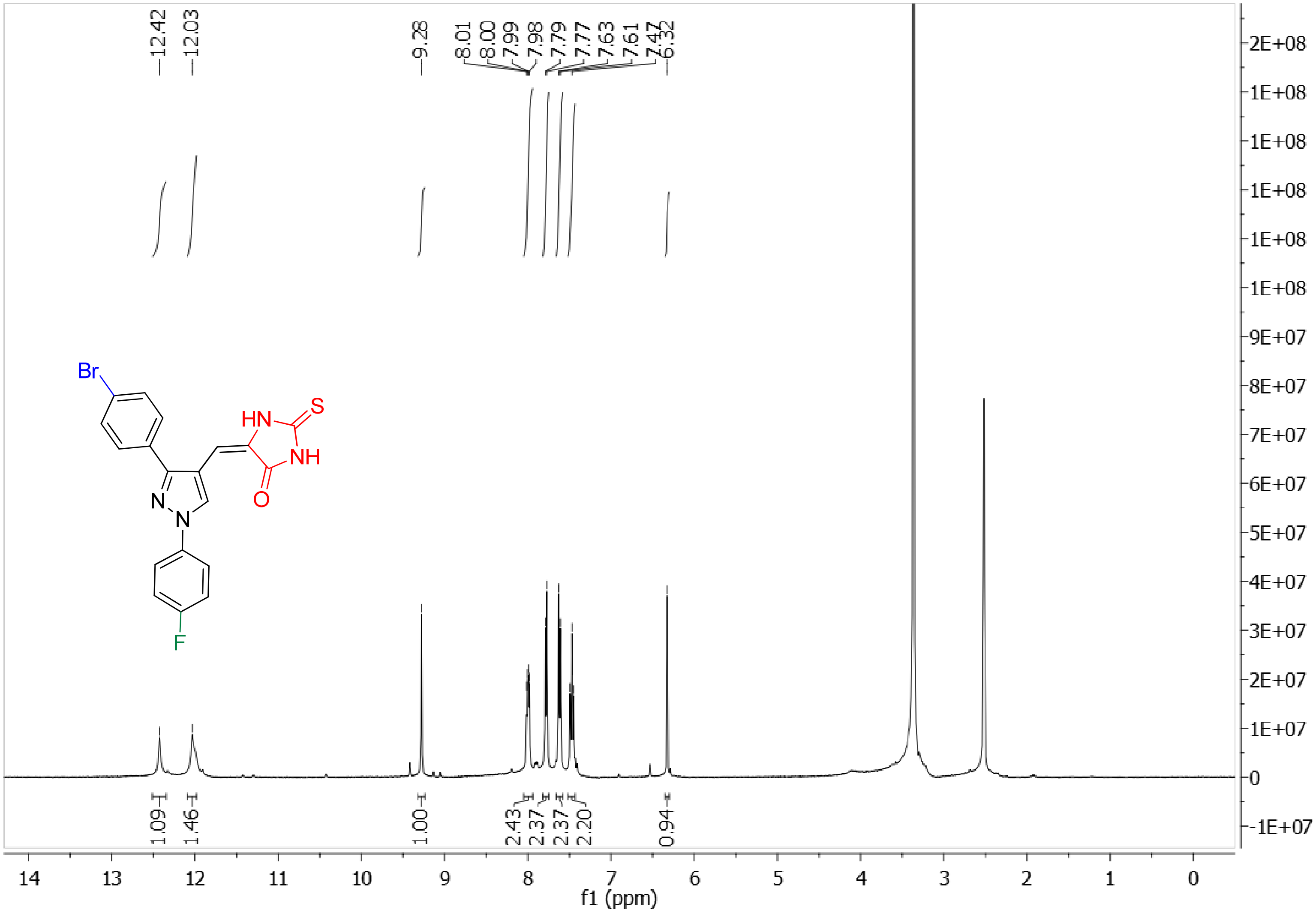

^{1}H NMR (DMSO-*d6*, 400 MHz) spectrum of 5-((3-(4-bromophenyl)-1-(4-fluorophenyl)-1*H*-pyrazol-4-yl)methylene)-2-thioxoimidazolidin-4-one **3j**

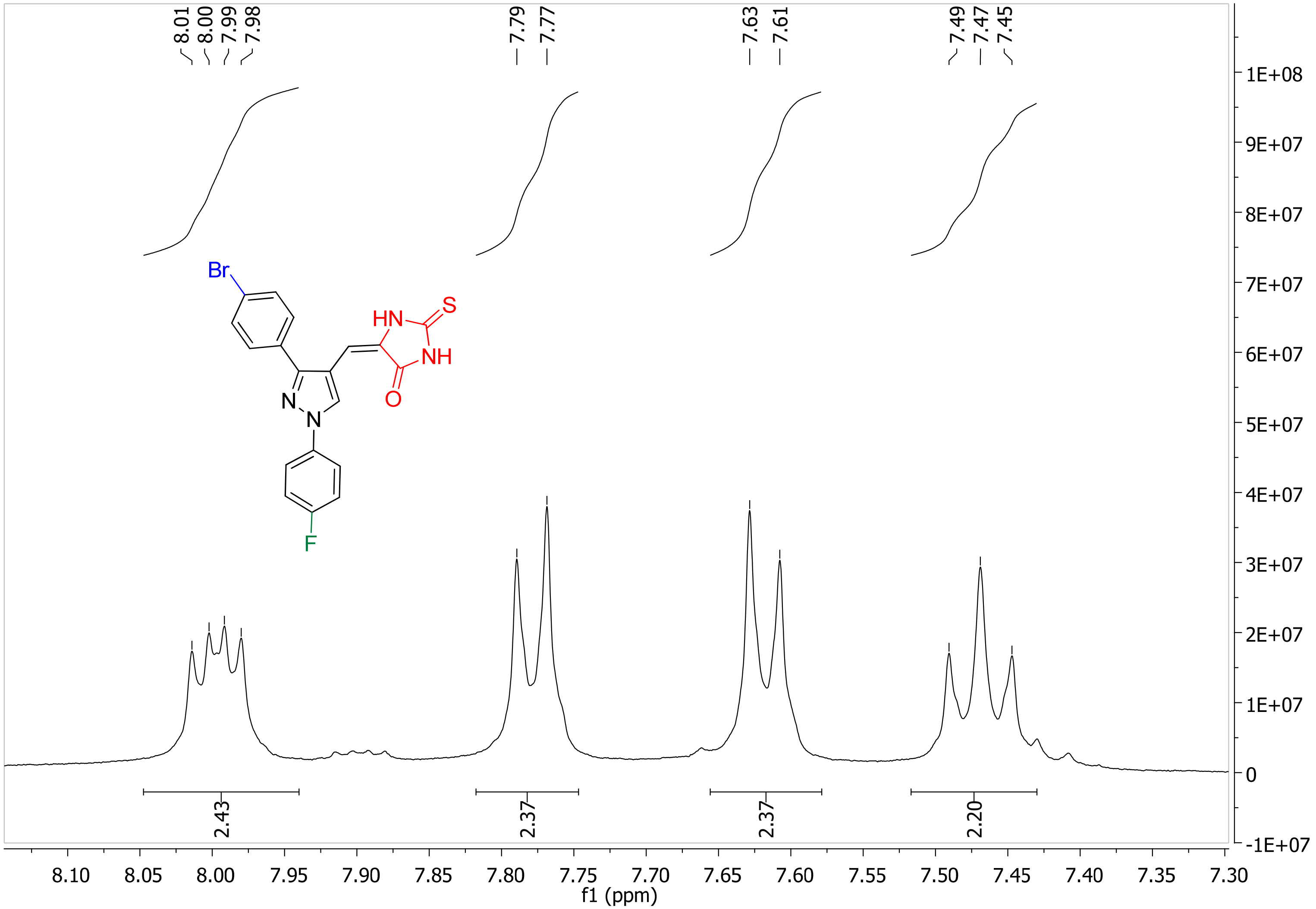

^{1}H NMR (DMSO-*d6*, 400 MHz) spectrum of 5-((3-(4-bromophenyl)-1-(4-fluorophenyl)-1*H*-pyrazol-4-yl)methylene)-2-thioxoimidazolidin-4-one **3j**

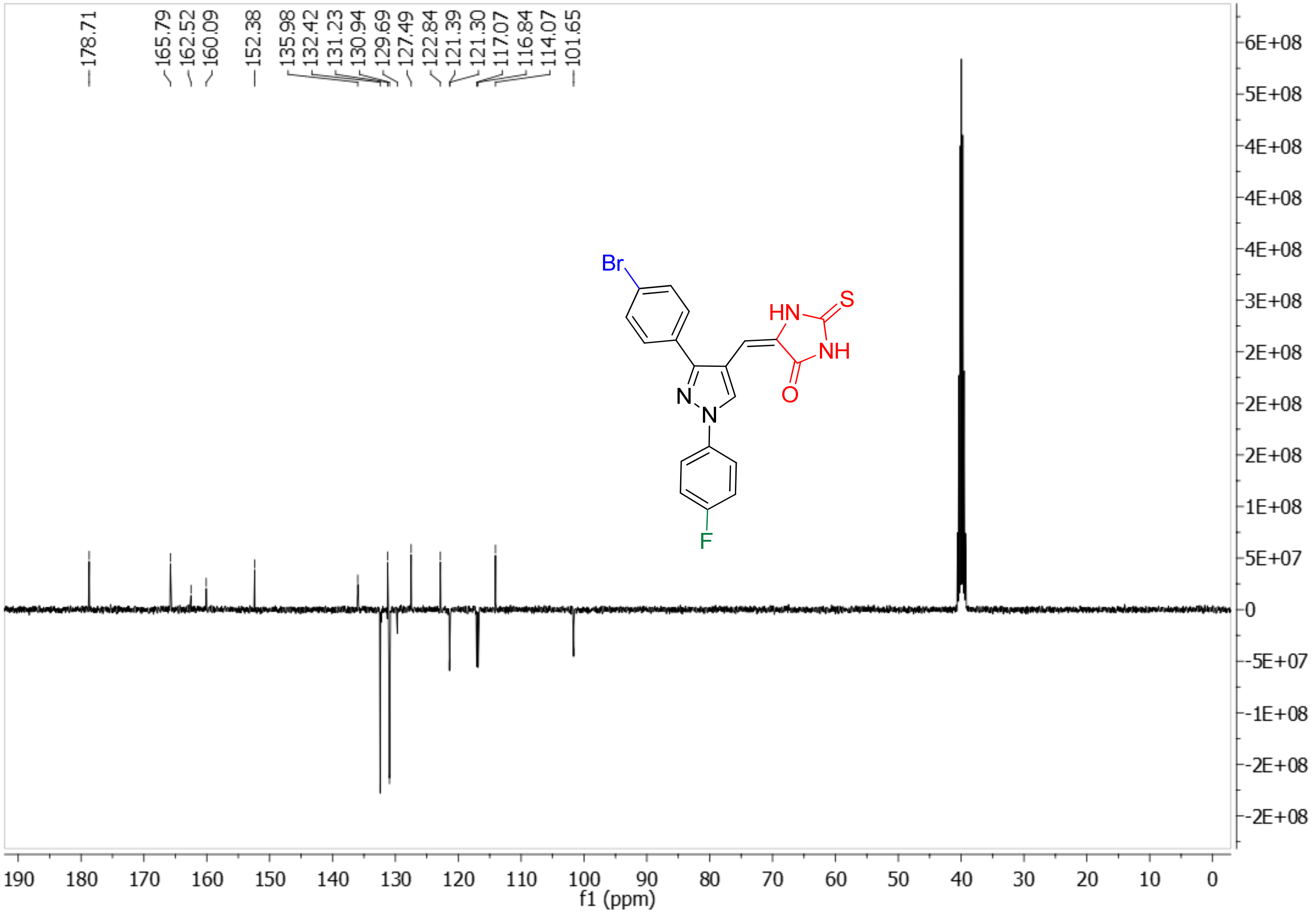

^{13}C APT NMR (DMSO-*d6*, 101 MHz) spectrum of 5-((3-(4-bromophenyl)-1-(4-fluorophenyl)-1*H*-pyrazol-4-yl)methylene)-2-thioxoimidazolidin-4-one **3j**

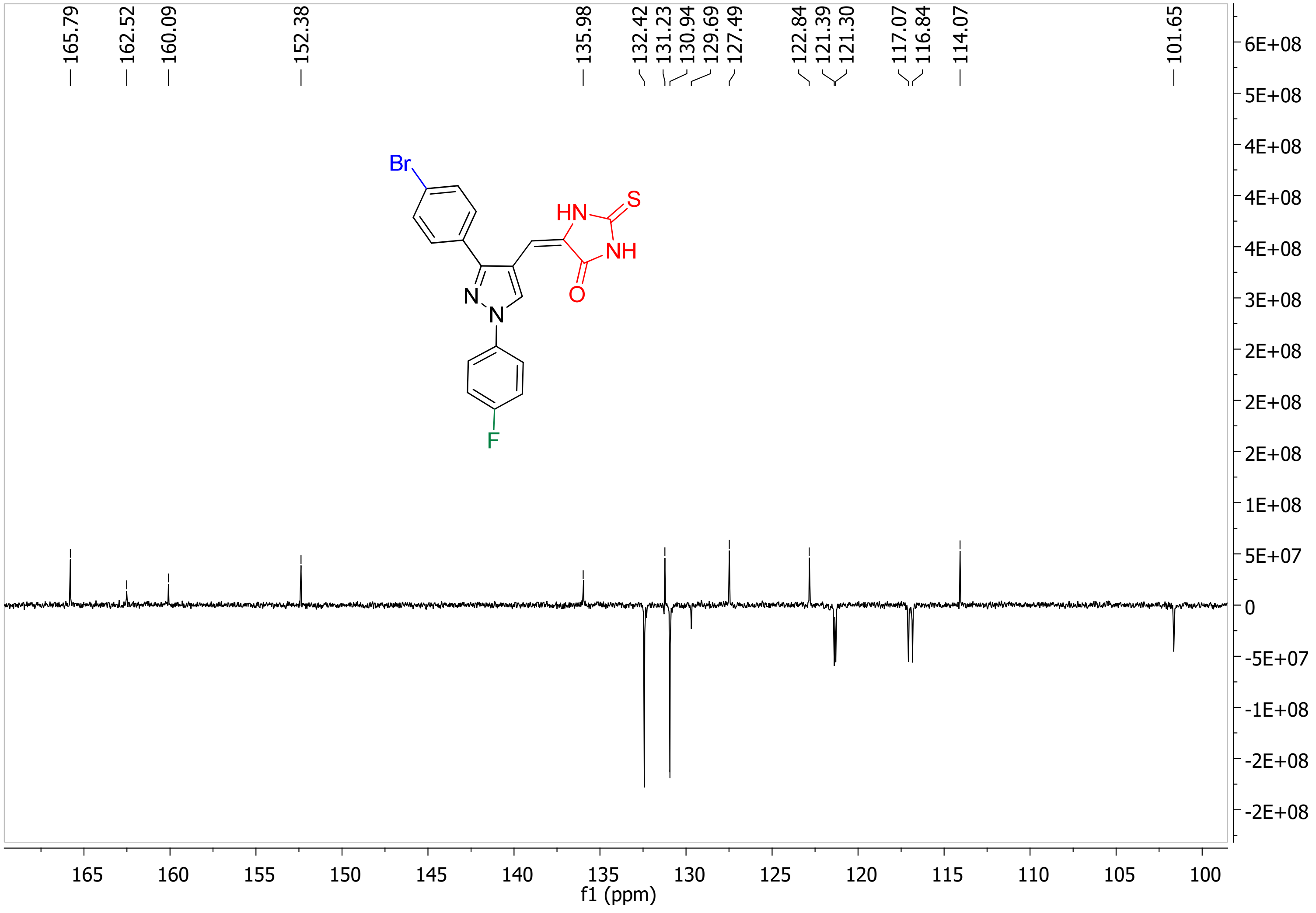

^{13}C APT NMR (DMSO-*d6*, 101 MHz) spectrum of 5-((3-(4-bromophenyl)-1-(4-fluorophenyl)-1*H*-pyrazol-4-yl)methylene)-2-thioxoimidazolidin-4-one **3j**

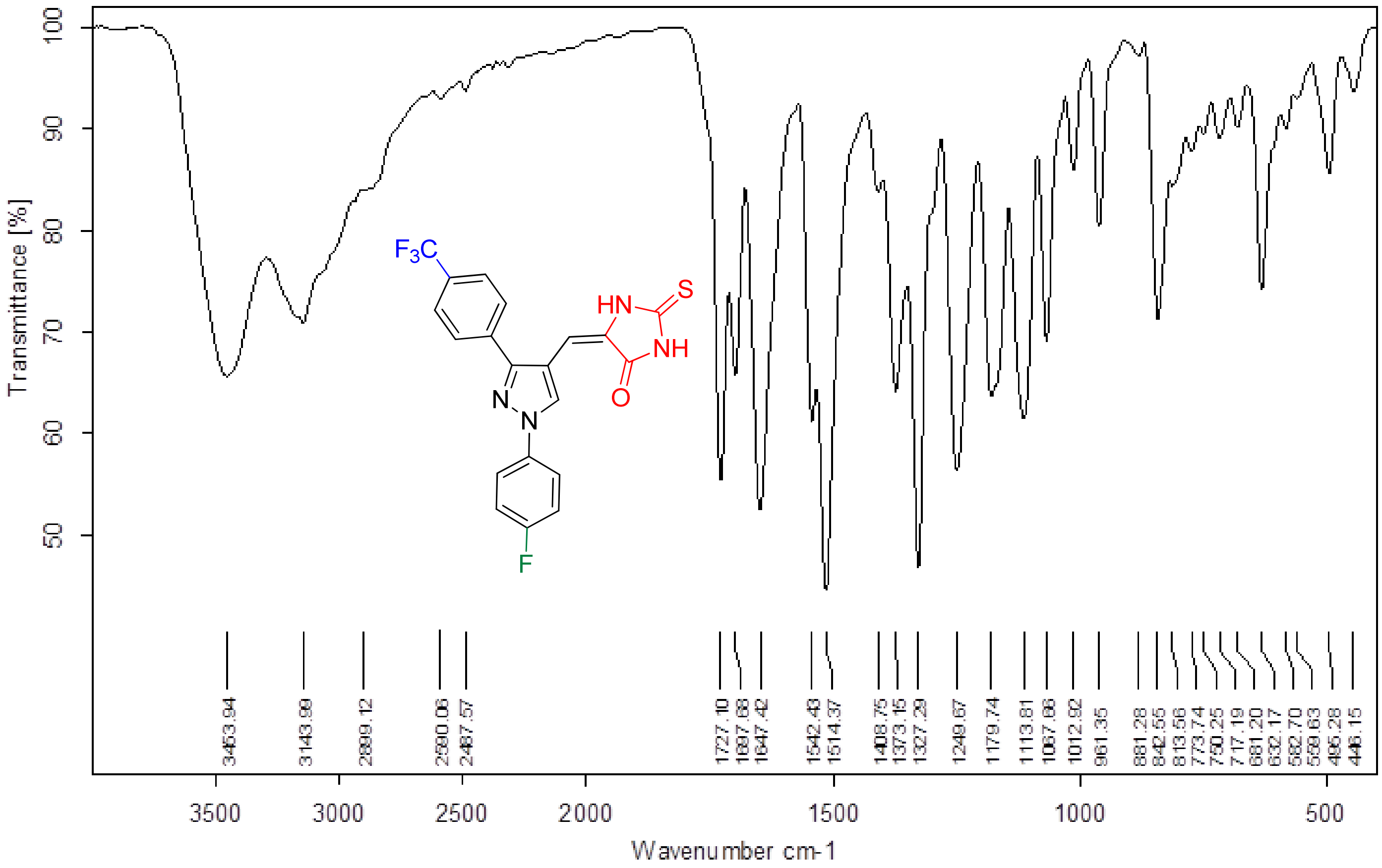

IR (KBr) spectrum of 5-((1-(4-fluorophenyl)-3-(4-(trifluoromethyl)phenyl)-1*H*-pyrazol-4-yl)methylene)-2-thioxoimidazolidin-4-one **3k**

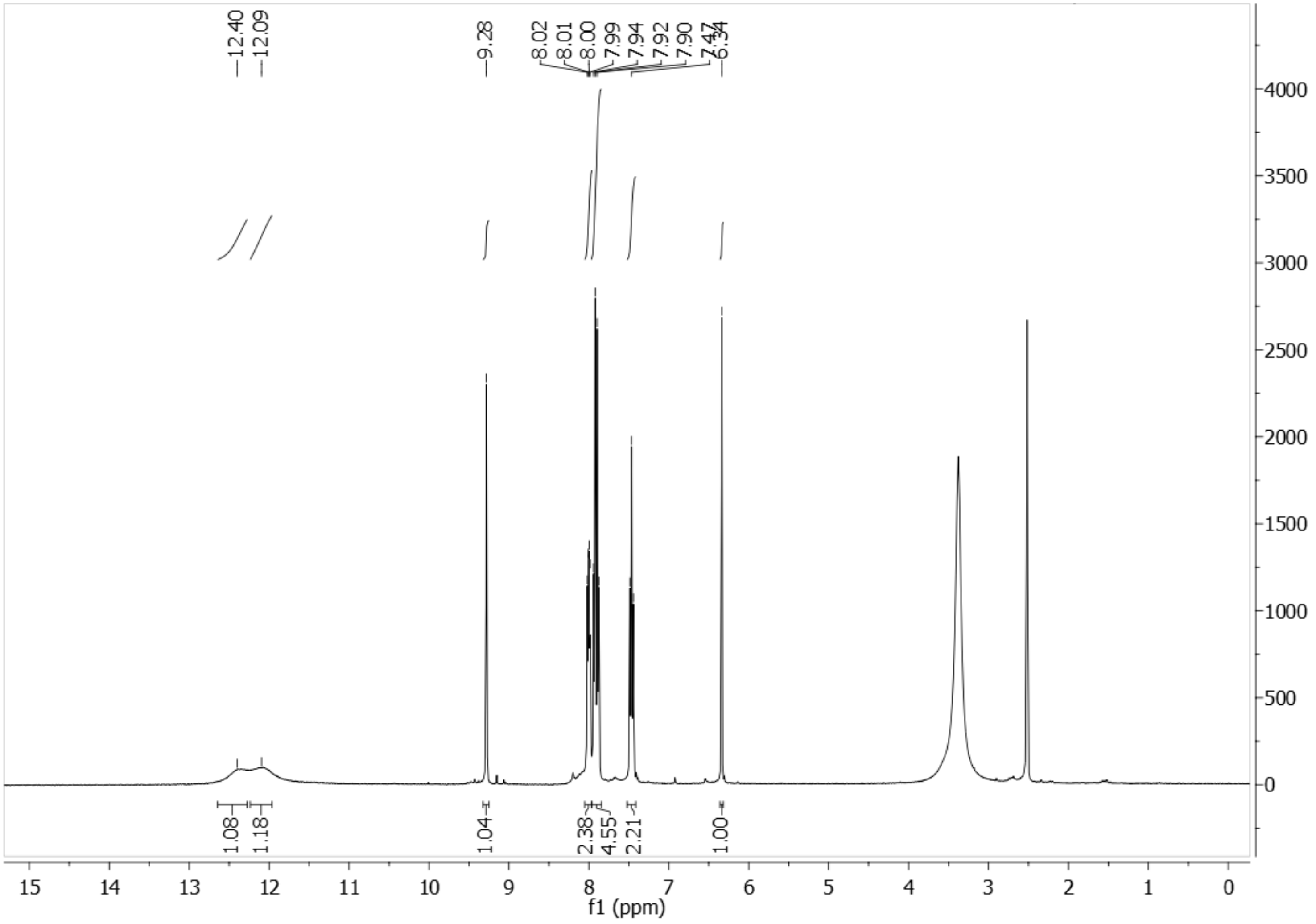

^{1}H NMR (DMSO-*d6*, 400 MHz) spectrum of 5-((1-(4-fluorophenyl)-3-(4-(trifluoromethyl)phenyl)-1*H*-pyrazol-4-yl)methylene)-2-thioxoimidazolidin-4-one **3k**

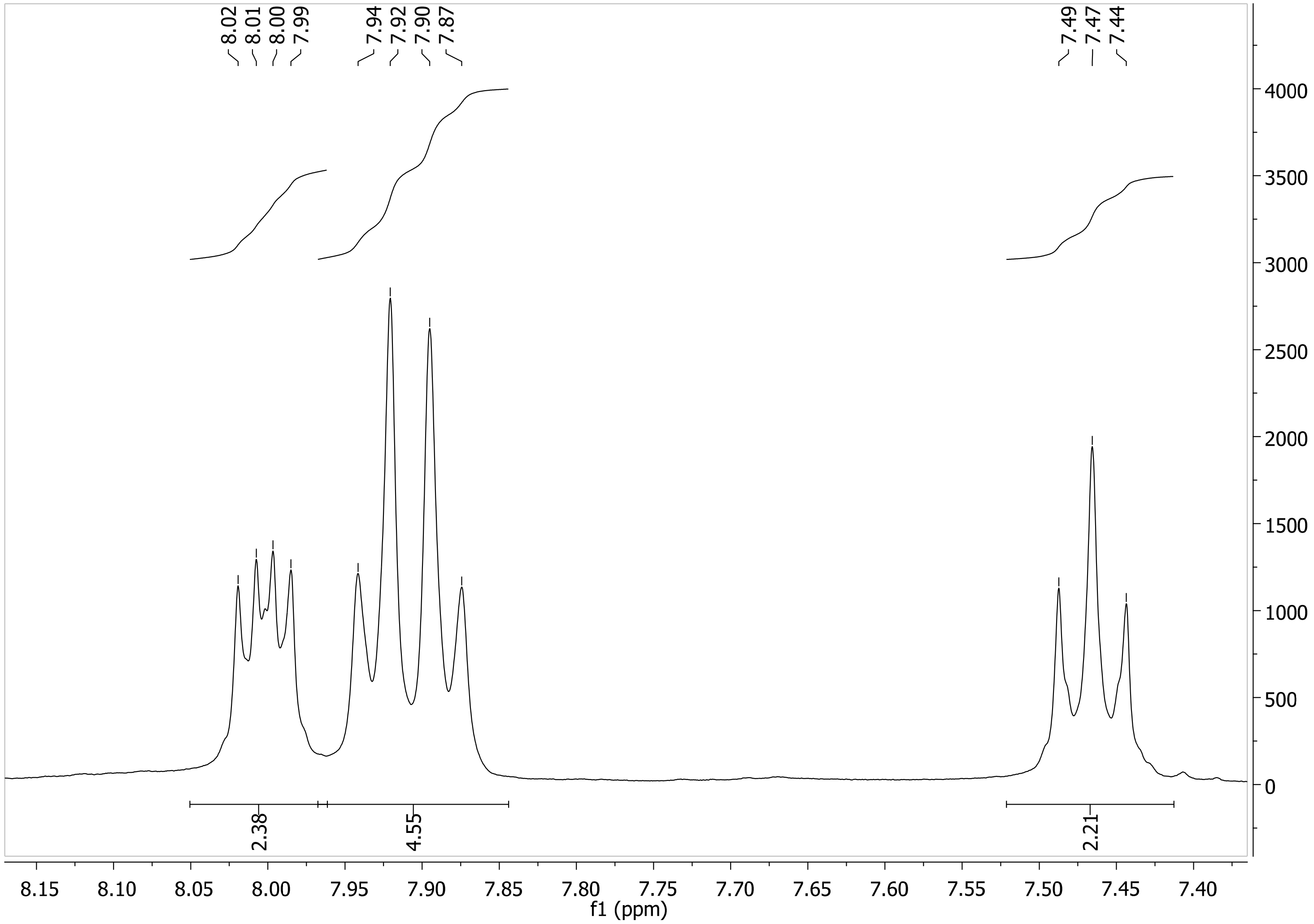

^{1}H NMR (DMSO-*d6*, 400 MHz) spectrum of 5-((1-(4-fluorophenyl)-3-(4-(trifluoromethyl)phenyl)-1*H*-pyrazol-4-yl)methylene)-2-thioxoimidazolidin-4-one **3k**

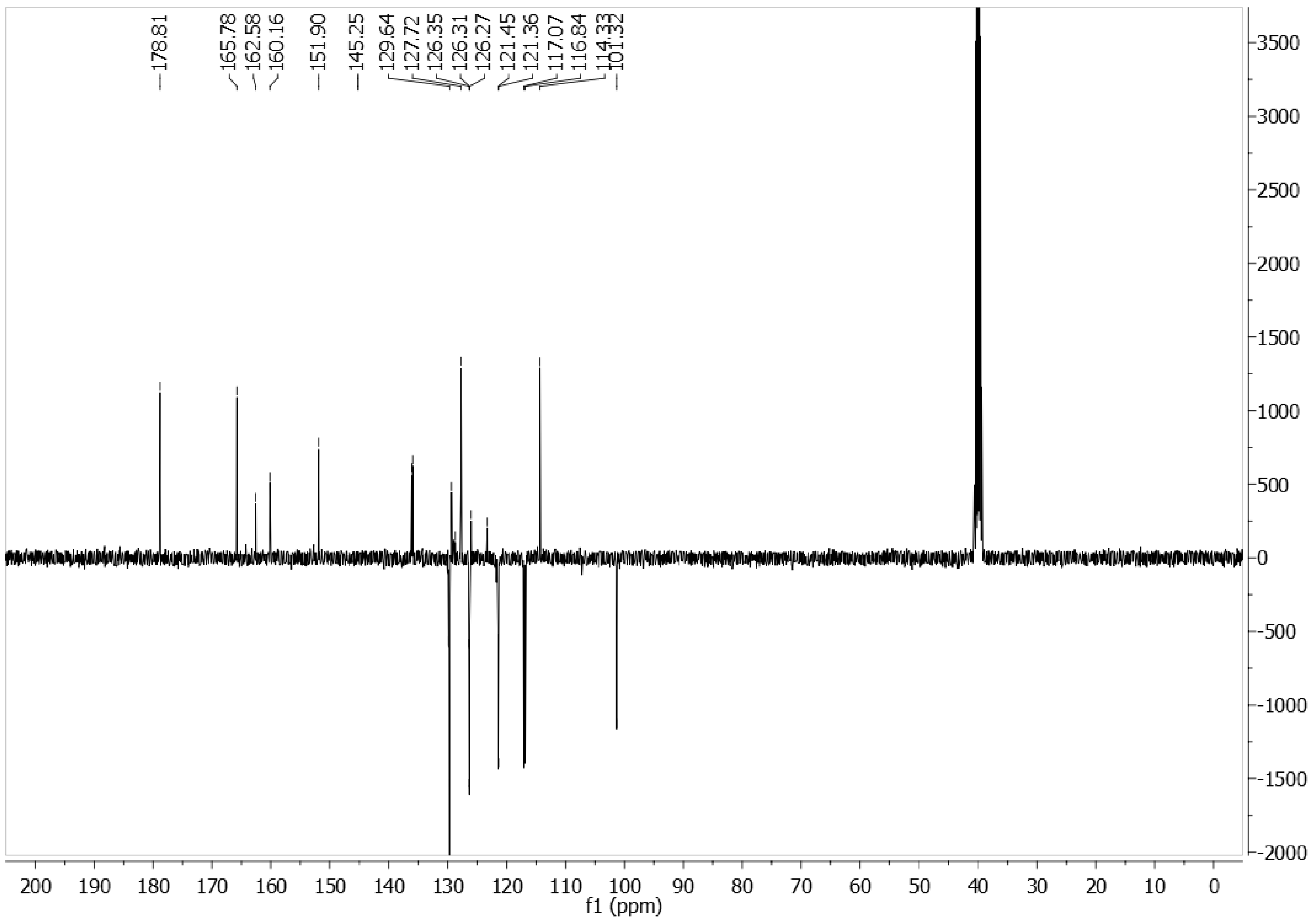

^{13}C APT NMR (DMSO-*d6*, 101 MHz) spectrum of 5-((1-(4-fluorophenyl)-3-(4-(trifluoromethyl)phenyl)-1*H*-pyrazol-4-yl)methylene)-2-thioxoimidazolidin-4-one **3k**

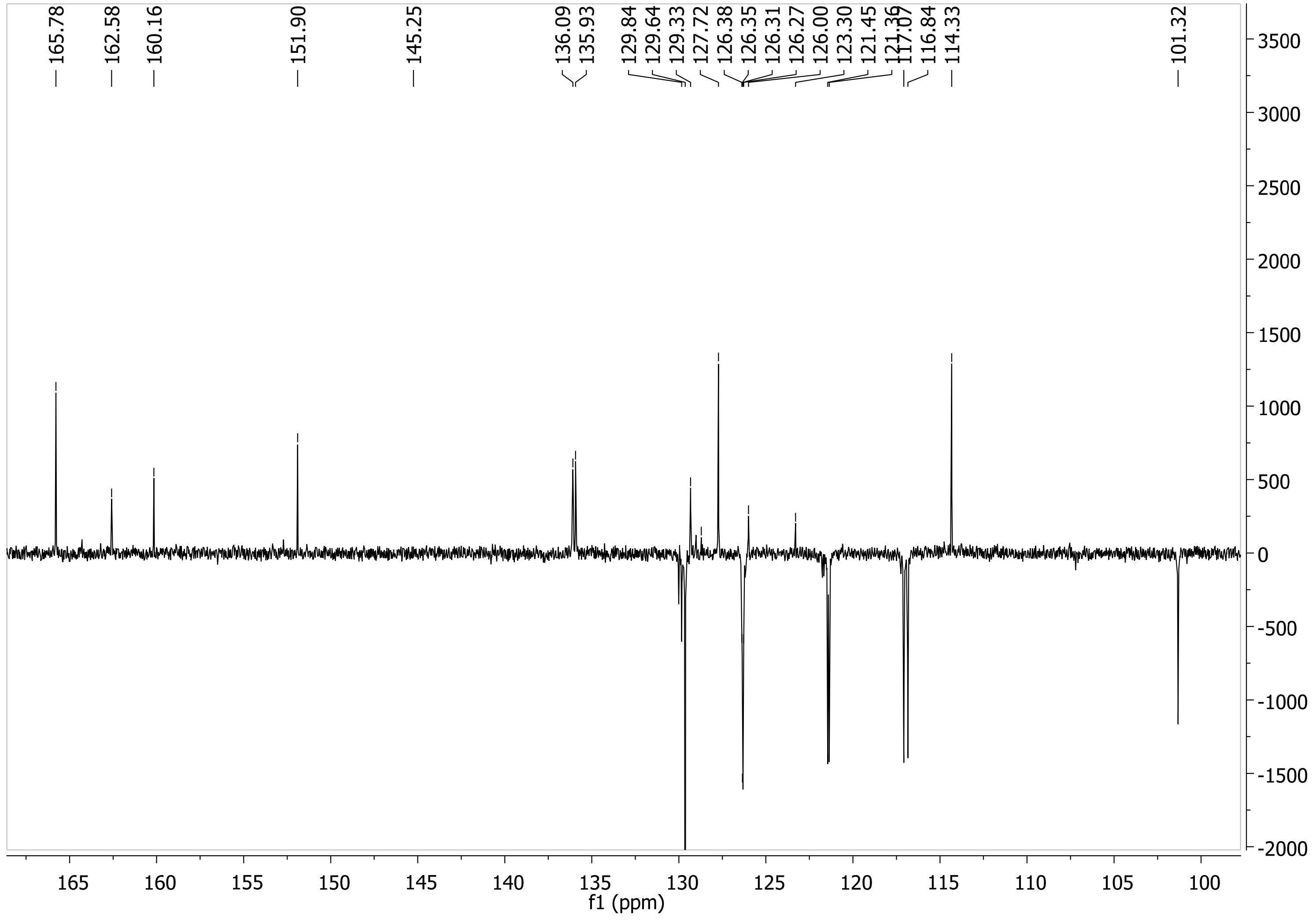

^{13}C APT NMR (DMSO-*d6*, 101 MHz) spectrum of 5-((1-(4-fluorophenyl)-3-(4-(trifluoromethyl)phenyl)-1*H*-pyrazol-4-yl)methylene)-2-thioxoimidazolidin-4-one **3k**

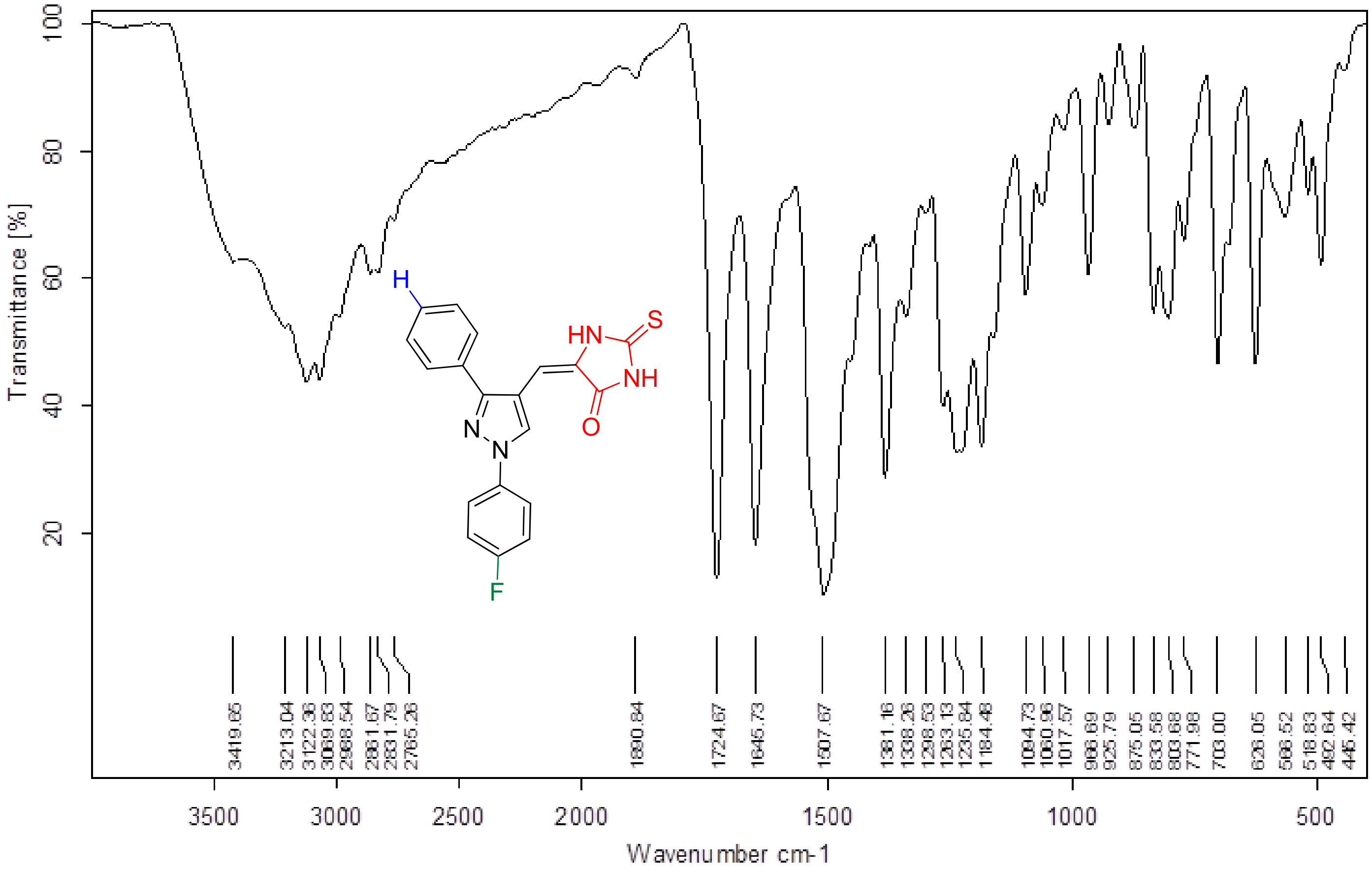

IR (KBr) spectrum of 5-((1-(4-fluorophenyl)-3-phenyl-1*H*-pyrazol-4-yl)methylene)-2-thioxoimidazolidin-4-one **3l**

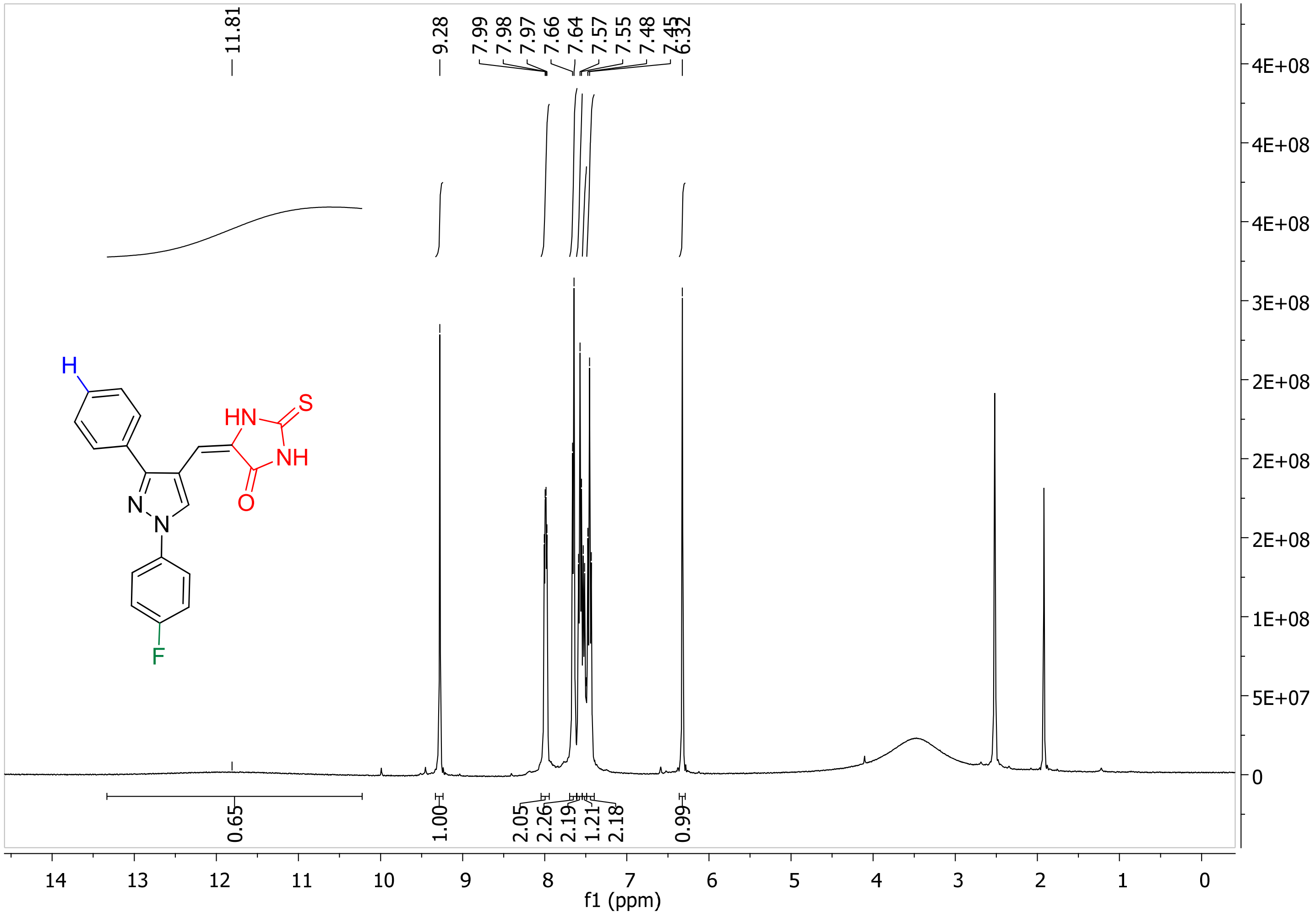

^{1}H NMR (DMSO-*d6*, 400 MHz) spectrum of 5-((1-(4-fluorophenyl)-3-phenyl-1*H*-pyrazol-4-yl)methylene)-2-thioxoimidazolidin-4-one **3l**

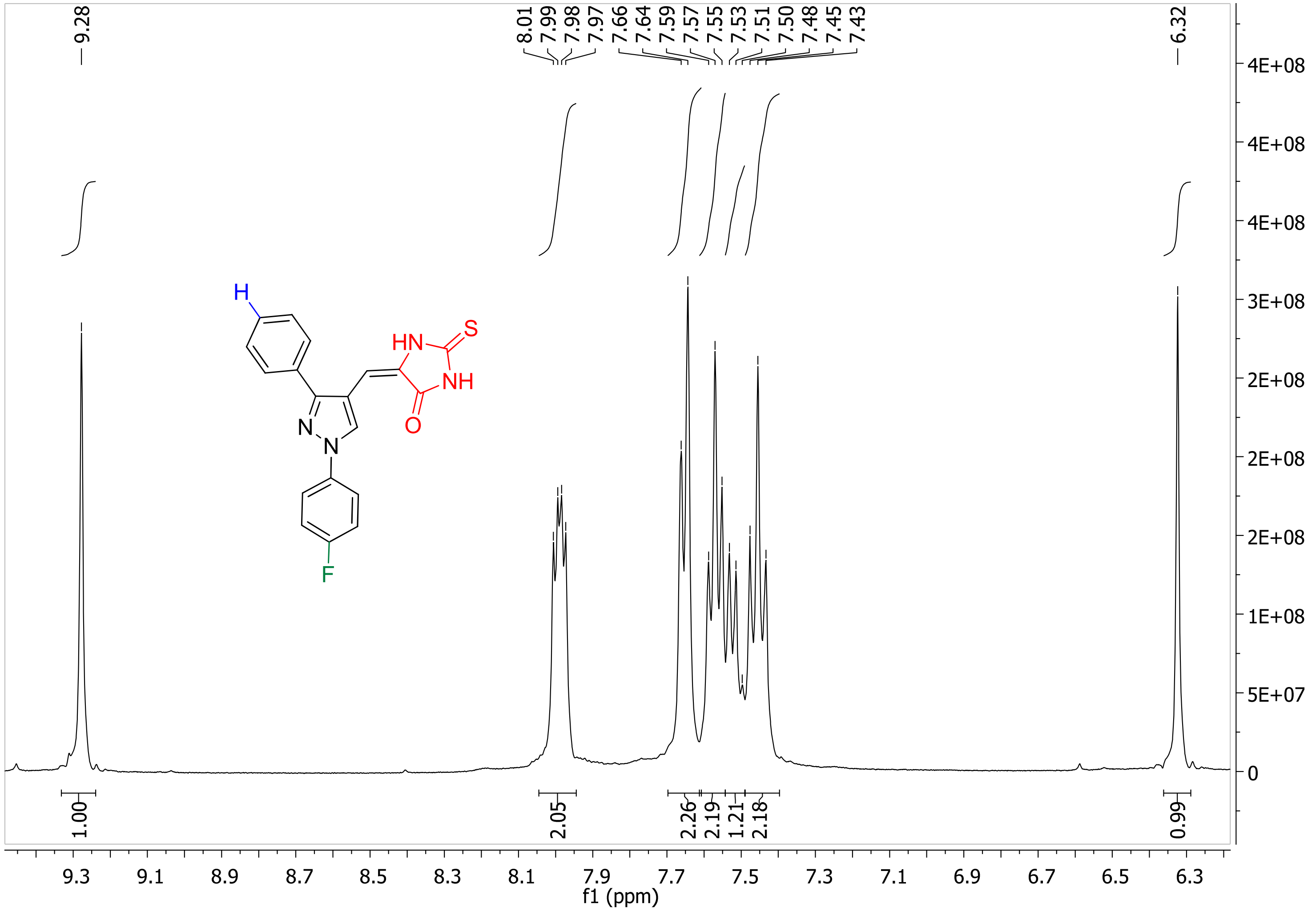

^{1}H NMR (DMSO-*d6*, 400 MHz) spectrum of 5-((1-(4-fluorophenyl)-3-phenyl-1*H*-pyrazol-4-yl)methylene)-2-thioxoimidazolidin-4-one **3l**

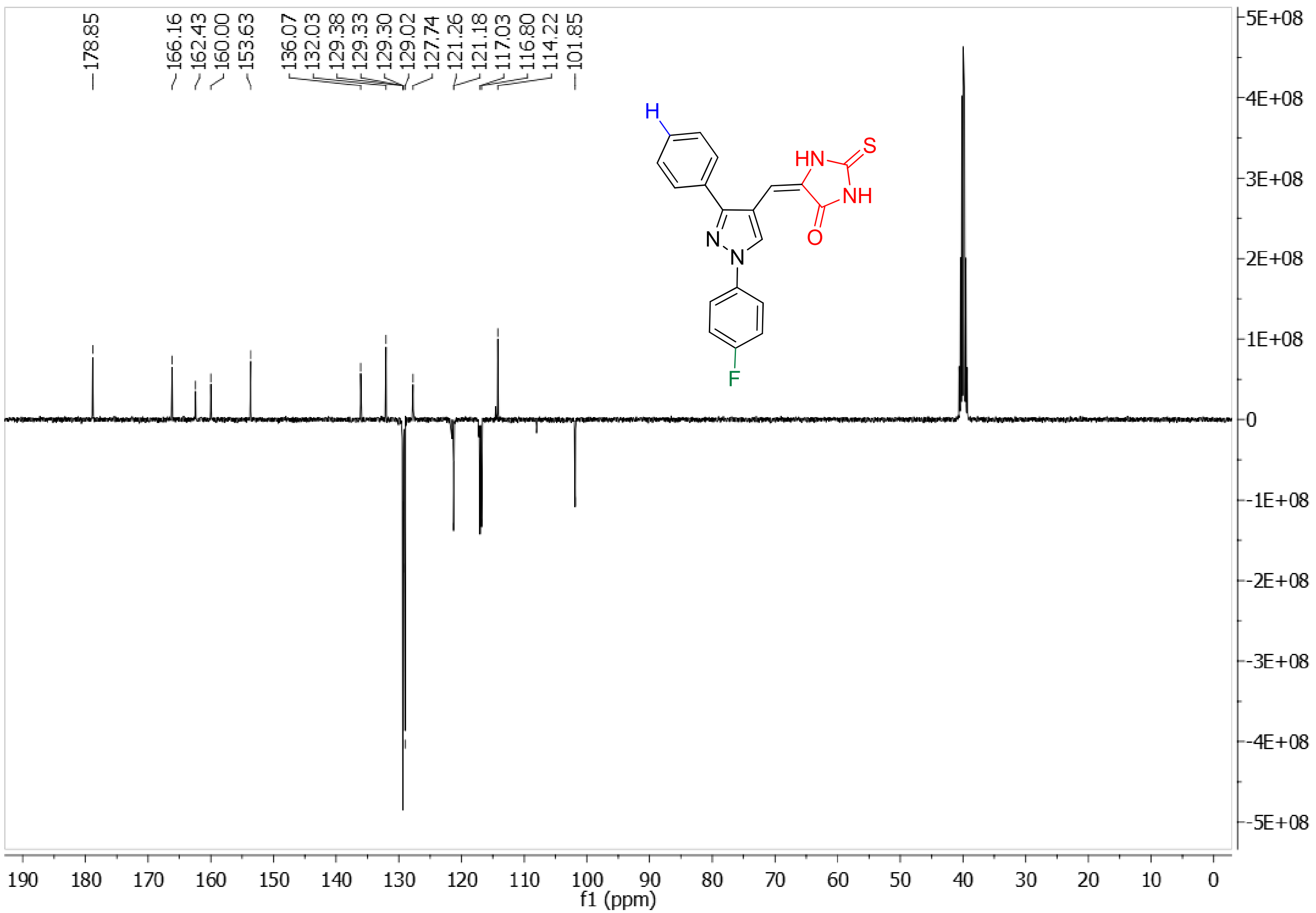

^{13}C APT NMR (DMSO-*d6*, 101 MHz) spectrum of 5-((1-(4-fluorophenyl)-3-phenyl-1*H*-pyrazol-4-yl)methylene)-2-thioxoimidazolidin-4-one **3l**

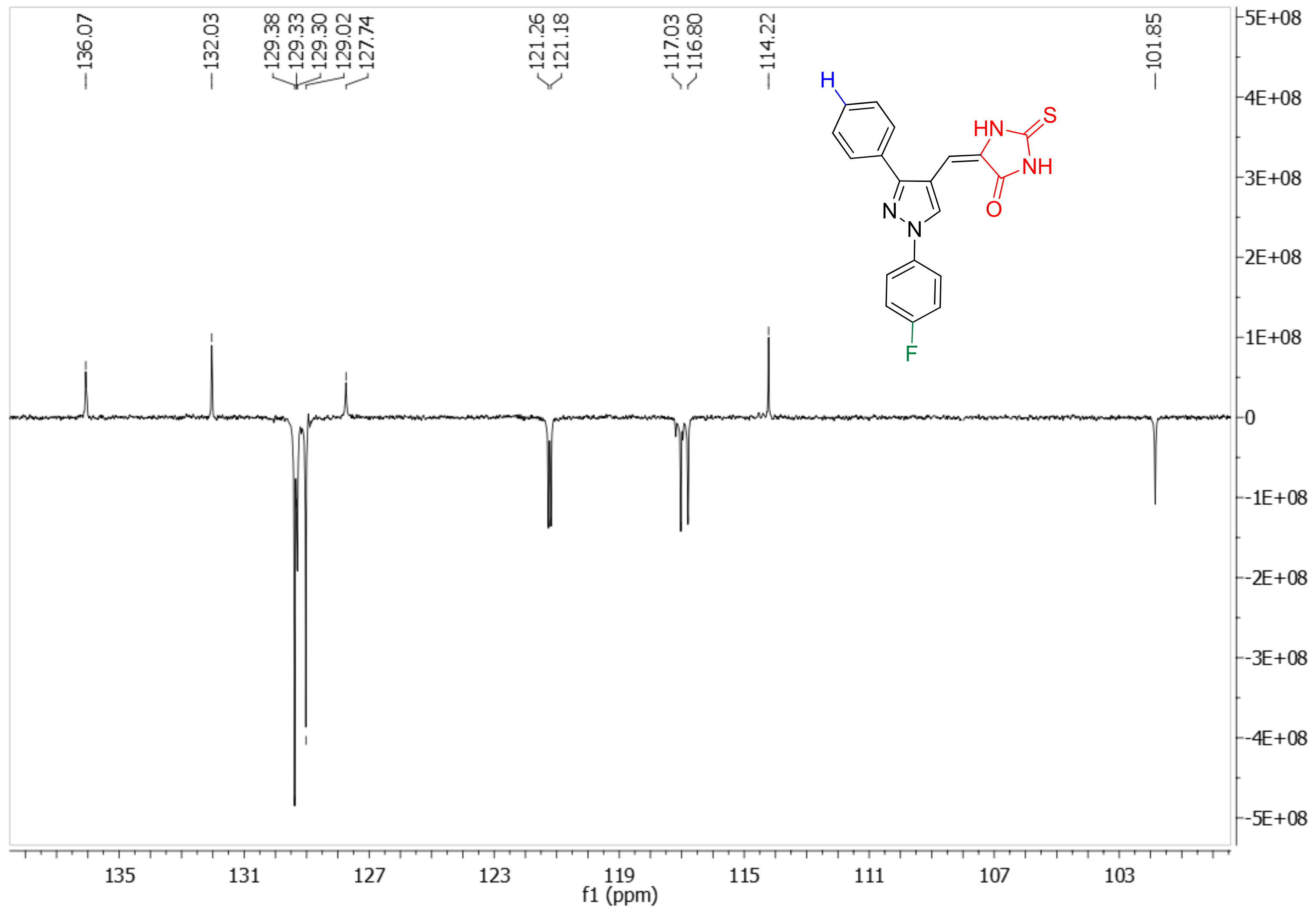

^{13}C APT NMR (DMSO-*d6*, 101 MHz) spectrum of 5-((1-(4-fluorophenyl)-3-phenyl-1*H*-pyrazol-4-yl)methylene)-2-thioxoimidazolidin-4-one **3l**

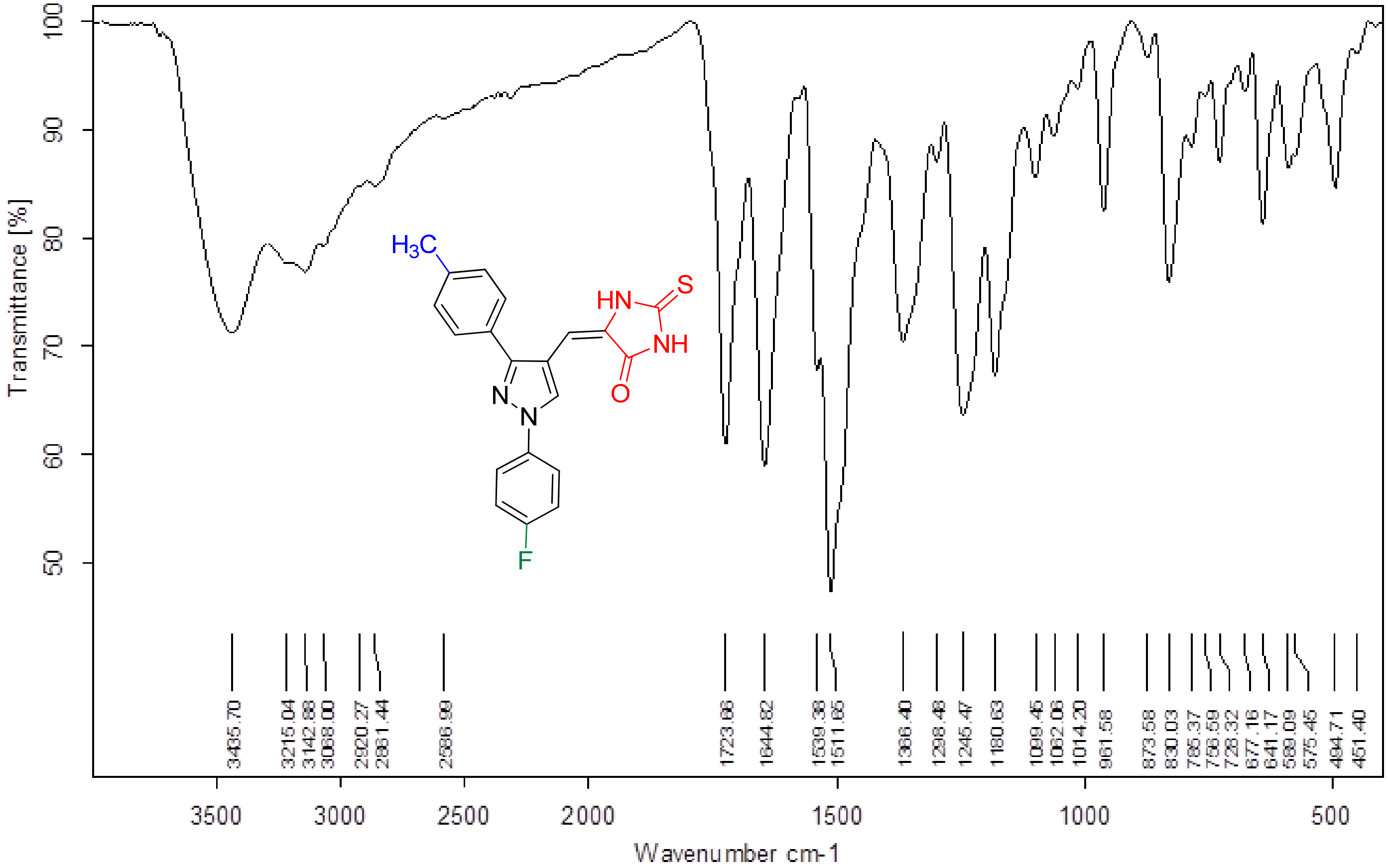

IR (KBr) spectrum of 5-((1-(4-fluorophenyl)-3-(*p*-tolyl)-1*H*-pyrazol-4-yl)methylene)-2-thioxoimidazolidin-4-one **3m**

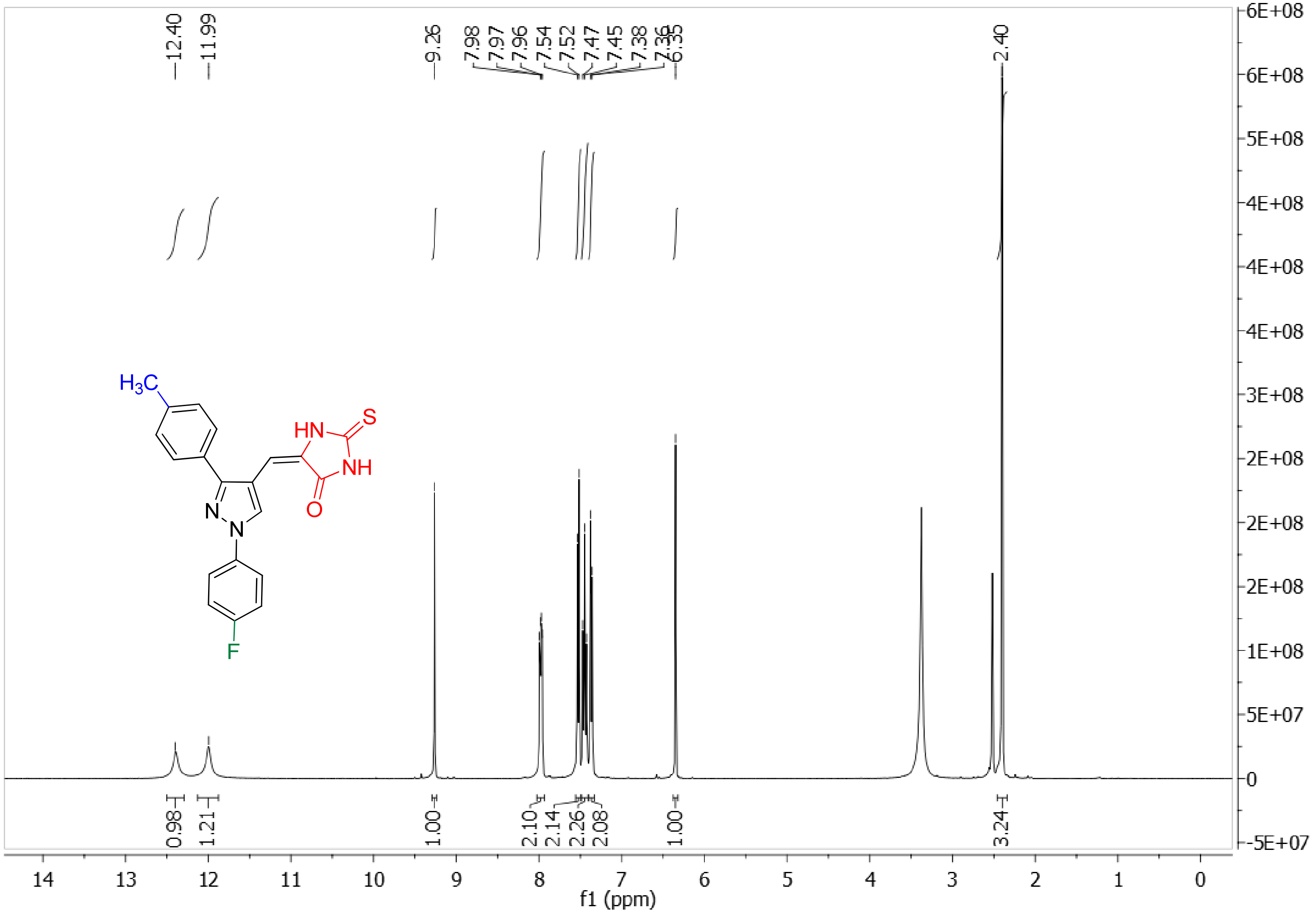

^{1}H NMR (DMSO-*d6*, 400 MHz) spectrum of 5-((1-(4-fluorophenyl)-3-(*p*-tolyl)-1*H*-pyrazol-4-yl)methylene)-2-thioxoimidazolidin-4-one **3m**

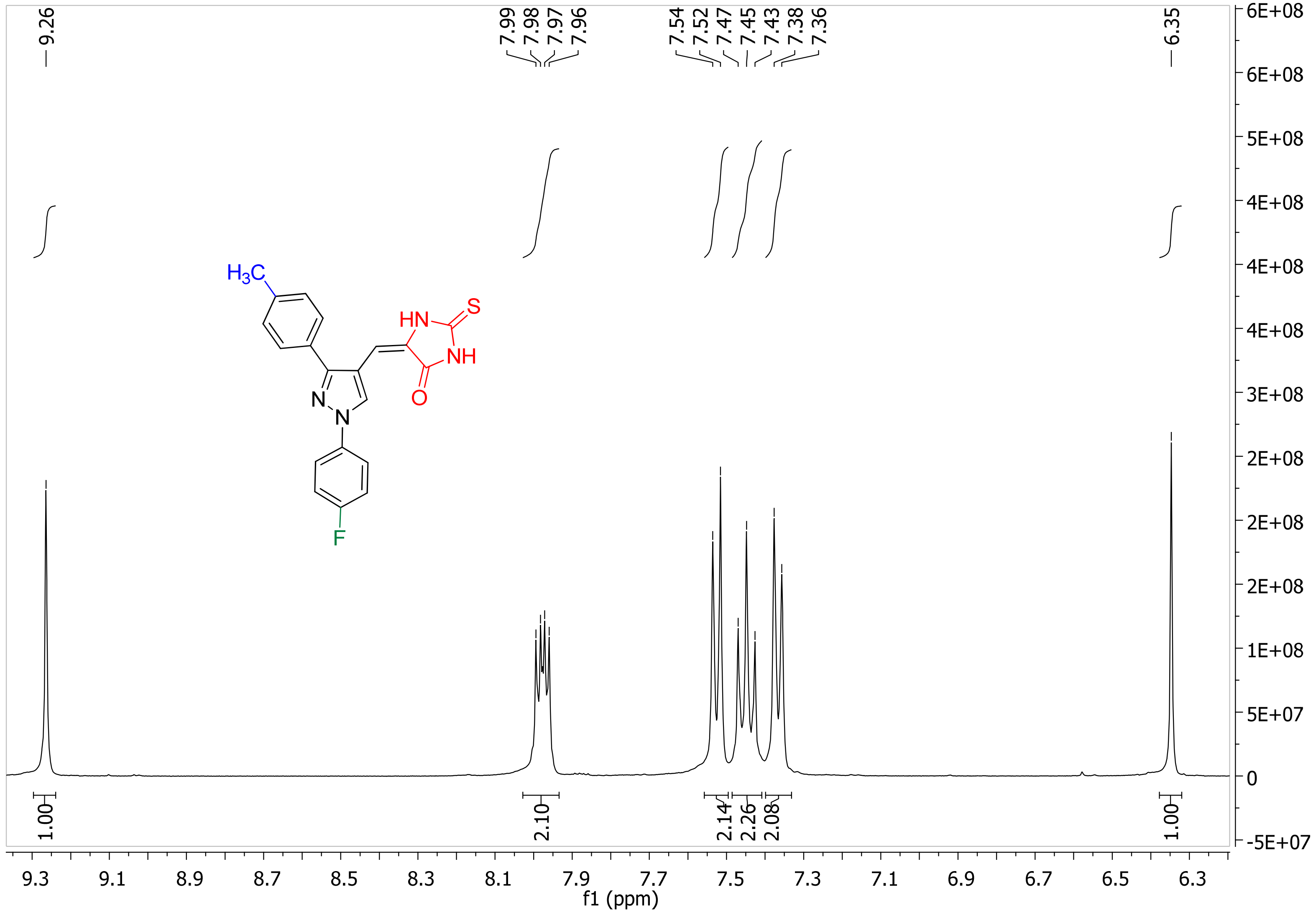

^{1}H NMR (DMSO-*d6*, 400 MHz) spectrum of 5-((1-(4-fluorophenyl)-3-(*p*-tolyl)-1*H*-pyrazol-4-yl)methylene)-2-thioxoimidazolidin-4-one **3m**

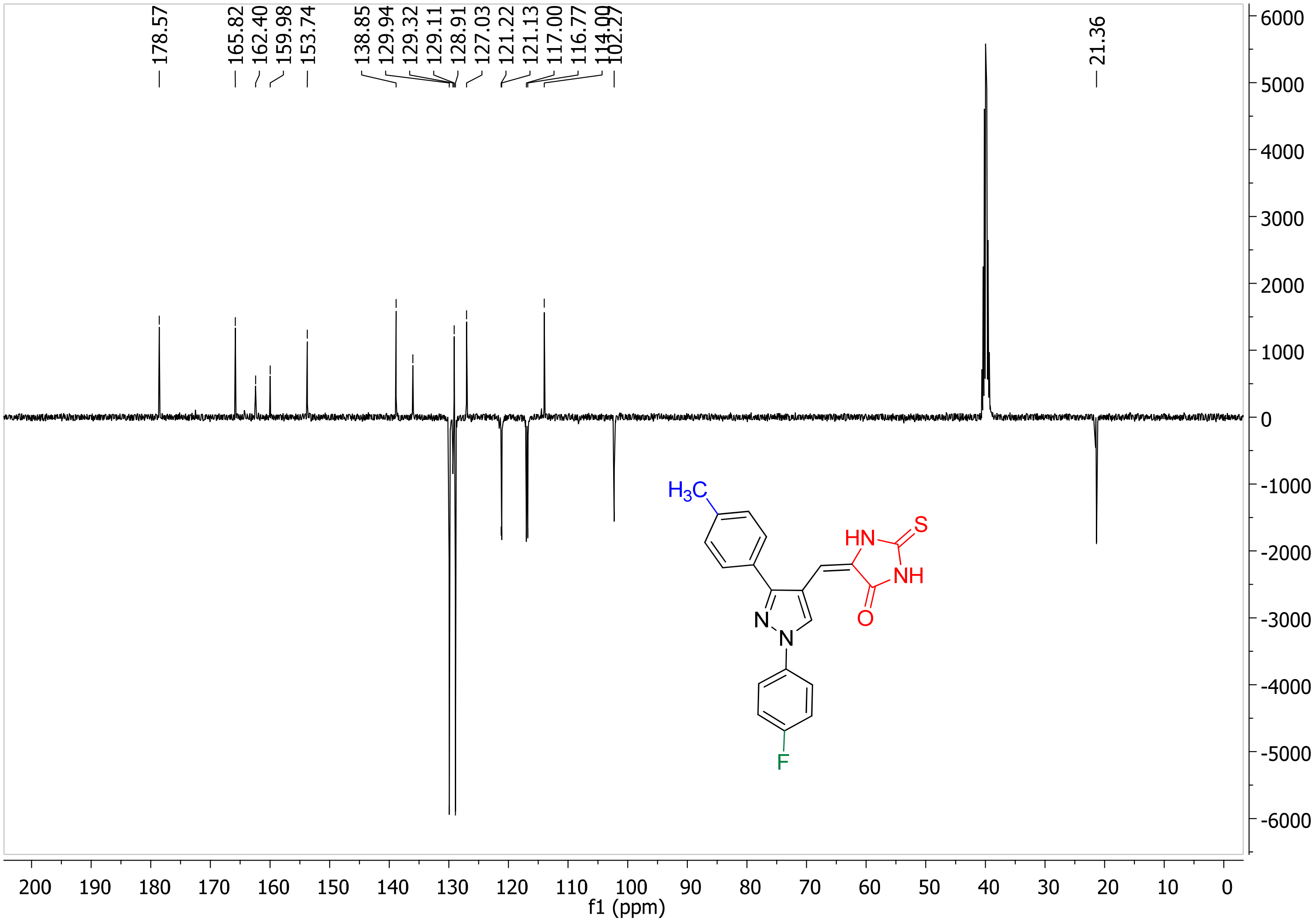

^{13}C APT NMR (DMSO-*d6*, 101 MHz) spectrum of 5-((1-(4-fluorophenyl)-3-(*p*-tolyl)-1*H*-pyrazol-4-yl)methylene)-2-thioxoimidazolidin-4-one **3m**

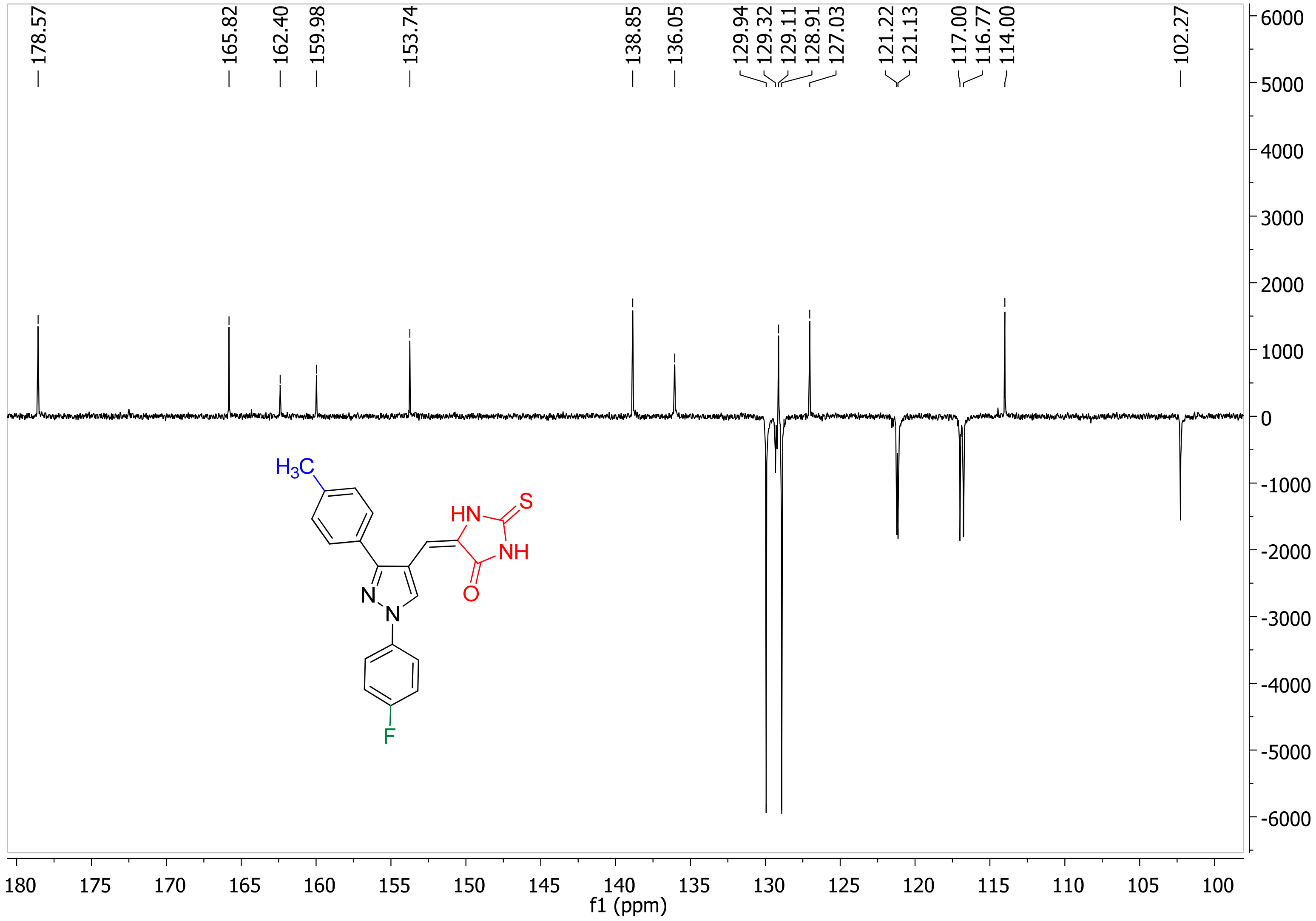

^{13}C APT NMR (DMSO-*d6*, 101 MHz) spectrum of 5-((1-(4-fluorophenyl)-3-(*p*-tolyl)-1*H*-pyrazol-4-yl)methylene)-2-thioxoimidazolidin-4-one

3m

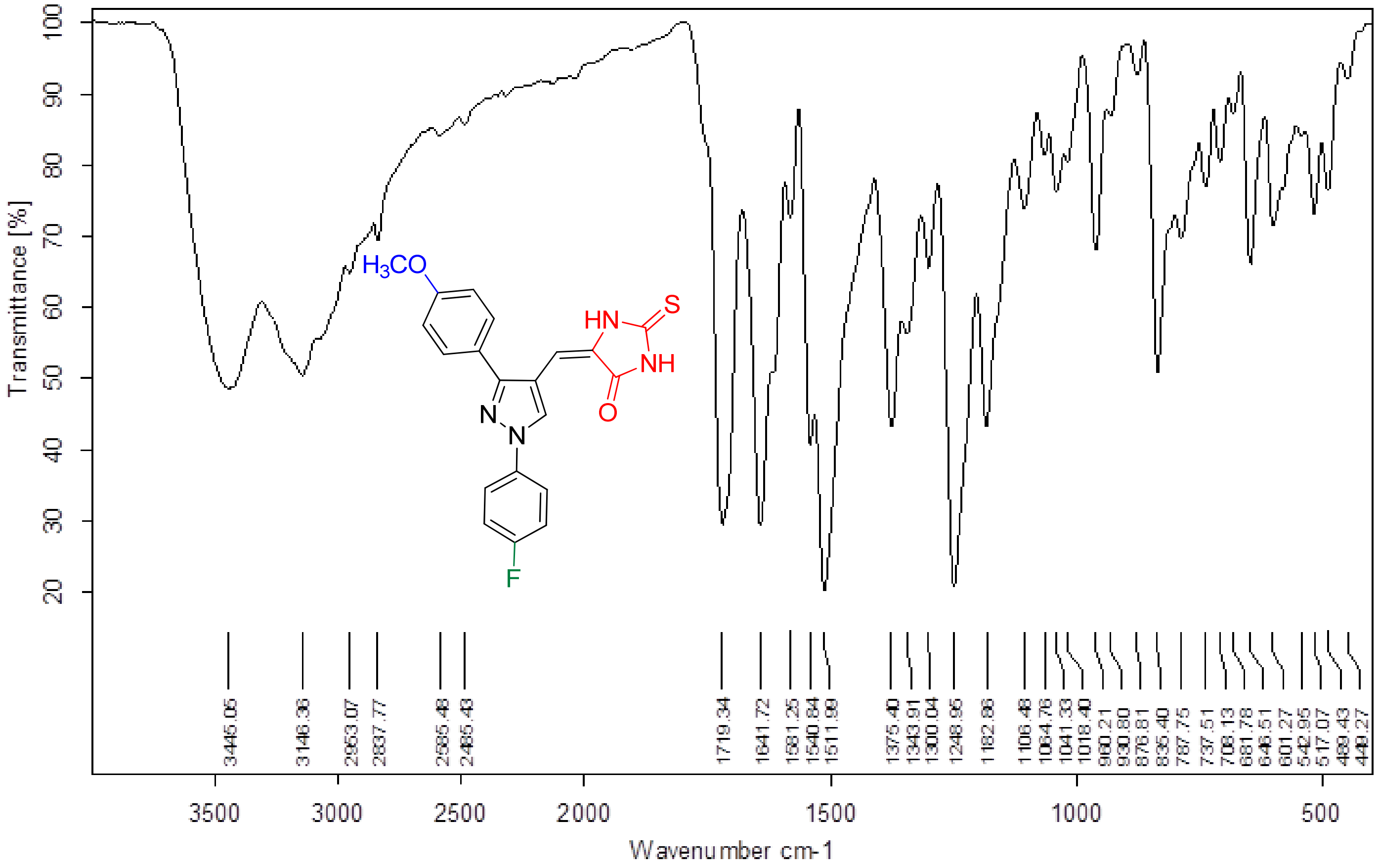

IR (KBr) spectrum of 5-((1-(4-fluorophenyl)-3-(4-methoxyphenyl)-1*H*-pyrazol-4-yl)methylene)-2-thioxoimidazolidin-4-one **3n**

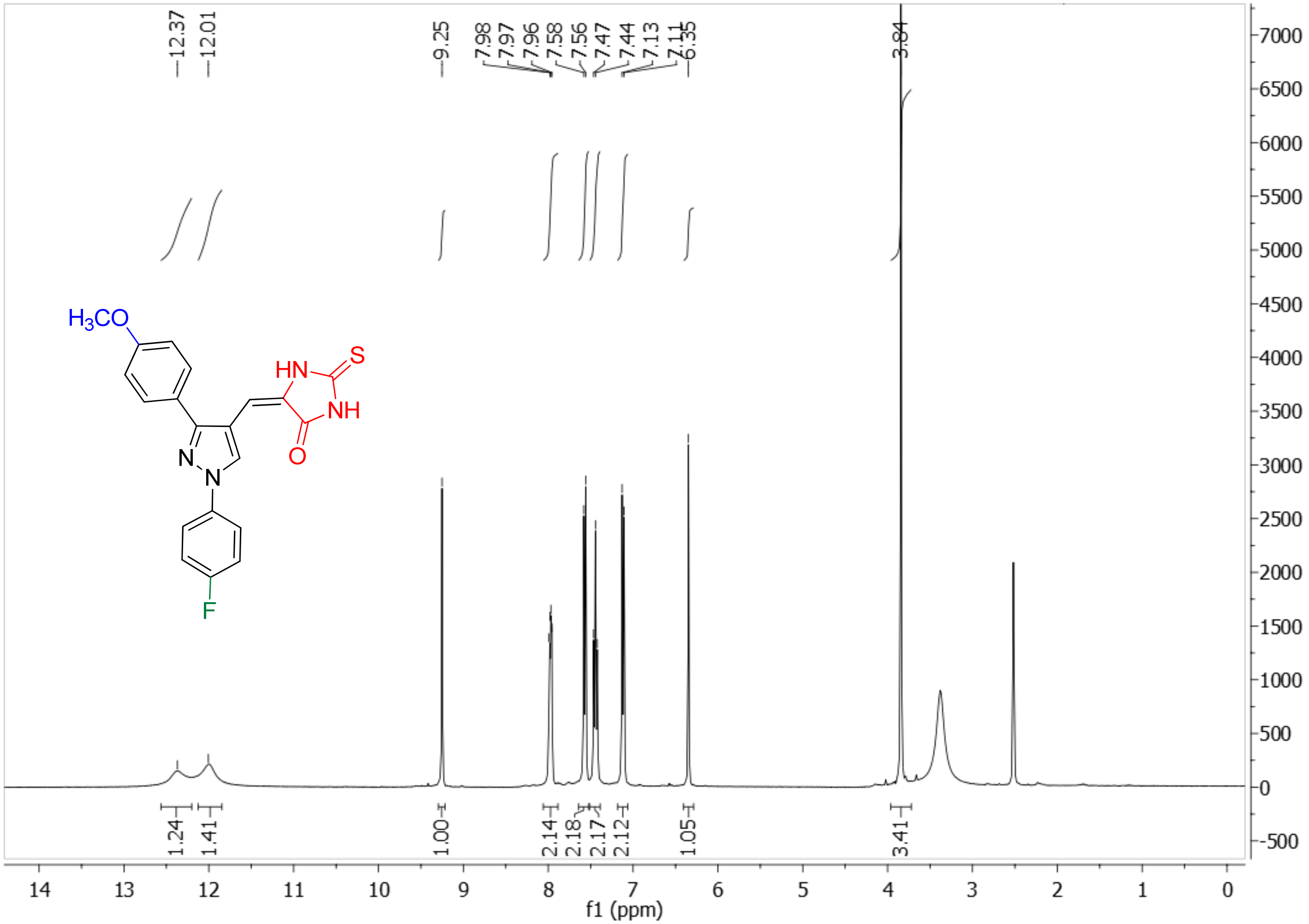

^{1}H NMR (DMSO-*d6*, 400 MHz) spectrum of 5-((1-(4-fluorophenyl)-3-(4-methoxyphenyl)-1*H*-pyrazol-4-yl)methylene)-2-thioxoimidazolidin-4-one **3n**

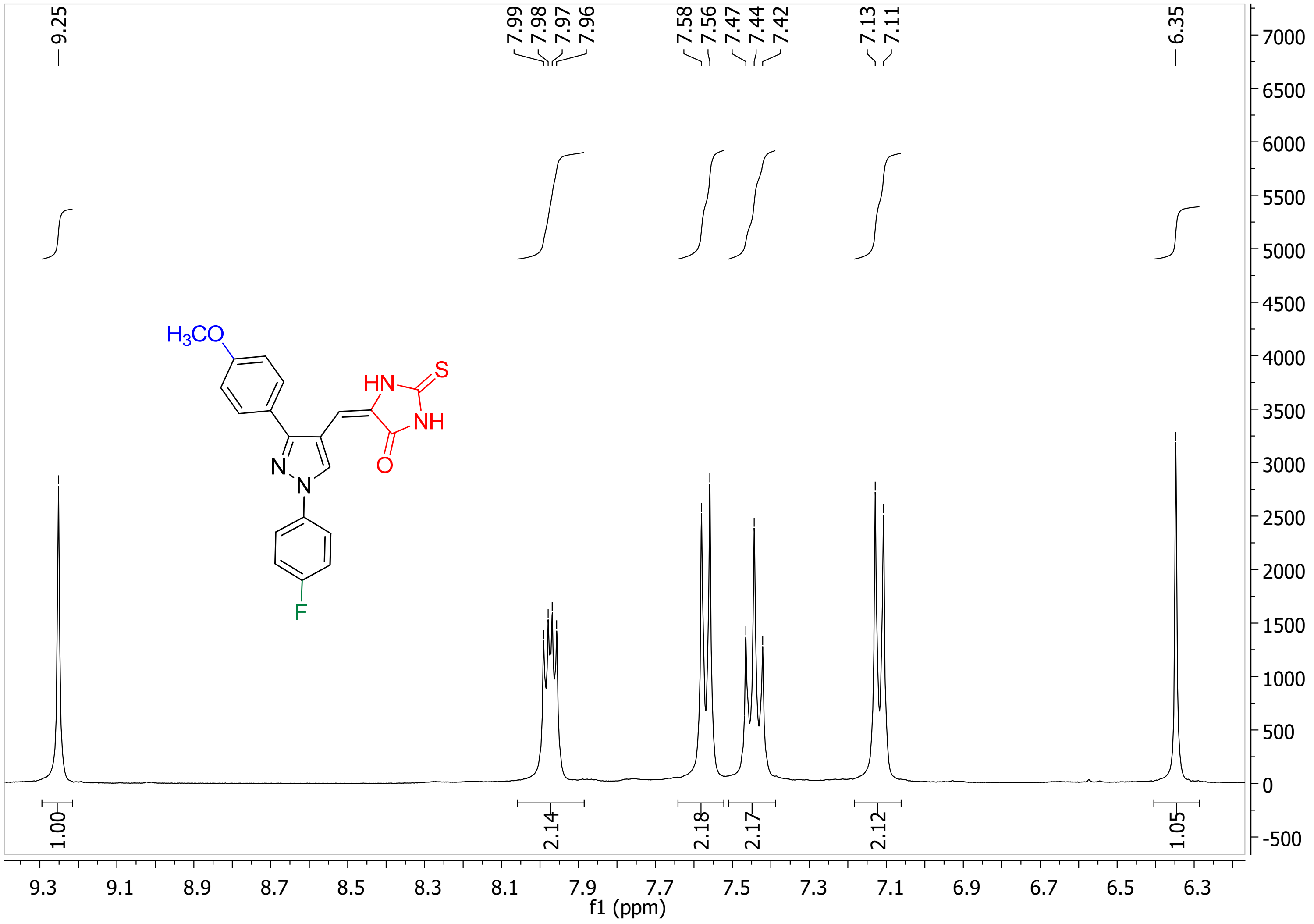

^{1}H NMR (DMSO-*d6*, 400 MHz) spectrum of 5-((1-(4-fluorophenyl)-3-(4-methoxyphenyl)-1*H*-pyrazol-4-yl)methylene)-2-thioxoimidazolidin-4-one **3n**

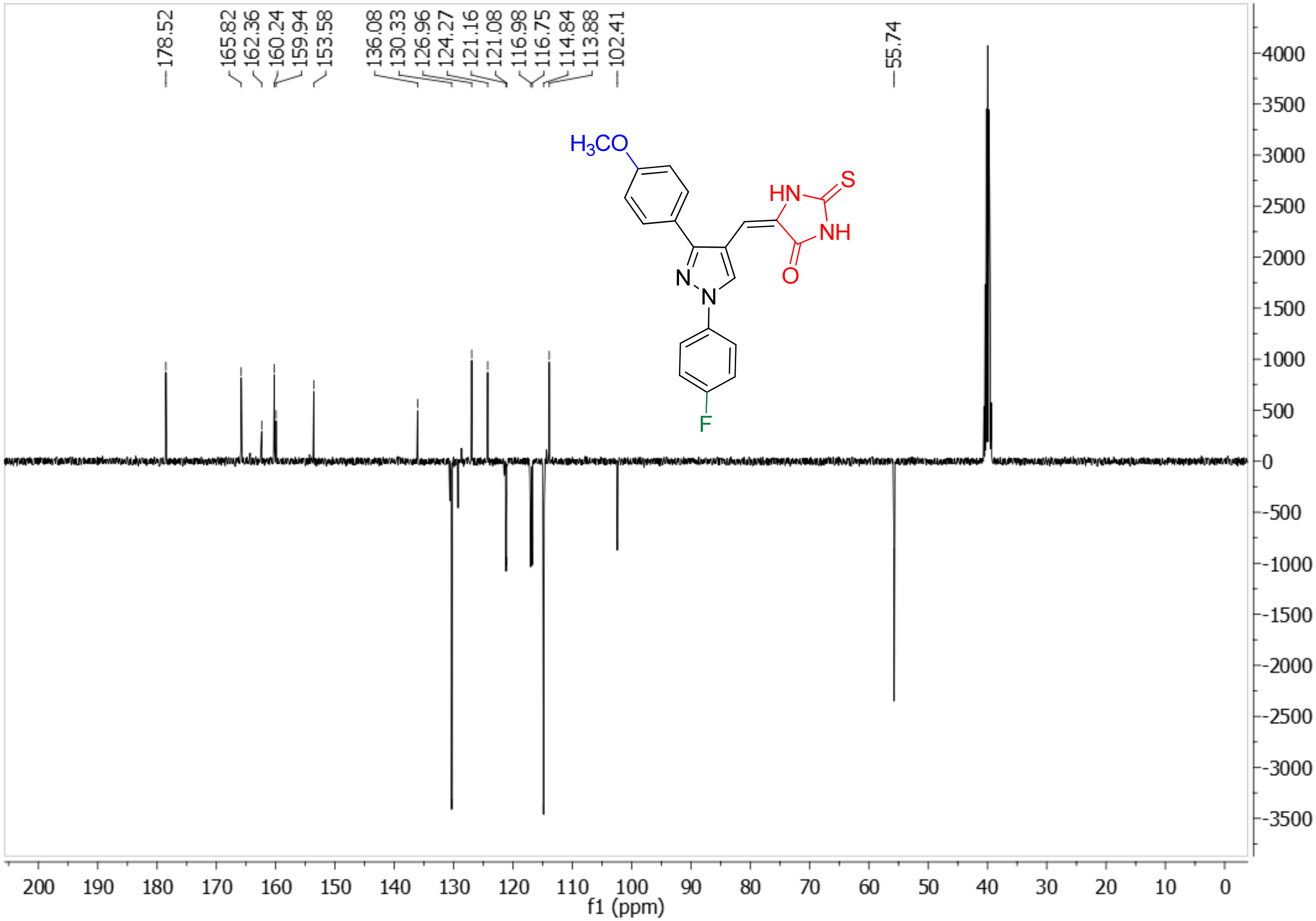

^{13}C APT NMR (DMSO-*d6*, 101 MHz) spectrum of 5-((1-(4-fluorophenyl)-3-(4-methoxyphenyl)-1*H*-pyrazol-4-yl)methylene)-2-thioxoimidazolidin-4-one **3n**

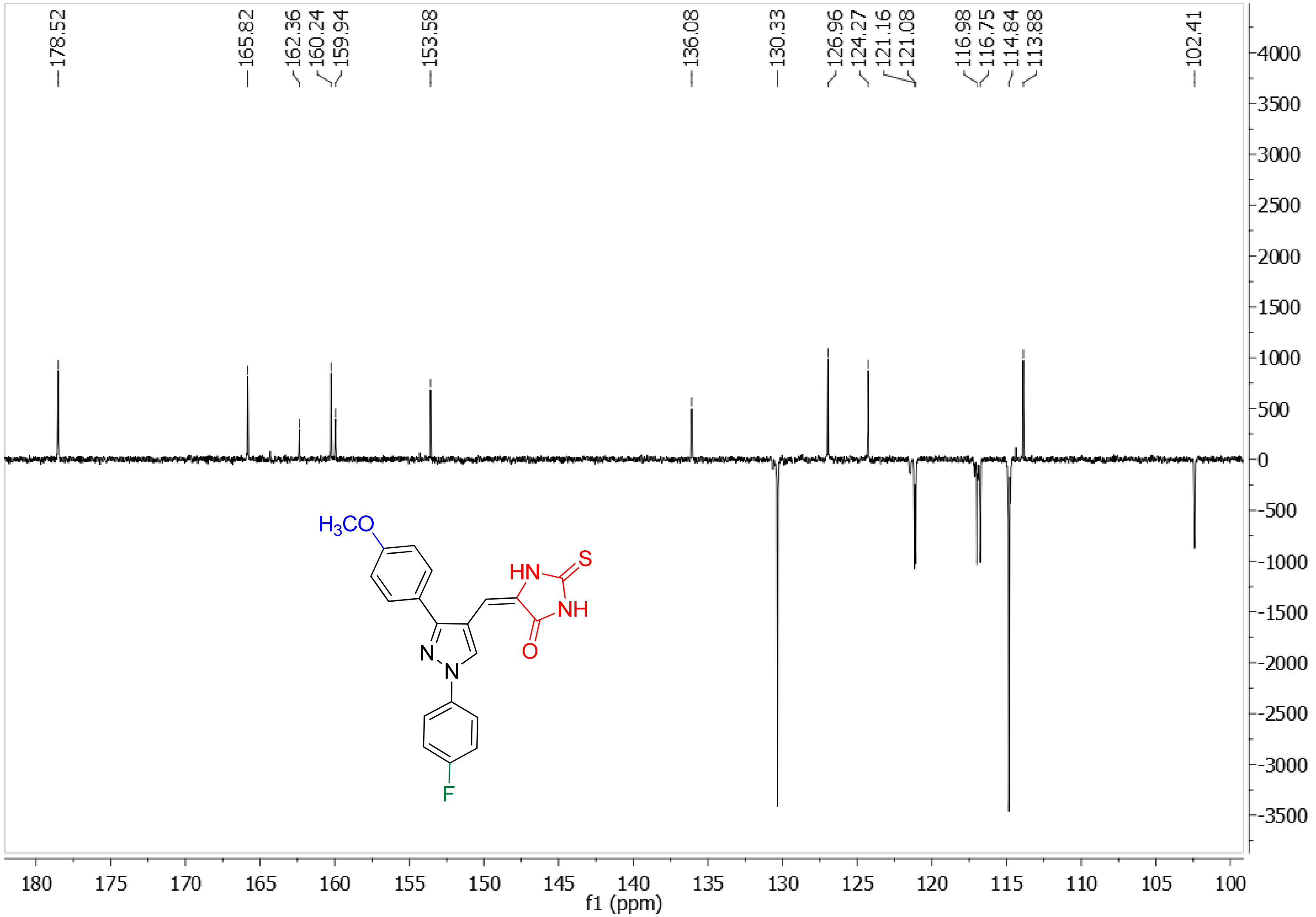

^{13}C APT NMR (DMSO-*d6*, 101 MHz) spectrum of 5-((1-(4-fluorophenyl)-3-(4-methoxyphenyl)-1*H*-pyrazol-4-yl)methylene)-2-thioxoimidazolidin-4-one **3n**

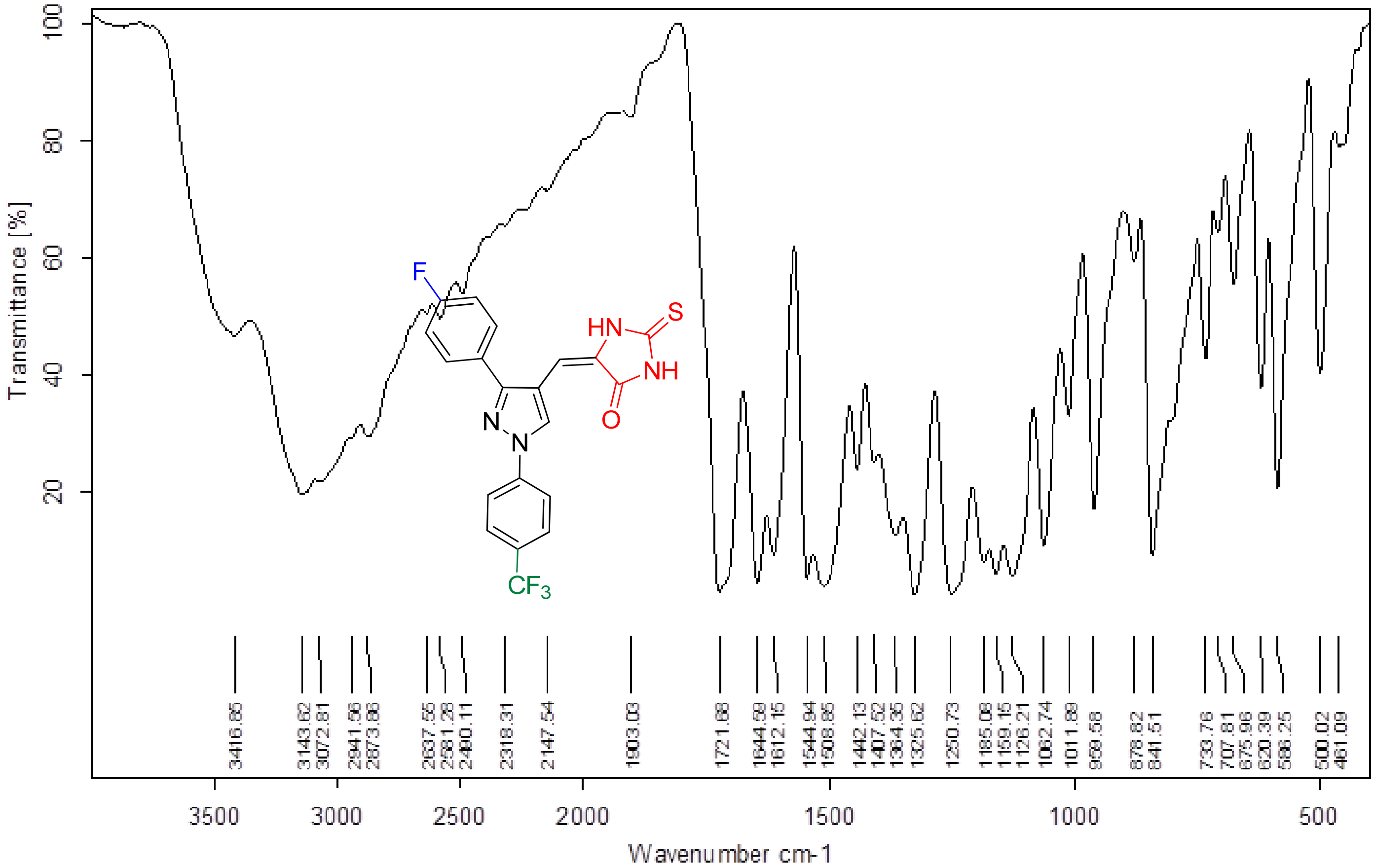

IR (KBr) spectrum of 5-((3-(4-fluorophenyl)-1-(4-(trifluoromethyl)phenyl)-1*H*-pyrazol-4-yl)methylene)-2-thioxoimidazolidin-4-one **3o**

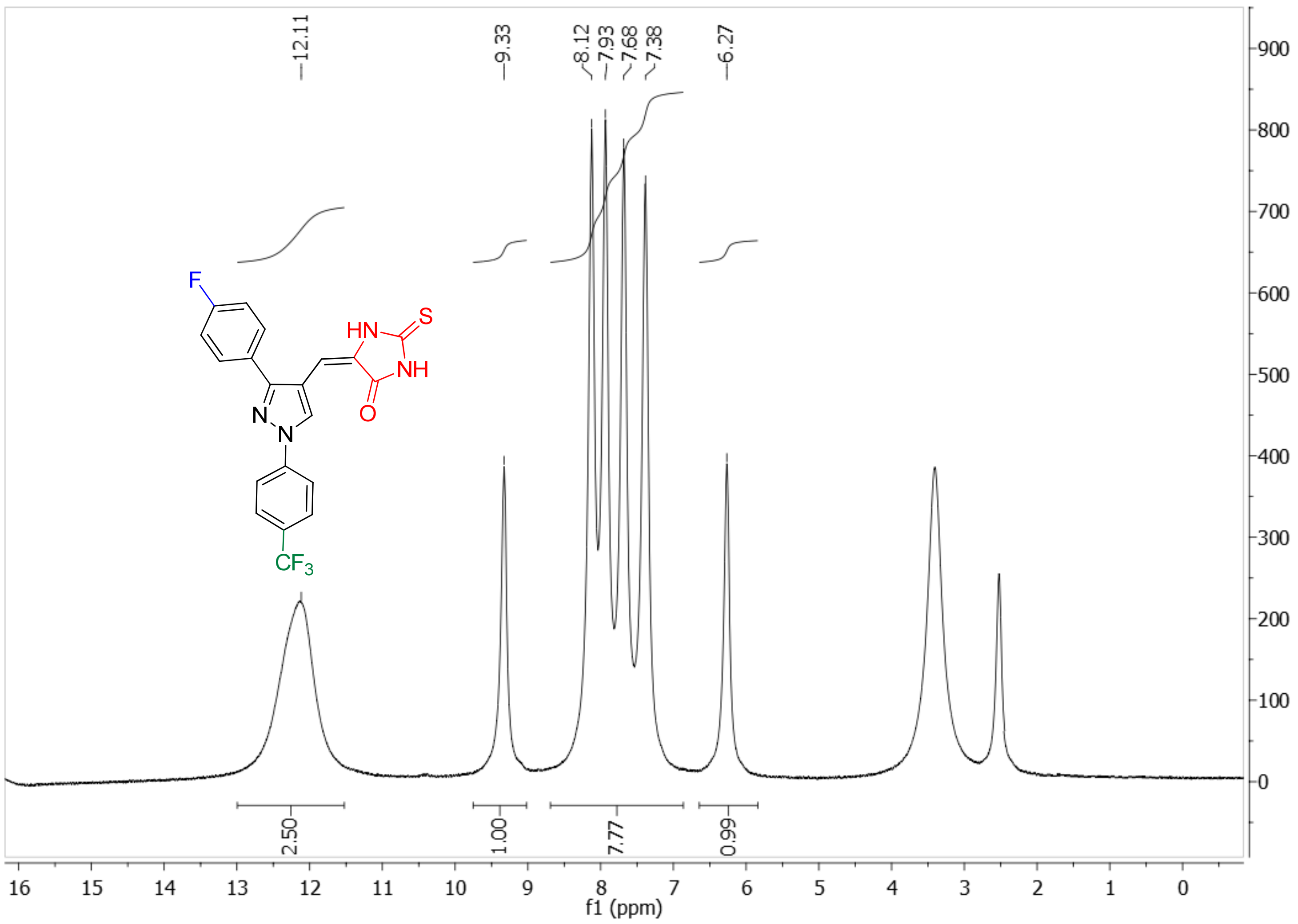

^{1}H NMR (DMSO-*d6*, 400 MHz) spectrum of 5-((3-(4-fluorophenyl)-1-(4-(trifluoromethyl)phenyl)-1*H*-pyrazol-4-yl)methylene)-2-thioxoimidazolidin-4-one **3o**

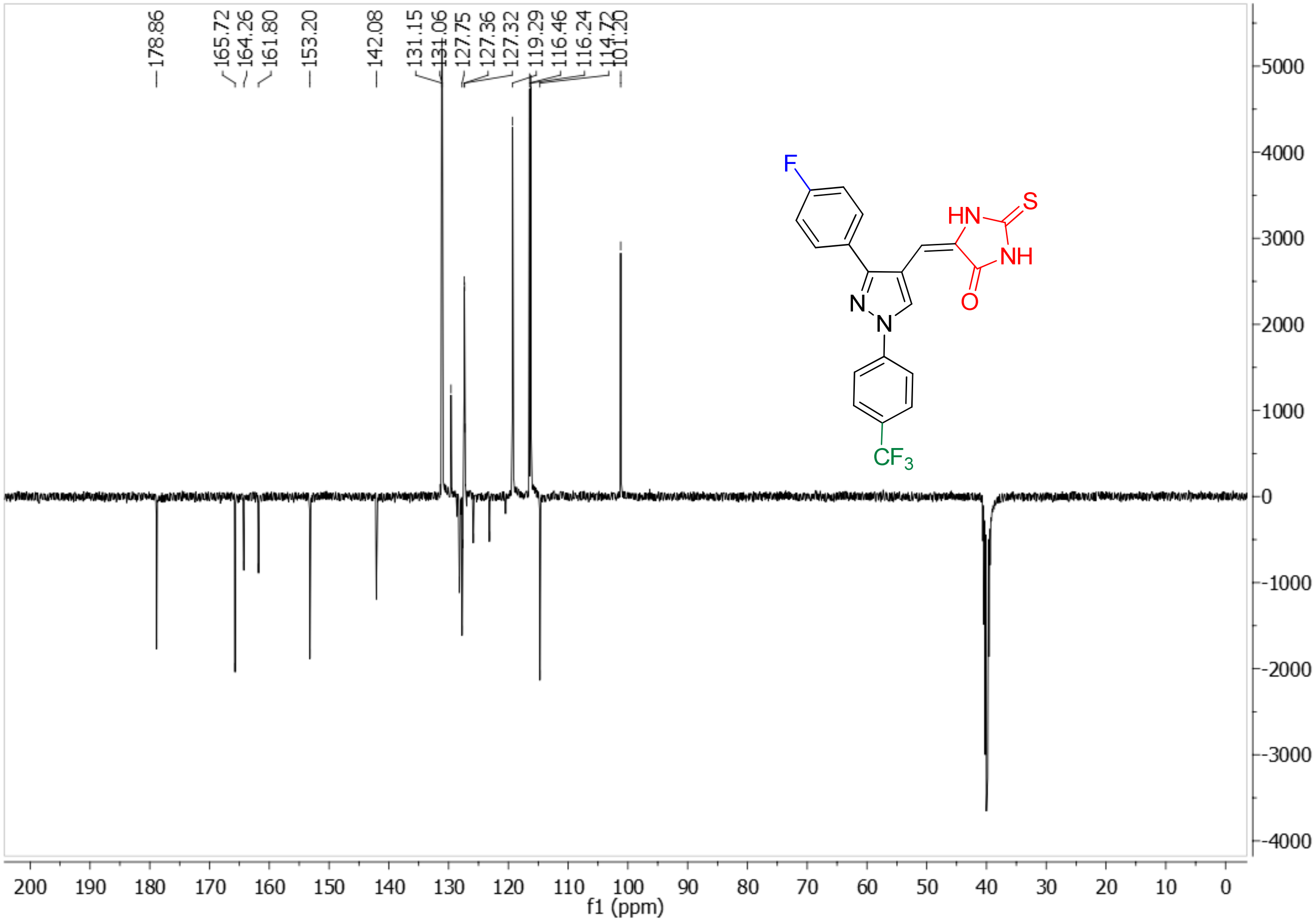

^{13}C APT NMR (DMSO-*d6*, 101 MHz) spectrum of 5-((3-(4-fluorophenyl)-1-(4-(trifluoromethyl)phenyl)-1*H*-pyrazol-4-yl)methylene)-2-thioxoimidazolidin-4-one **3o**

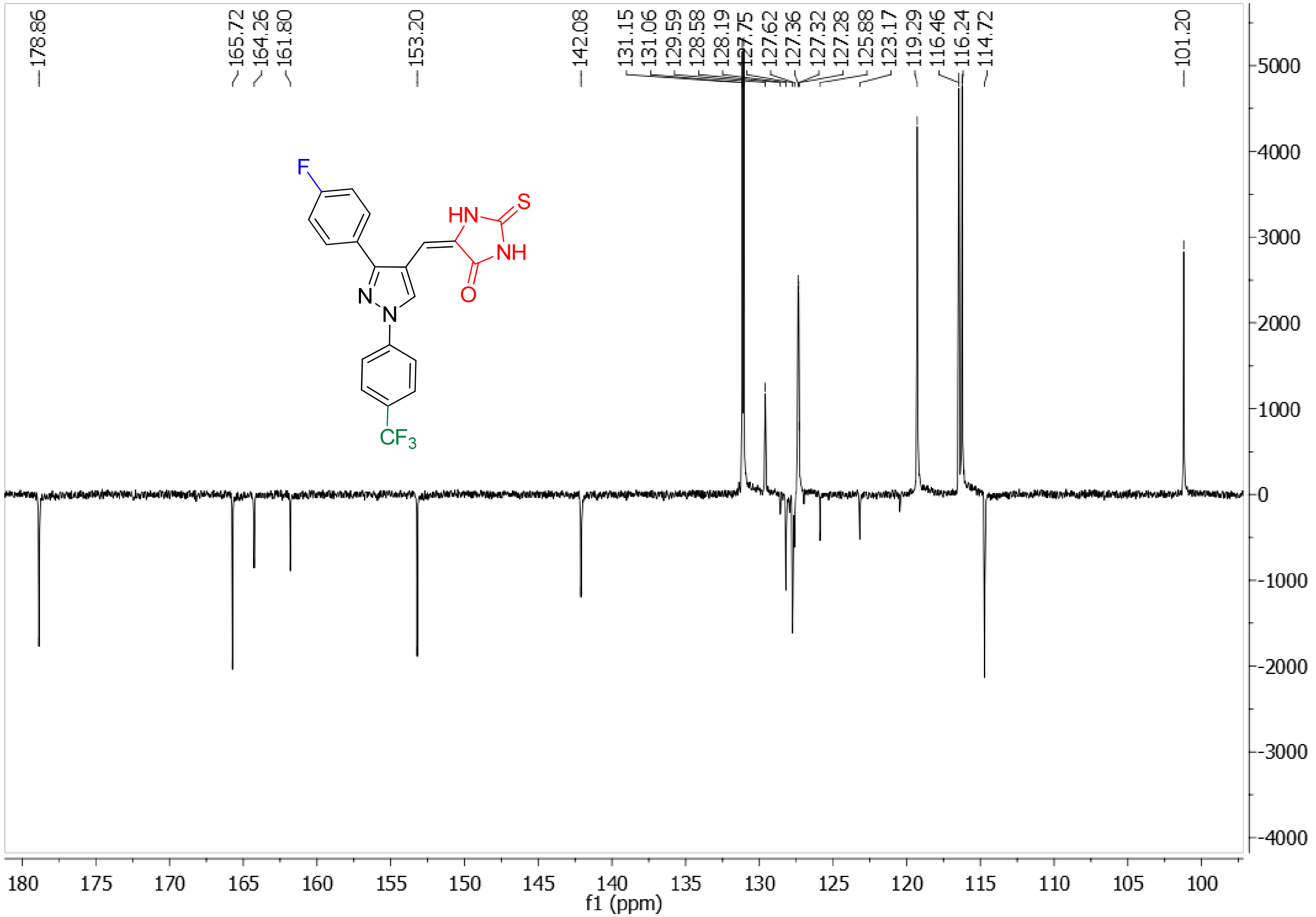

^{13}C APT NMR (DMSO-*d6*, 101 MHz) spectrum of 5-((3-(4-fluorophenyl)-1-(4-(trifluoromethyl)phenyl)-1*H*-pyrazol-4-yl)methylene)-2-thioxoimidazolidin-4-one **3o**

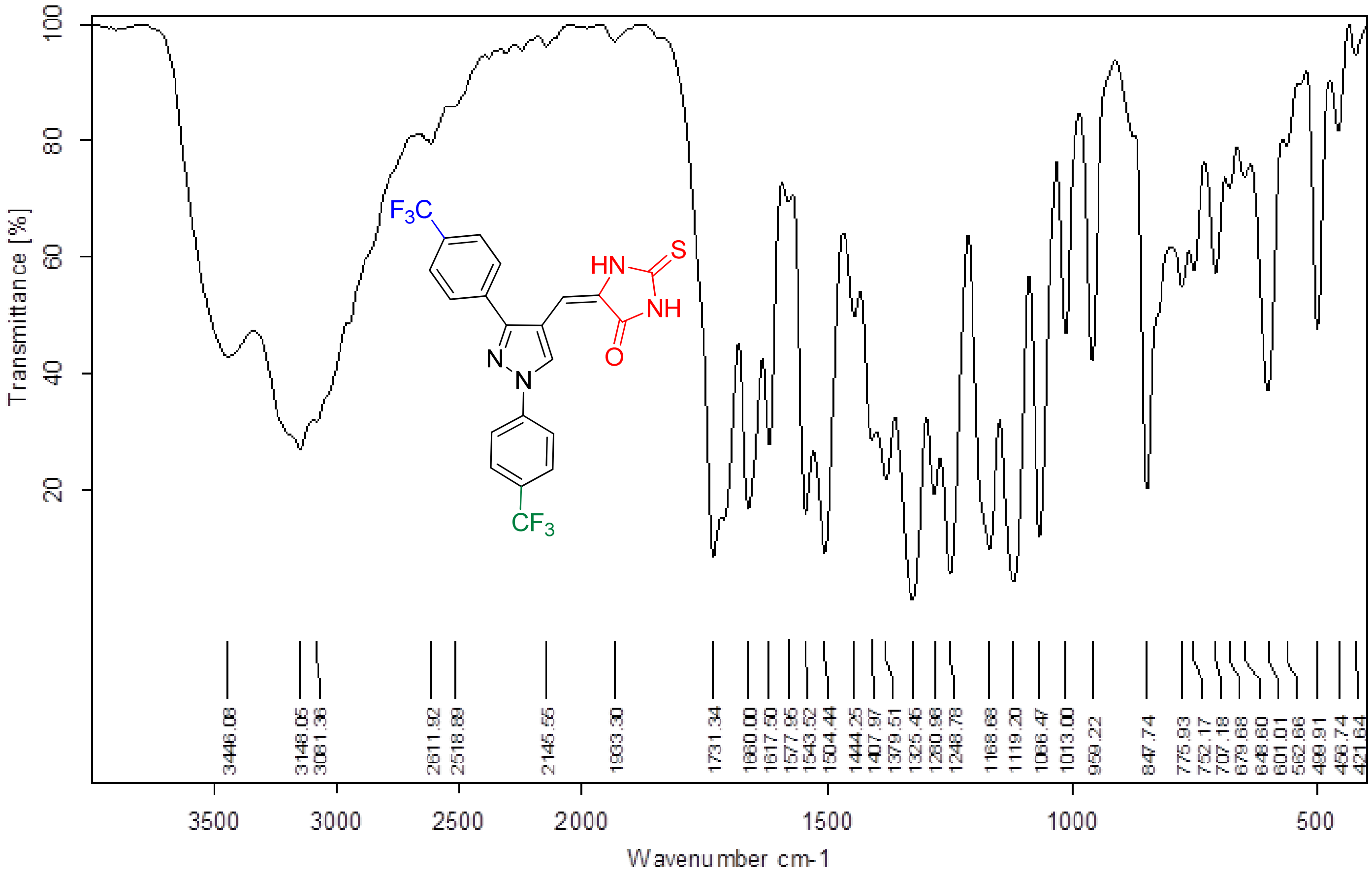

IR (KBr) spectrum of 5-((1,3-bis(4-(trifluoromethyl)phenyl)-1*H*-pyrazol-4-yl)methylene)-2-thioxoimidazolidin-4-one **3p**

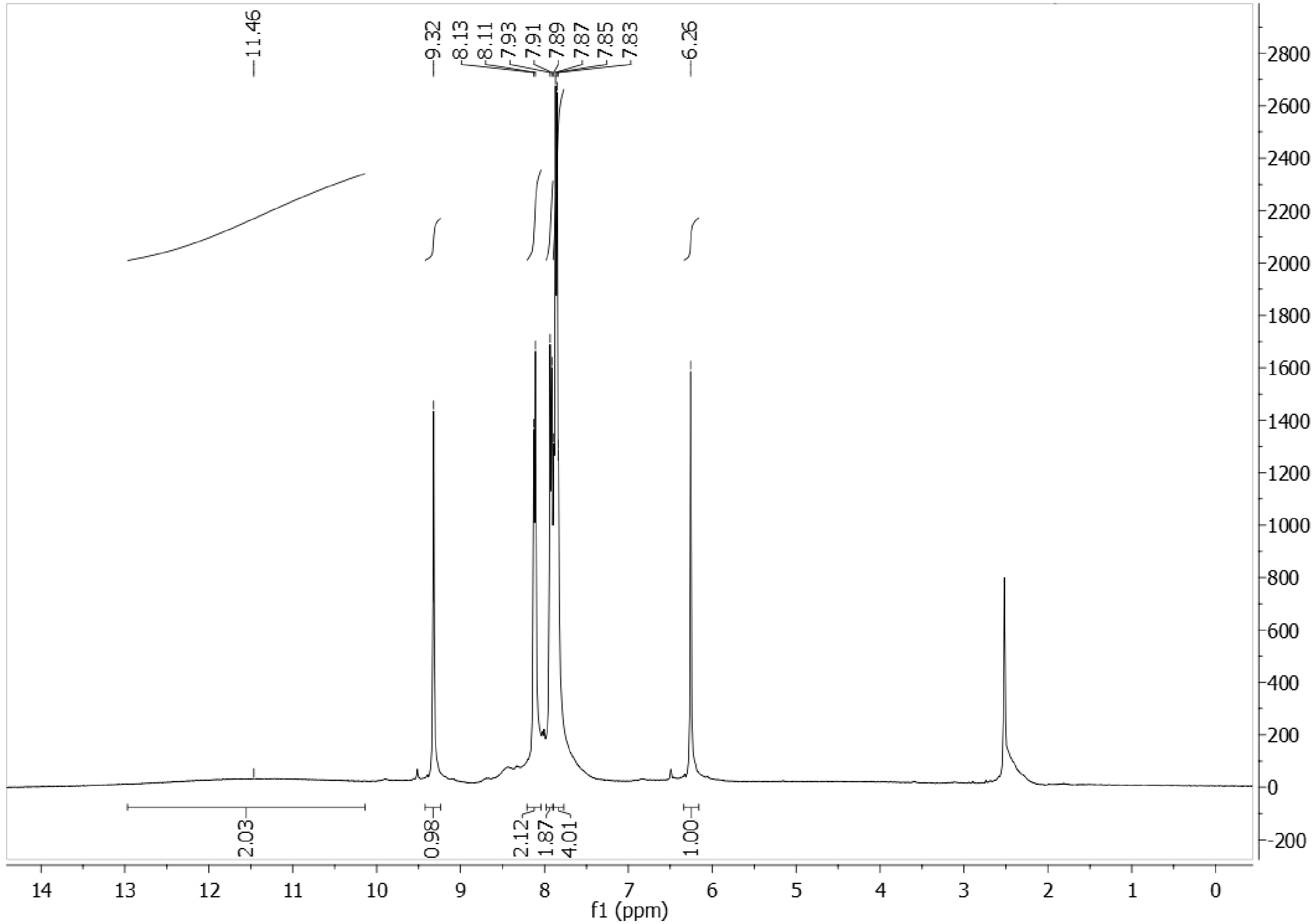

^{1}H NMR (DMSO-*d6*, 400 MHz) spectrum of 5-((1,3-bis(4-(trifluoromethyl)phenyl)-1*H*-pyrazol-4-yl)methylene)-2-thioxoimidazolidin-4-one **3p**

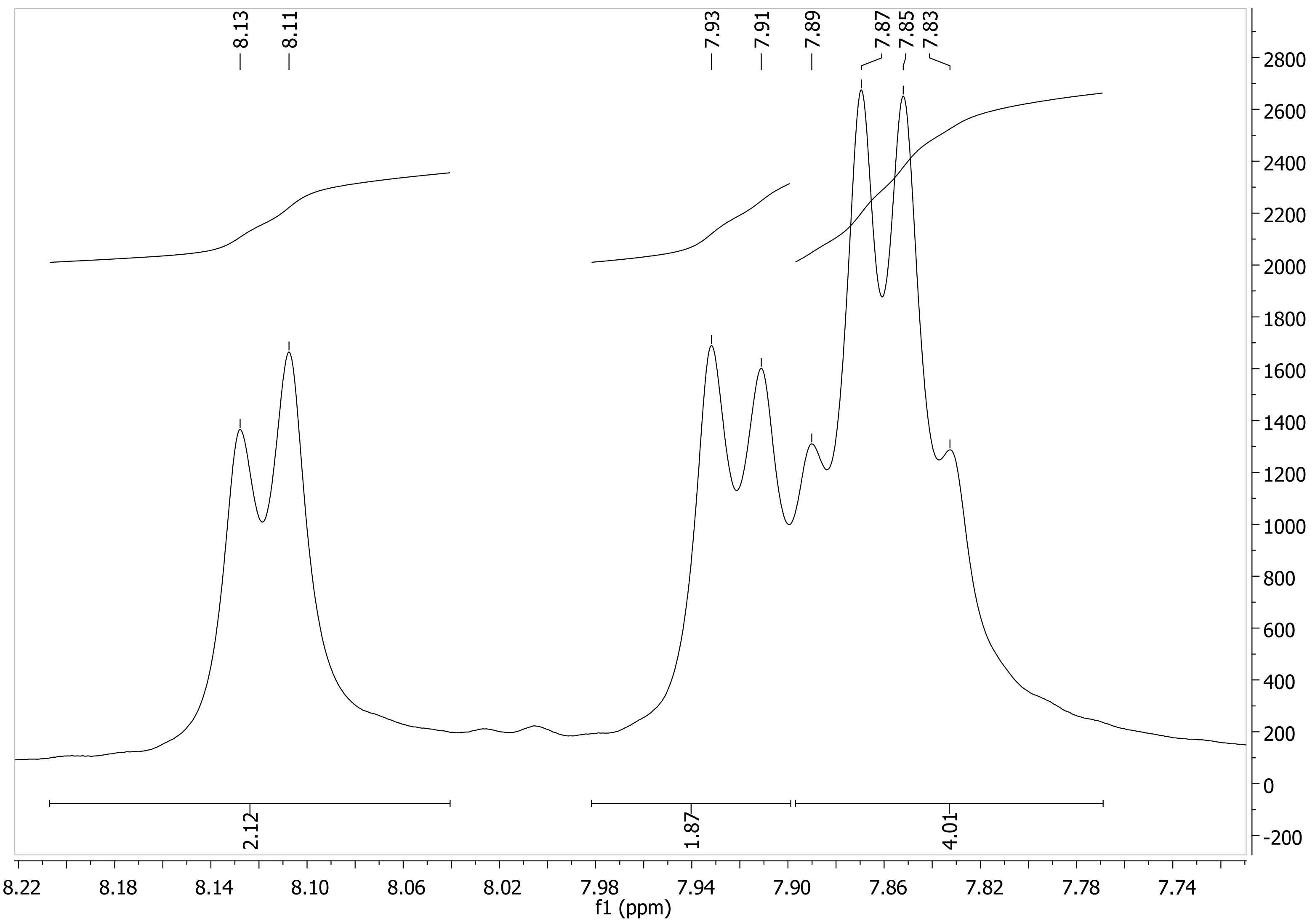

^{1}H NMR (DMSO-*d6*, 400 MHz) spectrum of 5-((1,3-bis(4-(trifluoromethyl)phenyl)-1*H*-pyrazol-4-yl)methylene)-2-thioxoimidazolidin-4-one **3p**

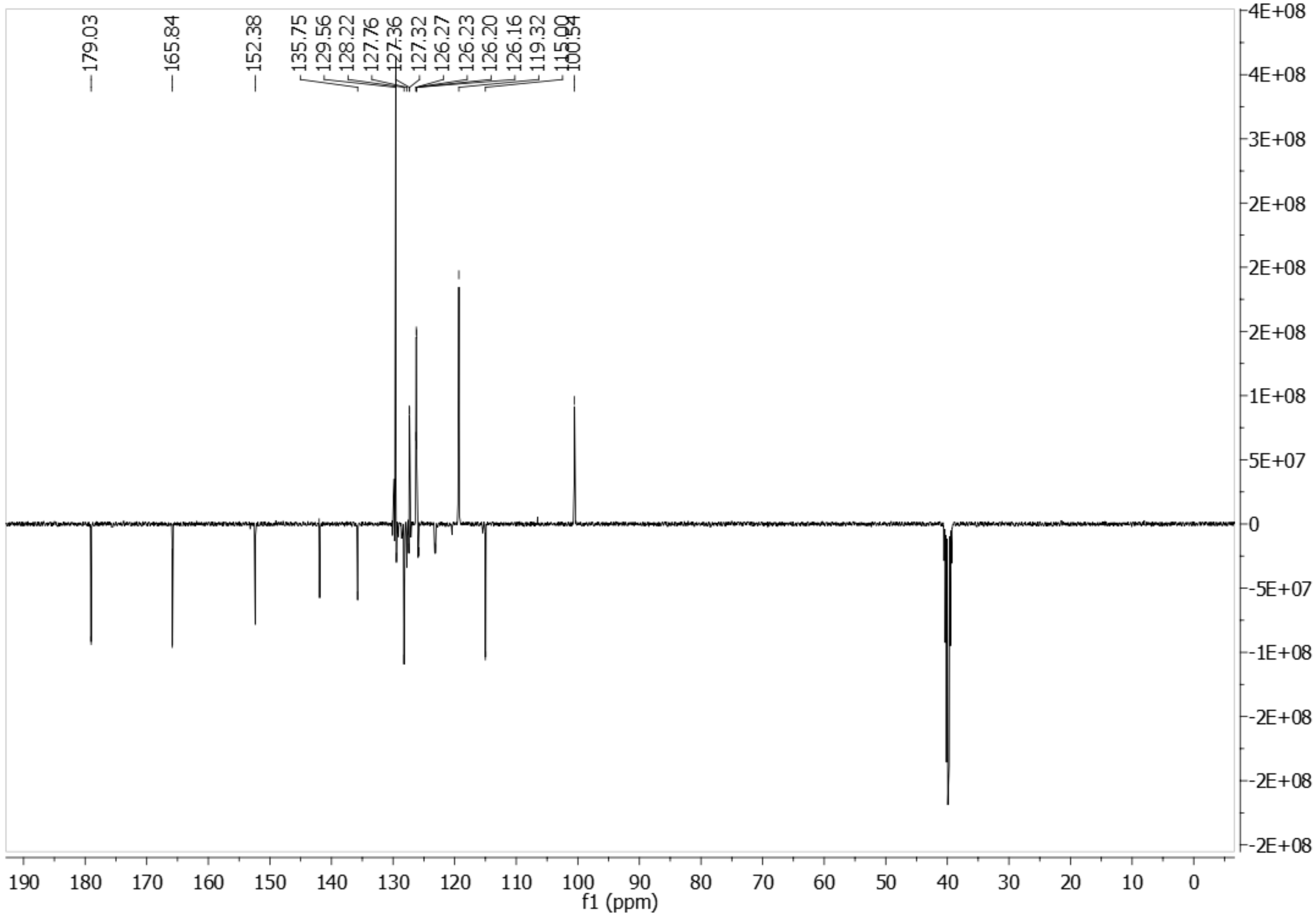

^{13}C APT NMR (DMSO-*d6*, 101 MHz) spectrum of 5-((1,3-bis(4-(trifluoromethyl)phenyl)-1*H*-pyrazol-4-yl)methylene)-2-thioxoimidazolidin-4-one **3p**

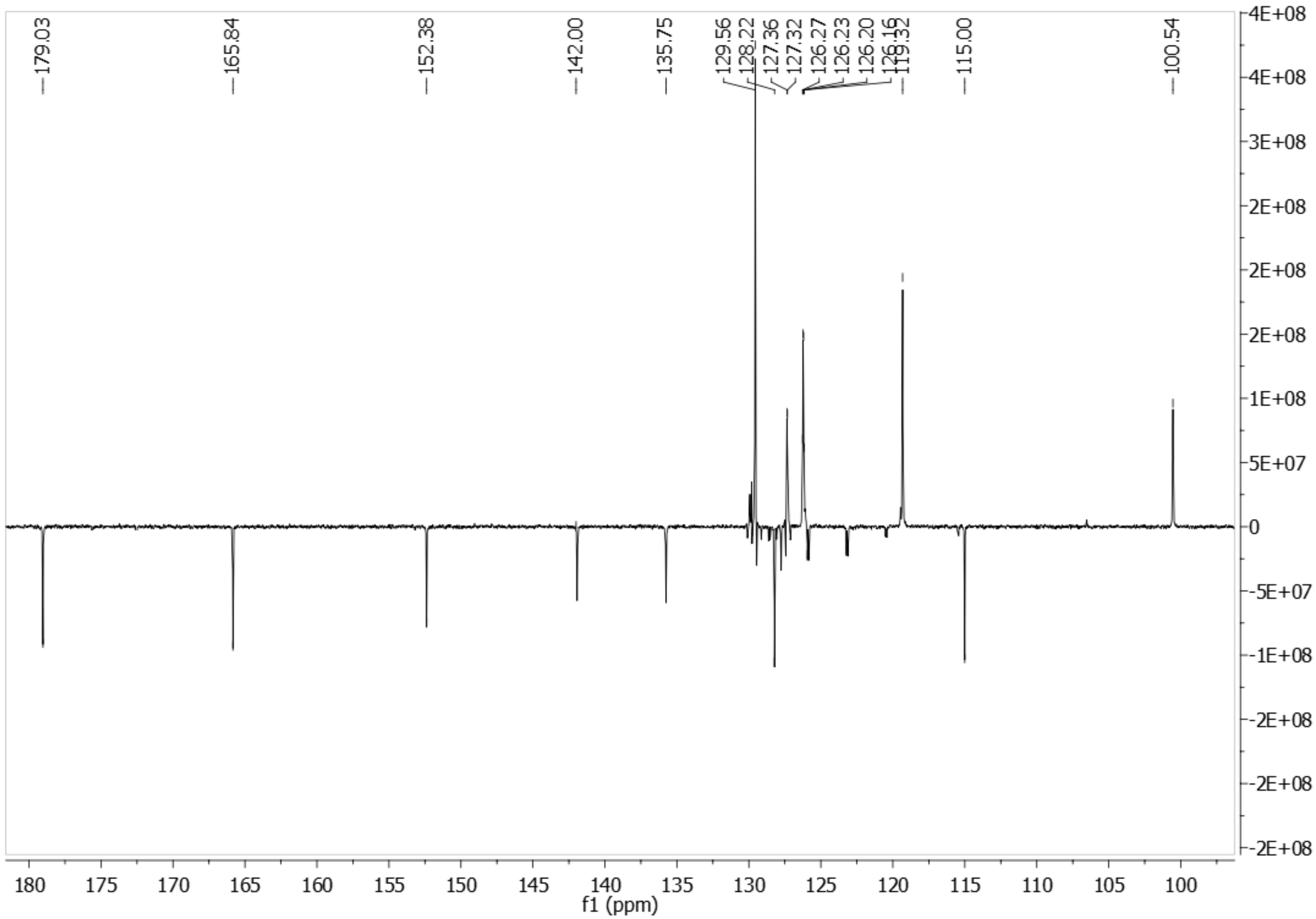

^{13}C APT NMR (DMSO-*d6*, 101 MHz) spectrum of 5-((1,3-bis(4-(trifluoromethyl)phenyl)-1*H*-pyrazol-4-yl)methylene)-2-thioxoimidazolidin-4-one **3p**

Researcher	: Dr.Alaa Zaki	email:	mob.
Assay	: Caspase 3 enzyme assay		
Samples	: 02 samples		
Ref.	: ---		
Date	: 11-07-2023		
Reader	: ROBONIK P2000 ELISA READER wl 450 nm		
Cell lines	: ---		
Kit used	: Invetrogen Casp3 (Active) eia kit		

Lab Report

Ser	Compound				Caspase 3	fld±
	code	mw	cells	IC50 uM	Pg/ml	
1	3i	---	LNCaP	---	358.9	4.667
2	3k	---	LNCaP	---	407.9	5.304
3	3o	---	LNCaP	---	268.4	3.490
4	3j	---	LNCaP	---	316.5	4.115
5	3p	---	LNCaP	---	334.6	4.351
6	3m	---	LNCaP	---	244.3	3.176
7	Enzalutamide	---	LNCaP	---	532.4	6.923
8	control	---	LNCaP	---	76.9	1

Detailed results

STANDARDS	ng/ml
St.1	2500
St.2	1250
St.3	625
St.4	313
St.5	156
St.6	78
St.7	39

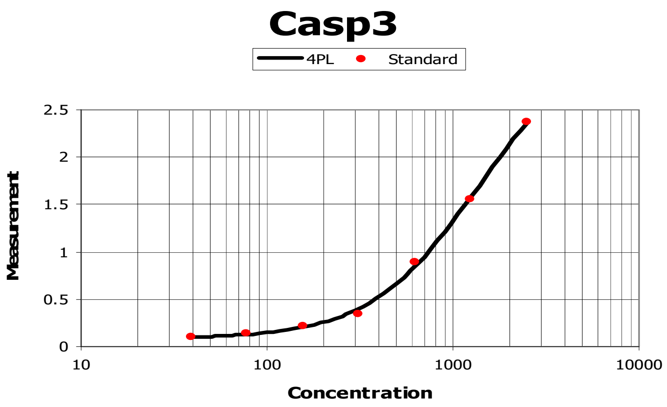

Plate map

	1	2	3	4	5	6	7	8	9	10	11	12
A	st1	**3i**	**3p**	--	--	--	--	--	--	--	--	--
B	St2	**3i**	**3p**	--	--	--	--	--	--	--	--	--
C	st3	**3k**	**3m**	--	--	--	--	--	--	--	--	--
D	st4	**3k**	**3m**	--	--	--	--	--	--	--	--	--
E	st5	**3o**	**Enzalutamide**	--	--	--	--	--	--	--	--	--
F	st6	**3o**	**Enzalutamide**	--	--	--	--	--	--	--	--	--
G	st7	**3j**	**cont**	--	--	--	--	--	--	--	--	--
H	B	**3j**	**cont**	--	--	--	--	--	--	--	--	--

Samples OD results

	1	2	3	4	5	6	7	8	9	10	11	12
A	2.395	0.501	0.453	0	0	0	0	0	0	0	0	0
B	1.584	0.486	0.466	0	0	0	0	0	0	0	0	0
C	0.922	0.572	0.344	0	0	0	0	0	0	0	0	0

D	0.376	0.554	0.332	0	0	0	0	0	0	0	0	0
E	0.241	0.372	0.753	0	0	0	0	0	0	0	0	0
F	0.163	0.367	0.729	0	0	0	0	0	0	0	0	0
G	0.131	0.428	0.227	0	0	0	0	0	0	0	0	0
H	0.026	0.441	0.192	0	0	0	0	0	0	0	0	0

Calibrator	Wells	Conc.	Raw (Corrected)	Backfit	Recovery %
Standard1	A1	2500	2.37	2515	100.6
Standard2	B1	1250	1.56	1224	97.95
Standard3	C1	625	0.896	661.7	105.9
Standard4	D1	312.5	0.35	273.3	87.46
Standard5	E1	156.3	0.215	164.7	105.4
Standard6	F1	78.13	0.137	87.41	111.9
Standard7	G1	39.06	0.105	45.15	115.6

Sample	Wells	Raw	Background Corrected	Conc.	Conc. (Average)	%CV	SD	SEM
3i	A2	0.501	0.467	364.2	358.9	2.1	7.55	5.34
	B2	0.486		353.5				
3k	C2	0.572	0.537	414.2	407.9	2.19	8.93	6.31
	D2	0.554		401.6				
3o	E2	0.372	0.344	270.3	268.4	0.994	2.67	1.89
	F2	0.367		266.5				
3j	G2	0.428	0.409	311.8	316.5	2.11	6.68	4.72
	H2	0.441		321.2				
3p	A3	0.453	0.434	329.9	334.6	1.98	6.61	4.68
	B3	0.466		339.2				
3m	C3	0.344	0.312	249	244.3	2.69	6.57	4.65
	D3	0.332		239.7				
Enzalutamide	E3	0.753	0.715	540.8	532.4	2.24	11.9	8.43
	F3	0.729		524				
control	G3	0.227	0.184	152.1	135.4	17.5	23.7	16.8
	H3	0.192		118.6				
Blank	H1	0.026	0	< Curve	-	-	-	-
